# Retrosynthesis prediction with an interpretable deep-learning framework based on molecular assembly tasks

**DOI:** 10.1038/s41467-023-41698-5

**Published:** 2023-10-03

**Authors:** Yu Wang, Chao Pang, Yuzhe Wang, Junru Jin, Jingjie Zhang, Xiangxiang Zeng, Ran Su, Quan Zou, Leyi Wei

**Affiliations:** 1 School of Software, Shandong University, Jinan, 250101 China; 2 Joint SDU-NTU Centre for Artificial Intelligence Research (C-FAIR), Shandong University, Jinan, 250101 China; 3 College of Computer Science and Electronic Engineering, Hunan University, Changsha, China; 4 College of Intelligence and Computing, Tianjin University, Tianjin, China; 5 Institute of Fundamental and Frontier Sciences, University of Electronic Science and Technology of China, Chengdu, China

**Keywords:** Synthetic chemistry methodology, Cheminformatics, Drug discovery and development

## Abstract

Automating retrosynthesis with artificial intelligence expedites organic chemistry research in digital laboratories. However, most existing deep-learning approaches are hard to explain, like a “black box” with few insights. Here, we propose RetroExplainer, formulizing the retrosynthesis task into a molecular assembly process, containing several retrosynthetic actions guided by deep learning. To guarantee a robust performance of our model, we propose three units: a multi-sense and multi-scale Graph Transformer, structure-aware contrastive learning, and dynamic adaptive multi-task learning. The results on 12 large-scale benchmark datasets demonstrate the effectiveness of RetroExplainer, which outperforms the state-of-the-art single-step retrosynthesis approaches. In addition, the molecular assembly process renders our model with good interpretability, allowing for transparent decision-making and quantitative attribution. When extended to multi-step retrosynthesis planning, RetroExplainer has identified 101 pathways, in which 86.9% of the single reactions correspond to those already reported in the literature. As a result, RetroExplainer is expected to offer valuable insights for reliable, high-throughput, and high-quality organic synthesis in drug development.

## Abstract (web summary)

Automating retrosynthesis prediction in organic chemistry is a major application of ML. Here the authors present RetroExplainer, which offers a high-performance, transparent and interpretable deep-learning framework providing valuable insights for drug development.

## Introduction

Retrosynthesis aims to identify a set of appropriate reactants for the efficient synthesis of target molecules, which is indispensable and fundamental in computer-assisted synthetic planning^1–3^. Retrosynthetic analysis was formalized by Corey^4–6^ and solved by the Organic Chemical Simulation of Synthesis (OCSS) program. Later, driven by sizeable experimental reaction data and significantly increased computational capabilities, various machine-learning-based approaches^7^, especially deep-learning (DL) models, have been proposed and achieved incremental performance^8^.

In the early age of data-driven retrosynthesis, researchers primarily focused on developing template-based retrosynthesis approaches that rely on a reaction template to transform products into reactants^9,10^. Among these approaches, molecular fingerprints with the multi-layer perceptron are often used to encode molecular products and recommend reasonable templates. For instance, Segler et al.^9^. utilized extended-connectivity fingerprints (ECFPs)^11^ with an expansion policy network to guide the template search, whereas Chen et al.^10^. adopted a strategy similar to a single-step retrosynthesis predictor for their neural-guided multi-step planning. However, the process of constructing reaction templates currently relies on manual encoding or complex subgraph isomorphism, making it difficult to explore potential reaction templates in vast chemical space. To address these issues, template-free and semi-template methods have emerged as promising alternatives, utilizing molecular fingerprints to obtain molecular-level representations. Chen et al.^12^. introduced the FeedForward EBM (FF-EBM) method, complemented by template-free models. FF-EBM leverages the fingerprinting technique to prioritize potential precursors. In addition to molecular fingerprints, existing template-free and semi-template approaches can be generally categorized into two classes: (1) sequence-based approaches^13–15^ and (2) graph-based approaches^16–18^. The two classes of the method mainly differ in the strategies of molecular representations; the molecules are usually represented as linearized strings for sequence-based approaches^13–15^, and as molecular graph structures for graph-based approaches^16–18^.

Sequence-based approaches have been used to represent target product molecules using serialized notations, such as SMILES (simplified molecular-input line-entry system)^19^. Liu et al.^20^. introduced the Seq2Seq model, which includes a bidirectional long short-term memory (LSTM)^21^ encoder and decoder for retrosynthetic translation. As with neural machine translation models, like Transformer^22^, sequence-based retrosynthetic approaches have gradually improved in performance. Karpov et al.^23^. adapted the Transformer architecture with modified learning rate schedules and snapshot learning for retrosynthesis analysis, and Tetko et al.^13^. proposed a Transformer-based retrosynthetic model with SMILES augmentation that improved performance. With the rise of the pretraining-finetuning paradigm, Irwin et al.^24^. proposed MolBART, which uses large-scale self-supervised pretraining to speed up the convergence of retrosynthesis tasks. However, there are two limitations: (1) the linearized molecule representations, like SMILES, are difficult to explore the direct structural information and atomic properties that are crucial for retrosynthesis analysis, and (2) the SMILES-based molecular representation approach is grammatically strict and not semantically valid, easily leading to frequent invalid syntaxes. To address these limitations, other approaches have been proposed to avoid invalid strings^25–27^ or embed more abundant structural information^28^.

Graph-based approaches are commonly used to represent molecules through graph structures, which are used to predict changes in the target molecule and infer the reactants. This is usually done through a two-stage paradigm that involves reaction center prediction (RCP) and synthon completion (SC). Initially, this idea was used in forward reaction prediction by Jin et al.^29^, who proposed using the Weisfeiler−Lehman isomorphism test^30^ and graph learning to predict reaction outcomes. With the development of graph neural networks (GNNs), many GNN-based frameworks have emerged for retrosynthesis and have achieved notable improvements in performance. For example, Shi et al.^17^. presented the G2G framework, which utilizes relational graph convolution network (R-GCN)^31^ for RCP and reinforcement learning for SC. Following the same paradigm, Yan et al.^32^. and Somnath et al.^33^. devised RetroXpert and GraphRetro, respectively; the former applied a graph attention network (GAT)^34^ variant for RCP and a sequence-based Transformer for SC, whereas the latter designed two massage passing neural networks (MPNNs)^35^ for the two stages. Different from the above approaches, Dai et al.^36^. proposed GLN, a method that leverages reaction templates to connect products and reactants. Nevertheless, traditional GNNs merely focus on the local structures of molecules, neglecting the effect of long-distance characteristics (e.g., Van der Waals force). To solve this problem, Ying et al.^37^. proposed Graphormer, introducing a shortest-path-based method for multi-scale topological encoding. In addition to the above methods directly modeling graph changes, there are other graph-based approaches that predict graph changes by translating reactants^18,38,39^.

Although existing retrosynthesis approaches have achieved significant progress in accelerating data-driven retrosynthesis prediction, they still suffer from the following intrinsic problems: (1) sequence-based approaches suffer from the loss of prior information about the molecules. Meanwhile, graph-based approaches neglect sequential information and long-range characteristics. Both approaches are constrained in feature representation learning, limiting further performance improvement. (2) Many of the existing DL-based retrosynthesis approaches face the problem of poor interpretability. Although some of them (e.g., template-based approaches) provide human-understanding predictions (since templates can be linked to literature precedents), the decision-making mechanism of the existing models remains unclear, which remarkably restricts the model’s reliability and practical applications. Importantly, they fail to explain how the models work or to provide the substantive insights. (3) Most existing approaches focus on the single-step retrosynthesis prediction that enables generating plausible reactants but is perhaps not purchasable and which are usually accompanied by a tedious process of hand-picking predictions. Therefore, the multi-step retrosynthesis prediction with pathway planning from products to accessible reactants is much more meaningful for experimental researchers in practical chemical synthesis.

**Fig. 1:**
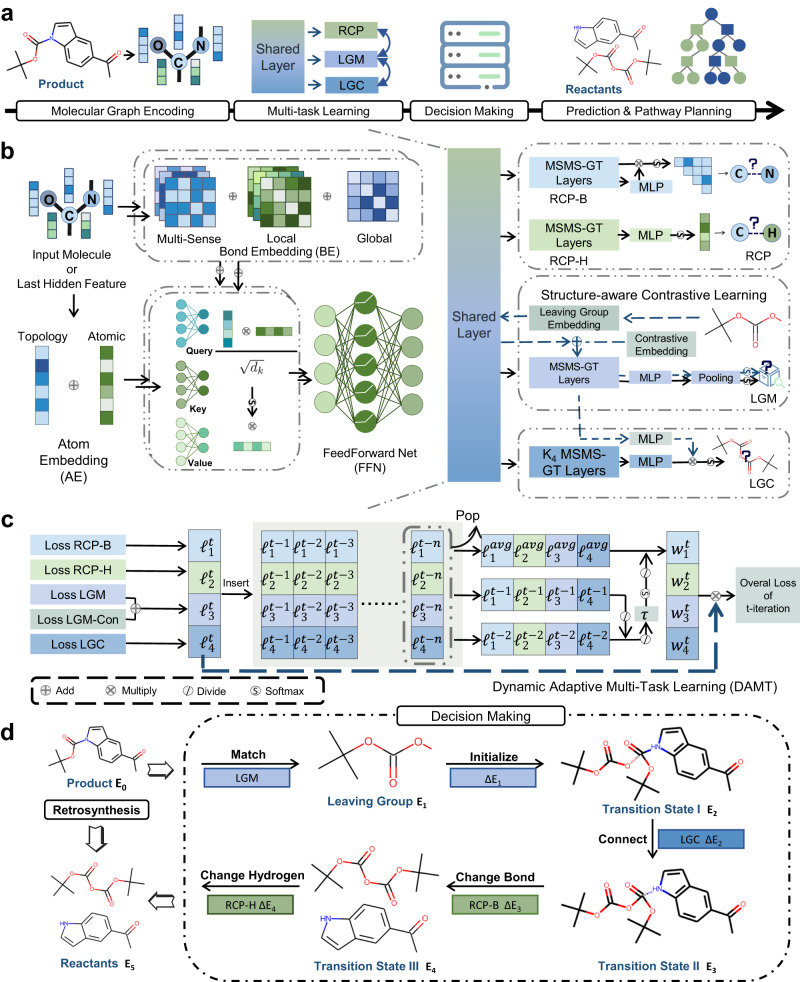
Overview of RetroExplainer. **a** The pipeline of RetroExplainer. We formulated the whole process as four distinct phases: (1) molecular graph encoding, (2) multi-task learning, (3) decision-making, and (4) prediction or multi-step pathway planning. **b** The architecture of the multi-sense and multi-scale Graph Transformer (MSMS-GT) encoder and retrosynthetic scoring functions. We considered the integration of multi-sense bond embeddings with both local and global receptive fields, blending them as attention biases during the self-attention execution phase. Upon obtaining shared features, we employed three distinct modules to evaluate the probabilities of five retrosynthetic events. These comprise: the reaction center predictor (RCP), which includes both a bond change predictor (RCP-B) and a hydrogen change evaluator (RCP-H); the leaving group matcher (LGM), enhanced with an additional contrastive learning strategy; and the leaving group connector (LGC). It is noteworthy to mention that the acronym MLP stands for multi-layer perceptron. **c** The dynamic adaptive multi-task learning (DAMT) algorithm. This algorithm is intended to acquire a group of weights according to the descent rates of losses and their value ranges to optimize the five evaluators equally. ${l}_{i}^{t}$ denotes the $i$ th kind of loss score in the $t$ th iteration. The ${l}_{i}^{{avg}}$ means the average of $i$ th type of loss value over the loss queue from ${l}_{i}^{t}$ to ${l}_{i}^{t-n}$, where $n$ is the length of queue we take into consideration. ${w}_{i}^{t}$ is the obtained weight of the $i$ th kind of loss score at the $t$ th iteration.$\tau$ is a temperature coefficient. d. The chemical-mechanism-like decision process. We designed a transparent decision process with six stages, assessed by five evaluators to obtain the energy scores ${E}_{1},\ldots {E}_{5}$. The $\Delta {E}_{i}$ is the gap between the ${E}_{i}$ and ${E}_{i+1}$.

In this study, we propose RetroExplainer, a chemical knowledge and DL-guided molecular assembly approach for retrosynthesis prediction with quantitative interpretability. The overall framework of the proposed approach is shown in Fig. 1. The contributions are generalized as follows:

- For a robust and informative molecular representation, we proposed a multi-sense and multi-scale Graph Transformer (MSMS-GT) for generalized molecular representation learning, dynamic adaptive multi-task learning (DAMT) for balanced multi-objective optimization, and structure-aware contrastive learning (SACL) for molecular structural information capturing. Results demonstrated that RetroExplainer performed exceptionally well on almost all of the 12 large-scale benchmark datasets, including three commonly used datasets (USPTO-50K, USPTO-FULL, and USPTO-MIT), and nine newly constructed datasets using molecular similarity splitting methods.
- For good interpretability, we introduced an energy-based molecular assembly process that offers transparent decision-making and interpretable retrosynthesis predictions. This process can generate an energy decision curve that breaks down predictions into multiple stages and allows substructure-level attributions; the former can help understand the “counterfactual” predictions to discover potential biases from datasets, and the latter can provide more granular references (such as the confidence of a certain chemical bond being broken) to inspire researchers to design customized reactants.
- To ensure the synthesizability of the product and avoid the tedious manual selection of candidate reactants, we integrated the proposed model with the Retro*^10^ algorithm and used it to predict the synthetic routes of 101 complex drug molecules. To validate the effectiveness of these routes, we used the SciFinder^n^ search engine^40^ for similar reaction searches, and the results showed that 86.9% of single-step reactions could correspond to literally reported reactions.

## Results

### Performance comparison on USPTO benchmark datasets

To assess the effectiveness of RetroExplainer, we compared it with 21 comparative retrosynthesis approaches on three commonly used USPTO benchmark datasets (USPTO-50K, USPTO-FULL, and USPTO-MIT). To ensure a fair comparison, we employed the same data-splitting method as the previous studies for model training and evaluation^32,36,41^.

Table 1 displays the predictive performance of our RetroExplainer and other existing approaches on the USPTO-50K dataset. The performance was evaluated using the top-*k* exact-match accuracy, with *k* set to 1, 3, 5, and 10. Compared to sequence-based and graph-based approaches, RetroExplainer achieved the optimal level in five out of nine metrics when *k* equals 1, 3, 5, and 1, 3 for known and unknown reaction types, respectively. Although our model did not achieve the optimal accuracy when *k* is equal to 10, the accuracy is close to that of the optimal model - LocalRetro, with a difference of only 0.2% and 1% under reaction class known and unknown, respectively. Moreover, considering the accuracies within the two scenarios outlined above (contingent on the provision of the reaction class), averaged across top-1, top-3, top-5, and top-10 predictions, our model achieved the highest accuracy, with a difference of 1% and 0.1% compared to the runner-up models, namely LocalRetro for known reaction types and R-SMILES for unknown reaction types, respectively.

**Table 1:** Performance of our RetroExplainer and the state-of-the-art methods on USPTO-50K benchmarks

Model	Top-*k* accuracy (%)							
	Reaction class known				Reaction class unknown			
	k = 1	3	5	10	1	3	5	10
**Fingerprint-based**								
RetroSim^41^	52.9	73.8	81.2	88.1	37.3	54.7	63.3	74.1
NeuralSym^8^	55.3	76.0	81.4	85.1	44.4	65.3	72.4	78.9
**Sequence-based**								
SCROP^59^	59.0	74.8	78.1	81.1	43.7	60.0	65.2	68.7
LV-Transformer^23^	-	-	-	-	40.5	65.1	72.8	79.4
AutoSynRoute^60^	-	-	-	-	43.1	64.6	71.8	78.7
TiedTransformer^61^	-	-	-	-	47.1	67.1	73.1	76.3
MolBART^62^	-	-	-	-	55.6	-	74.2	80.9
Retroformer^63^	64.0	82.5	86.7	90.2	53.2	71.7	76.6	82.1
RetroPrime^64^	64.8	81.6	85.0	86.9	51.4	70.8	74.0	76.1
R-SMILES^65^	-	-	-		56.3	**79.2**	**86.2**	91.0
DualTF^46^	65.7	81.9	84.7	85.9	53.6	70.7	74.6	77.0
**Graph-based**								
GLN^36^	64.2	79.1	85.2	90.0	52.5	69.0	75.6	83.7
G2Gs^17^	61.0	81.3	86.0	88.7	48.9	67.6	72.5	75.5
G2GT^18^	-	-	-	-	54.1	69.9	74.5	77.7
GTA^16^	-	-	-	-	51.1	67.6	73.8	80.1
GraphRetro^33^	63.9	81.5	85.2	88.1	53.7	68.3	72.2	75.5
Graph2SMILES^39^	-	-	-	-	52.9	66.5	70.0	72.9
RetroXpert^32^	62.1	75.8	78.5	80.9	50.4	61.1	62.3	63.4
GET^38^	57.4	71.3	74.8	77.4	44.9	58.8	62.4	65.9
LocalRetro^57^	63.9	86.8	92.4	**96.0**	53.4	77.5	85.9	**92.4**
RetroExplainer (**Ours**)	**66.8**	**88.0**	**92.5**	95.8	**57.7**	**79.2**	84.8	91.4

The performance regarding existing methods is derived from their references. The best-performing results are marked in bold.

**Fig. 2:**
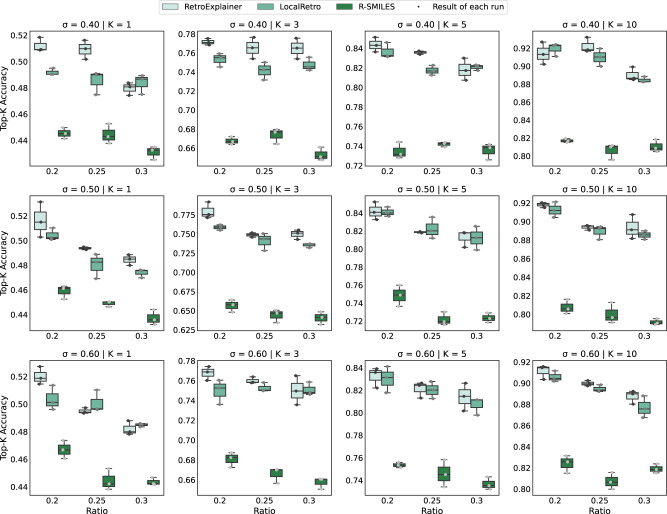
Performance comparison on the USPTO-50K dataset with Tanimoto similarity splits. The sub-figures represent the top-k accuracies (k = 1, 3, 5, 10) of our RetroExplainer and the existing methods on the USPTO-50K dataset. These are measured under various similarity thresholds for input molecule outcomes ($\sigma$ = 0.4, 0.5, 0.6) and different splitting ratios (0.2, 0.25, and 0.3) for the combined proportions of the validation and test set, respectively. Each result was derived from three repeated experiments conducted with distinct random seeds. The minimum, maximum, and median of the three data points are represented by the lower whisker, upper whisker, and central line within each box, respectively. Source data are provided as a Source Data file.

The current random splitting method of datasets often results in scaffold evaluation bias^42^. In the random splitting datasets, very similar molecules might be present in both the training and test sets, easily leading to information leakage of the training dataset and the production of bias in the model evaluation. To overcome the potential bias and validate the robustness of our method, we utilized the Tanimoto similarity splitting method proposed by Kovács et al. ^43^. for the USPTO-50K dataset. Specifically, we considered nine data splitting types with three degrees of similarity threshold (i.e., 0.4, 0.5, and 0.6) and three degrees of splitting ratio (i.e., 0.2, 0.25, and 0.3) for the test set, thus yielding nine Tanimoto similarity-based datasets. We evaluated and compared our RetroExplainer with the existing approaches on the nine datasets. To simplify the comparison, we selected only R-SMILES and LocalRetro as controls because they are the top-2 best-performing methods among the existing methods. Figure 2 illustrates the comparative results of our RetroExplainer, R-SMILES, and LocalRetro on the datasets, respectively. It can be seen from Fig. 2 that our RetroExplainer outperformed the top-1, -3, -5, and -10 accuracies of the benchmark controls on most of the nine datasets. This further demonstrates the effectiveness and robustness of RetroExplainer. Moreover, the results also demonstrate that our model has stronger domain adaptivity for unseen molecules with scaffolds compared to the existing approaches.

To further validate the effectiveness and robustness of RetroExplainer, we compared it with the state-of-the-art approaches on two much larger benchmark datasets (USPTO-FULL and USPTO-MIT). Table 2 presents the predictive results. As can be seen, RetroExplainer achieved the best performance with a large margin in all four metrics (top-1 accuracy, top-3 accuracy, top-5 accuracy, and top-10 accuracy) on both datasets. More specifically, RetroExplainer outperformed the runner-up R-SMILES by 2.5%, 4.1%, 2.7%, and 2.8% in top-1 accuracy, top-3 accuracy, top-5 accuracy, and top-10 accuracy on the USPTO-FULL benchmark, respectively; similar results can be observed on the USPTO-MIT dataset. The results demonstrate that RetroExplainer is more effective and robust for the conditions when evaluated on larger datasets and thus has more potential for large-scale training scenarios compared to previous state-of-the-art methods. Furthermore, ablation studies in Supplementary Information Note 1 on scale information and augmentation strategy discuss the effectiveness of these modules. Additionally, we provide a case study illustrating how MSMS-GT focuses on multi-scale molecular structures, which can be seen in Supplementary Information Note 2.

**Table 2:** Performance of our RetroExplainer and the state-of-the-art methods on USPTO-FULL and USPTO-MIT benchmarks

Model/Dataset	Top-k accuracy (%)			
	Reaction class unknown			
	k = 1	3	5	10
**USPTO-FULL**				
^F^RetroSim^41^	32.8	-	-	56.1
^F^NeuralSym^8^	35.8	-	-	60.8
^G^GLN^36^	39.3	**-**	-	63.7
^S^RetroPrime^64^	44.1	-	-	68.5
^G^RetroXpert^32^	49.4	63.6	67.6	71.6
^S^R-SMILES^65^	48.9	66.6	72.0	76.4
^G^RetroExplainer (**Ours**)	**51.4**	**70.7**	**74.7**	**79.2**
**USPTO-MIT**				
^F^RetroSim^41^	47.8	67.6	74.1	80.2
^S^AutoSynRoute^60^	54.1	71.8	76.9	81.8
^S^RetroTRAE^28^	58.3	-	-	-
^S^R-SMILES^65^	**60.3**	78.2	83.2	87.3
^G^LocalRetro^57^	54.1	73.7	79.4	84.4
^G^RetroExplainer (**Ours**)	**60.3**	**81.6**	**86.4**	**90.5**

^S^: Denotes sequence-based models.

^G^: Denotes graph-based models.

^F^: Denotes finger-prints-based models. The best-performing results are marked in bold.

Compared to previous retrosynthetic models like RetroXpert, G2G, and GraphRetro, RetroExplainer achieves superior performance perhaps due to its distinctive approach to data modeling. To guide the three scoring modules, RetroExplainer aims to simultaneously capture two types of distributions: the joint conditional distribution of RCs and LGs when the molecular graph of the product is provided, as well as the conditional distribution of connections among LGs when RCs, LGs, and the product are determined. The former distribution enables more informative representations that consider the influence of LGs on RCs through the joint distribution. This enhancement also improves the model’s generalization capability, especially in scenarios involving multiple RCs and LGs. This improvement circumvents the need for excessively iterative processes arising from multi-LG (and multi-RC) issues. Furthermore, the presence of multiple LGs presents challenges for conventional manual coding approaches in handling the connections between LGs and the current synthon. However, by learning from the conditional distribution of these connections, RetroExplainer adapts effectively to larger and more intricate datasets. More detailed comparisons can be found in Supplementary Information Note 3.

### RetroExplainer provides interpretable insights

**Fig. 3:**
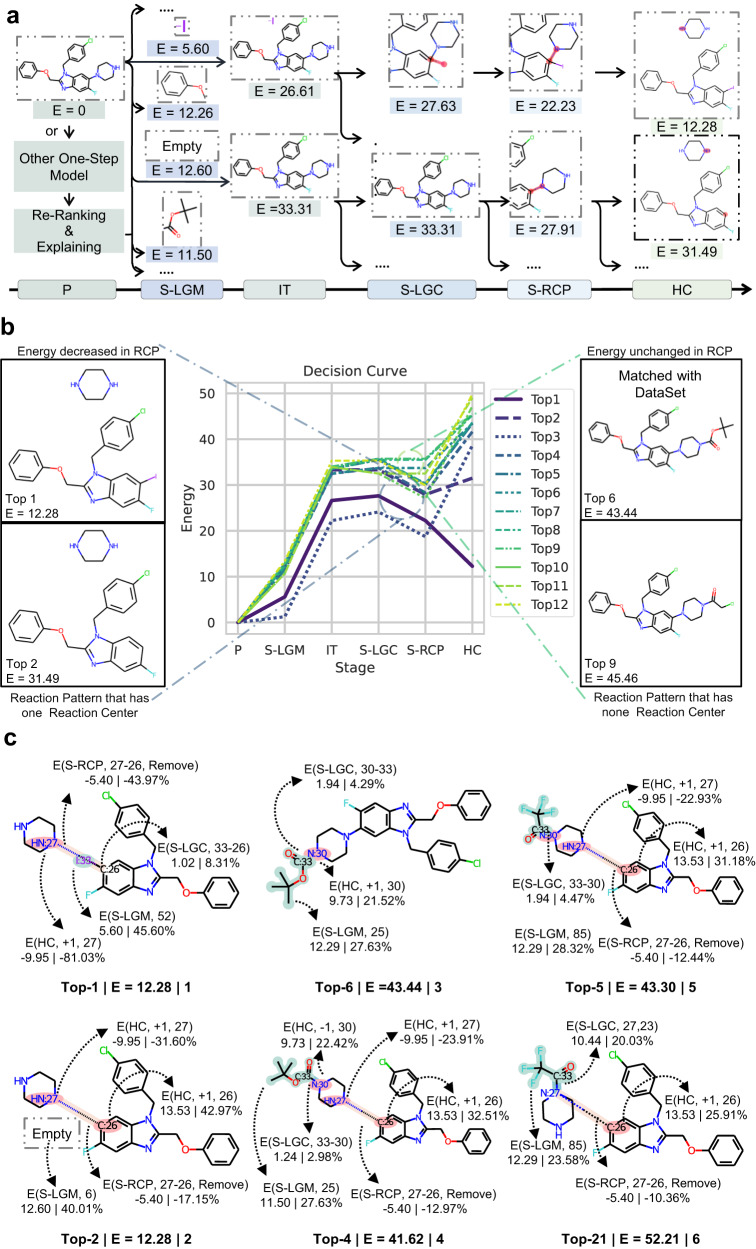
Generated explanations through a decision process based on molecular assembly. **a** The searching routes of two predictions, including reactions with and without leaving groups. **b** The decision curve of top-12 predictions by RetroExplainer. The same reaction patterns have the same gaps of energy. **c** Nine representative instances for substructure attributions, which allows a granular insight.

Inspired by the ${S}_{N}2$ mechanism^44^, we designed a transparent decision process via DL-guided molecular assembly for the interpretable retrosynthesis prediction. The decision process consists of six stages, as illustrated in Fig. 3a, which include the original product (P), leaving group matching (S-LGM), initializing (IT), leaving group connecting (S-LGC), reaction center bond changing (S-RCP), and hydrogen number changing (HC). The decision process generates the energy scores for each stage based on their contributions to the final decision. The energy scores are determined by the learned modules, such as LGM, reaction center prediction for attached hydrogen (RCP-H), reaction center prediction for bond (RCP-B), and LGC; see Supplementary Information Note 4 for more details on the calculation of energy scores. The six-stage decision process is described as follows (see Fig. 3a).

1. We started from the P stage and initially set the energy score of the stage (denoted as $E$ in Fig. 3a) as 0.
2. At S-LGM, we selected a leaving group (LG) assigned with an energy score based on the predicted probabilities given by the LGM module.
3. Afterward, we calculated the energy of the IT stage by adding the energy of the selected LG at the S-LGM stage and the probabilities of the corresponding event predicted by the RCP-H, RCP-B, and LGM modules, respectively. It’s important to note that this description encompasses two distinct stages: the S-LGM stage and the S-RCP stage.
4. We then used a dynamic programming algorithm to expand all possible nodes in the search tree during the LGC and RCP stages. We selected the events with probabilities larger than a preset threshold and fixed the energy scores of the stages accordingly.
5. Finally, we adjusted the hydrogen number and formal charge for each atom to ensure that the modified molecular graph obeyed the valence rule, and then we calculated the final energy scores based on the cost of HC.

The decision curve generated by our RetroExplainer provides insights into why the model may make “incorrect” predictions (chemically reasonable but not matching the ground truth recorded in the dataset) and also helps to identify potential biases in the training dataset.

As shown in Fig. 3b, the correct answer for synthesizing the target product by amine deprotecting is ranked top-6 by our model, while the top-1 prediction is for C-N coupling. The HC stage is found to be the key stage causing the difference in energy score, where RetroExplainer tends to predict reactants with an increase in hydrogen number of the amine, indicating the presence of HC bias for similar molecular scaffolds. Additionally, the straight lines with two slopes observed in the interval between LGC and RCP of the top-12 predictions suggest that the reaction patterns differ in two types of reaction centers (RCs) in the RCP, whether the C-N bond is broken (synthesizing or decomposing). RetroExplainer understands that the synthesizing pattern is beneficial for minimizing the energy score, and the corresponding slope of the line in the LGC−RCP interval is calculated as negative, explaining why the difficult reaction in which the hydrogen atom in the benzene ring is removed is ranked as top-2. Moreover, the removed hydrogen atom in the benzene ring results in a significant difference in energy compared with the top-1 prediction, an easier reaction to synthesize the target product, indicating the correlation between the decision curve and the reaction difficulty. More examples of RetroExplainer’s performance can be found in Supplementary Information Note 5.

By analyzing the percentage of energy contributed by each stage to the final energy score, the molecular assembly process offers ability to attribute at the substructure level. This is necessary because in cases with multiple RCs, changes in the number of multiple chemical bonds or multiple atomic hydrogens are often merged into the same stage, causing some ambiguity. Figure 3c displays six representative instances, which offer granular references.

Through the comparison between the top-1 and top-2 predictions (molecules 1 and 2 in Fig. 3c), we can discover that the energy scores can be potentially associated with the difficulty of the reactions, such as selectivity. The argument is that although the connection between I:33 and C:26 is not conducive to the reduction of energy, the energy increase by the addition of one hydrogen atom at C:26 is roughly 13 times larger than the former energy (13.5 and 1.02). Our finding is consistent with a previous study in which the C–N cross-coupling reaction usually involves a specific catalyst and selectivity problems^45^. Furthermore, the I:33 weakens this selectivity issue, which corresponds to the fact that the prediction for molecule 1 is assigned less energy than that for molecule 2.

By comparing molecules 3, 4, and 5, we can conclude that the reason the correct answer’s energy score is overestimated is that the model prefers to break the chemical bond between C:26 and N:27, which can bring about a benefit of 5.40 for bond cleavage and 9.95 for the decrease in energy score resulting from increasing the number of hydrogen atoms at N:27 by one. These two forms of energy reduction can completely offset the impact of the increase in energy caused by the increase in hydrogen atoms at C:26. Therefore, the correct answer from molecule 3 was ranked after predictions in molecule 2 and molecule 5 because it did not benefit from the energy decrease brought by breaking the C:26 and N:27 chemical bonds.

Another interesting phenomenon is that we observed the influence of steric hindrance on our model’s reasoning, which may imply that our model can learn some rules similar to reaction mechanisms. Comparing molecule 4 and molecule 6, their molecular structures are identical, but different energy scores were predicted. The only difference is that the LG is connected to a symmetric but differently numbered N. Although this leads to a change in the number of hydrogen atoms at N:27 and N:30 in the prediction from molecule 6, they can cancel each other out, and the sum of their effects on the energy value is only -0.53%. In contrast, the most important factor is the energy change brought by the chemical bond C:33−N:30 and the chemical bond C:33−N:27; the former is almost one-tenth of the latter. This is because the latter connection occurs before the chemical bond is broken, and the connection between C:30 and N:27 will cause greater steric hindrance than N:30, ultimately leading to an increase in energy. This explains why this prediction from molecule 6 was ranked as top-21.

**Fig. 4:**
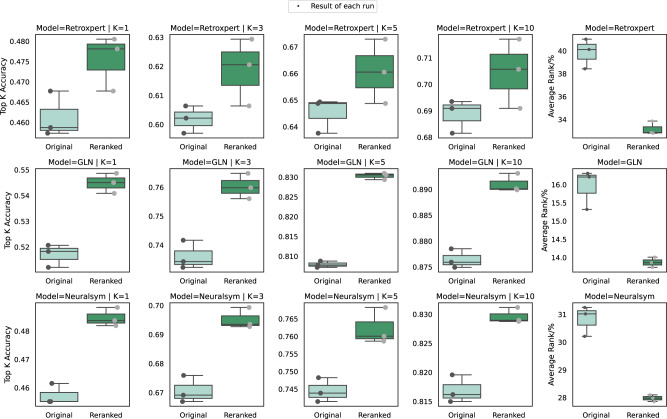
Re-ranking performance of RetroExplainer. The box plots depict re-ranking results in terms of top-1, -3, -5, -10 accuracy, and percentile average rank in comparison to three standard retrosynthesis models: Retroxpert, GLN, and Neuralsym. Each result was derived from three repeated experiments conducted with distinct random seeds. The minimum, maximum, and median of the three data points are represented by the lower whisker, upper whisker, and central line within each box, respectively. Source data are provided as a Source Data file.

Using the energy-based process as demonstrated in Fig. 3a, RetroExplainer can also re-rank the predictions made by other existing approaches (to improve comprehension, we offer the pseudocodes of the re-ranking algorithm in Supplementary Information Note 6), and the results for the predictions (candidate reactants) made by other existing approaches as well. To evaluate the re-ranking ability of our model, we re-ranked the predictions generated by three different retrosynthesis approaches (RetroXpert, GLN, and NeuralSym). They were chosen because of the relative ease by which their prediction results are obtained^46^. For each target product, we selected the top 50 predictions for re-ranking, and we introduced an evaluation strategy based on the average percentage rank to figure out the problem that many predictions have less than 50 results. The re-ranking results in terms of the top-1, -3, -5, and -10 accuracies are shown in Fig. 4, and indicate a significant improvement in prediction accuracy. These results suggest that RetroExplainer has a strong re-ranking ability that can improve the predictions of other existing methods.

### Extending RetroExplainer to retrosynthesis pathway planning

**Fig. 5:**
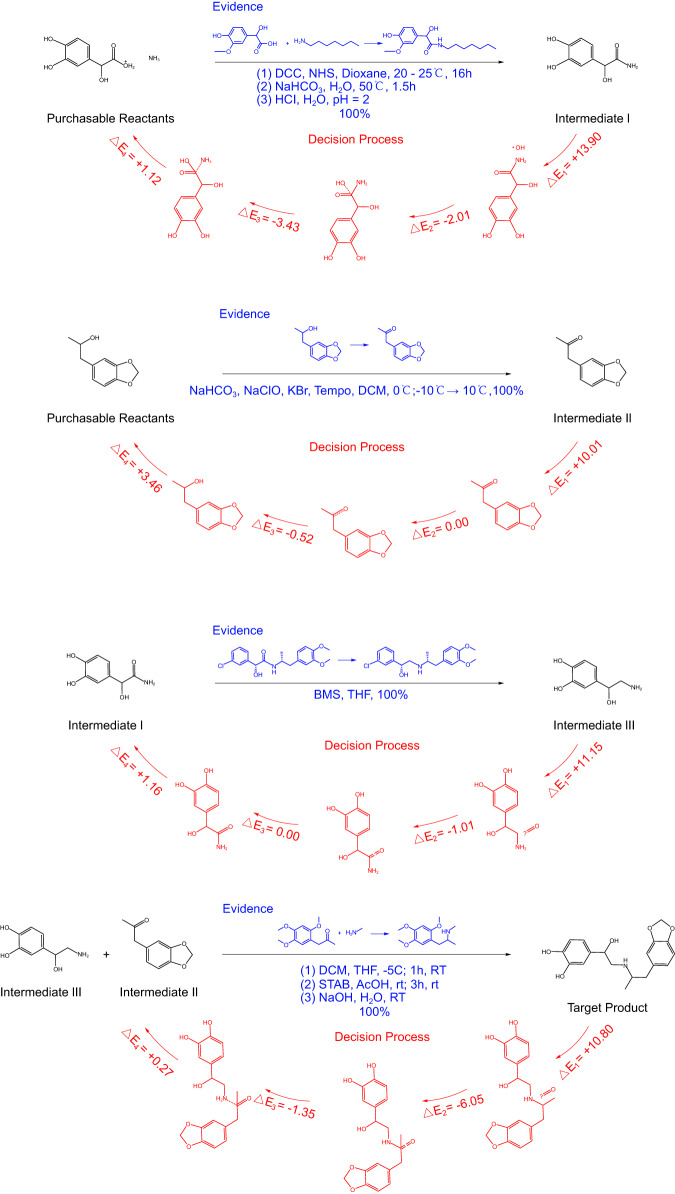
Retrosynthesis planning for protokylol has been carried out by RetroExplainer. A four-step synthetic route to protokylol is presented. In each step, the portion of the text in blue highlights the documented evidence supporting the reaction, whereas the red section describes the decision-making process of our model. Key abbreviations include: DCC for N,N’-Dicyclohexylcarbodiimide; NHS for N-Hydroxysuccinimide; DCM for Dichloromethane; BMS for Dimethyl sulfide borane; THF for Tetrahydrofuran; STAB for Sodium triacetoxyborohydride; RT for Room Temperature; and $\Delta E$ represents the change in energy scores value.

In order to improve the practicality of our RetroExplainer for pathway planning, we incorporated our model with the Retro*^10^ algorithm along with a list of purchasable molecules. To be specific, the single-step model of Retro* was replaced by our RetroExplainer. To illustrate the explanations provided by RetroExplainer, we used protokylol as an example. Protokylol is a β-adrenergic receptor agonist used as a bronchodilator. As shown in Fig. 5, our RetroExplainer devised a four-step synthetic route for protokylol. The energy scores of the decision process illustrate the key sub-processes that support RetroExplainer in making the corresponding predictions. To further demonstrate the practicality of our proposed scheme, we conducted a literature search to find evidence for each reaction step. Although many of the proposed reactions could not be found, we were able to find similar reactions with high yields that matched the proposed reactions. These reactions were found in articles by Ley et al. ^47^, Nair et al.^48^, Roberto et al.^49^, and Neudörffer et al.^50^, respectively. Moreover, we also provide 101 cases for pathway planning containing 176 single steps, in which 153 single-step predictions can be found through a SciFinder^n^ engine search^40^ with similar reaction patterns. For further information regarding the experimental setups, results of multi-planning routes, and the findings from literature searches, please refer to Supplementary Information Note 7, Supplementary Data 1, and Supplementary Data 2, respectively.

### Influence of reaction types

**Fig. 6:**
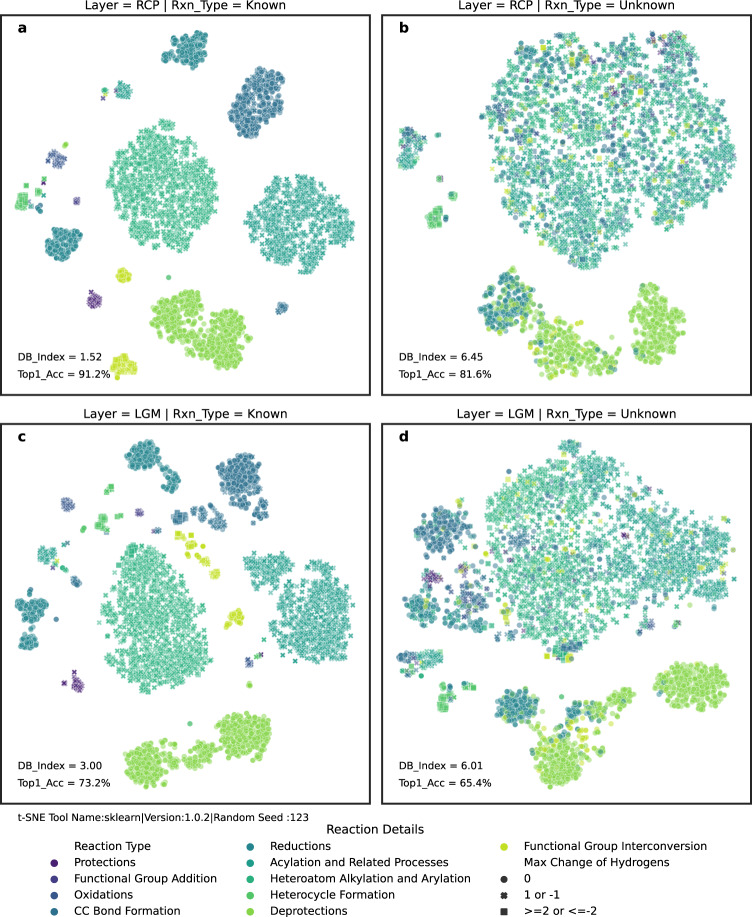
Distributions of t-SNE from hidden layers of RetroExplainer. **a**–**d** Hidden features are derived based on two criteria: (1) whether the reaction type is recognized, and (2) the origin of the hidden features (either from the reaction center predictor, RCP, layer, or the leaving group matcher, LGM, layer). Subsequently, these features are reduced to two dimensions using t-SNE techniques. Distinct colors represent various reaction types, while diverse styles are assigned based on the maximum count of hydrogen alterations.

In contrast to the circumstance where the reaction type is unknown, we add extra embeddings into a super node when the reaction type is given. This super node is then extracted as a graph-level representation after $L$ message-aggregation layers. Table 1 illustrates the increase in top-*k* accuracy when the reaction type is introduced. To investigate how the reaction type affects the performance of RetroExplainer, we extracted four types of hidden features based on their sources (from the last RCP layer or last LGM layer) and whether they are informed of the reaction type. The labels of the reaction type color the distributions of the compressed hidden features through t-SNE (t-distributed Stochastic Neighbor Embedding) in Fig. 6. It is evident that the reaction type imposes a more regular constraint on the hidden representation of the product than being free from reaction type limits. This enhances the location process of RCs and the matching procedure of LGs.

Furthermore, we find different clusters in the same reaction type from Fig. 6a, c, indicating that some reaction types can be further divided into more advanced classes depending on the specific task. For example, functional group interconversion (FGI) is divided into two clusters in Fig. 6a and three clusters in Fig. 6c, depending on their maximum change in the number of attached hydrogens from products to reactants. When the reaction type is not given (Fig. 6b, d), we can still obtain a rough outline relating to the maximum change in hydrogen count, which generalizes the similarities between different classes from a task perspective. Notably, according to the Davies−Bouldin (DB) Index^51^, with the reaction type as a label, the more similar the distributions are, the higher the accuracy of the task (from 81.6% to 91.2% on RCP and from 65.4% to 73.2% on LGM) is. Therefore, the results indicate that an effective way to improve the performance of the reaction-type-unknown model might be to impose extra constraints for hidden features only during the training stage (e.g., SupContrast^52^ and our contrastive technique for LGs). These constraints improve the fitting ability within the domain of the training sets. However, the extra regular loss functions might also increase the risk of overfitting, which means the degradation of generalization to samples out of the dataset distributions. Additionally, to reduce the effect of randomness on the clusters, we added nine t-SNE plots using different random seeds in Supplementary Information Note 8.

## Discussion

Although RetroExplainer achieves impressive performance and interpretability, there are several limitations in our proposed method that deserve further research in the future.

*Limited performance in predicting rare LGs*. The LGM module is designed as a predictive classifier on a pre-collected LGs database, which simplifies the difficulties in LGs generation from Eq. (S3.5) in Supplementary Information Note 3 and guarantees the legality of LGs by imposing a strong prior (that is, all the LGs come from our pre-collected database). However, our LGM method is limited in its flexibility in generating rare LGs, which is a common challenge for many data-driven models that are essentially limited by the quality of the training set. Even translation-based models are not as robust for predicting common LGs to generate unseen LGs. In addition, in our model, although we adopt several strategies like SACL that allow the LGM module to find similar LGs from the database as the agency of unseen LGs during the re-ranking exogenous reactants mode (can be referred to the algorithm in Supplementary Information Note 6) and enlarging the LGs database (in USPTO-FULL we collected about 70,000 LGs), we cannot entirely eliminate this bias (some cases are displayed in Supplementary Information Note 9). Therefore, several DL techniques, such as pretraining on LGM, meta-learning, active learning, and data augmentations, might be promising to be introduced for deep retrosynthetic learning to boost the robustness against rare LGs predictions.

*Limited flexibility of decision process.* We proposed a ${S}_{N}2$-like molecular assembly process to generate decision actions with energies for retrosynthesis predictions. However, this process is so fixed that some decision actions do not intuitively accord with other distinct reaction types, although it does work. For instance, reaction types without LGs are the pecial cases that intuitively fit the ${S}_{N}2$ mechanism (see cases in Supplementary Information Note 10). Therefore, many other reaction mechanisms can be introduced; for example, cycloaddition and elimination-addition, which can be flexibly referred to design the decision process. Additionally, we advise to add a mechanism selection module determined by the confidence of LGM and RCP to decide which type of mechanism is suitable to produce explanations easier for human understanding.

*The disability to produce finely-grained predictions*. Like most data-driven retrosynthesis models, our RetroExplainer is unable to predict more detailed reaction information, such as reaction operations, temperature, and duration, due to the research gap of corresponding DL models and the lack of public datasets, which has become a more and more urgent challenge to the development of an automated synthesis platform. This is an issue for our future research to explore.

## Methods

### Problem definition

For the convenience of description and discussion, we here briefly introduce the problem definition.

*Graph-based retrosynthesis prediction.* Generally, a chemical reaction can be denoted as $({\{{{{{{{\mathscr{G}}}}}}}_{{p;i}}\}}_{i=1}^{{Np}},{\{{{{{{{\mathscr{G}}}}}}}_{{r;j}}\}}_{j=1}^{{N}_{r}})$, where ${{{{{{\mathscr{G}}}}}}}_{p}$ represents a product graph and ${{{{{{\mathscr{G}}}}}}}_{r}$ is a reactant graph. Similarly, a reaction inference model can be ${f}_{\theta }({\{{{{{{{\mathscr{G}}}}}}}_{{p;i}}\}}_{i=1}^{{Np}},|,{\{{{{{{{\mathscr{G}}}}}}}_{{r;j}}\}}_{j=1}^{{N}_{r}})$, where $\theta$ is a group of learnable parameters. However, retrosynthesis prediction is not as simple as an inverse model ${f}_{\gamma }({\{{{{{{{\mathscr{G}}}}}}}_{{r;j}}\}}_{j=1}^{{N}_{r}},|,{\{{{{{{{\mathscr{G}}}}}}}_{{p;i}}\}}_{i=1}^{{Np}})$ for forwarding inference. In practical analysis, the input only includes the main product, which neglects any other by-products as a prior knowledge. Thus, the actual model for retrosynthesis is ${f}_{\gamma }({\{{{{{{{\mathscr{G}}}}}}}_{{r;j}}\}}_{j=1}^{{N}_{r}},{\{{{{{{{\mathscr{G}}}}}}}_{{p;i}}\}}_{i=1}^{{Np}}/\{{{{{{{\mathscr{G}}}}}}}_{{p;m}}\},|,{{{{{{\mathscr{G}}}}}}}_{{p;m}})$, where ${{{{{{\mathscr{G}}}}}}}_{{p;m}}$ denotes the main product. It is more complex than the forward prediction, which is why the performance of the forward model is generally better than that of retrosynthesis prediction.

*Reaction center (RC) and leaving group (LG).* In this work, the RC is defined as a subgraph ${{{{{{\mathscr{G}}}}}}}_{c}=\{\left(u,v\right),{e}_{{uv}}|{e}_{{uv}}\in {{{{{{\mathscr{G}}}}}}}_{p},{e}_{{uv}}\notin {{{{{{\mathscr{G}}}}}}}_{{r;j}},\forall {{{{{{\mathscr{G}}}}}}}_{{r;j}}\in {\{{{{{{{\mathscr{G}}}}}}}_{{r;i}}\}}_{i=1}^{{N}_{r}}\}$, where $\left(u,v\right)$ denotes an atom pair and ${e}_{{uv}}$ is their edge. Furthermore, synthons are a set of graphs modified by products according to RCs. These synthons are not usually chemically valid and can be later converted to final reactants by attaching LGs. Herein, LG ${{{{{{\mathscr{G}}}}}}}_{l}$ is defined as a subgraph from reactants, which contains reactants atoms and edges that do not occur in the main product. Namely, LG records information from by-products ${\{{{{{{{\mathscr{G}}}}}}}_{{p;i}}\}}_{i=1}^{{Np}}/\{{{{{{{\mathscr{G}}}}}}}_{{p;m}}\}$.

*Transformer from a graph neural network (GNN) perspective.* Before this section, the Preliminaries and Notes section in Supplementary Information Note 11 is recommended. In the data flow of the Transformer, a sentence can be seen as a fully connected graph with the semantic edge, where word tokens are processed as nodes. In this view, multi-head attention (MHA) can be factorized as follows:

1 $${m}_{a}^{\left(l\right)}{{{{{\rm{:=}}}}}}\mathop{\sum }\limits_{{w}^{{\prime} }\in {{{{{{\mathscr{N}}}}}}}_{w}}{\widetilde{a}}_{{w}^{{\prime} }w}{S}_{{w}^{{\prime} }}^{\left(l-1\right)}{W}_{V},\quad{m}_{w}^{\left(l\right)}:={\widetilde{a}}_{{ww}}{S}_{w}^{\left(l-1\right)}{W}_{V},$$

2 $$s.t.{\widetilde{a}}_{{w}^{{\prime} }w}={SOFTMAX}\left(\frac{{S}_{{w}^{{\prime} }}^{\left(l-1\right)}{W}_{Q}{\left({S}_{w}^{\left(l-1\right)}{W}_{K}\right)}^{T}}{\sqrt{{d}_{k}}}\right),$$

3 $${{S}^{{{{\hbox{'}}}{{\hbox{'}}}}}}_{w}^{\left(l\right)}={{COMBINE}}^{\left(l\right)}\left({m}_{a}^{\left(l\right)},{m}_{w}^{\left(l\right)}\right):={CONCAT}\left({\left\{{m}_{a}^{\left({l;k}\right)}+{m}_{w}^{\left({l;k}\right)}\right\}}_{k=1}^{{n}_{{head}}}\right){W}^{O},$$

where ${{{{{{\mathscr{N}}}}}}}_{w}=S/\left\{w\right\}$ is the semantic neighbor set of $w$, and ${d}_{k}$ is the hidden dimension of ${W}_{K}$. Then, Eq. (S11.10) acts like a non-linear propagation layer in a typical GNN, which transforms ${{S}^{{{{\hbox{'}}}{{\hbox{'}}}}}}_{w}^{\left(l\right)}$ to final ${S}_{w}^{\left(l\right)}$. Note that the normalized attention matrix ${\widetilde{A}}_{s}$ composed of ${\widetilde{a}}_{{w}^{{\prime} }w}$ of each word token pair $\left({w}^{{\prime} },w\right)$ can be considered as a group of adaptive parameters that describe the dynamic distribution of semantic edges. However, the standard Transformer cannot handle edges on a topological space. As a variant of the Transformer, our RetroExplainer solves this edge embedding problem.

### The framework of the proposed RetroExplainer

The overview of our RetroExplainer is illustrated in Fig. 1a. As shown, our RetroExplainer contains four major modules: (1) MSMS-GT module, (2) DAMT learning module, (3) explainable decision-making module, and (4) prediction and pathway planning module. The workflow of our RetroExplainer is described below.

The MSMS-GT module, illustrated in Fig. 1b (right), utilizes a multi-sense and multi-scale bond embedding strategy for the chemical bonds and topological embedding of the atoms to capture chemically important information. The molecular vectors resulted from former two encoders are blended via the MHA mechanism. In the DAMT learning module, the resulting hidden molecular representations are simultaneously fed into three specific task heads: RCP, LGM, and LGC, shown in Fig. 1b (left), which are trained using a DAMT learning strategy (as shown in Fig. 1c) to train each sub-task equally. RCP identifies changes in bonds and atoms’ hydrogen count, LGM matches the LGs (as described in the *Reaction center (RC) and leaving group (LG)* section) from the collected database for products, and LGC connects the LGs and fragments from the product. The decision-making module transforms the product into reactants using a decision process consisting of five retrosynthetic actions (as shown in Fig. 1d) and energy scores for decision curves, thus simulating a reversed molecular assembly process. Finally, based on single-step predictions, a heuristic tree-search algorithm is integrated into the last module to discover efficient synthetic routes with transparent decision-making processes while ensuring the accessibility of the starting reactants. More details about the MSMS-GT module, DAMT learning module, and decision-making module are described below.

### Multi-sense and multi-scale Graph Transformer (MSMS-GT) module

*Atomic and topological embedding.* Given reaction data ${S}_{r}$ represented by SMILES, the related molecule graph of the product ${{{{{{\mathscr{G}}}}}}}_{p}=\left({{{{{\mathscr{V}}}}}}{{{{{\mathscr{,}}}}}}{{{{{\mathscr{A}}}}}}\right)$ is constructed, where atoms are viewed as a set of nodes ${{{{{\mathscr{V}}}}}}$ with size $N$. To fit such graph data into the Transformer variant, a naive approach considers atoms as word tokens, which are then a reference to build a fully connected graph of the semantic domain. In RetroExplainer, we adopt this simple method and additionally employ a topological embedding that measures a node’s importance in the space domain’s graph instead of using conventional position encoding, which destroys original permutation invariance. In detail, we use degree counts of the node to describe the above importance. Therefore, the initial node feature ${h}_{v}^{\left(0\right)}$ of atom $v$ can be calculated as follows:

4 $${h}_{v}^{\left(0\right)}=\mathop{\sum }\limits_{i=1}^{{K}_{a}}{\phi }_{{x}_{i}}\left({x}_{i}\right)+{\phi }_{d}\left({{{{{\rm{deg}}}}}} (v)\right),$$

where $\phi \left(\cdot \right)$ with different subscripts denote different embedding functions, ${X}_{i}$ is the $i$-th atomic feature (e.g., atomic number, formal charge), and $\deg \left(v\right){\mathbb{\in }}{\mathbb{R}}$ is the total degree count of atom $v$. By introducing such structural signals as strong prior knowledge, the attention score in Eq. (S11.7) can capture both semantic and space domain information.

*Multi-sense and multi-scale bond embedding*. Equipped with topological embedding, RetroExplainer is improved because beneficial structure messages are passed for better graph understanding. Nonetheless, it still neglects abundant bond information, which makes the model unable to distinguish some isomers that share the exact degree count of each atom. The details can be found in Supplementary Information Note 12.

Therefore, it is necessary to introduce bond information to improve the graph expressivity of the model. In the implementation, we encode bond properties (e.g., $\sigma$ orbit, $\pi$ orbit, conjugated bond), which are then represented as adjacency matrices${\{{A}_{i}\in {\{{{{{\mathrm{0,1}}}}}\}}^{N\times N}\}}_{i=1}^{{K}_{b}}$ of different ${K}_{b}$ senses, rather than using the type-based approach to embed edge features. The reason is that by directly exposing specific reaction-related attributes (e.g., whether in the ring) hidden by bond type, RetroExplainer can better understand complex reaction data. Furthermore, some bond types share the same properties (e.g., C-C and C=C have $\sigma$ orbit, but conventional encoding for bond type cannot express such signal, whereas multi-sense embedding is free of this message gap).

With bond encoding improved, the next problem is how to integrate it into graph data for a Transformer. It is not easy because an atom-pair-based bond cannot be embedded to the degree that a single atom defines. Inspired by Eq. (2), where atom-pair-based semantic edges are learned, we consider bond information as natural prior knowledge of the molecular structure and add it to the semantics score before ${SOFTMAX}\left(\cdot \right)$. Then we have:

5 $${m}_{a}^{\left(l\right)}=\mathop{\sum }\limits_{u\in {{{{{{\mathscr{N}}}}}}}_{v}}{\widetilde{a}}_{{uv}}{h}_{u}^{\left(l-1\right)}{W}_{V},\, {m}_{v}^{\left(l\right)}\,=\, {\widetilde{a}}_{{vv}}{h}_{v}^{\left(l-1\right)}{W}_{V},$$

6 $${a}_{{uv}}=\frac{{h}_{u}^{\left(l-1\right)}{W}_{Q}{\left({h}_{v}^{\left(l-1\right)}{W}_{K}\right)}^{T}}{\sqrt{{d}_{k}}}+\mathop{\sum }\limits_{i}^{{K}_{b}}{\phi }_{i}\left({A}_{{i;uv}}\right),\quad{\widetilde{a}}_{{uv}}={{{{{\rm{SOFTMAX}}}}}}\left({a}_{{uv}}\right),$$

where ${{{{{{\mathscr{N}}}}}}}_{v}{{{{{\mathscr{=}}}}}}{{{{{\mathscr{V}}}}}}{{{{{\mathscr{/}}}}}}\left\{v\right\}$ is a set of neighbors of atom $v$. However, it still cannot be as expressive as a simple GNN in practice, which excessively focuses on 1-hop neighbors in the space domain. To alleviate this problem, Graphormer proposes a shortest-path embedding to acquire a global scope, and it achieves SOTA (state-of-the-art) performances in graph domain tasks. Meanwhile, a global distance ${A}_{g}$ can be easily calculated by bond length or three-dimensional (3D) conformation optimized by MMFF^53^. Introducing global embedding, we have:

7 $${a}_{{uv}}=\frac{{h}_{u}^{\left(l-1\right)}{W}_{Q}{\left({h}_{v}^{\left(l-1\right)}{W}_{K}\right)}^{T}}{\sqrt{{d}_{k}}}+\mathop{\sum }\limits_{i}^{{K}_{b}}{\phi }_{i}\left({A}_{i{{{{{\rm{;}}}}}}{uv}}\right)+{RBF}\left({A}_{g{{{{{\rm{;}}}}}}{uv}}\right),$$

where RBF is a Gaussian radial basis function calculated as follows:

8 $${{{{{\rm{RBF}}}}}}\left(A\right)=\frac{\exp \left({-\left(A-{\phi }_{{mean}}\right)}^{2}/2{\phi }_{{std}}^{2}\right)}{\sqrt{2\pi }{\phi }_{{std}}}.$$

It should be noted that global embedding introduces spatial information but ignores bond features in the overall scale. Furthermore, atom environment (AE), which regards several atoms as a token, plays a significant role in retrosynthetic prediction. Therefore, inspired by RetroTRAE^28^, we propose a multi-level AE embedding that captures various radii of AEs by simply calculating different powers of the 0-1 adjacency matrix ${A}_{i}$. Thus, the final attention score is calculated as follows:

9.1 $$bia{s}_{uv}=\underbrace{{\sum }_{i}^{{K}_{b}}{\sum }_{j=1}^{{K}_{r}}{\phi }_{i}({A}\,_{i;uv}^{j})}_{Local\,Item}+\underbrace{RBF({A}_{g;uv})}_{Global\,Item},$$

9.2 $${a}_{{uv}}=\frac{{h}_{u}^{\left(l-1\right)}{W}_{Q}{\left({h}_{v}^{\left(l-1\right)}{W}_{K}\right)}^{T}}{\sqrt{{d}_{k}}}+{bia}{s}_{{uv}},$$

where ${A}\,_{i}^{j}$ is the *j-*th power of ${A}_{i}$, which describes AE with *j* radius; and ${K}_{r}$ is the maximum radius of AEs we take into account. Note that we later use the local item to denote ${\phi }_{i}({A}\,_{{i;uv}}^{j})$ and global item to denote ${RBF}({A}_{{g;uv}})$. What calls for special attention is that ${A}\,_{i}^{j}$ with ${A}_{i}\in {\left\{{{{{\mathrm{0,1}}}}}\right\}}^{N\times N}$ records the number of paths for length $j$ between each atom, which describes $j$ radius environment for each central atom. Finally, in Eq. (9), we firstly employ a self-attention mechanism to learn a semantic relation for the combined feature of node, secondly, introduce a multi-level environment${\{{A}\,_{i}^{j}\}}_{j=1}^{{K}_{r}}$ and multi-sense embedding ${\{{A}_{i}\}}_{i=1}^{{K}_{b}}$ to take advantage of abundant bond information on a local scale, and thirdly, add a global embedding for atoms to capture a global scope in the spatial domain. Therefore, by calculating ${a}_{{uv}}$ in Eq. (9), the expressive power of RetroExplainer is at least as powerful as GNNs. Additionally, the global item ${RBF}({A}_{{g;uv}})$ allows the chance to introduce the 3D distance embeddings, although that has been proven to be unnecessary, according to the results in Supplementary Information Note 13.

### Specific task heads and decision-making module

In RetroExplainer, three specific task heads are designed for different demands whose outputs are all useful to retrosynthetic analysis for the planning of reaction pathways. In detail, the RCP gives a probability distribution for each bond change which decides RCs; LGM evaluates a compatibility score between the product and each candidate LG; and LGC determines the location where the RC and LG connect. Notice that all specific task heads are closely related, so they are modeled in a multi-task distribution manner. Thus, the distribution of the three units can be modeled as follows:

10 $${p}_{\theta }\left({\left\{{G}_{r;i}\right\}}_{i=1}^{{N}_{r}}\bigg|{G}_{p;m}\right)=\underbrace{{p}_{{\theta }_{1}}\left({\left\{{G}_{r;j}\right\}}_{j=1}^{{N}_{r}}\bigg|{G}_{p;m},{\left\{{G}_{l;\,j}\right\}}_{j=1}^{{K}_{l}}\right)}_{LGC}\underbrace{{p}_{{\theta }_{2}}\left({\left\{{G}_{c;i}\right\}}_{i=1}^{{K}_{c}},{\left\{{G}_{l;i}\right\}}_{i=1}^{{K}_{l}}\bigg|{G}_{p;m}\right)}_{RCP\&LGM},$$

where ${K}_{c}$ and ${K}_{l}$ denote the number of RCs and LGs, respectively. Notice that ${p}_{{\theta }_{1}}\left(\cdot \right)$ in Eq. (10) also obscurely models the conditional distribution between the given main product and unknown by-products because once the by-products are predicted correctly, the related reactants can be easily influenced by dual models of higher performance synthesis prediction.

*Reaction center prediction (RCP).* Instead of directly predicting the bond type of RCs, we factorize it into two sub-tasks for bond (RCP-B) or hydrogen (RCP-H) change. Between the two sub-tasks, we model the bond change prediction as a sparse edge link identification task and the hydrogen change prediction as node-level classification with $2k+1$ labels, where $k$ is the max number of changed hydrogens. To predict RCs for a given product ${{{{{{\mathscr{G}}}}}}}_{p}$ with node representation ${h}_{v}^{\left(L\right)}$ after $L$ layers, we calculate the probabilities of bond change ${p}_{{uv}}$ for atom pair $\left(u,v\right)$ and hydrogen change ${p}_{v}$ for atom $v$, which can be viewed as follows:

11 $${p}_{{uv}}=\sigma \left({CONCAT}\left({\left\{\frac{{h}_{u}^{\left(L\right)}{W}_{Q}{\left({h}_{v}^{\left(L\right)}{W}_{K}\right)}^{T}}{\sqrt{{d}_{k}}}\right\}}_{i=1}^{{n}_{{head}}}\right){W}_{{bond}}\right),$$

12 $${p}_{v}={SOFTMAX}\left({h}_{v}^{\left(L\right)}{W}_{{atom}}\right),$$

where ${W}_{{bond}}$ and ${W}_{{atom}}$ denote linear layers that aggregate messages from different heads. In addition, RCP is optimized as follows:

13 $${{{{{{\mathscr{L}}}}}}}_{B}=-\mathop{\sum }\limits_{({{{{{{\mathscr{G}}}}}}}_{p},{RC})}\mathop{\sum }\limits_{\left(u,v\right)\in {RC}}{y}_{{uv}}\log \left({p}_{{uv}}\right),\quad{{{{{{\mathscr{L}}}}}}}_{H}=-\mathop{\sum }\limits_{({{{{{{\mathscr{G}}}}}}}_{p},{RC})}\mathop{\sum }\limits_{v\in {RC}}\log \left({p}_{v{{{{{\rm{;}}}}}}{y}_{v}}\right).$$

*Leaving group matcher (LGM).* We model LGM as a graph-level multi-classification task instead of an autoregressive one. The reason is that we find a small ratio (about 0.46%) between the LG and the number of reactions, meaning several definite patterns appear in LGs. As a result, after applying a heuristic breadth-first traversal algorithm to unify permutations for nodes in the same kind of LGs, we can obtain 231 types of LG in USPTO-50K. Based on these collected LGs, we construct a vocabulary ${{{{{{\mathscr{V}}}}}}}_{{LG}}$ whose index maps a determinate LG. Therefore, the predicted distribution ${p}_{{{{{{{\mathscr{G}}}}}}}_{p}}$ and optimizing target for LGM are as follows:

14 $${p}_{{{{{{{\mathscr{G}}}}}}}_{p}}={h}_{{{{{{{\mathscr{G}}}}}}}_{p}}{W}_{{LG}},\,{h}_{{{{{{{\mathscr{G}}}}}}}_{p}}={h}_{s}^{\left(L\right)},\,{{{{{{\mathscr{L}}}}}}}_{{LG}}=-\mathop{\sum }\limits_{{{{{{{\mathscr{G}}}}}}}_{p}}\log \frac{\exp \left({p}_{{{{{{{\mathscr{G}}}}}}}_{p{{{{{\rm{;}}}}}}{y}_{+}}}\right)}{{\sum }_{y\in {{{{{{\mathscr{V}}}}}}}_{{LG}}}\exp \left({p}_{{{{{{{\mathscr{G}}}}}}}_{p{{{{{\rm{;}}}}}}y}}\right)},$$

where ${h}_{{{{{{{\mathscr{G}}}}}}}_{p}}$ is a graph-level representation for ${{{{{{\mathscr{G}}}}}}}_{p}$, and ${h}_{s}$ denotes the feature of the super node. The super node is like $< {cls} >$ token in natural language processing and is a virtual node in a practical sense, which is set to be connected by a virtual edge to all the other nodes.

*Leaving group connector* *(LGC)*. We also design LGC as a sparse edge link process like RCP-B in Eq. (11). The difference is that we only predict the connections between connected atoms in LGs and the product, which reduces computation complexity from $O({NM})$ to $O(N)$, where $N,\,M$ denotes the number of atoms for product and LG, respectively. The motivation is the limited number of connected atoms in LGs.

*Chemical knowledge and deep-learning (DL)-guided molecular assembly decision process*. RetroExplainer offers a more transparent and interpretable approach to retrosynthesis analysis compared to previous end-to-end prediction methods. As illustrated in Fig. 3a, the process can be divided into six stages: P, S-LGM, IT, S-RCP, S-LGC, and HC. The process is evaluated using a flexible user-designed energy function that calculates the predicted probability distribution. In the S-LGM step, the energy score is based on the compatibility between the LG and the product, as determined by LGM. In the IT step, the selected LG is not attached, and all potential RCs remain intact. In S-LGC, the connected bonds are identified and scored using LGC. It is important to note that information on connected bond types is recorded in the matched LG. In bond changing, RCP identifies and scores the bonds from RCs. Finally, in the HC step, we adjust the amount of hydrogen for noncompliant atoms and filter out all illegal molecules to generate the final reactants. In this study, we define the energy function as the negative logarithm of the probabilities of associated conditions.

### Augmentation modules

*Structure-aware Contrastive Learning (SACL)*. In LGM, given a product ${{{{{{\mathscr{G}}}}}}}_{p}$, we directly predict the index of the most appropriate LG for ${{{{{{\mathscr{G}}}}}}}_{p}$, which, however, still ignores the rich structural information of LG and limits the performance of overall prediction. Hence, benefiting from contrastive learning techniques in molecular representations (e.g., MolCLR^54^, 3D Infomax^55^), we also adopt a contrastive learning strategy that minimizes the divergence of representations between LG ${h}_{{{{{{{\mathscr{G}}}}}}}_{{LG}}}^{+}$ and matched product ${h}_{{{{{{{\mathscr{G}}}}}}}_{p}}^{+}$ and maximize it in any other conditions. Based on this supervised contrastive-learning-based idea, an optimizing object inspired by SupContrast can be viewed as follows:

15 $${{{{{{\mathscr{L}}}}}}}_{c}=-\mathop{\sum }\limits_{{{{{{{\mathscr{G}}}}}}}_{{{{{\mathrm{lg}}}}}}}\frac{1}{\left|{{{{{{\mathscr{G}}}}}}}_{p}^{+}\right|}\mathop{\sum }\limits_{{{{{{{\mathscr{G}}}}}}}_{p}^{+}}\log \frac{\exp \left({sim}\left({h}_{{{{{{{\mathscr{G}}}}}}}_{{{{{\mathrm{lg}}}}}}},\,{h}_{{{{{{{\mathscr{G}}}}}}}_{p}}^{+}\right)/\tau \right)}{{\sum }_{{{{{{{\mathscr{G}}}}}}}_{p}}\exp \left({sim}\left({h}_{{{{{{{\mathscr{G}}}}}}}_{{{{{\mathrm{lg}}}}}}},\,{h}_{{{{{{{\mathscr{G}}}}}}}_{p}}\right)/\tau \right)},$$

where ${sim}\left({h}_{u},{h}_{v}\right)$ measures the similarity between ${h}_{u}$ and ${h}_{v}$, and $\tau$ is a hyper-parameter and a scalar. In practice, recognizing a positive instance for every batch brings in extra costs. For this reason, after regular forward of LGM, we decorate every ${h}_{{{{{{{\mathscr{G}}}}}}}_{{LG}}}$ with the same contrastive token and then input it to the LGM to predict the corresponding index in vocabulary ${{{{{{\mathscr{V}}}}}}}_{{LG}}$. Thus, the enhanced loss function ${{{{{{\mathscr{L}}}}}}}_{{LG}}$ for LGM is as follows:

16 $${{{{{{\mathscr{L}}}}}}}_{{{{{\mathrm{lg}}}}}}=-\frac{1}{2}\left (\mathop{\sum }\limits_{{{{{{{\mathscr{G}}}}}}}_{p}}\log \frac{\exp \left({p}_{{{{{{{\mathscr{G}}}}}}}_{p{{{{{\rm{;}}}}}}{y}_{+}}}/\tau \right)}{{\sum }_{y\in {{{{{{\mathscr{V}}}}}}}_{{{{{\mathrm{lg}}}}}}}\exp \left({p}_{{{{{{{\mathscr{G}}}}}}}_{p{{{{{\rm{;}}}}}}y}}/\tau \right)}\right.\left.+\mathop{\sum }\limits_{{{{{{{\mathscr{G}}}}}}}_{{{{{\mathrm{lg}}}}}}}\log \frac{\exp \left({p}_{{{{{{{\mathscr{G}}}}}}}_{{{{{\mathrm{lg}}}}}{{{{{\rm{;}}}}}}{y}_{+}}}/\tau \right)}{{\sum }_{y\in {{{{{{\mathscr{V}}}}}}}_{{{{{\mathrm{lg}}}}}}}\exp \left({p}_{{{{{{{\mathscr{G}}}}}}}_{{{{{\mathrm{lg}}}}}{{{{{\rm{;}}}}}}y}}/\tau \right)}\right),$$

where ${p}_{{{{{{{\mathscr{G}}}}}}}_{{LG}}}=\left({h}_{{{{{{{\mathscr{G}}}}}}}_{{LG}}}+{h}_{{con}}\right){W}_{{LG}}$ denotes the distribution that LGM predicts, and ${h}_{{con}}$ is the feature of the contrastive token. This strategy plays the same role as Eq. (14) without the above extra costs. Furthermore, what needs to be emphasized is that we only apply this technique in the training stage. As a result, we can make RetroExplainer perceive the structural information of LGs without extra prior knowledge.

*Dynamic adaptive multi-task learning (DAMT)*. To predict probabilities of bond changes ${p}_{{uv}}$, hydrogen attachments ${p}_{v}$, and LGM ${p}_{{LG}}$ jointly, product ${{{{{{\mathscr{G}}}}}}}_{p}$ is first sent into shared layers to learn the mutual representation of three tasks, which is then passed to each specific task layer. Because of the immediate relevance between the RC and LG, it can be a mutual promotion learning process for multi-task training. However, optimizing four targets at the same time can be conflicting for parameters in a shared layer due to the discordance of complexity for tasks and magnitude for loss functions. In RetroExplainer, we propose a multi-task learning strategy that can adaptively adjust weights for the above three losses. In detail, we introduce a descent rate for $i$-th loss ${r}_{i}^{\left(t\right)}$ in $t$-th training step to measure the complexity of $i$-th task and a normalizing coefficient ${\alpha }_{i}$ to unify the magnitude of $i$-th loss. Combined above, the total loss of $t$-th step ${{{{{{\mathscr{L}}}}}}}_{T}^{\left(t\right)}$ is as follows:

17 $${{{{{{\mathscr{L}}}}}}}_{T}^{\left(t\right)}=\mathop{\sum }\limits_{i=1}^{{K}_{t}}\left(\frac{\exp \left(\frac{{r}_{i}^{\left(t\right)}}{\tau }\right)}{{\sum }_{j=1}^{{K}_{t}}\exp \left(\frac{{r}_{j}^{\left(t\right)}}{\tau }\right)}{\alpha }_{i}^{t}{{{{{{\mathscr{L}}}}}}}_{i}^{\left(t\right)}\right),\,{r}_{i}^{\left(t\right)}=\frac{{{{{{{\mathscr{L}}}}}}}_{i}^{\left(t-1\right)}}{{{{{{{\mathscr{L}}}}}}}_{i}^{\left(t-2\right)}},\,{\alpha }_{i}^{t}=\frac{n}{{\sum }_{j=t-1}^{t-n}{{{{{{\mathscr{L}}}}}}}_{i}^{(\,j)}},$$

where $n$ is the capacity of the queue that we take into consideration to obtain $\alpha$. The pseudocode of DAMT can be found in Supplementary Information Note 14.

### Model implementation details

We trained RetroExplainer using the AdamW^56^ optimizer for gradient descent with a weight decay rate of $0.01$. Additionally, we adopted a polynomial decay learning rate scheduler with an extra warm-up stage. The learning rate was controlled by the scheduler to increase linearly and rapidly to a preset peak value ($2\times {10}^{-5}$) from the initial learning rate ($1\times {10}^{-7}$) and then decrease slowly with the process of iterations. The whole training phase was monitored by the early stop strategy with the patient epoch and the maximum epoch set to $50$ and $2000$, respectively. The shared MSMS-GT encoder layer was set to 16, and each sublayer was assigned as a single layer, where the dimensions of the hidden layer and feedforward network were 512 and 1024 for the three datasets (i.e., USPTO-50K, USPTO-FULL, and USPTO-MIT), respectively. Additionally, for DAMT, we set the queue length to $50$ to obtain the normalizing coefficients. The complete training phase for the USPTO-50K dataset takes around 40 hours when the reaction type is provided and 38 hours when it is not provided, utilizing a single RTX3090 GPU core; by contrast, it takes roughly 7.5 and 14.5 days for the larger datasets, USPTO-MIT and USPTO-FULL, respectively, when run on three RTX3090 GPU cores in parallel.

### Supplementary information

Supplementary Information

Peer Review File

Description of Additional Supplementary Files

Supplementary Data 1

Supplementary Data 2

### Source data

Source Data

## Data Availability

We used the benchmark datasets USPTO-50K, USPTO-MIT, and USPTO-FULL for all our experiments. For a fair comparison, we used the same version and splits as those provided by Yan et al. ^32^. for USPTO-50K and USPTO-FULL. The USPTO-MIT dataset is provided by Chen et al.^57^, as Yan et al.^32^. have not run their model on this dataset. Additionally, because the USPTO-50K comes with the risk of a data leakage caused by erroneously using an atomic mapping algorithm, we shuffle these mapping numbers to ensure the position of RCs is not concentrated in the first position of the atomic arrangement. Source data are provided with this paper through 10.6084/m9.figshare.23590230. Source data are provided with this paper.

## Code availability

All code used in data analysis and preparation of the manuscript, alongside a description of necessary steps for reproducing results, can be found in a GitHub repository accompanying this manuscript: https://github.com/wangyu-sd/RetroExplainer. Additionally, the source code is also available at Zenodo repertory^58^ through https://zenodo.org/record/8251390.
