# Supplementary material for: Retrosynthesis prediction with an interpretable deep-learning framework based on molecular assembly tasks
Source: Nat Commun. 2023 Oct 3;14:6155. doi: 10.1038/s41467-023-41698-5 (PMC10547708; doi:10.1038/s41467-023-41698-5)
Supplement: Supplementary file 1 — Supplementary Information [file 41467_2023_41698_MOESM1_ESM.pdf]

# Supplementary Information

**Retrosynthesis prediction with an interpretable deep-learning framework based on molecular assembly tasks**

Yu Wang[1,2], Chao Pang[1,2], Yuzhe Wang[1,2], Junru Jin[1,2], Jingjie Zhang[1,2], Xiangxiang Zeng[4], Ran Su[5], Quan Zou [3*], and Leyi Wei[,2*]

[1] School of Software, Shandong University, Jinan 250101, China

[2]Joint SDU-NTU Centre for Artificial Intelligence Research (C-FAIR), Shandong University, Jinan 250101, China

[3]Institute of Fundamental and Frontier Sciences, University of Electronic Science and Technology of China

[4]College of Computer Science and Electronic Engineering, Hunan University, Changsha, China

[5]College of Intelligence and Computing, Tianjin University, Tianjin, China

*Corresponding author:

Quan Zou: zouquan@nclab.net

Leyi Wei: weileyi@sdu.edu.cn

# Contents


Supplementary Note 1. Ablation studies on scale information and strategies of MSMS-GT ..... 4

Supplementary Note 2. Illustration for multi-scale expressivity on edge representation ..... 8

Supplementary Note 3. Comparations against previous popular methods from the molding view ..... 11

Supplementary Note 4. The calculation details of proposed decision process ..... 13

Supplementary Note 5. Top-12 predictions and decision path cures ..... 17

Supplementary Note 6. Details of reranking existing approaches for performance improvement ..... 23

Supplementary Note 7. The multi-planning algorithm we used and the 101 pathways we found ..... 25

Supplementary Note 8. Scatters of t-SNE using nine different random seeds ..... 26

Supplementary Note 9. Prediction cases on rare leaving groups ..... 35

Supplementary Note 10. Cases on non-leaving-group reactions ..... 39

Supplementary Note 11. Preliminaries and Notes ..... 41

Supplementary Note 12. A case for the isomer cannot be distinguished by the models without bond encoder ..... 43

Supplementary Note 13. Comparations on 3D-RetroExplainer and 2D-RetroExplainer ..... 44

Supplementary Note 14. About dynamic adaptive multi-task learning strategy (DAMT) ..... 46

Supplementary Note 15. Atom-perturbation-based explainability (APEx) and reaction type tracing analysis ..... 49

Supplementary Note 16. Failure cases of RetroExplainer trained on USPTO-50K dataset ..... 53

**Supplementary Note 17. Reaction type mutation experiment and directed retrosynthesis prediction** ............................... 57

**Supplementary references** ............................................................................................................................................................. 58

## Supplementary Note 1. Ablation studies on scale information and strategies of MSMS-GT

To examine the influence of attention bias as described in Eq. (9.1) of the main text on the outcomes, we conducted an ablation study guided by scale information (refer to Supplementary Figures 1a-c). As depicted in Supplementary Figure 1.1 a-d, the top-1 accuracy follows a pattern of gradual increase followed by decrease as the max hop count rises (ranging from 0 to 5), reaching its zenith at a max hop count of 4. This behavior could stem from a competitive interaction between global and local elements. When the max hop count is reduced to a relatively small positive integer, it signifies that the local element in Eq. (9.1) of the main text misses a greater number of high-order components compared to prior settings. This, in turn, leads to attention biases among different heads, causing them to disregard more local scale structures, thereby resulting in a decrease in top-1 accuracy.

Conversely, when the max hop count is set to four, a balance is struck between the local and global terms. Their cumulative effect, encapsulated within the attention bias, encompasses the multi-scale structural characteristics. Moreover, it is evident that the global term holds more significance than the local term. The drop in accuracy is more pronounced when the global term is masked, in contrast to setting the local term to zero. Interestingly, when the global term is masked, the local term exerts a more substantial impact on overall performance, indicating an overlap in the embedded structural insights provided by these two types of terms.

To evaluate the distinct impacts of the proposed strategies in RetroExplainer (contrastive learning strategy, self-adaptive coefficient, and multi-task learning), we conducted an ablation study encompassing overall prediction and four subtasks. The study involved four versions: 1) the

standard version with a max hop set to 4 (Norm), 2) the version without the contrastive learning strategy (w/o CL), 3) the version without the self-adaptive coefficient in multi-task learning (w/o SA), and 4) the version where all shared layers were removed, and each subtask was trained individually (w/o JL). The findings are presented in Supplementary Figure 1.2.

Comparing the Normal and w/o CL versions highlights the value of the contrastive training process in RetroExplainer. This process aids the model in grasping the structural information associated with the leaving group, leading to a notable enhancement of approximately 5% in the top-1 accuracy for the LGM task. Moreover, the RCP and LGC tasks also exhibit improvements. This can be attributed to the dissemination of shared knowledge from the LGM layer to other layers.

In terms of multi-task learning, we excluded all self-adaptive coefficients outlined in Eq. (16) from the main text and aggregated all sub-losses. The results depicted in Supplementary Figure 1.2 show that the w/o SA version underperforms in comparison to the other versions across most tasks. This disparity primarily arises from improper coefficients that fail to effectively balance the sub-tasks of varying complexities. It's worth noting that, while an alternative to the standard version could involve introducing a distinct set of hyperparameters for each task as fixed coefficients for corresponding loss functions, this approach raises two significant challenges: 1) finding the optimal combination of coefficients becomes intricate; and 2) even if optimal coefficients are determined, they may not dynamically suit every training epoch as fixed hyperparameters. In contrast, the adaptive coefficients in RetroExplainer are imperative for the multi-task learning framework to circumvent these issues.

Furthermore, directly removing the entire multi-task learning framework in RetroExplainer and training sub-tasks individually resulted in the w/o JL version. This version outperforms others in certain simpler tasks (e.g., LGC task) due to the reduced constraints imposed by the LGC layer on

learnable parameters. However, due to the absence of multi-task learning, the shared knowledge acquired from easier tasks cannot be effectively transferred to more challenging tasks (e.g., LGM task), ultimately leading to a performance decline in these demanding tasks. Consequently, we can deduce that both contrastive training and dynamic adaptive multi-task learning significantly enhance RetroExplainer's performance.

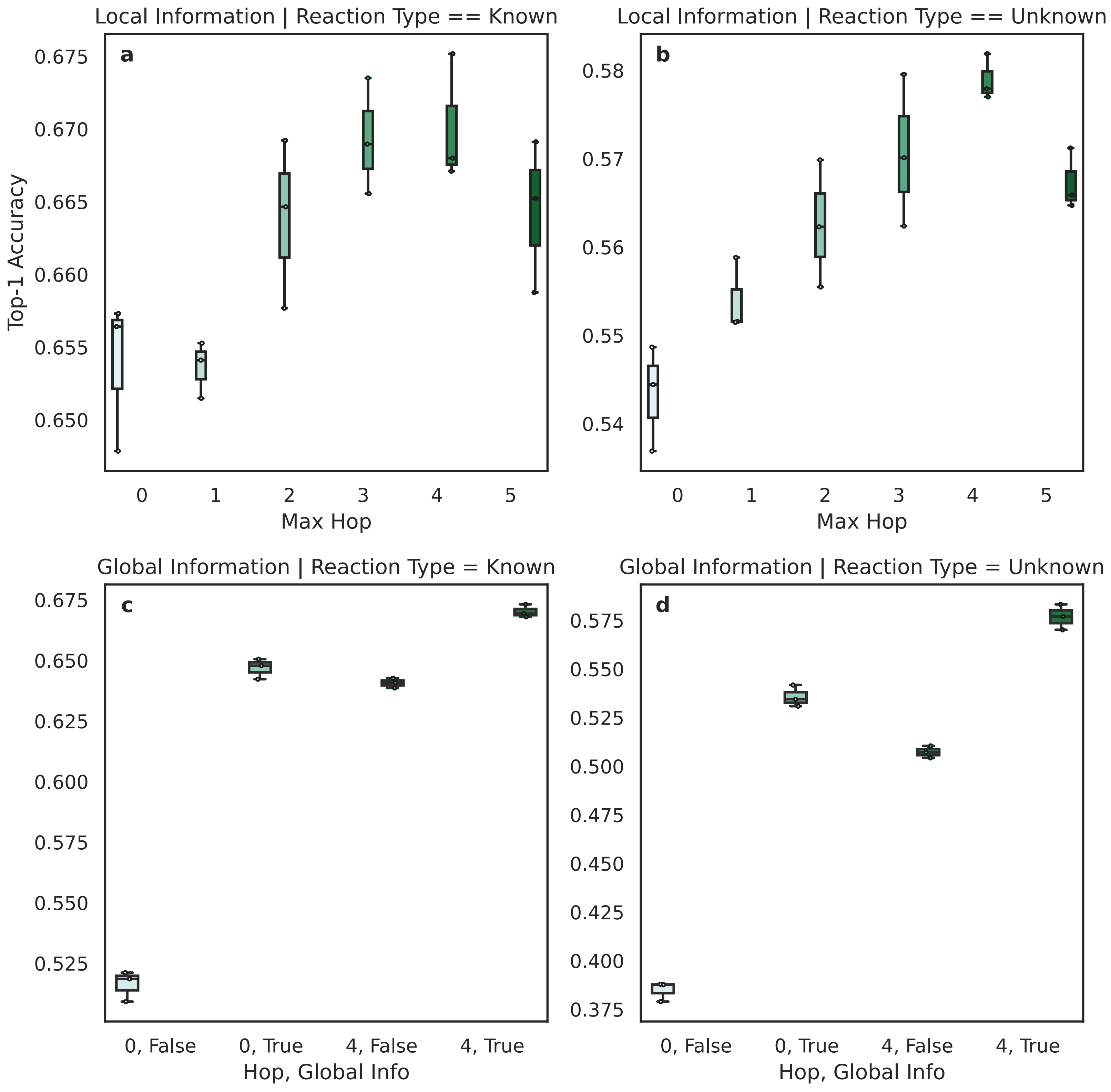


**Supplementary Figure 1.1. Ablation studies of scale information on known and unknown conditions for reaction types, respectively.** In a and b, we mainly focus on max hop of local item ranging from 0 to 5, and remove the global item to verify the validity. When max hop equals 4, the top1 accuracy reaches the maximum on both known and unknown reaction types. In c and d, we verified the impact on local item, global item as well as their combinations. In terms of the local item, we set parameter of max hop to 4; while the hop of 0 denotes the local item is removed.

Each result was derived from three repeated experiments conducted with distinct random seeds. The minimum, maximum, and median of the three data points are represented by the lower whisker, upper whisker, and central line within each box, respectively.

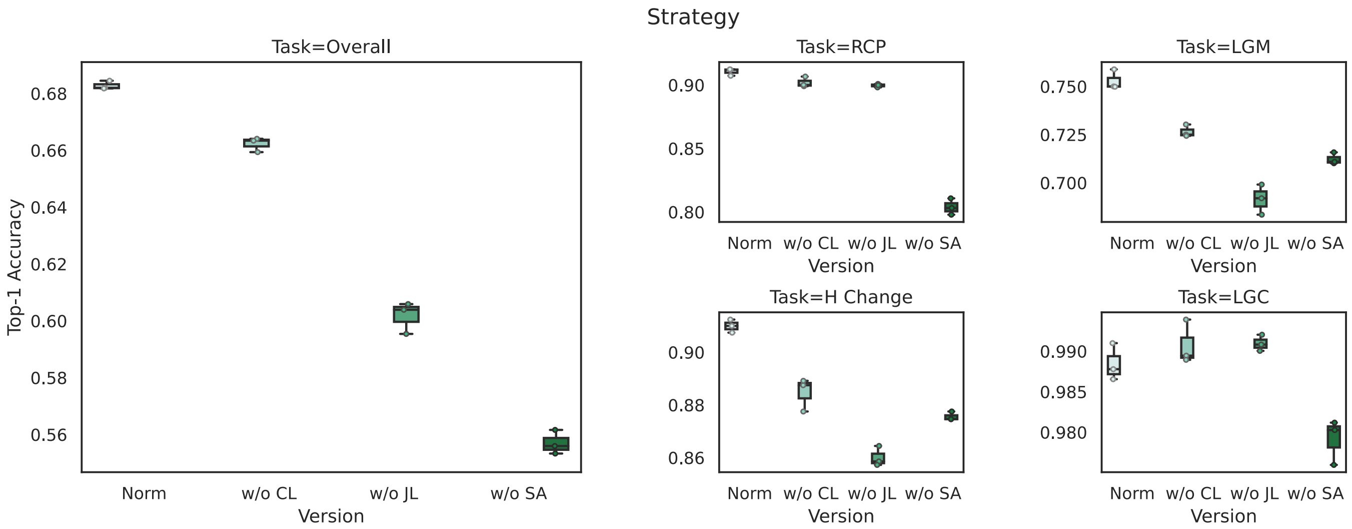


**Supplementary Figure 1.2. Ablation studies on different strategies for overall prediction and four subtasks.** We consider four conditions to compare: 1) normal version with max hop set to 4 (Norm), 2) without contrastive learning strategy (w/o CL), 3) without self-adaptive coefficient in multi-task learning (w/o SA), and 4) removing all shared layers and training each subtask individually (w/o JL).. Each result was derived from three repeated experiments conducted with distinct random seeds. The minimum, maximum, and median of the three data points are represented by the lower whisker, upper whisker, and central line within each box, respectively.

## Supplementary Note 2. Illustration for multi-scale expressivity on edge representation

In order to comprehend the impact of RetroExplainer's expressiveness on edge features, we extract the attention bias from Eq. (9.1) of the main text, forming adjacent matrices that represent edge features for the sampled molecular graph from different self-attention heads. This process results in the presentation of three distinct heat maps, as depicted in Supplementary Figure 2 and Supplementary Figure 3.

Comparing the adjacent matrix of the rudimentary bond type (see Supplementary Figure 2a) to the proposed bond embedding (see Supplementary Figure 2b), it becomes evident that the latter encapsulates a more intricate spectrum of topological edge information. In this representation, each self-attention head assigns varying levels of attention bias to atoms within the molecular graph. Notably, within these adjacent matrices originating from different heads, certain patterns manifest as diagonal trees. These patterns signify that globally focused heads are predominantly influenced by global elements. Conversely, other patterns form squares with diverse side lengths, indicating that locally focused heads are primarily influenced by local elements.

To emphasize this observation, we calculate the RV coefficient[1] between attention biases and global items, which serves as a metric to gauge the similarity between the two types of matrices. Subsequently, we arrange the heatmaps of attention bias in descending order based on their RV coefficient values. Supplementary Figure 3 illustrates that the heatmaps of attention bias can be categorized into three classes:

1. Heatmaps dominated by global items, boasting an RV coefficient exceeding 0.90 and patterns inclined towards diagonal trees.

2. Heatmaps controlled by local items, displaying a zero RV coefficient and square-like patterns.

3. Heatmaps characterized by a blend of global and local items, exhibiting an RV coefficient ranging from 0.61 to 0.50, with patterns encompassing both diagonal trees and squares.

In summation, these heatmaps underscore that the multi-head bond embedding block not only adeptly captures diverse levels of molecular structural information but also offers enhanced interpretability in contrast to the conventional approach of edge feature concatenation typically employed in standard Graph Neural Networks for graph edge representation.

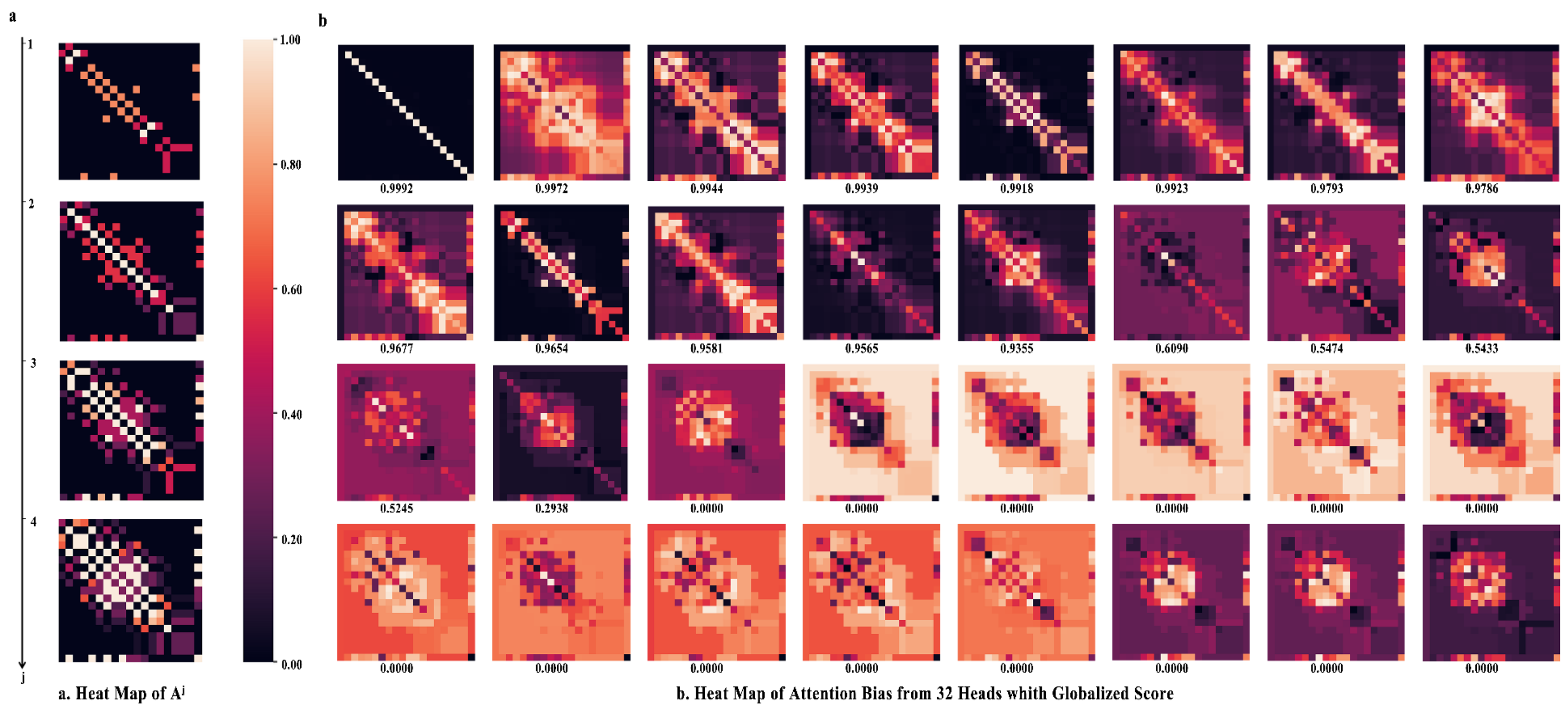


**Supplementary Figure 2. Heat maps of attention bias. a.** The conventional bond adjacent matrix with its j-th power, where j is ranged from 1 to 4. **b.** the bond embeddings extracted from different attention bias heads of our MSMS-GT. The number under each attention map denotes its similarity to the global item that measures the degree of globalization.

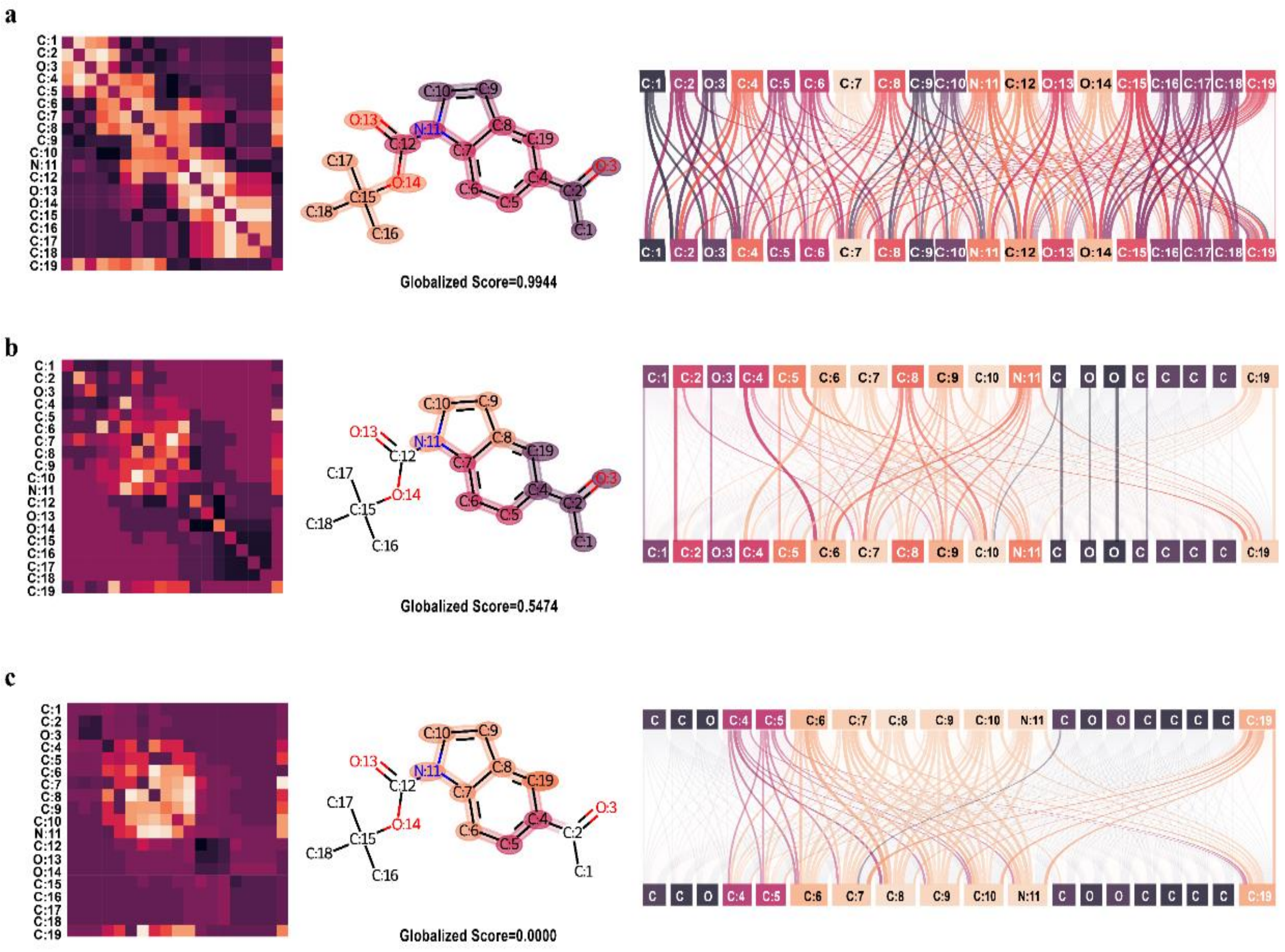


**Supplementary Figure 3. Heat map of attention bias.** The j-hop matrices are normalized. The j-th power of the adjacent matrix can be used to measure the topological relation for the j-hop atom pairs in a molecule. The RV coefficient sorts the heatmaps of attention bias with the global item matrix in descending order. **a.** global-item-dominated heatmap. **b.** local-item-dominated heatmap. **c.** mixed-item-dominated heatmap.

## Supplementary Note 3. Comparations against previous popular methods from the molding view

Given the product graph as $\mathcal{G}_p$ , reactant graph set $\{\mathcal{G}_{r;i}\}_{i=1}^{N_r}$ with size of $N_r$, and template $T$, the comparations are as follows:

1) Direct template-based model $p_{\theta_{cls}}$ with learnable parameters $\theta_{cls}$ simplifies the retrosynthesis as a multi-classification task on templates:

$$p_{\theta_{cls}}\left(\{\mathcal{G}_{r;i}\}_{i=1}^{N_r}\middle|\mathcal{G}_p\right) = p_{\theta_{cls}}(T|\mathcal{G}_p)f_t\left(\{\mathcal{G}_{r;i}\}_{i=1}^{N_r}\middle|T,\mathcal{G}_p\right), \quad (S3.1)$$

where $f_t(\cdot)$ means a deterministic function that transforms the product to reactants given the corresponding template.

2) Integrating more prior knowledge, GLN $p_{\theta_{gln}}$predicts following a structural pipeline:

$$p_{\theta_{gln}}\left(\{\mathcal{G}_{r;i}\}_{i=1}^{N_r}\middle|\mathcal{G}_p\right) = \underbrace{p_{\theta_{rc}}\left(\{\mathcal{G}_{rc;i}\}_{i=1}^{N_{rc}}\middle|\mathcal{G}_p\right)}_{Reaction\ Center}\underbrace{p_{\theta_t}\left(T\middle|\mathcal{G}_p,\{\mathcal{G}_{rc;i}\}_{i=1}^{N_{rc}}\right)}_{Template\ matching}\underbrace{p_{\theta_r}\left(\{\mathcal{G}_{r;i}\}_{i=1}^{N_r}\middle|T,\mathcal{G}_p\right)}_{Reactant\ matching}, \quad (S3.2)$$

3) RetroXpert and G2G follow a simplified two-step paradigm:

$$p_{\theta_{rx}}\left(\{\mathcal{G}_{r;i}\}_{i=1}^{N_r}\middle|\mathcal{G}_p\right) = \underbrace{p_{\theta_{rc}}\left(\{\mathcal{G}_{rc;i}\}_{i=1}^{N_{rc}}\middle|\mathcal{G}_p\right)}_{Reaction\ Center}\underbrace{f_s\left(\{\mathcal{G}_{s;i}\}_{i=1}^{N_s}|\mathcal{G}_p,\{\mathcal{G}_{rc;i}\}_{i=1}^{N_{rc}}\right)}_{Reaction\ Center\ to\ Synthon}\underbrace{p_{\theta_r}\left(\{\mathcal{G}_{r;i}\}_{i=1}^{N_r}\middle|\{\mathcal{G}_{s;i}\}_{i=1}^{N_s}\right)}_{Synthon\ to\ Reactant}, \quad (S3.3)$$

where $f_s(\cdot)$ denotes a deterministic function that transforms the product according to the predicted reaction center and $\{\mathcal{G}_{s;i}\}_{i=1}^{N_s}$ means a set of synthon graph with a size $N_s$. G2G can be understood

as the same as RetroXpert in a general perspective.

4) Directly transforming the synthon to reactants is difficult. So, SemiRetro leverage semi-template for synthon completion, acting more like what GLN does.

$$p_{\theta_{SR}}\left(\{\mathcal{G}_{r;i}\}_{i=1}^{N_r}\middle|\mathcal{G}_p\right)=\underbrace{p_{\theta_{rc}}\left(\{\mathcal{G}_{rc;i}\}_{i=1}^{N_{rc}}\middle|\mathcal{G}_p\right)}_{Reaction\ Center}\underbrace{p_{\theta_{st}}\left(T_{semi}\middle|\mathcal{G}_p,\{\mathcal{G}_{rc;i}\}_{i=1}^{N_{rc}}\right)}_{Semi-Template\ matching}\underbrace{f_{st}\left(\{\mathcal{G}_{r;i}\}_{i=1}^{N_r}\middle|T_{semi},\mathcal{G}_p\right)}_{Reactant\ matching}, \qquad (S3.4)$$

where $f_{st}(\cdot)$ is responsible for the final reactants through predicted semi-template and origin product which is deterministic.

5) GraphRetro introduces the concept of leaving groups, avoiding the direct generation of absent substructure in product to reduce fitting difficulty.

$$p_{\theta_{gr}}\left(\{\mathcal{G}_{r;i}\}_{i=1}^{N_r}\middle|\mathcal{G}_p\right)=\prod_{i=1}^{N_r}\left(\underbrace{p_{\theta_{rc}}(\mathcal{G}_{rc;i}|\mathcal{G}_p)}_{Reaction\ Center}\underbrace{p_{\theta_{lg}}\left(\mathcal{G}_{lg;i}\middle|\mathcal{G}_p,\mathcal{G}_{rc;i},\{\mathcal{G}_{lg;k}\}_{k=0}^{i-1}\right)}_{Leaving\ Group}\underbrace{f_r(\mathcal{G}_{r;i}|\mathcal{G}_p,\mathcal{G}_{lg;i})}_{Reactant}\right), \qquad (S3.5)$$

where $f_r(\cdot)$ is a hand-coded chemical-rule-based function that generates reactants by predicted leaving groups and product.

## Supplementary Note 4. The calculation details of proposed decision process

After training RetroExplainer and inputting the product needed to be synthesis, we will get five probability matrices:

$$\begin{cases} P^{LG} := \mathbb{R}^{N_{size}}, \\ P^{CLG} := \mathbb{R}^{N_{size}}, \\ P^{RC} := \mathbb{R}^{N_{pro} \times N_{pro}}, \\ P^{CT} := \mathbb{R}^{N_{pro} \times N_{gate}}, \\ P^{HC} := \mathbb{R}^{N_{pro} \times N_{state}}, \end{cases} \qquad (S4.1)$$

where LG, CLG, RC, CT, and HC denote leaving group, contrastive leaving group, reaction center, connection, and hydrogen changing respectively. $N_{size}, N_{pro}, N_{gate}, and\ N_{state}$ are the number of collected leaving groups, the product atoms, the gate atoms, and the hydrogen change states, respectively. The gate atom is the leaving group atom that is the 1-hop neighbor of product atoms. In leaving group matching (S-LGM), we set the energy of input product to zero and generate leaving groups (maybe one more) according to $P^{LG}$, and then we have:

$$E_{S-LGM} = -\ln P_k^{LG}, k \in [0, n_{size}], \qquad (S4.2)$$

where $k$ is the index of generated leaving groups. Then in initiating (IT), we suppose all the reaction centers and connections are negative:

$$E_{IT} = \underbrace{E_{LGM}}_{Leaving\ Group\ Item} + \underbrace{\sum_{i}^{N_{pro}} \sum_{j}^{i} -ln\left(1 - P_{ij}^{RC}\right)}_{Reaction\ Center\ Unchanged} + \underbrace{\sum_{i}^{N_{pro}} \sum_{j}^{N_{gate}} -\ln\left(1 - P_{ij}^{LGC}\right)}_{Leaving\ Group\ Connection\ Unchanged} + \underbrace{\sum_{i}^{N_{pro}} -ln\left(P_{i0}^{HC}\right)}_{Hydrogen\ Number\ Unchanged} \qquad (S4.3)$$

Then we search for the potential connections and reaction centers in the flowing connecting (S-LGC) and reaction center prediction (S-RCP) stage:

$$E_{S-LGC} = \sum_{i}^{N_{gate}} \sum_{j}^{N_{ct}} ln\left(1 - P_{ij}^{LGC}\right) - ln\left(P_{ij}^{LGC}\right), \qquad (S4.4)$$

$$E_{S-RCP} = \sum_{m}^{N_{rc}} \sum_{n}^{m} ln(1 - P_{mn}^{RC}) - ln(P_{mn}^{RC}), \qquad (S4.5)$$

where $N_{ct}$ and $N_{rc}$ denote the number of selected connections and reaction centers. The bond types in S-LGC are determined by the message recorded in leaving groups; while the bond types in S-RCP are determined by iterating over every possibility according to the valence rule. After that, we change the hydrogen number and formal charge for every atom to follow the valence rule $Valence(\cdot)$ in hydrogen changing:

$$E_{HC} = \sum_{i}^{N_{pro}} -\ln\left(P_{ij}^{HC}\right) + \ln\left(P_{0j}^{HC}\right), j = Valence(atom_i) \qquad (S4.6)$$

Finally, we have:

$$E_{total} = E_{IT} + E_{S-LGC} + E_{S-RCP} + E_{HC} \qquad (S4.7)$$

The $E_{S-LGC}$ is not contained because it has been already added in $E_{IT}$. Some cases can be seen in Supplementary Figure 1 about the decision path curves according to these energy scores. Also, the pseudocode can be referred as follows:

---

**Supplementary Box 1: Search Tree For $\mathbf{S_N}$2-like Decision Process**

---

**Input:** Product molecular graph $\mathcal{G}_p$, pre-collected leaving group library $\{\mathcal{G}_{lg;i}\}_{i=1}^{n_{size}}$, pretrained RetroExplainer $f_{\theta^*}(\cdot)$;

**Output:** List of reactants with decision actions and energies $S{:}\left\{\left(\{\mathcal{G}_{r;i}, a_i, e_i\}_{i=1}^{n_a}\right)_j\right\}_{j=1}^{|S|}$;

$E \leftarrow 0, Initializing\ S, queue\ Q;$

$Enqueue\left(Q \leftarrow \left(\mathcal{G}_p, a_P, E\right)\right);$

$P^{LG}, P^{RC}, P^{HC} \leftarrow Get\ probabilities\ f_{\theta^*}\left(\mathcal{G}_p\right);$

$E \leftarrow \sum_{i}^{N_{pro}} \sum_{j}^{i} -ln\left(1 - P_{ij}^{RC}\right) + \sum_{i}^{N_{pro}} - ln\left(P_{i0}^{HC}\right) + E;$

$\boldsymbol{For}\ index\ k\ \boldsymbol{in}\ sorted(P^{LG}):$

**Supplementary Box 1: Search Tree For $S_N$2-like Decision Process**

$E \leftarrow E + E_{LGM}, according\ to\ Eq. 12;$

$Enqueue\left(Q \leftarrow \left(\mathcal{G}_{lg;k}, a_{LGM}, E_{LGM}\right)\right);$

$P^{CT} \leftarrow f\left(\mathcal{G}_{r|it}\right), \mathcal{G}_{r|it} \leftarrow\ Concat\left(\mathcal{G}_p, \mathcal{G}_{lg;k}\right);$

$E \leftarrow E + \sum_{i}^{N_{pro}} \sum_{j}^{N_{gate}} -ln\left(1 - P_{ij}^{LGC}\right), current\ E\ is\ equal\ to\ E_{IT}\ in\ Eq. 13;$

$Enqueue\left(Q \leftarrow \left(\mathcal{G}_{r|it}, a_{IT}, E\right)\right);$

$\boldsymbol{For}\ potential\ connection\ \left\{\left(atom_i, atom_j\right)_1, \ldots\right\}\ \boldsymbol{in}\ P^{LGC}:$

  $E \leftarrow E + E_{CT}, according\ to\ Eq. 14;$

  $\mathcal{G}_{r|ct} \leftarrow Connect\left(\mathcal{G}_{r|it}, \left\{\left(atom_i, atom_j\right)_1, \ldots\right\}\right);$

  $Enqueue\left(Q \leftarrow \left(\mathcal{G}_{r|ct}, a_{CT}, E\right)\right);$

  $\boldsymbol{For}\ potential\ reacentre\ \{(atom_m, atom_n, bond\ type)_1, \ldots\}\ \boldsymbol{in}\ P^{RC}:$

    $E \leftarrow E + E_{RC}, according\ to\ Eq. 15;$

    $\mathcal{G}_{r|rc} \leftarrow BondChange\left(\mathcal{G}_{r|ct}, \{(atom_m, atom_n, bond\ type)_1, \ldots\}\right);$

    $Enqueue\left(Q \leftarrow \left(\mathcal{G}_{r|rc}, a_{RC}, E\right)\right);$

    $E \leftarrow E + E_{HC}, according\ to\ Eq. 16;$

    $\mathcal{G}_r \leftarrow HrdrogenChange\left(\mathcal{G}_{r|rc}\right);$

    $Enqueue\left(Q \leftarrow \left(\mathcal{G}_r, a_{HC}, E\right)\right);$

    $Add(S \leftarrow Q);$

    $E \leftarrow E - E_{HC}, Dequeue\left(Q \rightarrow \left(\mathcal{G}_r, a_{HC}, E\right)\right);$

    $E \leftarrow E - E_{RC}, Dequeue\left(Q \rightarrow \left(\mathcal{G}_{r|rc}, a_{RC}, E\right)\right);$

  $\boldsymbol{end\ For};$

  $E \leftarrow E - E_{CT}, Dequeue\left(Q \rightarrow \left(\mathcal{G}_{r|ct}, a_{CT}, E\right)\right);$

**Supplementary Box 1: Search Tree For $S_N2$-like Decision Process**

$\boldsymbol{end\ For}$;

$$E \leftarrow E - \left( \sum_{i}^{N_{pro}} \sum_{j}^{N_{gate}} -ln\left(1 - P_{ij}^{LGC}\right) \right), Dequeue\left(Q \rightarrow \left(\mathcal{G}_{r|it}, a_{IT}, E\right)\right);$$

$$E \leftarrow E - E_{LGM}, Dequeue\left(Q \rightarrow \left(\mathcal{G}_{lg;k}, a_{LGM}, E_{LGM}\right)\right);$$

$S \leftarrow sorted(S, key = e_{na})$;

**return** $S$;

## Supplementary Note 5. Top-12 predictions and decision path cures

The decision curve explains the contribution of each stage for the final decision, consisting of P (input product and set to zero), S-LGM (leaving group matching, decided by the confidence of generated leaving groups), IT (initiating, supposing all the reaction centers unchanged if have reaction centers and the generated leaving groups unconnected if have leaving groups), S-LGC (leaving group connecting, decided by the confidence of selected connection condition), S-RCP (reaction center prediction, decided by the confidence of the selected reaction centers), HC (hydrogen changing, decided by the confidence of the selected hydrogen changing vector ). Usually, the decision path curve presents a pattern like a peak, in which the rising part denotes the confidence of generated leaving groups (lower energy implies a higher confidence) and the potential of all possible reaction centers or connections (higher energy denotes there exits higher confidence of reaction center or connections); while the descending part the confidence of selected connection, reaction centers, and changed hydrogen number (lower energy implies a higher confidence).

Case A and Case C: The diversity of the prediction is mainly concentrated in different leaving groups. This often happens when RetroExplainer is so certain about the predicted reaction centers, according to the parallel lines between LGC and RCP (same reaction centers means same energy drop values in this section).

Case B: Multiple reaction centres, leaving groups, and connection modes result in the diversity of the predictions. The ground truth reactants are ranked as the top-4. And its decision path curve is depicted in Supplementary Figure 4. Case B does not have a descending interval, indicating RetroExplainer thinks a low confidence for the carbamate decomposing to secondary amine and

ester hydrogen carbonate in LGC and HC. The top-5 prediction also occurs similar phenomenon, implying the deficiencies of similar reaction patterns in the training data.

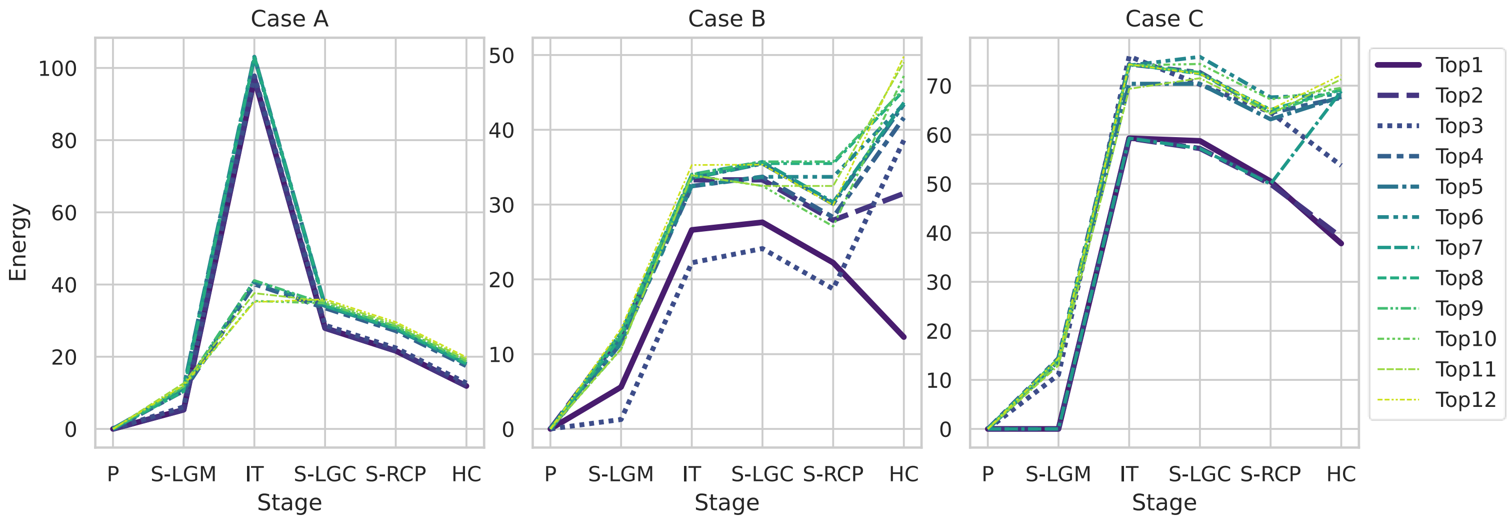


**Supplementary Figure 4. Decision path curve of Top-12 predictions on three cases in Supplementary Table 1.** P, LGM, IT, LGC, RCP, and HC denote product, leaving group matching, initiating, leaving group connecting, reaction center predicting, and hydrogen changing respectively.

## Supplementary Table 1. Top-12 predictions of RetroExplainer.

| Case A | | | |
|---|---|---|---|
| Reaction | 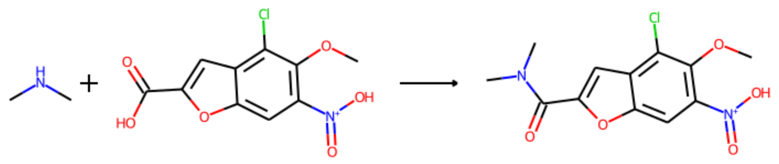 | | |
| Prediction | 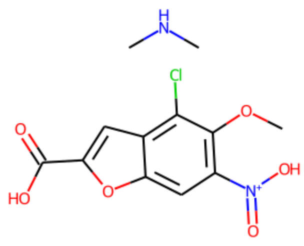 E=11.89 (Ground Truth) [0.0, 5.31, 97.01, 27.93, 21.63, 11.89] | 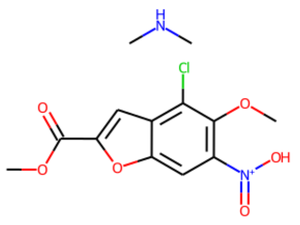 E=11.90 [0.0, 5.31, 97.01, 27.93, 21.63, 11.90] | 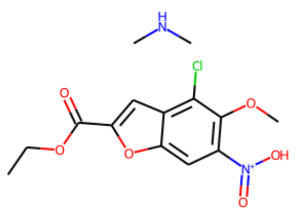 E=12.69 [0.0, 6.11, 97.81, 28.73, 22.43, 12.69] |

| | | | |
|---|---|---|---|
| | 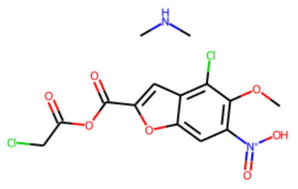<br>E=17.44<br>[0.0, 10.58, 40.11, 33.48, 27.18, 17.44] | 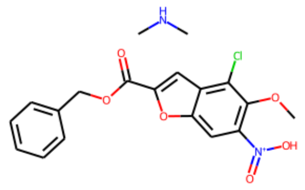<br>E=17.96<br>[0.0, 11.38, 103.08, 34.0, 27.7, 17.96] | 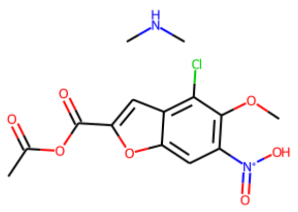<br>E=18.0<br>[0.0, 11.21, 40.92, 34.04, 27.74, 18.0] |
| | 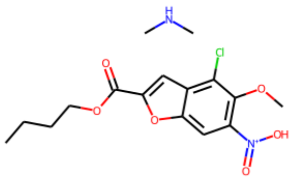<br>E=18.02<br>[0.0, 11.43, 103.13, 34.05, 27.75, 18.02] | 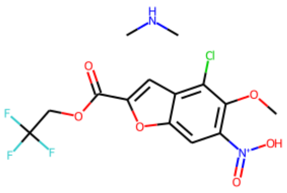<br>E=17.89<br>[0.0, 10.38, 103.21, 34.13, 27.83, 18.09] | 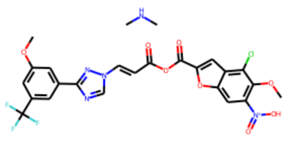<br>E=18.62<br>[0.0, 11.68, 41.14, 34.65, 28.35, 18.62] |
| | 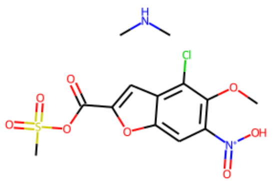<br>E=18.76<br>[0.0, 11.73, 35.45, 34.8, 28.5, 18.76] | 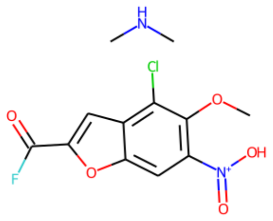<br>E=19.33<br>[0.0, 12.65, 37.61, 35.37, 29.07, 19.33] | 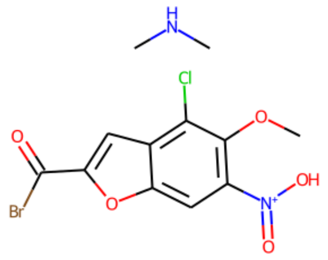<br>E=19.87<br>[0.0, 12.14, 35.15, 35.91, 29.61, 19.87] |
| Case B | | | |
| Reaction | 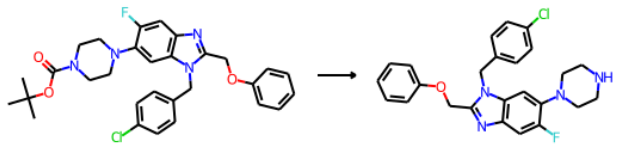 | | |
| Prediction | 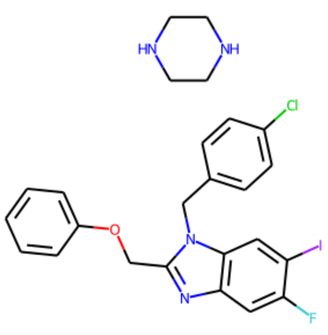 | 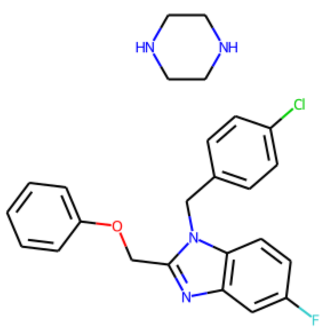 | 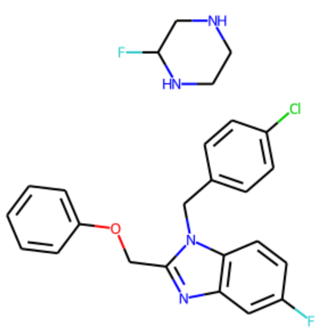 |

| | | | |
|---|---|---|---|
| | E=12.28<br><br>[0.0, 5.6, 26.61, 27.63, 22.23, 12.28] | E=31.49<br><br>[0.0, 12.6, 33.31, 33.31, 27.91, 31.49] | E=38.60<br><br>[0.0, 1.26, 22.22, 24.12, 18.72, 38.60] |
| | 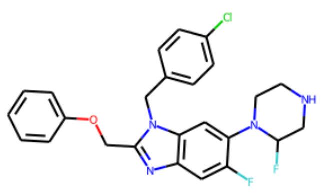<br>E=40.78<br><br>[0.0, 1.26, 22.22, 24.21, 24.21, 40.78] | 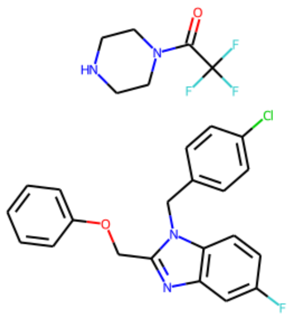<br>E=43.40<br><br>[0.0, 12.29, 33.55, 35.49, 30.09, 43.4] | 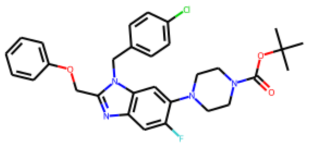<br>E=43.44 (Ground Truth)<br><br>[0.0, 11.5, 32.47, 33.71, 33.71, 43.44] |
| | 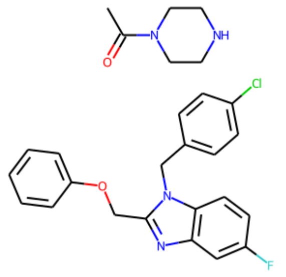<br>E=43.64<br><br>[0.0, 12.9, 34.02, 35.73, 30.33, 43.64] | 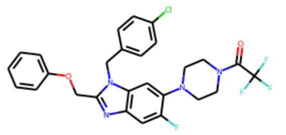<br>E=45.22<br><br>[0.0, 12.29, 33.55, 35.49, 35.49, 45.22] | 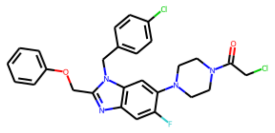<br>E=45.46<br><br>[0.0, 12.9, 34.02, 35.73, 35.73, 45.46] |
| | 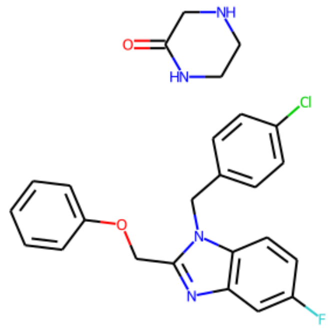<br>E=47.20<br><br>[0.0, 10.68, 33.93, 32.49, 27.09, 47.20] | 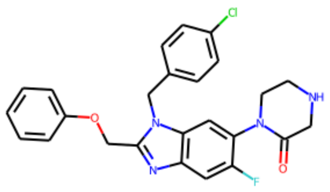<br>E=49.02<br><br>[0.0, 10.68, 33.93, 32.49, 32.49, 49.02] | 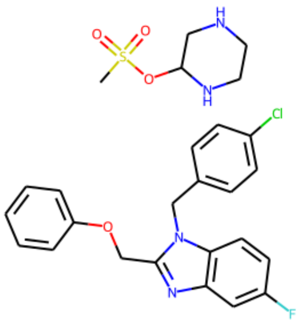<br>E=49.82<br><br>[0.0, 13.41, 35.29, 35.34, 29.94,<br><br>49.82] |
| Case C | | | |
| Reaction | 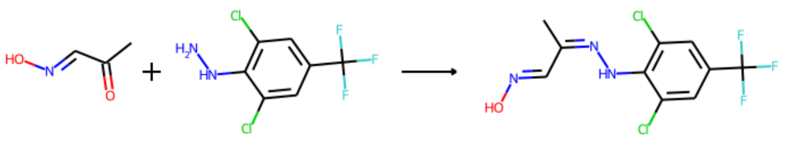 | | |

| | | | |
|---|---|---|---|
| Prediction | 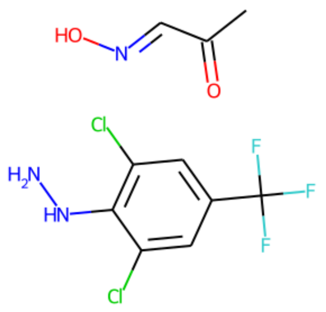<br>E=37.81 (Ground Truth)<br>[0.0, 0.0, 59.29, 58.75, 50.5, 37.81] | 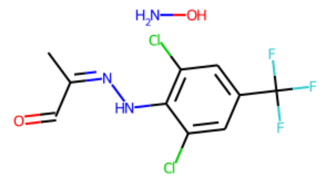<br>E=39.08<br>[0.0, 0.0, 59.29, 57.19, 49.95, 39.08] | 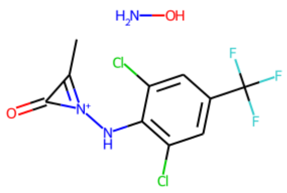<br>E=53.71<br>[0.0, 11.05, 75.98, 70.25, 64.58, 53.71] |
| | 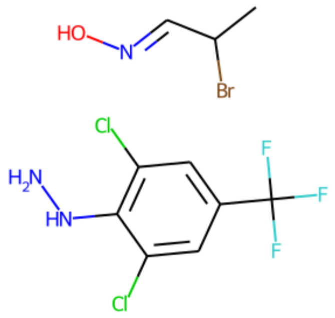<br>E=67.54<br>[0.0, 14.4, 74.35, 72.74, 64.49, 67.54] | 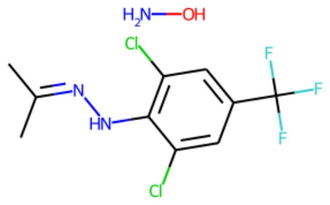<br>E=67.73<br>[0.0, 13.89, 70.38, 70.38, 63.14, 67.73] | 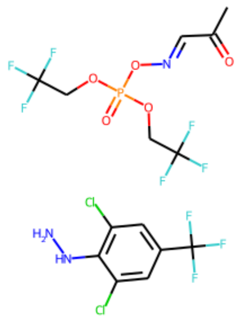<br>E=68.19<br>[0.0, 13.63, 74.13, 75.89, 67.64, 68.19] |
| | 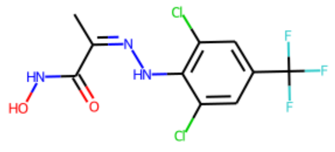<br>E=68.88<br>[0.0, 0.0, 59.29, 57.19, 49.95, 68.88] | 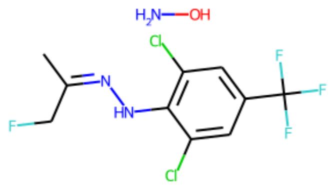<br>E=69.14<br>[0.0, 14.4, 74.35, 72.4, 65.16, 69.14] | 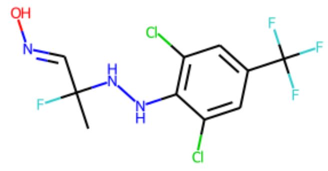<br>E=69.35<br>[0.0, 14.4, 74.35, 72.74, 64.49, 69.35] |
| | 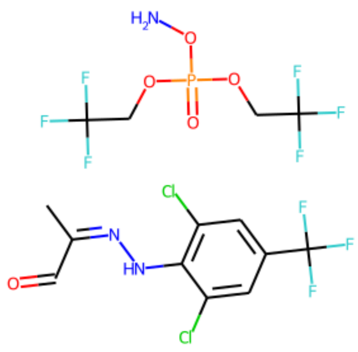<br>E=69.56 | 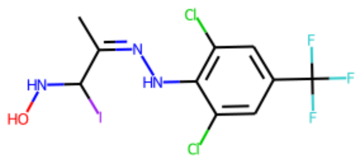<br>E=71.30 | 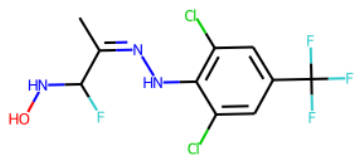<br>E=72.18 |

| | | | |
|---|---|---|---|
| | [0.0, 13.63, 74.13, 74.43, 67.19, 69.56] | [0.0, 12.95, 69.35, 71.52, 64.28, 71.30] | [0.0, 14.4, 74.35, 72.4, 65.16, 72.18] |

## Supplementary Note 6. Details of reranking existing approaches for performance improvement

RetroExplainer is capable of explaining and scoring reactants after identifying corresponding events including conditions of leaving group matching, bond changes, connections and hydrogens. To make this function more clearly, we provide a pseudocode as follows:

---

**Supplementary Box 2: Explaining and scoring for exogenous predictions**

---

**Input:** Product molecular graph $\mathcal{G}_p$, pre-collected leaving group library $\{\mathcal{G}_{lg;i}\}_{i=1}^{n_{size}}$, pretrained RetroExplainer $f_{\theta^*}(\cdot)$, exogenous predictions graph $\{\mathcal{G}_{r;i}\}_{i=1}^{N(pred)}$;

**Output:** List of reactants with decision actions and energies $S\!:\left\{\left(\{\mathcal{G}_{r;i}, a_i, e_i\}_{i=1}^{n_a}\right)_j\right\}_{j=1}^{|S|}$;

$P^{LG}, P^{RC}, P^{HC} \leftarrow Get\ probabilities\ f_{\theta^*}(\mathcal{G}_p);$

$\boldsymbol{For}\ prediction\ \mathcal{G}_{r;i}\ \boldsymbol{in}\ \{\mathcal{G}_{r;i}\}_{i=1}^{N(pred)}$:

$\quad E \leftarrow 0, Initializing\ queue\ Q;$

$\quad Enqueue\left(Q \leftarrow (\mathcal{G}_p, a_P, E)\right);$

$\quad \mathcal{G}_{lg} \leftarrow CheckLG(\mathcal{G}_{r;i}, \mathcal{G}_p);$

$\quad \boldsymbol{If}\ \mathcal{G}_{lg} \in \{\mathcal{G}_{lg;i}\}_{i=1}^{n_{size}}$:

$\quad\quad k \leftarrow Index\left(\{\mathcal{G}_{lg;i}\}_{i=1}^{n_{size}}, \mathcal{G}_{lg}\right);$

$\quad \boldsymbol{else}$:

$\quad\quad k \leftarrow argmax(P^{CLG}), P^{CLG} \leftarrow f_{\theta^*}(\mathcal{G}_{lg});$

$\quad \boldsymbol{end\ If};$

$\quad E \leftarrow E + E_{LGM}, according\ to\ Eq.12\ and\ k$:

$\quad Enqueue\left(Q \leftarrow (\mathcal{G}_{lg}, a_{LGM}, E)\right);$

$\quad P^{CT} \leftarrow f_{\theta^*}(\mathcal{G}_{p||lg});$

**Supplementary Box 2: Explaining and scoring for exogenous predictions**

$E \leftarrow E_{IT}, according\ to\ Eq. 13;$

$Enqueue\left(Q \leftarrow \left(\mathcal{G}_{\lg\ ||LG}, a_{IT}, E_{IT}\right)\right)$

$\mathcal{G}_{r|ct} \leftarrow CheckCT\left(\mathcal{G}_{r;i}, \mathcal{G}_p\ \right);$

$E \leftarrow E + E_{LGC}, according\ to\ Eq. 14\ and\ \mathcal{G}_{r|ct}.$

$Enqueue\left(Q \leftarrow \left(\mathcal{G}_{r|ct}, a_{CT}, E\right)\right);$

$\mathcal{G}_{r|rc} \leftarrow CheckRC\left(\mathcal{G}_{r;i}, \mathcal{G}_p\ \right);$

$E \leftarrow E + E_{RC}, according\ to\ Eq. 15\ and\ \mathcal{G}_{r|rc};$

$Enqueue\left(Q \leftarrow \left(\mathcal{G}_{r|rc}, a_{RC}, E\right)\right);$

$\mathcal{V}_{r|hc} \leftarrow CheckHC\left(\mathcal{G}_{r;i}, \mathcal{G}_p\ \right);$

$E \leftarrow E + E_{HC}, according\ to\ Eq. 16\ and\ \mathcal{V}_{r|hc};\ ;$

$Enqueue\left(Q \leftarrow \left(\mathcal{G}_r, a_{HC}, E\right)\right);$

$Add(S \leftarrow Q);$

$S \leftarrow sorted(S, key = e_{na});$

**return** $S$;

## Supplementary Note 7. The multi-planning algorithm we used and the 101 pathways we found

We provided a convenient interface that integrated the efficient Retro*[2] algorithm to guarantee the end reactants are purchasable. In detail, we developed two versions based on Retro*-0 and Retro* in which we merely replaced the origin cost scores as our energy scores. Considering the difficulty in extracting and accessing the synthesis route dataset based on the USPTO-FULL, we directly adopted the original pretrained neural parameters when using the Retro* version. Additionally, we used about 231 million commercially available building blocks collected from eMolecules[3] to determine whether the end reactants are purchasable.

To verify the effectiveness of the 101 pathways exhibited in Supplementary Information Data 1, we used SciFinder$^{N}$ engine to manually search for the similar reactions that had been reported in literatures before. The results can be observed in Supplementary Information Data 2. Among the results, 150/176 single-step predictions were similar to reaction patterns reported previously. Whether the reaction has similar patterns is determined by whether the searching result has the similarity entry for the enquiry reaction in the SciFinder$^{n}$ webpage.

## Supplementary Note 8. Scatters of t-SNE using nine different random seeds

To mitigate the issue of non-deterministic clustering caused by the random seed, we have included an additional nine embedded scatter plots in the t-SNE plots. These scatter plots were generated using different random seeds ranging from 132 to 124. The resulting figures are shown below.

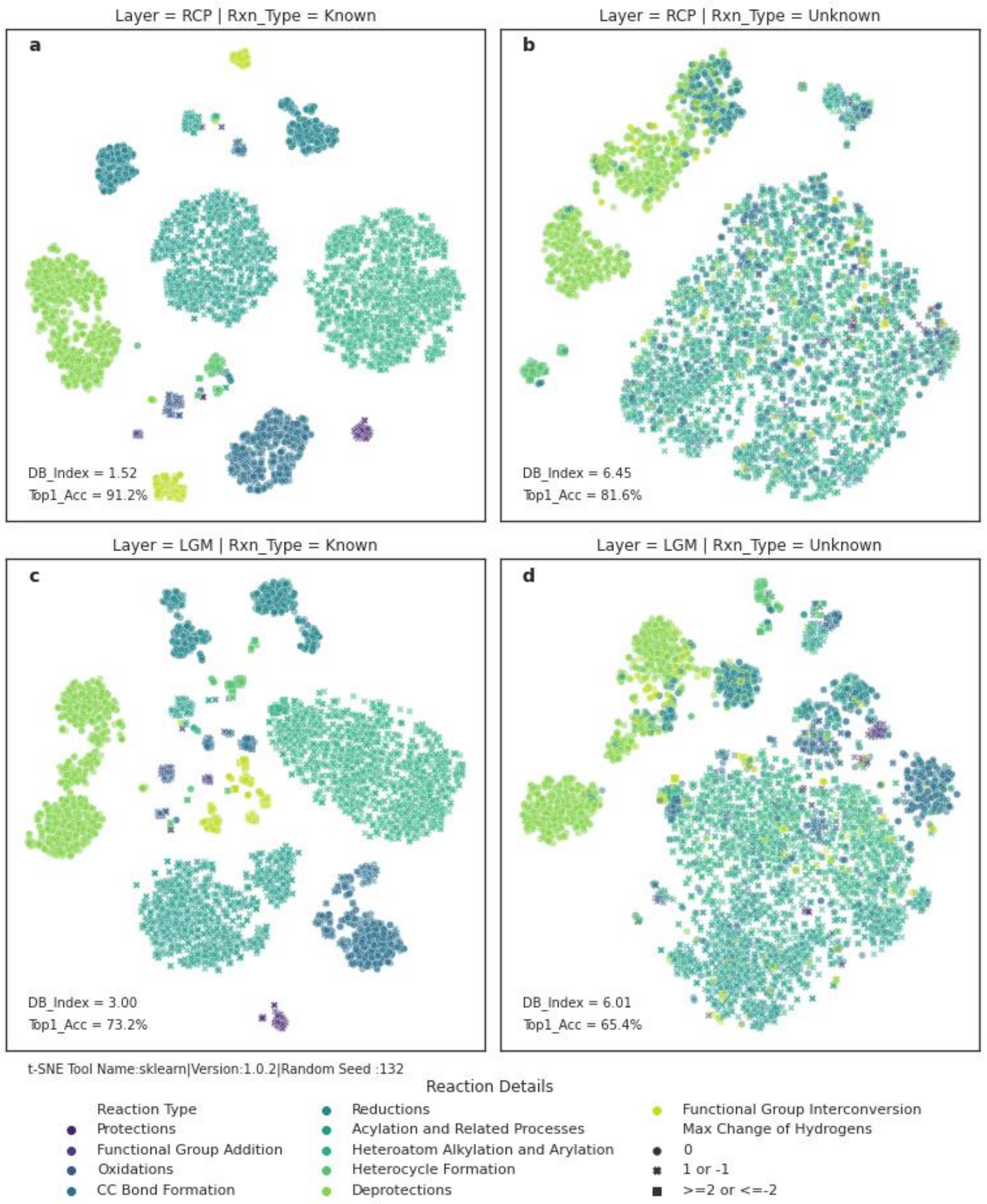


**Supplementary Figure 5. Distributions of t-SNE from hidden layers of RetroExplainer with random seed of 132. a-d.** Hidden features are extracted based on the following criteria: 1) the determination of whether the reaction type is known, and 2) the identification of the source of the hidden features (whether from the RCP layer or LGM

layer). Subsequently, these features are compressed into two dimensions using t-SNE tools. Distinct reaction types are indicated by varying colors, while diverse styles are established based on the maximum hydrogen number change.

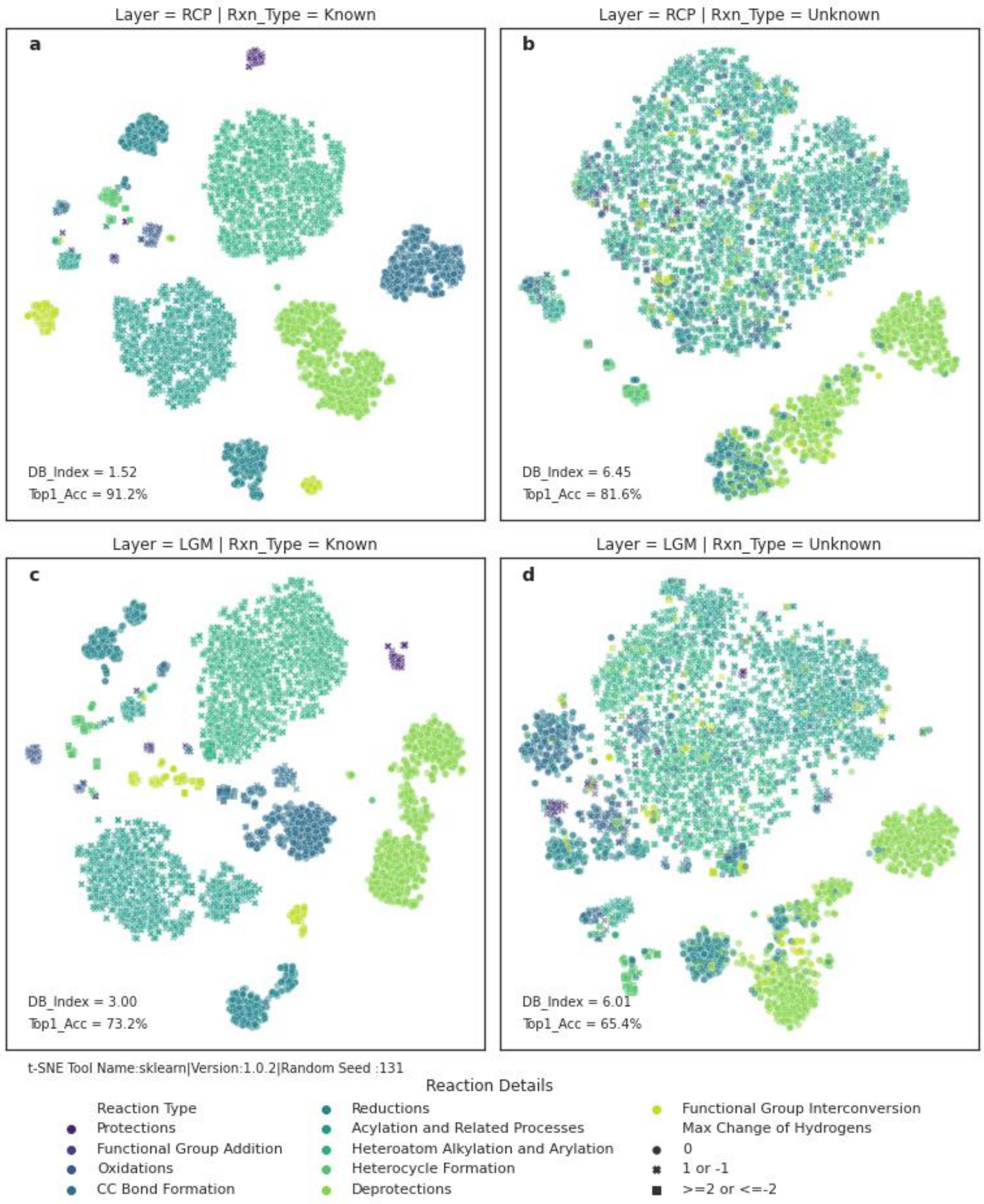


**Supplementary Figure 6. Distributions of t-SNE from hidden layers of RetroExplainer with random seed of 131. a-d.** Hidden features are extracted based on the following criteria: 1) the determination of whether the reaction type is known, and 2) the identification of the source of the hidden features (whether from the RCP layer or LGM layer). Subsequently, these features are compressed into two dimensions using t-SNE tools. Distinct reaction types

are indicated by varying colors, while diverse styles are established based on the maximum hydrogen number change.

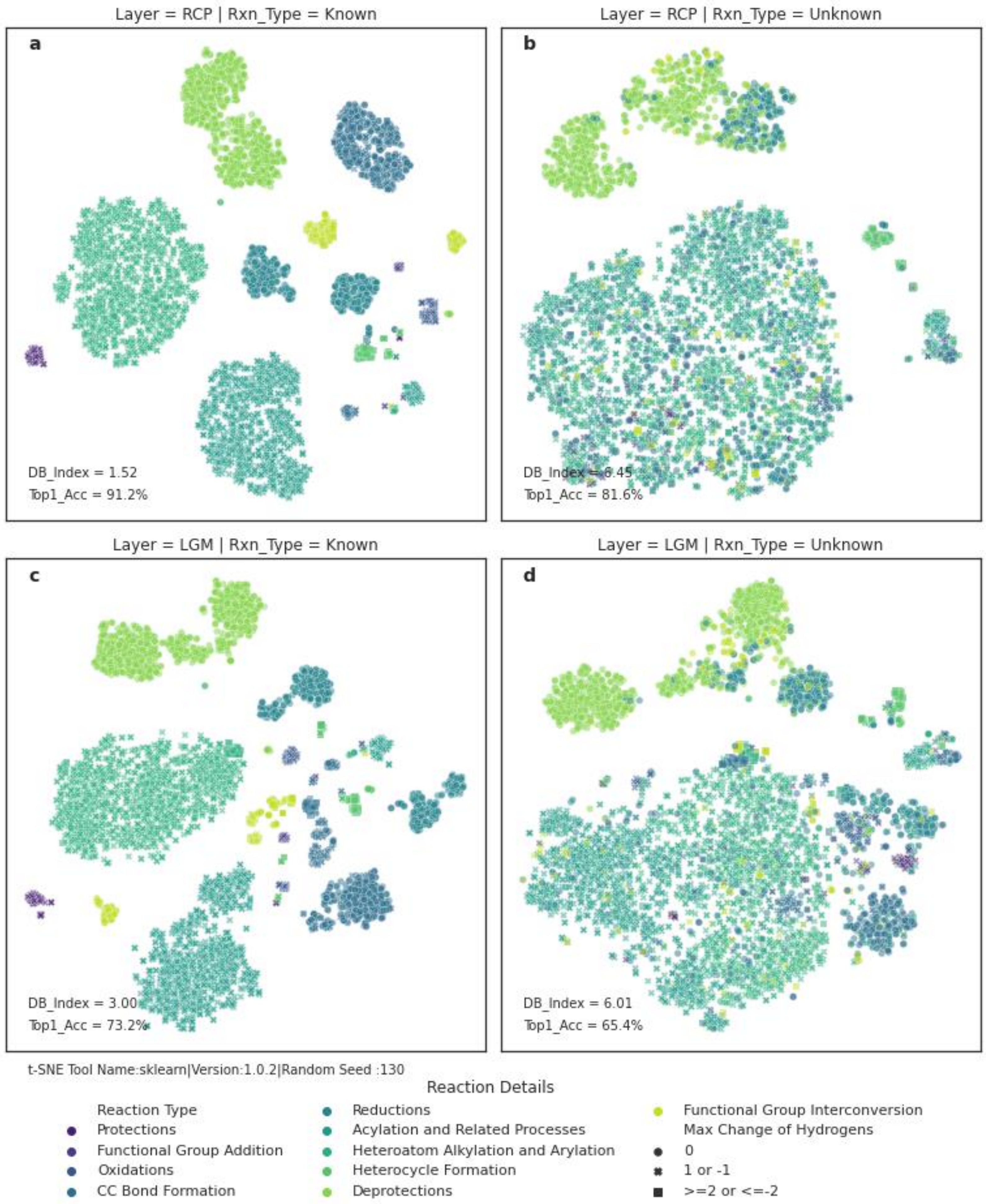


**Supplementary Figure 7. Distributions of t-SNE from hidden layers of RetroExplainer with random seed of 130. a-d.** Hidden features are extracted based on the following criteria: 1) the determination of whether the reaction type is known, and 2) the identification of the source of the hidden features (whether from the RCP layer or LGM layer). Subsequently, these features are compressed into two dimensions using t-SNE tools. Distinct reaction types are indicated by varying colors, while diverse styles are established based on the maximum hydrogen number change.

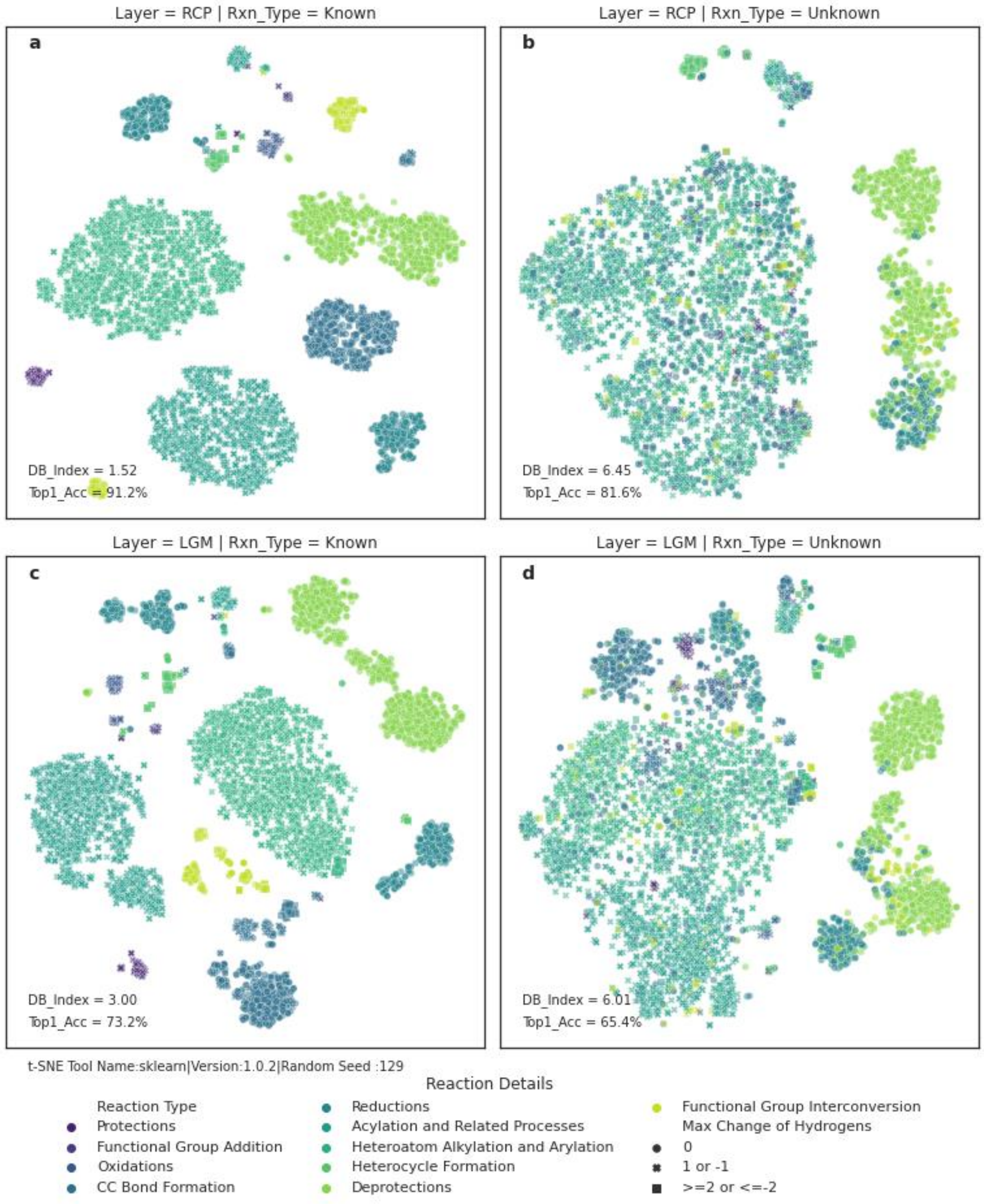


**Supplementary Figure 8. Distributions of t-SNE from hidden layers of RetroExplainer with random seed of 129. a-d.** Hidden features are extracted based on the following criteria: 1) the determination of whether the reaction type is known, and 2) the identification of the source of the hidden features (whether from the RCP layer or LGM layer). Subsequently, these features are compressed into two dimensions using t-SNE tools. Distinct reaction types are indicated by varying colors, while diverse styles are established based on the maximum hydrogen number change.

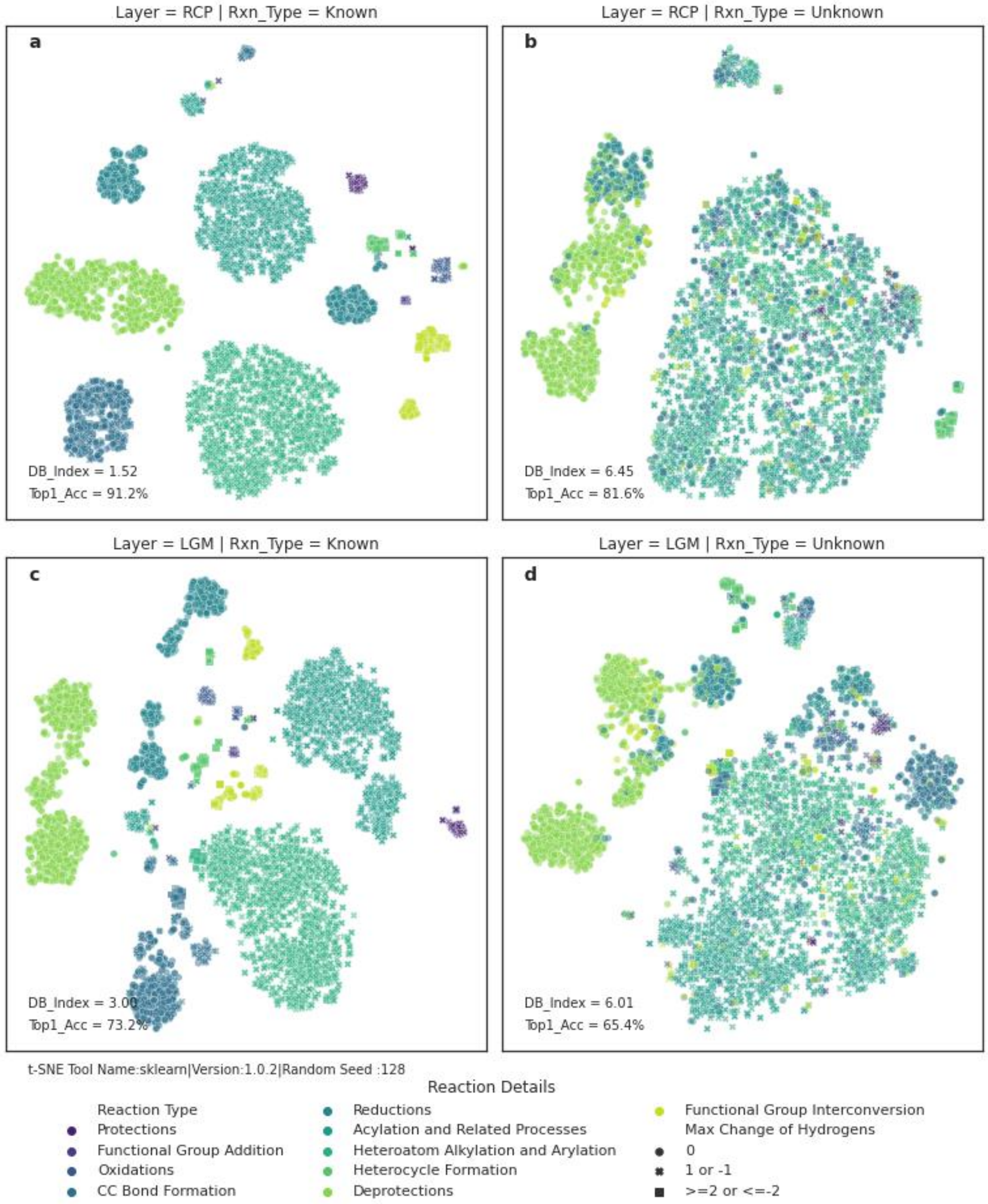


**Supplementary Figure 9. Distributions of t-SNE from hidden layers of RetroExplainer with random seed of 128. a-d.** Hidden features are extracted based on the following criteria: 1) the determination of whether the reaction type is known, and 2) the identification of the source of the hidden features (whether from the RCP layer or LGM layer). Subsequently, these features are compressed into two dimensions using t-SNE tools. Distinct reaction types are indicated by varying colors, while diverse styles are established based on the maximum hydrogen number change.

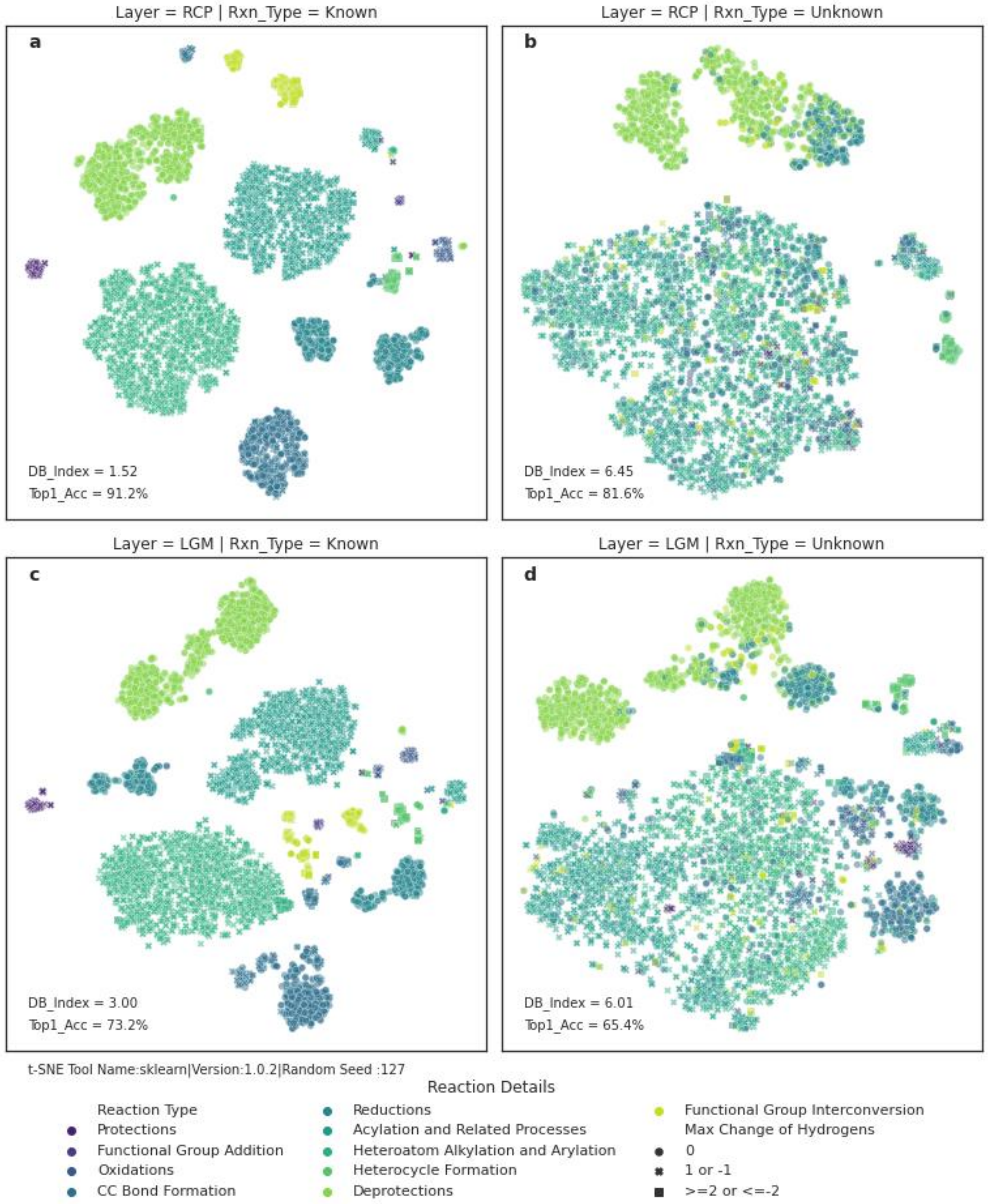


**Supplementary Figure 10. Distributions of t-SNE from hidden layers of RetroExplainer with random seed of 127. a-d.** Hidden features are extracted based on the following criteria: 1) the determination of whether the reaction type is known, and 2) the identification of the source of the hidden features (whether from the RCP layer or LGM layer). Subsequently, these features are compressed into two dimensions using t-SNE tools. Distinct reaction types are indicated by varying colors, while diverse styles are established based on the maximum hydrogen number change.

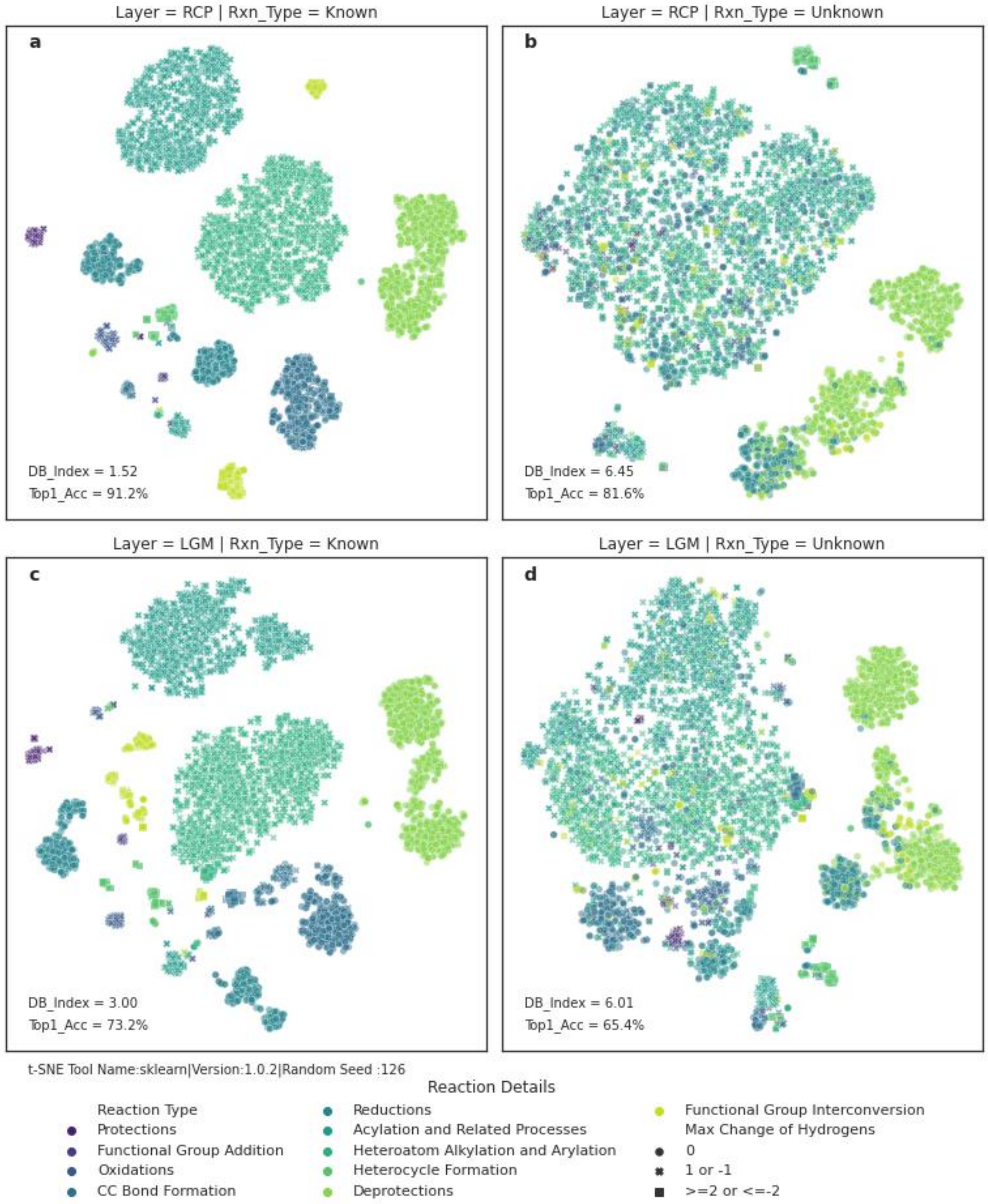


**Supplementary Figure 11. Distributions of t-SNE from hidden layers of RetroExplainer with random seed of 126. a-d.** Hidden features are extracted based on the following criteria: 1) the determination of whether the reaction type is known, and 2) the identification of the source of the hidden features (whether from the RCP layer or LGM layer). Subsequently, these features are compressed into two dimensions using t-SNE tools. Distinct reaction types are indicated by varying colors, while diverse styles are established based on the maximum hydrogen number change.

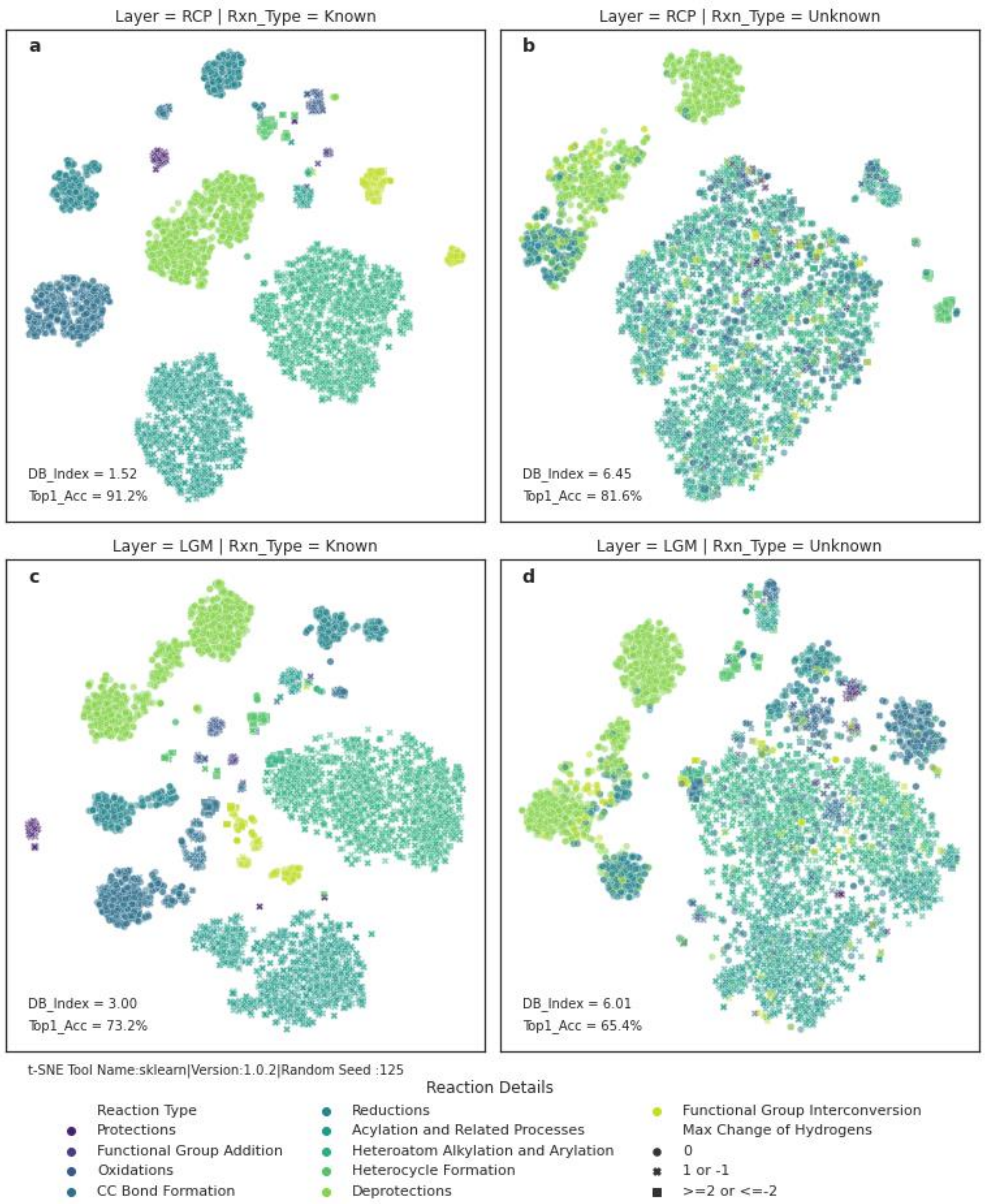


**Supplementary Figure 12. Distributions of t-SNE from hidden layers of RetroExplainer with random seed of 125. a-d.** Hidden features are extracted based on the following criteria: 1) the determination of whether the reaction type is known, and 2) the identification of the source of the hidden features (whether from the RCP layer or LGM layer). Subsequently, these features are compressed into two dimensions using t-SNE tools. Distinct reaction types are indicated by varying colors, while diverse styles are established based on the maximum hydrogen number change.

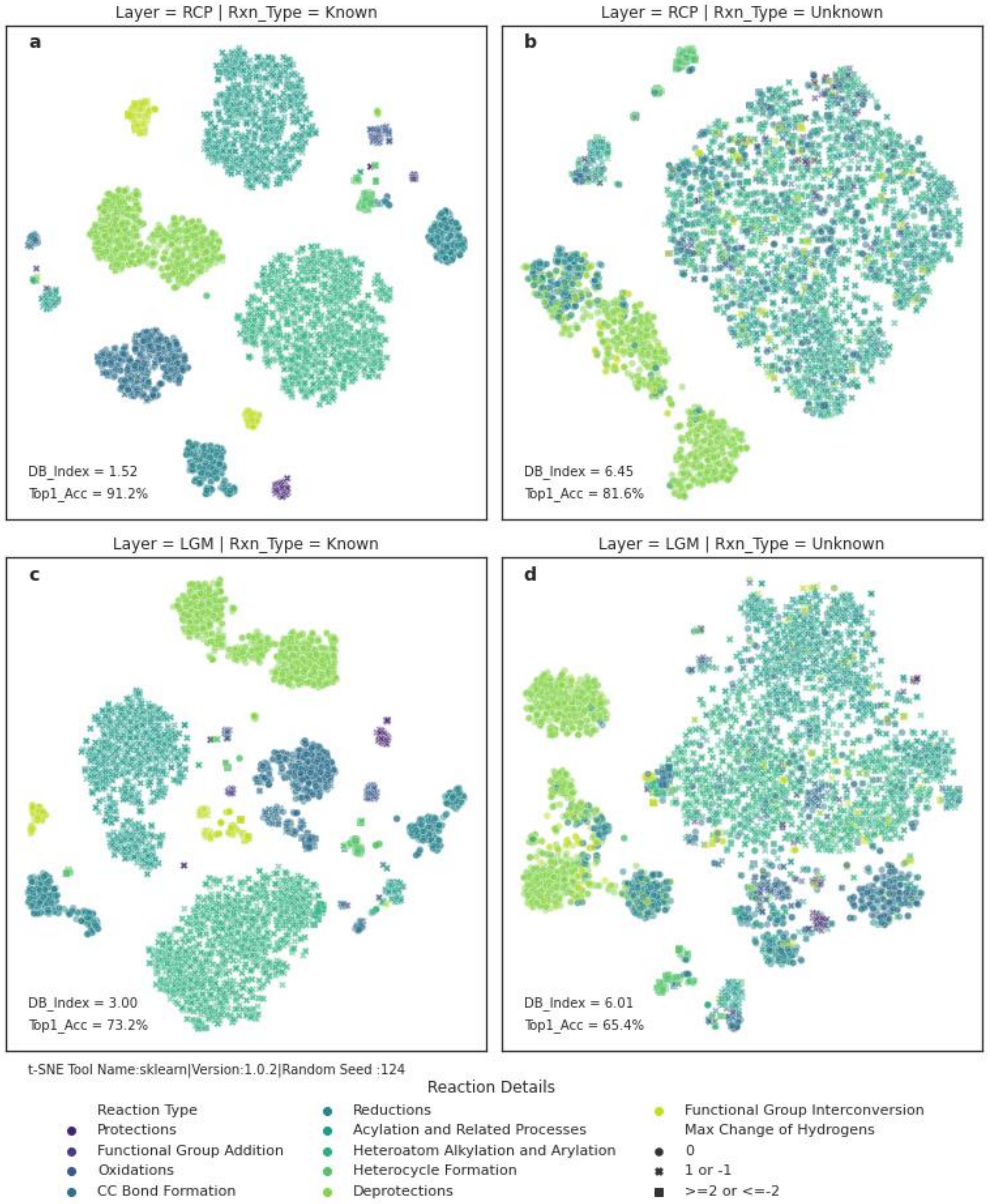


**Supplementary Figure 13. Distributions of t-SNE from hidden layers of RetroExplainer with random seed of 124. a-d.** Hidden features are extracted based on the following criteria: 1) the determination of whether the reaction type is known, and 2) the identification of the source of the hidden features (whether from the RCP layer or LGM layer). Subsequently, these features are compressed into two dimensions using t-SNE tools. Distinct reaction types are indicated by varying colors, while diverse styles are established based on the maximum hydrogen number change.

## Supplementary Note 9.  Prediction cases on rare leaving groups

**Supplementary Table 2. Prediction cases on rare leaving groups**

<table>
<tr><td colspan="4">Case A</td></tr>
<tr><td>Reaction</td><td colspan="3">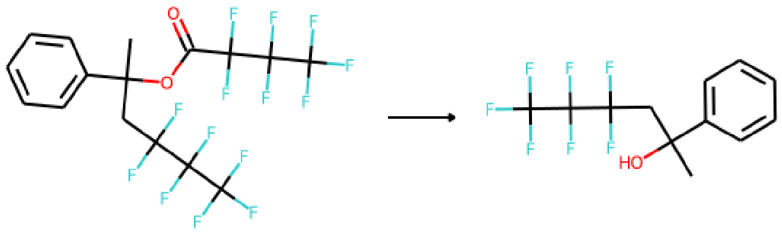</td></tr>
<tr><td rowspan="3">Prediction</td><td>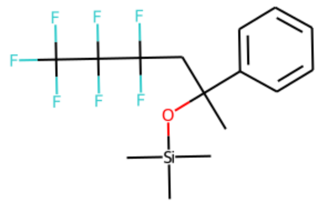<br>E=19.87<br>[0.0, 10.07, 37.49, 25.4, 25.4, 19.87]</td><td>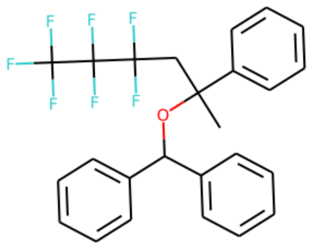<br>E=21.12<br>[0.0, 10.45, 25.46, 26.65, 26.65, 21.12]</td><td>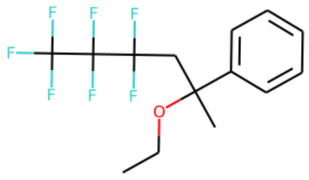<br>E=21.38<br>[0.0, 10.52, 27.93, 26.91, 26.91,21.38]</td></tr>
<tr><td>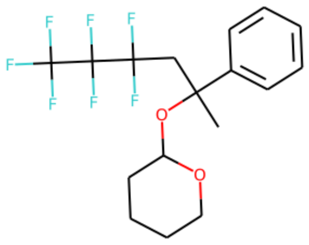<br>E=21.66<br>[0.0, 11.51, 30.66, 27.19, 27.19, 21.66]</td><td>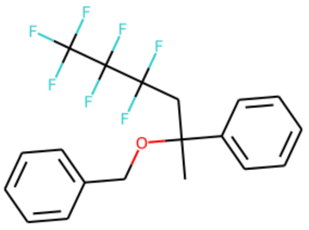<br>E=22.22<br>[0.0, 11.48, 29.54, 27.75, 27.75, 22.22]</td><td>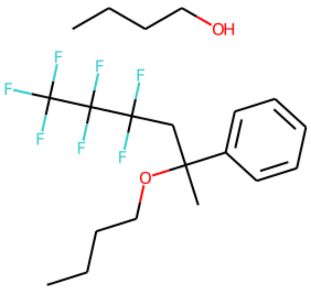<br>E=27.19<br>[0.0, 11.22, 29.21, 29.47, 27.19, 27.19]</td></tr>
<tr><td>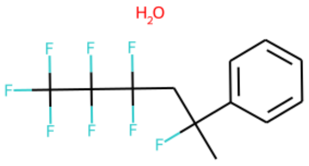<br>E=30.87<br>[0.0, 8.08, 22.14, 22.77, 20.49,</td><td>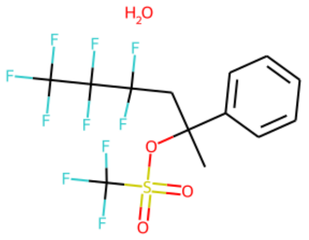<br>E=34.95<br>[0.0, 9.2, 28.8, 26.85, 24.57, 34.95]</td><td>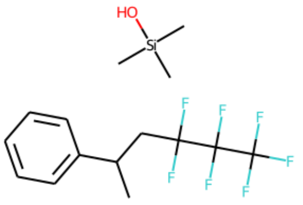<br>E=35.98<br>[0.0, 10.07, 37.49, 25.4, 23.12,</td></tr>
</table>

| | | | |
|---|---|---|---|
| | 30.87] | | 35.98] |
| | 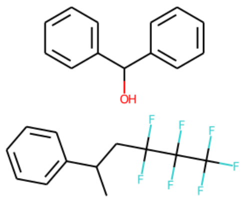 E=37.23 [0.0, 10.45, 25.46, 26.65, 24.37, 37.23] | 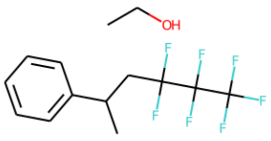 E=37.49 [0.0, 10.52, 27.93, 26.91, 24.63, 37.49] | 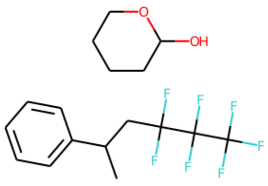 E=37.77 [0.0, 11.51, 30.66, 27.19, 24.91, 37.77] |
| Case B | | | |
| Reaction | 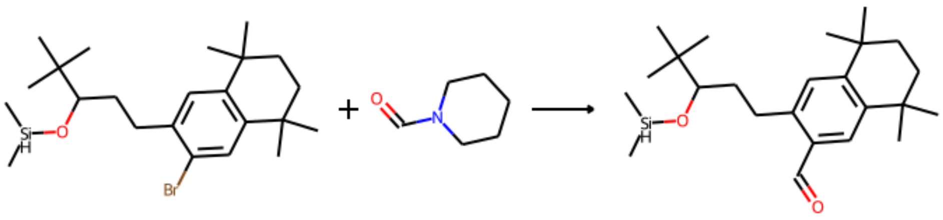 | | |
| Prediction | 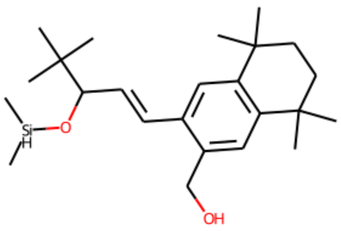 E=12.28 [0.0, 1.35, 9.53, 9.53, 9.23, 14.55] | 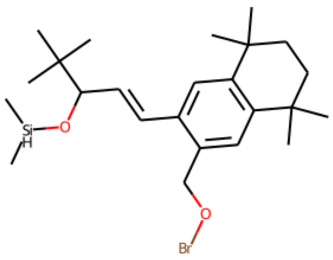 E=14.97 [0.0, 3.02, 14.08, 11.46, 11.16, 14.97] | 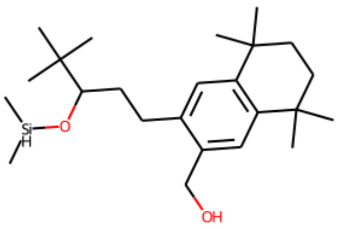 E=15.08 [0.0, 1.35, 9.53, 9.53, 10.49, 15.08] |
| | 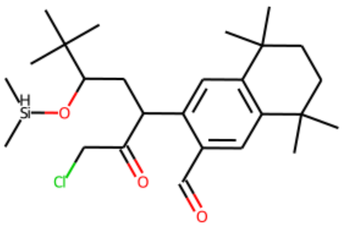 E=15.39 [0.0, 4.64, 17.43, 19.37, 19.37, | 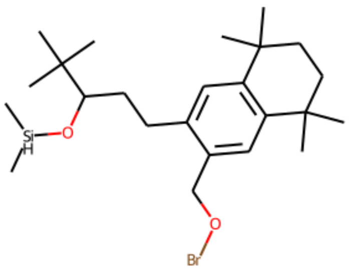 E=15.50 [0.0, 3.02, 14.08, 11.46, 12.42, | 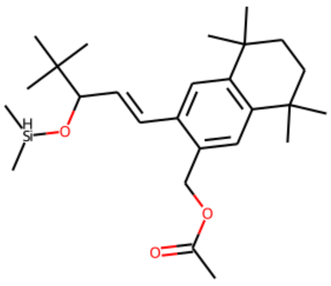 E=16.20 [0.0, 2.64, 11.54, 12.69, 12.39, |

| | | | |
|---|---|---|---|
| | 15.39] | 15.50] | 16.2] |
| | 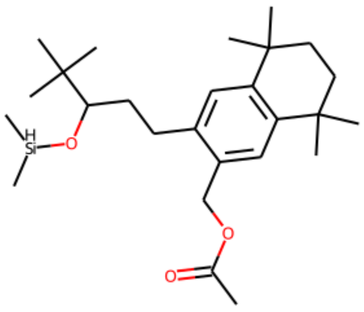<br>E=16.73<br>[0.0, 2.64, 11.54, 12.69, 13.65, 16.73] | 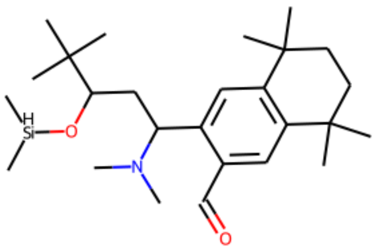<br>E=16.89<br>[0.0, 6.1, 20.2, 20.87, 20.87, 16.89] | 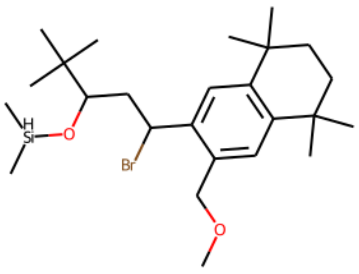<br>E=17.27<br>[0.0, 6.32, 30.07, 17.21, 18.17, 17.27] |
| | 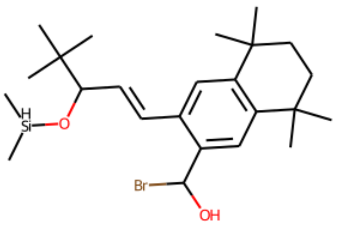<br>E=17.57<br>[0.0, 3.02, 14.08, 15.63, 15.33, 17.57] | 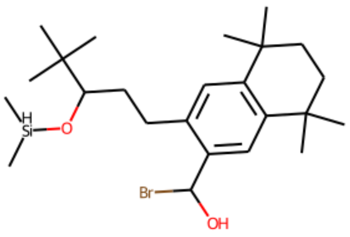<br>E=18.10<br>[0.0, 3.02, 14.08, 15.63, 16.59, 18.1] | 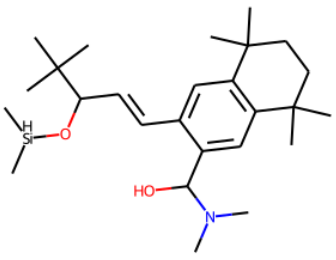<br>E=19.24<br>[0.0, 6.1, 20.2, 17.3, 17.0, 19.24] |
| Case C | | | |
| Reaction | 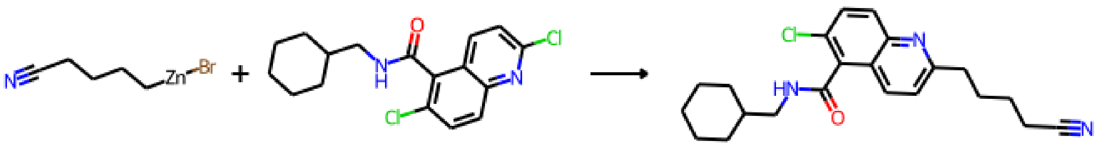 | | |
| Prediction | 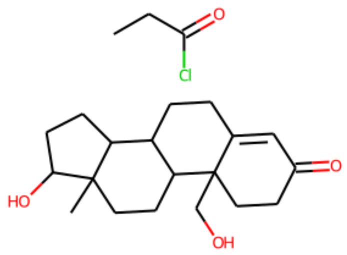<br>E=7.19 | 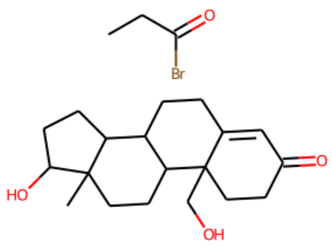<br>E=8.63 | 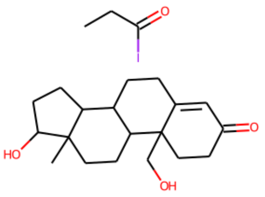 |

| | | | |
|---|---|---|---|
| | [0.0, 0.54, 23.6, 19.52, 13.55, 7.19] | [0.0, 1.97, 24.61, 20.96, 14.99, 8.63] | E=53.71<br>[0.0, 11.05, 75.98, 70.25, 64.58, 53.71] |
| | 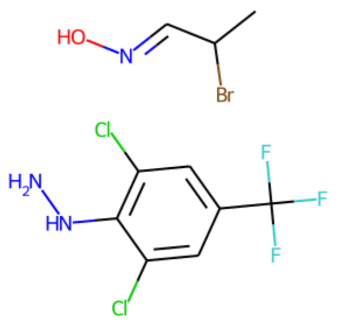<br>E=7.19<br>[0.0, 0.54, 23.6, 19.52, 13.55, 7.19] | 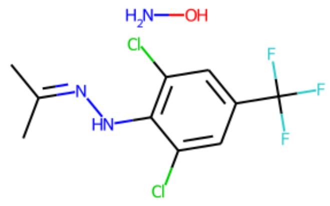<br>E=67.73<br>[0.0, 13.89, 70.38, 70.38, 63.14, 67.73] | 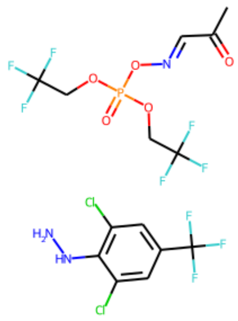<br>E=68.19<br>[0.0, 13.63, 74.13, 75.89, 67.64, 68.19] |
| | 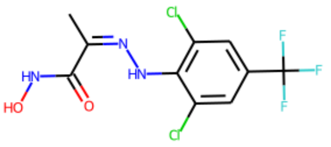<br>E=68.88<br>[0.0, 0.0, 59.29, 57.19, 49.95, 68.88] | 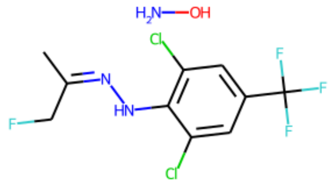<br>E=69.14<br>[0.0, 14.4, 74.35, 72.4, 65.16, 69.14] | 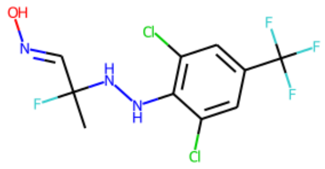<br>E=69.35<br>[0.0, 14.4, 74.35, 72.74, 64.49, 69.35] |
| | 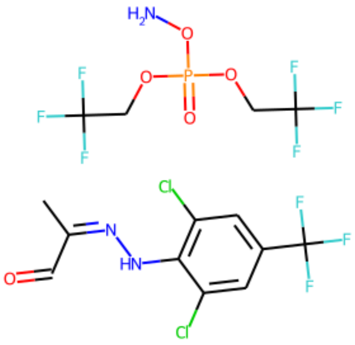<br>E=69.56<br>[0.0, 13.63, 74.13, 74.43, 67.19, 69.56] | 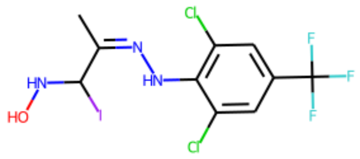<br>E=71.30<br>[0.0, 12.95, 69.35, 71.52, 64.28, 71.30] | 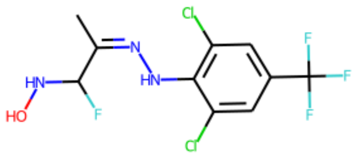<br>E=72.18<br>[0.0, 14.4, 74.35, 72.4, 65.16, 72.18] |

## Supplementary Note 10. Cases on non-leaving-group reactions

In our collected leaving group lists, there is a special empty leaving group which are responsible for those reaction types without leaving groups. Some cases for mentioned reaction types are listed as below:

**Supplementary Table 3. Cases for the reaction types that don't need leaving groups.**

| Reactions | | Atom Indices of Product |
|---|---|---|
| Structures | 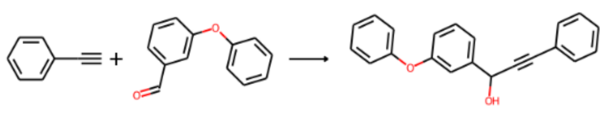 | 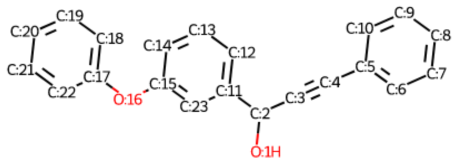 |
| Type | C-C Bond Formation, Reduction | |
| Decision Steps | 1. Select Leaving Group with Index 6 and Cost 0.61,<br>2. Initial Cost:36.19,<br>3. Replace Bonds: between 1 and 2, from Bond Type 1.0 to Bond Type 2.0 with Cost -8.71,<br>4. Remove Bonds: between 2 and 3, with Bond Type 1.0 and Cost -8.03,<br>5. H number change -1 cost of atom 1: 0.01,<br>6. H number change 1 cost of atom 3: 2.46. | |
| Structures | 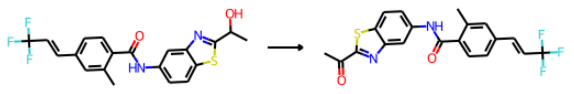 | 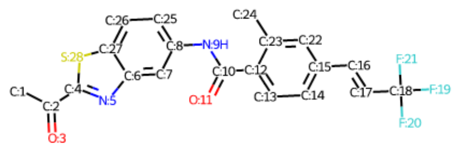 |
| Type | Oxidation | |
| Decision Steps | 1. Select Leaving Group with Index 6 and Cost 0.06,<br>2. Initial Cost:6.48, | |

<table>
<tr><td></td><td colspan="2">3. Replace Bonds: between 2 and 3, from Bond Type 2 to Bond Type 1 with Cost -2.65,<br>4. H number change 1 cost of atom 2: 0.26,<br>5. H number change 1 cost of atom 3: 0.50.</td></tr>
<tr><td>Structures</td><td>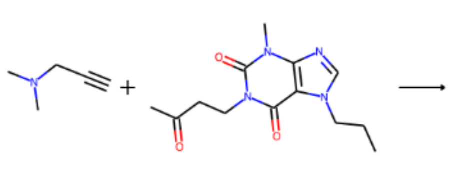</td><td>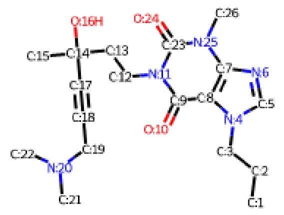</td></tr>
<tr><td>Type</td><td colspan="2">C-C Bond Formation, Reduction</td></tr>
<tr><td>Decision Steps</td><td colspan="2">1. Select Leaving Group with Index 6 and Cost 0.94,<br>2. Initial Cost:12.10,<br>3. Remove Bonds: between 11 and 12, with Bond Type 1 and Cost -0.51,<br>4. Replace Bonds: between 14 and 16, from Bond Type 1 to Bond Type 2 with Cost -3.20,<br>5. Remove Bonds: between 14 and 17, with Bond Type 1 and Cost -0.83,<br>6. H number change 1 cost of atom 11: 0.21,<br>7. H number change 1 cost of atom 12: 16.32,<br>8. H number change -1 cost of atom 16: 1.15,<br>9. H number change 1 cost of atom 17: 0.03</td></tr>
</table>

The index of the empty leaving group is 6. The bond type 1, 1.5, 2, 3 refers single, aroma, double, triple bond, respectively.

## Supplementary Note 11. Preliminaries and Notes

**GNNs.** Let $\mathcal{G} := (\mathcal{V}, \mathcal{A})$ denote a graph where $\mathcal{V}$ is vertex set with size $N = |\mathcal{V}|$, $\mathcal{A} \in \{0,1\}^{N\times N\times K}$ is 0-1 adjacent matrix with $K$ properties. GNNs aim to acquire node-, edge- or graph-level representations depend on specific task. Generally, current GNNs[4-7] follow a learning framework which aggregates neighbor messages and updates own feature iteratively. Let $h_v^{(l)}$ denote representation of node $v$ at the $l$-th layer, and $h_v^{(0)}$ is initialized to the feature vector of $v$. Then, the $l$-th iteration can be calculated as following framework:

$$m_a^{(l)} = AGGREGATE_N^{(l)}\left(\left\{\tilde{\mathcal{A}}_{uv}, h_u^{(l-1)} \middle| u \in \mathcal{N}_v\right\}\right), \quad (S11.1)$$

$$m_v^{(l)} = AGGREGATE_I^{(l)}\left(\{\tilde{\mathcal{A}}_{vu} | u \in \mathcal{N}_v\}\right) h_v^{(l-1)}, \quad (S11.2)$$

$$h_v^{(l)} = COMBINE^{(l)}\left(m_v^{(l)}, m_a^{(l)}\right), \quad (S11.3)$$

where $\mathcal{N}_v$ is the set of 1-hop neighbors of $v$, $\tilde{\mathcal{A}}_{uv} \in \mathbb{R}^K$ denotes a normalized edge feature between $u$ and $v$, $m_a^{(l)}$, $m_v^{(l)}$ gathers structural massage from view of $\mathcal{N}_v$ and $v$ respectively, $COMBINE(\cdot)$ is used to fuse message from two views to update final feature of $v$. Although different GNNs have different aggregation schemes, they can be factorized to above three parts. For example, A usual expression and its decomposition of GIN[7] can be as follows:

$$h_v^{(l)} = MLP^{(l)}\left(\left(1+\epsilon^{(l)}\right)h_v^{(l-1)} + \sum_{u\in\mathcal{N}_v} h_u^{(l-1)}\right), \quad (S11.4)$$

$$m_a^{(l)} := \sum_{u\in\mathcal{N}_v} h_u^{(l-1)}, \quad m_v^{(l)} := \left(1+\epsilon^{(l)}\right)h_v^{(l-1)}, \quad h_v^{(l)} := MLP^{(l)}\left(m_v^{(l)} + m_a^{(l)}\right). \quad (S11.5)$$

Moreover, for graph-level task, a READOUT function is used to aggregate node features $h_{v_i}^{(L)}$ into graph-level representation $h_g$:

$$h_g = READOUT\left(\left\{h_v^{(L)} \middle| v \in \mathcal{G}\right\}\right). \quad (S11.6)$$

It should be noted that, $READOUT(\cdot)$ must be a permutation invariance function, e.g., $MAX(\cdot), MEAN(\cdot), SUM(\cdot)$, etc.

**Transformer.** The Transformer framework is known as specializing in sequence process which can be divided into encoder and decoder layers. Both layers consist of a multi-head attention module (MHA) and a feedforward network (FFN). Given a sentence matrix $S \in \mathbb{R}^{n \times d}$, where $n$ is number of tokens and $d$ is the hidden dimension. The input $S$ is projected by three matrices $W_Q \in \mathbb{R}^{d \times d_Q}, W_K \in \mathbb{R}^{d \times d_K}, W_V \in \mathbb{R}^{d \times d_V}$ respectively. Representations $S^l$ of $l$-th layer can be calculated as:

$$ATTEN^{(l)}\left(S^{(l-1)}\right) = SOFTMAX\left(\frac{S^{(l-1)}W_Q\left(S^{(l-1)}W_K\right)^T}{\sqrt{d_k}}\right)S^{(l-1)}W_V, \tag{S11.7}$$

$$MHA^{(l)}\left(S^{(l-1)}\right) = CONCAT\left(ATTEN_1^{(l)}\left(S^{(l-1)}\right), \dots, ATTEN_{n_{head}}^{(l)}\left(S^{(l-1)}\right)\right)W^O, \tag{S11.8}$$

$$S'^{(l)} = MHA^{(l)}\left(LN\left(S^{(l-1)}\right)\right) + S^{(l-1)}, \qquad S^{(l)} = FFN^{(l)}\left(LN\left(S'^{(l)}\right)\right) + S'^{(l)}, \tag{S11.9}$$

where $W^O \in \mathbb{R}^{n_{head}d_v \times d}$ is a projection matrix which integrates information from different heads and $LN(\cdot)$ is a layer norm function. For sentence level task, we can obtain a global vector $S_g$ by a pooling function on representation from last layer $L$:

$$S_g = POOLING\left(\left\{S_w^{(L)} \middle| w \in S\right\}\right), \tag{S11.10}$$

where $w$ is word token in sentence $S$, $POOLING(\cdot)$ also must be $MAX(\cdot), MEAN(\cdot), SUM(\cdot)$ or any other permutation invariance functions like $READOUT(\cdot)$.

## Supplementary Note 12. A case for the isomer cannot be distinguished by the models without bond encoder

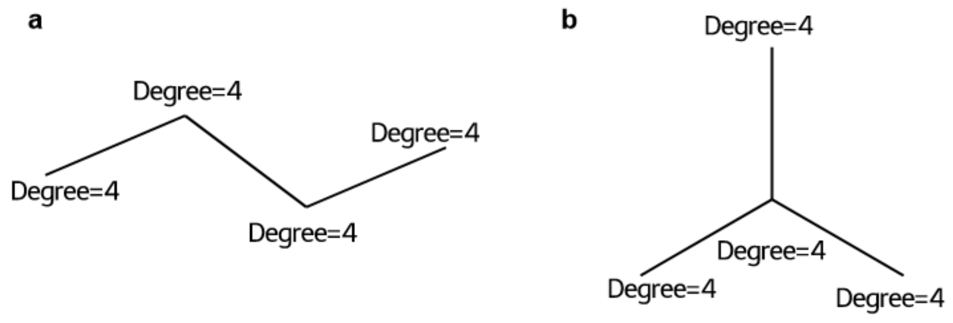


**Supplementary Figure 14. Isomers demonstrating the necessity of bond encoder.** a and b illustrate an isomer pair cannot be distinguished by the models without bond encoder.

## Supplementary Note 13. Comparations on 3D-RetroExplainer and 2D-RetroExplainer

During the model design phase, we consider adding the 3D conformation of input molecular optimized by MMFF[8]. However, given the extra heavy complexity for introducing 3D information, the performance degradation compared with 2D version, and the frequent nonconvergence rate of MMFF, we finally decide to abandon the 3D-RetroExplainer. This is similar to the way that chemists can extrapolate retrosynthesis using only 2D molecules. Results in USPTO-50K are listed in Supplementary Table 4.

The degradation of performance mostly is mostly because following reasons: 1) The 3D conformation calculated by MMFF is not stable enough, leading to the perplexity for RetroExplainer to understand. Maybe a larger parameter capacity for RetroExplainer is needed. 2) For the most conditions, retrosynthesis task is not relevant to too detailed 3D information. Only given the 2D structural formular of product, an experienced Chemist also can plan the retrosynthetic pathway. Even for asymmetric chemistry, some simple descriptor of bond is just enough.

**Supplementary Table 4. Results of 2D/3D-RetroExplainer in USPTO-50K datasets.**

| Model\Metrics | | Top-k Accuracy (%) | | | |
|---|---|---|---|---|---|
| | | k=1 | k=3 | k=5 | k=10 |
| Reaction Type Known | 2D-RetroExplainer | 66.8 | 88.0 | 92.5 | 95.8 |
| | 3D-RetroExplainer | 60.6 | 80.7 | 87.8 | 93.9 |

| | | | | | |
|---|---|---|---|---|---|
| Reaction Type Unknown | 2D-RetroExplainer | 57.7 | 79.2 | 84.8 | 91.4 |
| | 3D-RetroExplainer | 52.4 | 75.9 | 82.4 | 89.6 |

Note that the configurations of 3D-RetroExplainer are kept same as 2D-RetroExplainer.

## Supplementary Note 14. About dynamic adaptive multi-task learning strategy (DAMT)

In the RetroExplainer training phase, we encounter five losses needed to be optimized parallelly: leaving group matching loss, reaction centre prediction loss, leaving group connecting loss, hydrogen change loss, and contrastive learning loss, which is different from the previous work[9-11] that contains two simple loss that need to be optimized serially. Optimizing parallelly five losses are difficult to balance the final performance using the traditional gradient descent algorithm, due to different magnitudes of gradients for those losses, which are usually encountered in computer vision domain. Previous works for joint learning strategies are not suitable for the conditions we encountered. For instance, GradNrom[12] needs to maintain a large computational graph which only applies to simple multi-layer perceptron. In the manuscript, we propose an effective self-adaptive learning module to optimize the above five losses parallelly to equally focus on each task shown in Supplementary Table 3. Thus, the proposed framework is also potential to be applied in other multi-objective optimization scenarios. The algorithm of DAMT can be referred as follows:

---

**Supplementary Box 3: The parameter update process using DAMT**

---

**Input:** Reaction graph pair $\{\mathcal{G}_p, \mathcal{G}_r\}$, RetroExplainer model $f_{\theta^0}(\cdot)$, the size of the queue $n$, max epochs $n_p$ of training stage Temperature $\tau$;

**Output:** A trained RetroExplainer model $f_{\theta^*}(\cdot)$;

$T: \{t_i\}_{i=1}^{K_t} \leftarrow Get\ lables\ through\ AtomAlignment\ (\mathcal{G}_p, \mathcal{G}_r)\ for\ K_t\ tasks$;

$Q \leftarrow Initializing\ the\ queue$;

$\boldsymbol{For}\ t\ epoch\ \boldsymbol{in}\ [1, \ldots, n_p]$:

    $\boldsymbol{For}\ batched\ graph\ set\ \{\mathcal{G}_{p1}, \mathcal{G}_{p2}, \ldots\}\ in\ total\ dataset\ \mathcal{D}_{train}$:

**Supplementary Box 3: The parameter update process using DAMT**

$T_{pred} \leftarrow f_{\theta^{t-1}}(\{\mathcal{G}_{p1}, \mathcal{G}_{p2}, \dots\})$;

$Get\ vector\ \boldsymbol{\mathcal{L}}_T^{(t)}: \left\{\mathcal{L}_i^{(t)}\right\}_{i=1}^{K_t} \leftarrow Criterion(T, T_{pred})$;

$\boldsymbol{If}\ Size(Q) < 2$:

$Get\ scalar: \mathcal{L}_T^{(t)} \leftarrow \sum_{i=1}^{K_t} \mathcal{L}_i^{(t)}$, $\boldsymbol{Continue}$;

$\boldsymbol{end\ If}$;

$Decent\ Rates: \left\{r_i^{(t)} \leftarrow \frac{\mathcal{L}_i^{(t-1)}}{\mathcal{L}_i^{(t-2)}}\right\}_{i=1}^{K_t}$; $\boldsymbol{\mathcal{L}}_T^{(t-1)}, \boldsymbol{\mathcal{L}}_T^{(t-2)} \leftarrow Get(Q)$;

$n' \leftarrow \min(n, size(Q))$;

$\boldsymbol{\mathcal{L}}_T^{(t-1)}, \dots, \boldsymbol{\mathcal{L}}_T^{(t-n')} \leftarrow Get(Q)$;

$Nomalizing\ Coefficients \left\{\alpha_i^t = \frac{n'}{\sum_{j=t-1}^{t-n'} \mathcal{L}_i^{(j)}}\right\}_{i=1}^{K_t}$;

$Get\ scalar: \mathcal{L}_T^{(t)} \leftarrow \sum_{i=1}^{K_t} \left(softmax\left(\frac{r_i^{(t)}}{\tau}\right) \alpha_i^t \mathcal{L}_i^{(t)}\right)$;

$\theta^t \leftarrow Optimize\left(\theta^{t-1}, \mathcal{L}_T^{(t)}\right)$;

$Enqueue\left(Q \leftarrow \boldsymbol{\mathcal{L}}_T^{(t)}\right)$;

$\boldsymbol{If}\ size(Q) > n$:

$Dequeue\left(Q \rightarrow \boldsymbol{\mathcal{L}}_T^{(t-n)}\right)$;

$\boldsymbol{end\ If}$;

**end For**;

**end For**;

$\theta^* \leftarrow \theta^{n_p}$;

**return** $f_{\theta^*}(\cdot)$;

**Supplementary Table 5. The original loss cures in training phase.**

| Task | LGM (averaged by contrastive learning loss) | RCP | LGC | HC |
|---|---|---|---|---|
| Loss Curve | 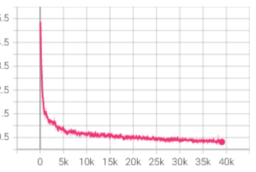 | 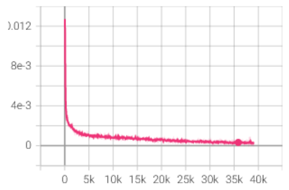 | 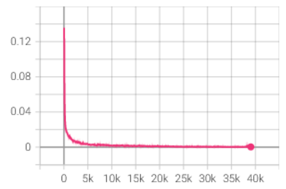 | 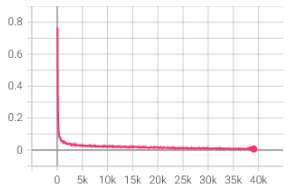 |
| Range | [2.768e-1, 5.359] | [1.89e-4, 1.2e-2] | [6.38e-5, 1.35e-1] | [4.97e-3, 7.67e-1] |

## Supplementary Note 15. Atom-perturbation-based explainability (APEx) and reaction type tracing analysis

To explore the substructure impact of the input molecule and the RetroExplainer's comprehension of each task, we employ the APEx explainability algorithm outlined below. In broad terms, we document the initial loss of the given sample, mask atom and edge features, compute loss value change rates, and ultimately derive the contribution graph using these rates. It's important to note that the score (applicable to atoms or edges) within the contribution graph directly signifies how the loss will increase relative to its initial value when the corresponding substructure is masked. To enhance the clarity of the contribution graph, we extract multiple tasks for visualization, as depicted in Supplementary Figure 15a. The results indicate that the ester amide serves as a pivotal functional group for the RCP task, while the ester group emerges as the most significant substructure for the LGM task. For a holistic prediction, we judiciously consider the scale and range of loss values across the four subtasks. Ultimately, the ester amide emerges as the critical functional group in the sampled reaction.

In an extension of our approach, we integrate APEx with reaction type mutation experiments. In detail, given a product molecule labeled with a reaction type, APEx can yield a contribution aligned with the input reaction type. Utilizing this process, we keep the product molecule fixed while sequentially altering the reaction type label, inputting it into APEx each time. This provides a contribution vector whose elements delineate sensitivity to the focused atom for each mutated reaction type. As displayed in Supplementary Figure 15b, we present both a rigid label version, focusing solely on the mutated reaction type the substructure primarily concerns, and a flexible label version that combines all mutated reaction types using the contribution vector. Both versions can

indicate the likely source reaction type for the focused substructure within a specific task. For example, in the RCP task with the hard label version, the hydroxyl group is linked to reaction type five (deprotections), implying that RCP considers deprotections to be the most probable method for synthesizing the hydroxyl group. In the LGM task, the hydroxyl group is more likely synthesized via reaction type 8 (functional group interconversion). Since ignoring suboptimal solutions of other reaction types when contribution scores for different types are closely ranked isn't prudent, the soft label version is indispensable. In these soft label versions, the substructure's color is calculated via a weighted sum based on ten anchor colors and the contribution scores. Overall predictions using the soft label version in Supplementary Figure 15b indicate that RetroExplainer prioritizes heterocyclic sulfur and underscores amine structures in synthesizing the sampled product

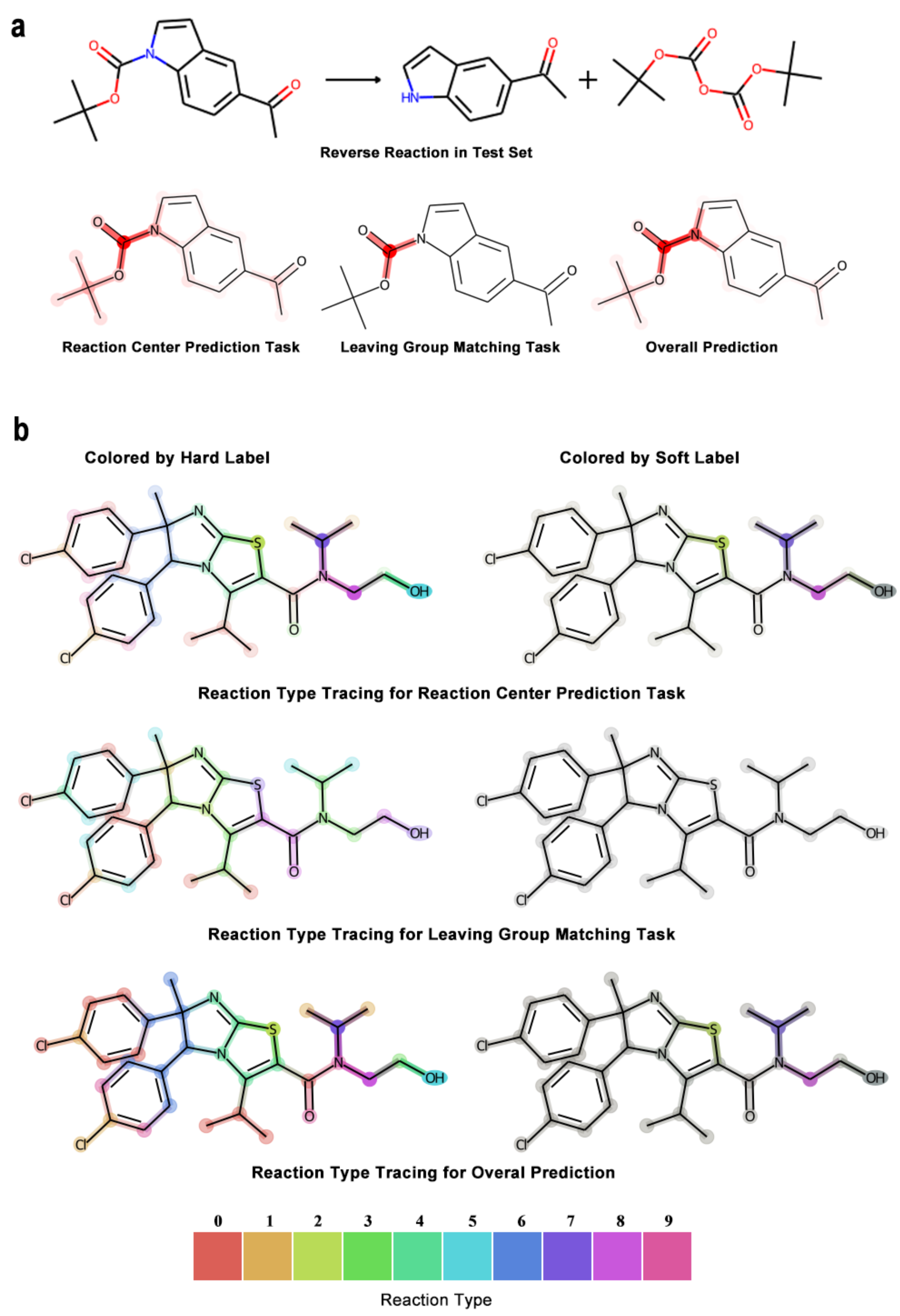


.

**Supplementary Figure 15. Reaction type mutations. a. Within the test set, the figure presents the sampled reverse reaction alongside the contribution graph extracted via the atom-perturbation-based explainability algorithm.** From this contribution graph, RetroExplainer highlights the significance of ester amide, ester, and ester amide as critical substructures corresponding to the RCP, LGM, and Overall prediction tasks, respectively. **b. The outcomes of reaction type tracing for the three extracted tasks are depicted in this part.** With regard to hard labels, the molecule is tinted using anchor colors, indicative of the top-1 reaction type in the contribution vector for each atom. Conversely, employing soft labels results in a blended color composition. This mixed hue is determined by the weighted and aggregated RGB values derived from the contribution vector. The intensity of the substructure's color corresponds to its contribution to the corresponding inverse reaction.

**Supplementary Box 4: Atom Perturbation-based Explainability**

**Input:** Reaction graph pair $\{\mathcal{G}_p, \mathcal{G}_r\}$, well-trained RetroExplainer model $f_{\theta^*}(\cdot)$;

**Output:** contribution graph set $\{\mathcal{G}_c^i = (X_c^i \in \mathbb{R}^N, A_c^i \in \mathbb{R}^{N\times N})\}_{i=1}^{K_t}$ for task set $\mathcal{T} = \{t_i\}_{i=1}^{K_t}$;

$\mathcal{L}^0 \leftarrow \{\ell_i^0\}_{i=1}^{K_t}, \ell_i^0 \leftarrow criterion_i(f_{\theta^*}(\mathcal{G}_p), \mathcal{G}_r);$

$\boldsymbol{For}\ atom\ \boldsymbol{in}\ \mathcal{G}_p$:

$\quad \tilde{X}_p \leftarrow X_p, \tilde{X}_p[atom] \leftarrow 0;$

$\quad \tilde{A}_p \leftarrow A_p, \tilde{A}_p[:, atom] \leftarrow 0, \tilde{A}_p[atom, :] \leftarrow 0;$

$\quad \mathcal{L}^{atom} \leftarrow \{\ell_i^{atom}\}_{i=1}^{K_t}, \ell_i^{atom} \leftarrow \frac{criterion_i(f_{\theta^*}(\tilde{G}_p), \mathcal{G}_r)}{\ell_i^0} - 1;$

**end For**;

$\{\mathcal{G}_c^i \leftarrow (X_c^i, A_c^i)\}_{i=1}^{K_t}, X_c^i \leftarrow normalize([\ell_i^1, \ldots, \ell_i^N]); A_c^i \leftarrow expand(X_c^i);$

**return** $\{\mathcal{G}_c^i\}_{i=1}^{K_t}$

## Supplementary Note 16. Failure cases of RetroExplainer trained on USPTO-50K dataset

To show the limitations of RetroExplainer, we task some failure cases for example as shown in Supplementary Table 6 and Supplementary Table 7.

Case A: RetroExplainer focus on unmatched reaction center and thus generates unmatched leaving groups. This is mainly because RetroExplainer predicts the wrong number of reaction centers (the ground truth should be 0), which most probably caused by some similar structures that have none-zero reaction centers in the used dataset. Thus, RetroExplainer is confused when predicting reaction centers. Notice that this doesn't mean RetroExplainer cannot constitutionally predict none-reaction-center reactions. If the all the element in the predicted reactivity matrix tend to zero (or lower than pre-set threshold), RetroExplainer will skip the reaction center selection stage (replace bond type action or remove bond action).

Case B: Though predicting correct reaction center, RetroExplainer cannot generate correct leaving groups. The correct answer should be Chlorine substituent but are oxhydryl group, ketone group and Bromide substituent in the top-3 predictions of RetroExplainer.

Case C: To show the diversity reaction type that RetroExplainer predicts, we adopt top-1(oxidation rather than reduction), top-8(C-O coupling) and top-10 (ring formatting) answers in this case.

Case D: RetroExplainer is in capable of predict well for ring-forming reactions. The top-1 prediction can exactly match the ground truth. The decision steps of the correct prediction show the whole

process: RetroExplainer generate ketone group (the index of leaving group is 2) numbered as 29 and then directly connect the atom 29 with atom 11 (a carbon atom with double bond). After that, RetroExplainer cut off the double bond between atom 11 and atom 10 then finally add two hydrogens to atom 10 to follow the valency rule. For the top-2,3 results, RetroExplainer predicts unmatched reaction centers similar as Case A, generating oxhydryl group and methoxy group as leaving groups, respectively.

Case E: A failure case of ring-forming reactions predicted by RetroExplainer. The three generated reactants are potential to transform into target product but they are highly unstable in practice. The decision steps show the generated process of the top-2 prediction (most similar to the ground truth).

**Supplementary Table 6. Failure cases of RetroExplainer.**

| | | | |
|---|---|---|---|
| Case A | | | |
| Reaction | 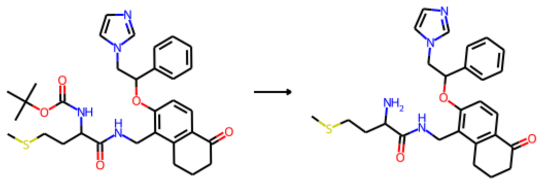 | | |
| Prediction | 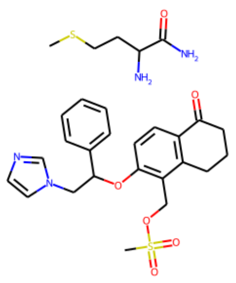 | 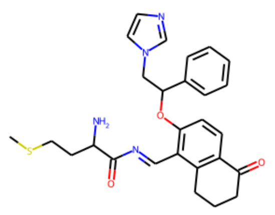 | 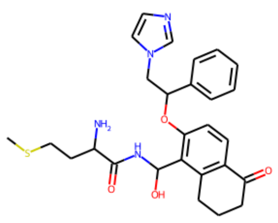 |
| Energy | 7.79 | 10.72 | 11.10 |
| Type | Wrong Leaving Groups and Wrong Reaction Centers. | | |
| Case B | | | |
| Reaction | 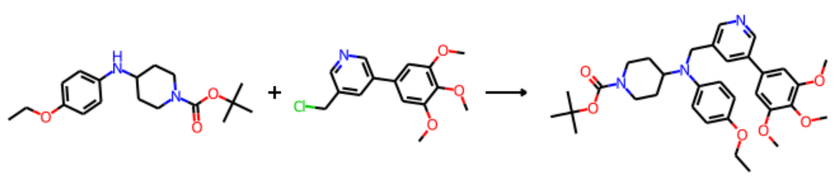 | | |

| | | | |
|---|---|---|---|
| Prediction | 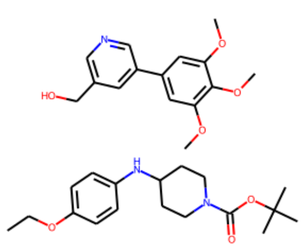 | 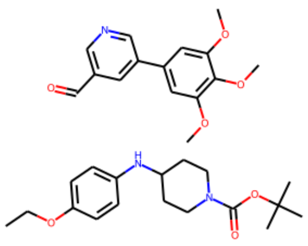 | 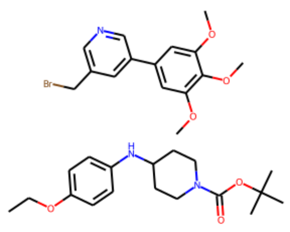 |
| Energy | 8.41 | 13.77 | 14.34 |
| Type | Wrong Leaving Groups and True Reaction Centers | | |
| Case C | | | |
| Reaction | 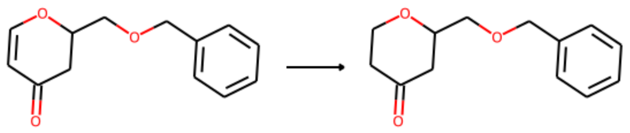 | | |
| Prediction | 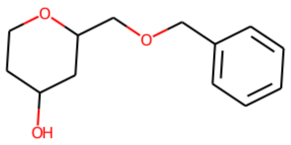 | 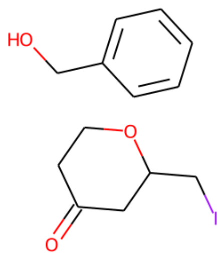 | 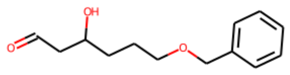 |
| Energy | 19.0 | 25.56 (top-8) | 27.42 (top-10) |
| Type | Wrong Leaving Groups and Wrong Reaction Centers | | |

**Supplementary Table 7. Ring-opening and ring-forming reactions cases.**

| | | | |
|---|---|---|---|
| Case D | | | |
| Reaction | 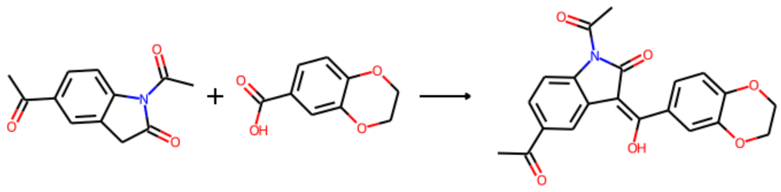 | | |
| Prediction | 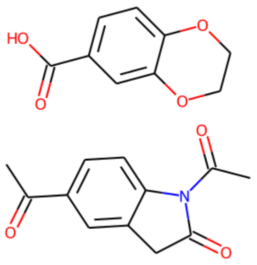 | 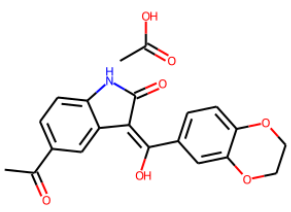 | 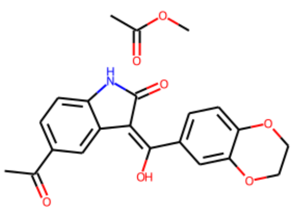 |
| Energy | 11.50 | 12.79 | 14.76 |
| | 1. Select Leaving Group with Index 2 and Cost 2.42, | | Atom Indices of Product |

| | | | |
|---|---|---|---|
| Decision Steps of The Correct Prediction | 2. Initial Cost:12.61,<br>3. Add Bonds: between 11 and 29 with Bond Type 2.0 and Cost -0.10,<br>4. Remove Bonds: between 10 and 11, with Bond Type 2.0 and Cost -1.02,<br>5. H number change 2 cost of atom 10: 0.00 | | 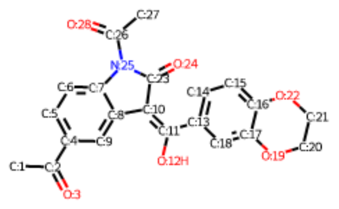 |
| Case E | | | |
| Reaction | 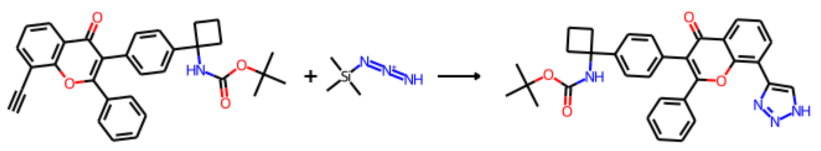 | | |
| Prediction | 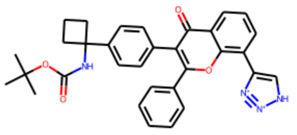 | 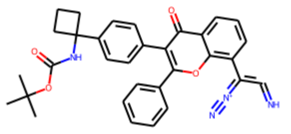 | 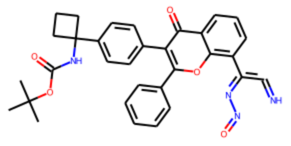 |
| Energy | 10.77 | 11.86 | 13.85 |
| Decision Steps of the Top-2 Prediction | 1. Select Leaving Group with Index 6 and Cost 0.44,<br>2. Initial Cost:14.13,<br>3. Remove Bonds: between 27 and 28, with Bond Type 1.5 and Cost -1.81,<br>4. Replace Bonds: between 28 and 29, from Bond Type 1.5 to Bond Type 3 with Cost -1.56,<br>5. H number change 1 cost of atom 27: 1.09. | | Atom Indices of Product<br>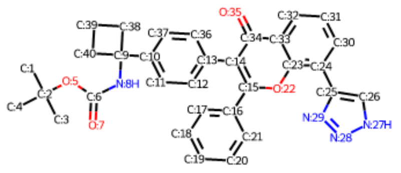 |

## Supplementary Note 17. Reaction type mutation experiment and directed retrosynthesis prediction

As mentioned earlier, reaction type embeddings enhance prediction performance by imposing constraints on the features of hidden layers. However, this profound prior knowledge can also prove to be a significant obstacle when attempting predictions for target reaction types that deviate from the training set distribution. To mitigate this limitation and to showcase the generalizability of RetroExplainer, we employ a random sampling approach on the test set. We carefully select cases that match the criteria of different reaction types (e.g., heterocycle for a reaction type involving heterocycle formation). Subsequently, we alter the reaction type of the selected sample to various other types and feed it into RetroExplainer for predictions.

As depicted in Supplementary Figure 16, the predicted reactants that lie beyond the dataset's domain distribution align well with the corresponding reaction types. This illustrates that our RetroExplainer comprehends various reaction types effectively and exhibits good generalization. Simultaneously, from a practical standpoint, by providing the directed reaction type and product molecule as input, RetroExplainer can predict robust and targeted reactants that cater to diverse user requirements.

Furthermore, the embeddings of reaction types that we have learned hold potential for other related tasks such as encoding reactions and inferring organic mechanisms, thereby extending the utility of our approach beyond the current scope.

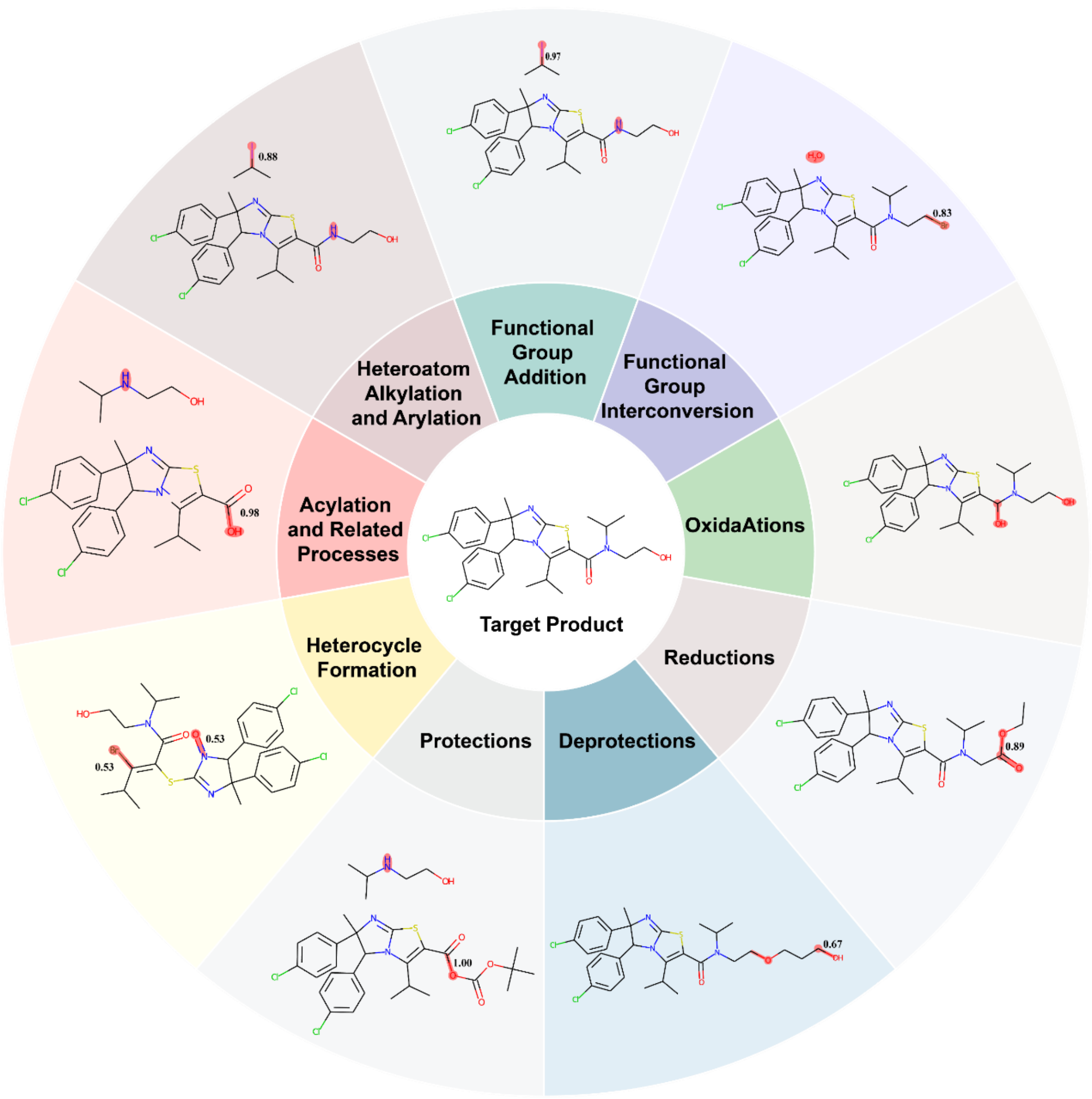


**Supplementary Figure 16. Reaction type mutation experiment.** A product randomly selected from the test set is combined with various reaction type embeddings by altering their indices and subsequently fed into RetroExplainer. Despite the fact that the majority of these instances do not adhere to the distribution pattern of the original cases, RetroExplainer continues to exhibit strong performance, yielding predictions that align with the associated reaction types consistently.

## Supplementary references


1 Mayer, C. D., Lorent, J. & Horgan, G. W. Exploratory analysis of multiple omics datasets using the adjusted RV coefficient. *Stat Appl Genet Mol Biol* **10**, Article 14, doi:10.2202/1544-6115.1540 (2011).

2 Chen, B., Li, C., Dai, H. & Song, L. in *International Conference on Machine Learning (ICML).* (2020).

3 <https://downloads.emolecules.com/free/2023-04-01/> (

4 Thomas, N. K. & Max, W. in *International Conference on Learning Representations* (2017).

5 Hamilton, W., Ying, Z. & Leskovec, J. in *Advances in Neural Information Processing Systems* Vol. 30 (eds I. Guyon *et al.*) (2017).

6 Veličković, P. *et al.* in *International Conference on Learning Representations.*

7 Keyulu, X., Weihua, H., Jure, L. & Stefanie, J. in *International Conference on Learning Representations* (2019).

8 Halgren, T. A. Merck molecular force field. I. Basis, form, scope, parameterization, and performance of MMFF94. *Journal of Computational Chemistry* **17**, 490-519, doi:https://doi.org/10.1002/(SICI)1096-987X(199604)17:5/6<490::AID-JCC1>3.0.CO;2-P (1996).

9 Shi, C., Xu, M., Guo, H., Zhang, M. & Tang, J. in *Proceedings of the 37th International Conference on Machine Learning* Article 818 (JMLR.org, 2020).

10 Yan, C. *et al.* in *Advances in Neural Information Processing Systems.* (eds H. Larochelle *et al.*) 11248-11258 (Curran Associates, Inc.).

11 Somnath, V. R., Bunne, C., Coley, C., Krause, A. & Barzilay, R. in *Advances in Neural Information Processing Systems.* (eds M. Ranzato *et al.*) 9405-9415 (Curran Associates, Inc.).

12 Chen, Z., Badrinarayanan, V., Lee, C.-Y. & Rabinovich, A. GradNorm: Gradient Normalization for Adaptive Loss Balancing in Deep Multitask Networks. (2017).
