# Supplementary material for: Retrosynthesis prediction with an interpretable deep-learning framework based on molecular assembly tasks
Source: Nat Commun. 2023 Oct 3;14:6155. doi: 10.1038/s41467-023-41698-5 (PMC10547708; doi:10.1038/s41467-023-41698-5)
Supplement: Supplementary file 3 — Description of Additional Supplementary Files [file 41467_2023_41698_MOESM3_ESM.pdf]

<u>Description of Additional Supplementary Files</u>

Supplementary Data 1

Description: Multi-step planning results using RetroExplainer containing 101 synthetic routes. File Name:

Supplementary Data 2

Description: Findings from literature searches based on 101 synthetic routes.
