# Supplementary material for: Retrosynthesis prediction with an interpretable deep-learning framework based on molecular assembly tasks
Source: Nat Commun. 2023 Oct 3;14:6155. doi: 10.1038/s41467-023-41698-5 (PMC10547708; doi:10.1038/s41467-023-41698-5)
Supplement: Supplementary file 4 — Supplementary Data 1 [file 41467_2023_41698_MOESM4_ESM.pdf]

Route 1<br>Step 1	Null	
Step 1	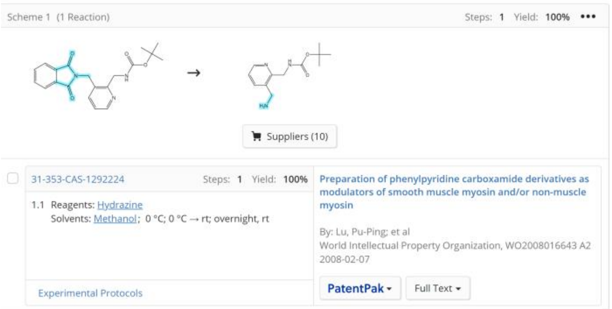	https://scifinder-n.cas.org/search/reaction/63f331b3692896544bfa16f2/1
Step 2	Null	
Route 2<br>Step 1	Null	
Step 3	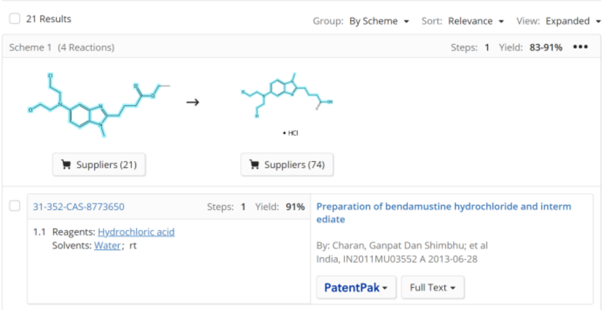	https://scifinder-n.cas.org/search/reaction/63f35e2a692896544bfdb0b4/1
Route 3<br>Step 1	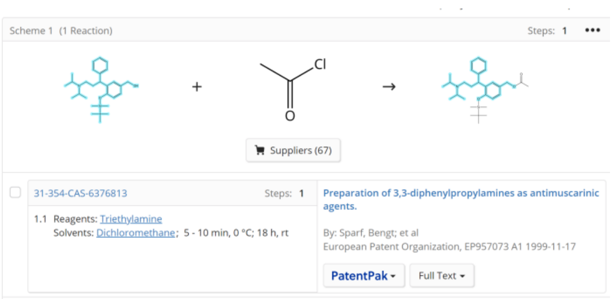	https://scifinder-n.cas.org/search/reaction/63f35eaa692896544bfdba94/1
Route 4<br>Step 1	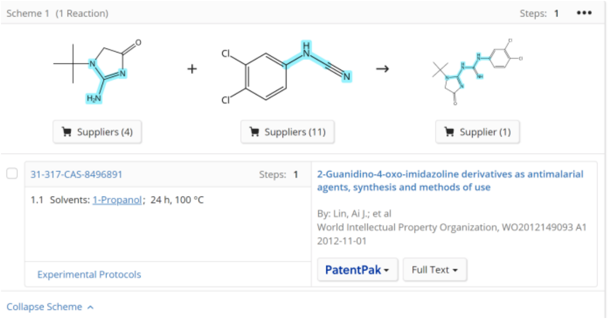	https://scifinder-n.cas.org/search/reaction/63f35f17692896544bfdc1e8/1

Route 5<br>Step 1	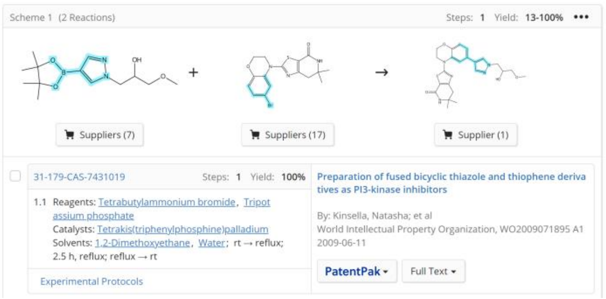	https://scifinder-n.cas.org/search/reaction/63f35fa9692896544bfdc9e4/1
Step 2	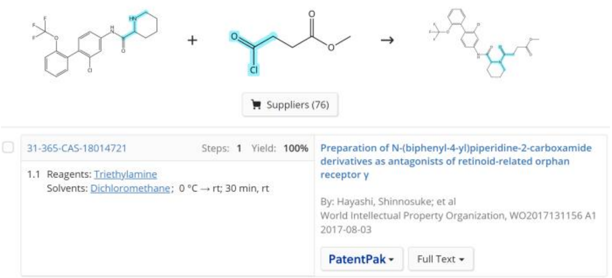	https://scifinder-n.cas.org/search/reaction/63f3600e692896544bfdcf6c/1
Step 3	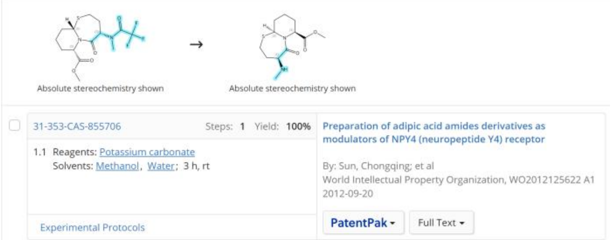	https://scifinder-n.cas.org/search/reaction/63f36073692896544bfdd49b/1
Step 4	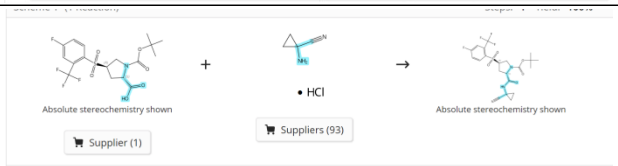	https://scifinder-n.cas.org/search/reaction/63f360bb692896544bfdd815/1
Step 5	Null	
Route 6<br>Step 1	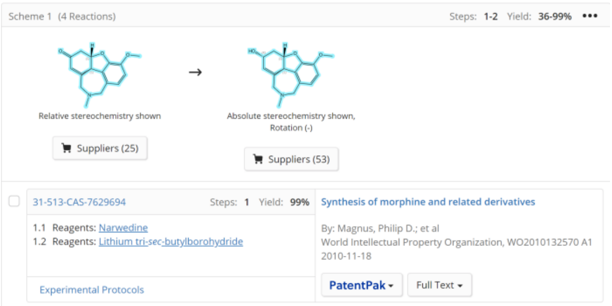	https://scifinder-n.cas.org/search/reaction/63f36201692896544bfdeb3c/1
Route 7<br>Step 1	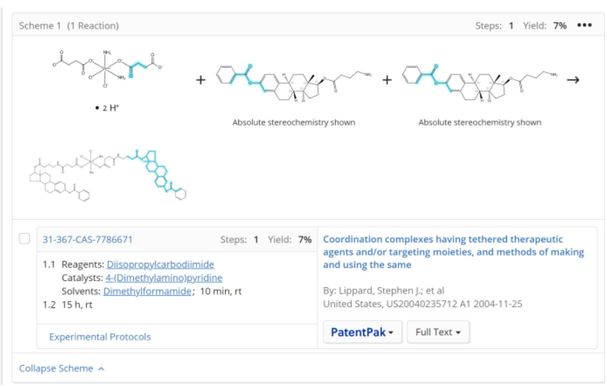	https://scifinder-n.cas.org/search/reaction/63f36249692896544bfdef43/1

<table>
<tr><td>Route 8
Step 1</td><td>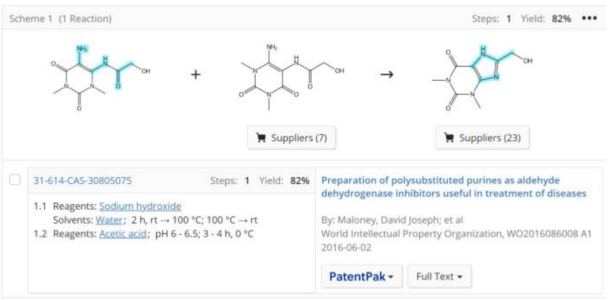
</td><td>https://scifinder-n.cas.org/search/reaction/63f36325692896544bfdfc71/1</td></tr>
<tr><td>Step 2</td><td>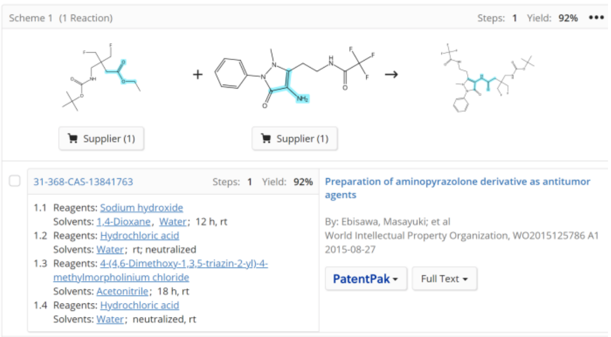
</td><td>https://scifinder-n.cas.org/search/reaction/63f363e4692896544bfe0871/1</td></tr>
<tr><td>Step 3</td><td>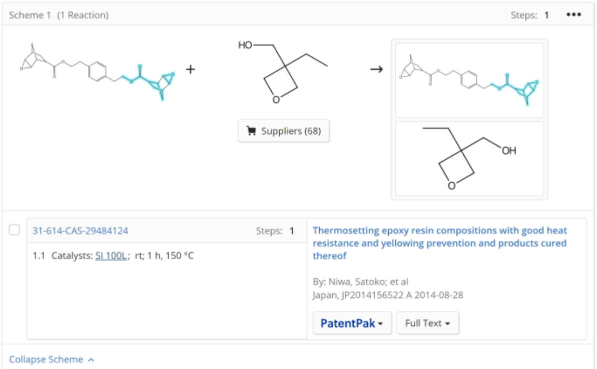
</td><td>https://scifinder-n.cas.org/search/reaction/63f364c6692896544bfe1457/1</td></tr>
<tr><td>Route 9
Step 1</td><td>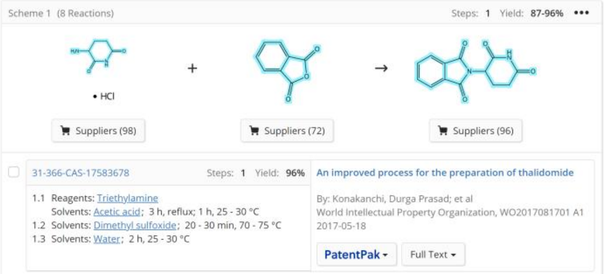
</td><td>https://scifinder-n.cas.org/search/reaction/63f36567692896544bfe1c68/1</td></tr>
<tr><td>Route 10
Step 1</td><td>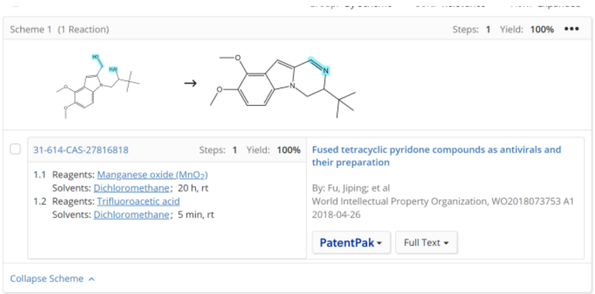
</td><td>https://scifinder-n.cas.org/search/reaction/63f365a4692896544bfe1fbf/1</td></tr>
<tr><td>Step 2</td><td>Null</td><td></td></tr>
</table>

<table>
<tr><td>Route 11
Step 1</td><td>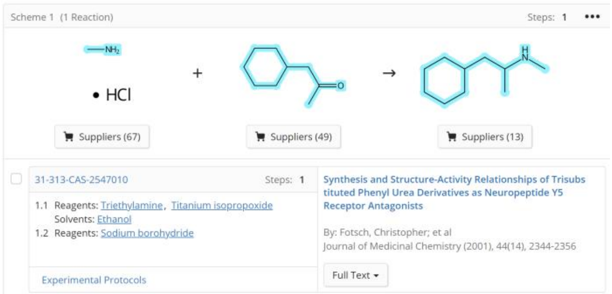
</td><td>https://scifinder-n.cas.org/search/reaction/63f36635692896544bfe27fc/1</td></tr>
<tr><td>Route 12
Step 1</td><td>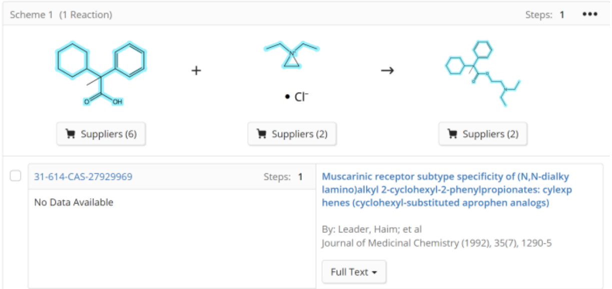
</td><td>https://scifinder-n.cas.org/search/reaction/63f36c01692896544bfe8555/1</td></tr>
<tr><td>Route 13
Step 1</td><td>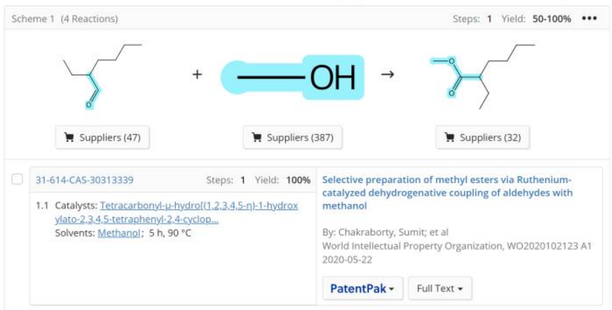
</td><td>https://scifinder-n.cas.org/search/reaction/63f36c7e692896544bfe8e12/1</td></tr>
<tr><td>Step 2</td><td>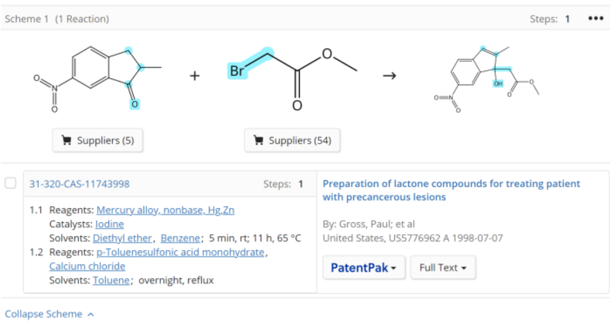
</td><td>https://scifinder-n.cas.org/search/reaction/63f36d10692896544bfe98f1/1</td></tr>
<tr><td>Route 14
Step 1</td><td>Null</td><td></td></tr>
<tr><td>Step 2</td><td>Null</td><td></td></tr>
<tr><td>Route 15
Step 1</td><td>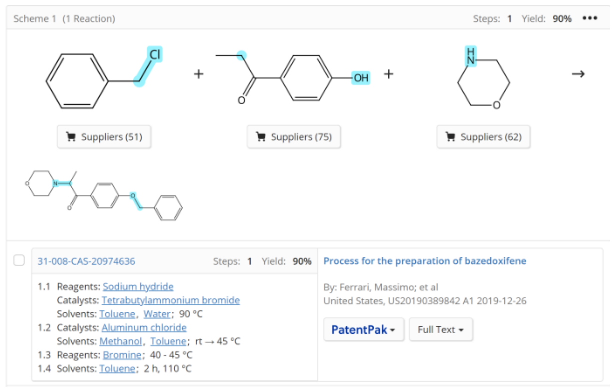
</td><td>https://scifinder-n.cas.org/search/reaction/63f36f75692896544bfec35e/1</td></tr>
</table>

Route 16<br>Step 1	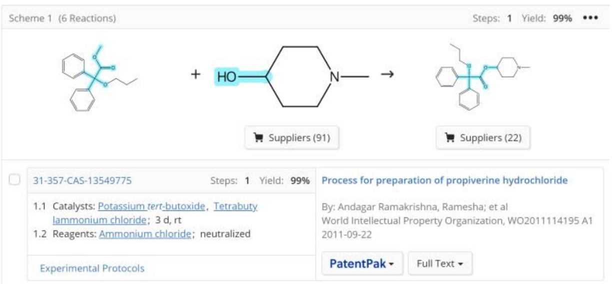	https://scifinder-n.cas.org/search/reaction/63f36fba692896544bfec6d2/1
Route 17<br>Step 1	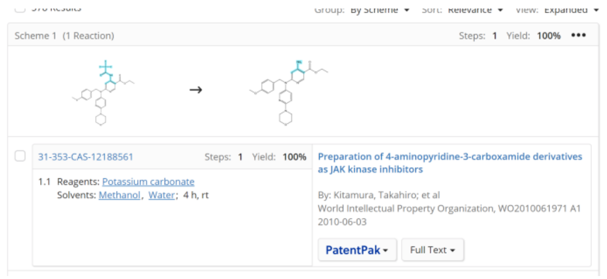	https://scifinder-n.cas.org/search/reaction/63f36ff6692896544bfeca65/1
Step 2	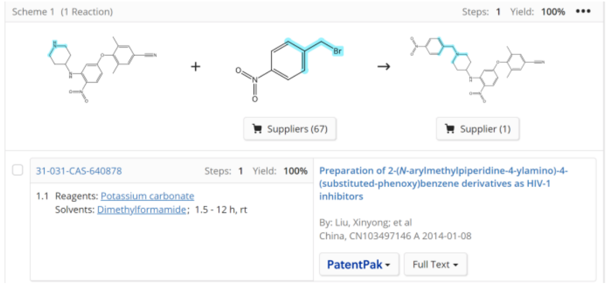	https://scifinder-n.cas.org/search/reaction/63f3706169289654 4bfed0b1/1
Step 3	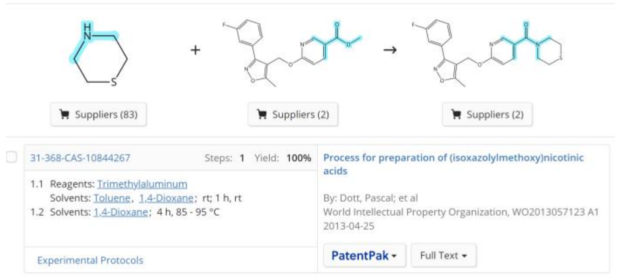	https://scifinder-n.cas.org/search/reaction/63f3709869289654 4bfed467/1
Route 18<br>Step 1	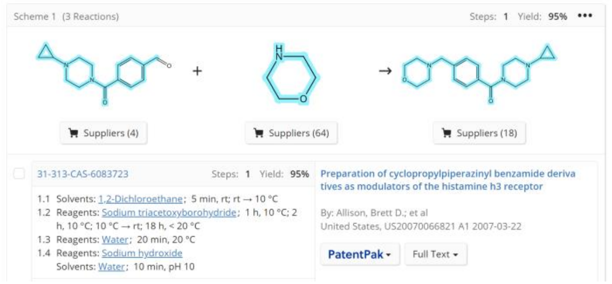	https://scifinder-n.cas.org/search/reaction/640597eb3136c60ac9283a8b/1
Route 19<br>Step 1	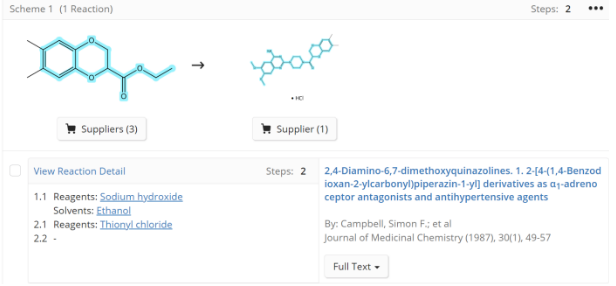	https://scifinder-n.cas.org/search/reaction/640598213136c60ac9283fe2/1
Route 20<br>Step 1	Null	

Step 2	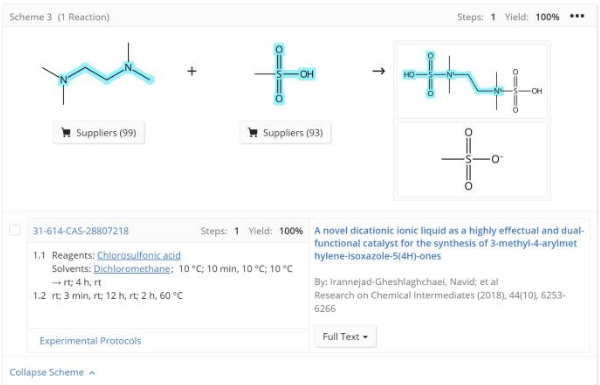	https://scifinder-n.cas.org/search/reaction/640598b83136c60ac9284d70/1
Route 21<br>Step 1	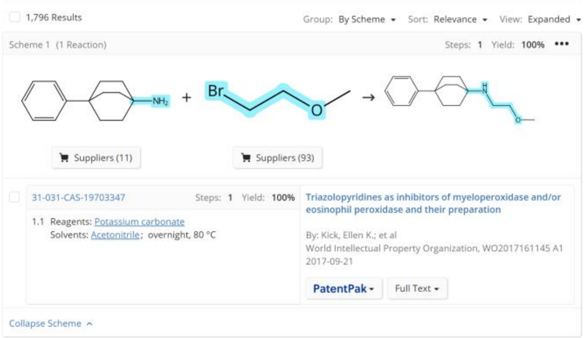	https://scifinder-n.cas.org/search/reaction/64059d4d0e98827b63fa2cf1/1
Step 2	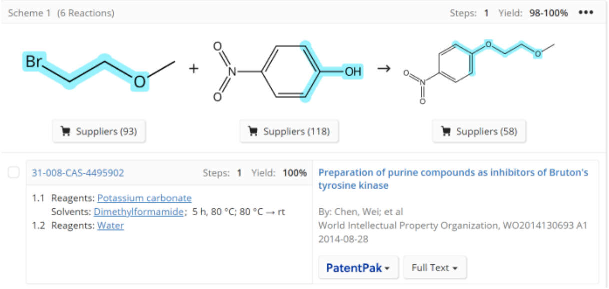	https://scifinder-n.cas.org/search/reaction/64059fea0e98827b63fa6fe0/1
Route 22<br>Step 1	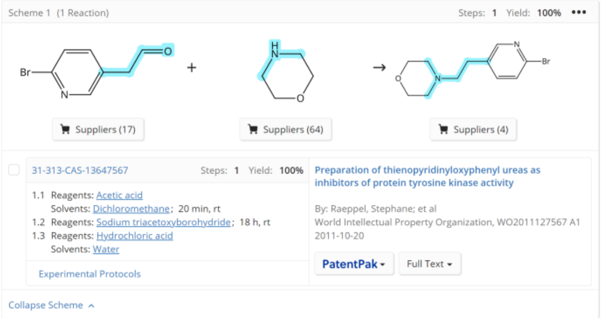	https://scifinder-n.cas.org/search/reaction/6405a59f0e98827b63fb1461/1
Step 2	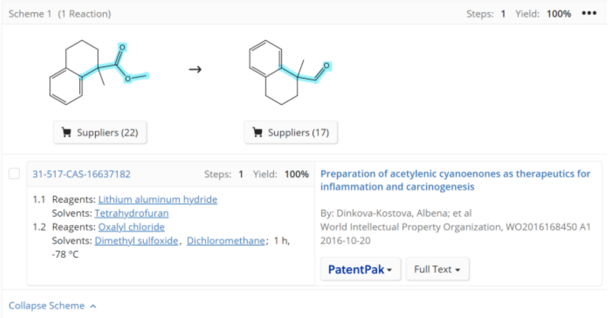	https://scifinder-n.cas.org/search/reaction/6405b00a0e98827b63fc33b1/1

Step 3	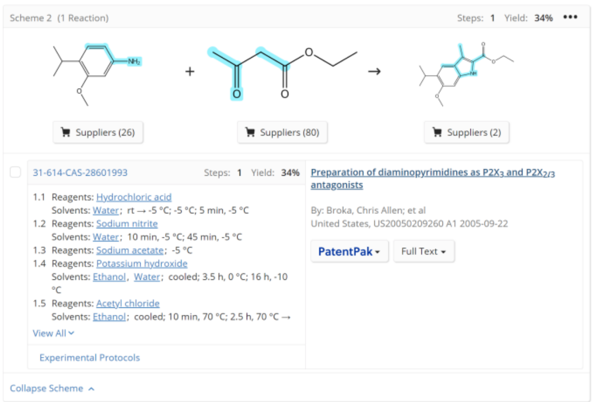	https://scifinder-n.cas.org/search/reaction/6405b1140e98827b63fc4e86/1
Route 23<br>Step 1	Null	
Step 2	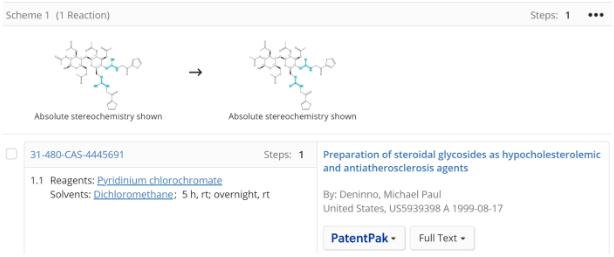	https://scifinder-n.cas.org/search/reaction/6405b1fd0e98827b63fc643c/1
Step 3	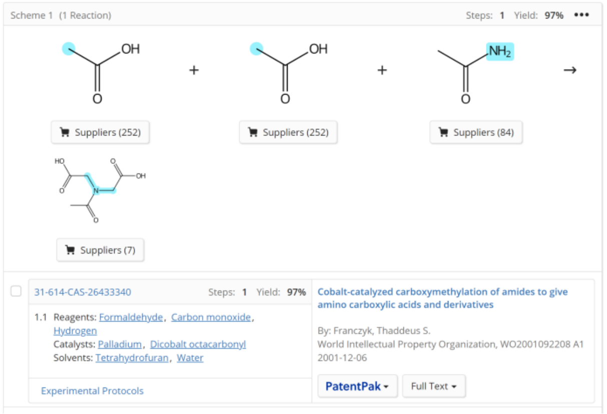	https://scifinder-n.cas.org/search/reaction/6405b2bc0e98827b63fc7732/1
Route 24<br>Step 1	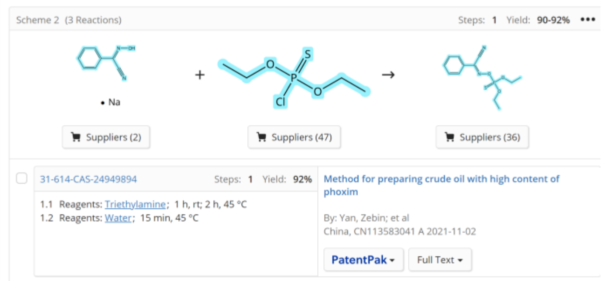	https://scifinder-n.cas.org/search/reaction/6405c29f0e98827b63fdc97d/1

Route 25<br>Step 1	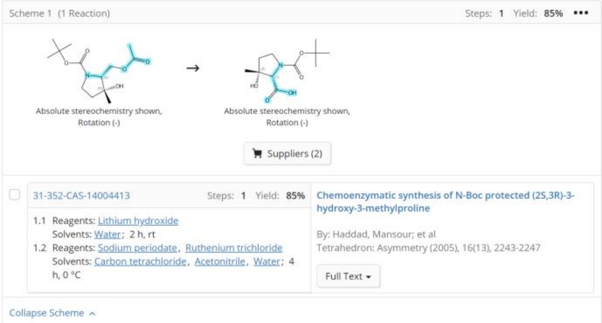	https://scifinder-n.cas.org/search/reaction/6405cac60e98827b63fe6b4a/1
Step 2	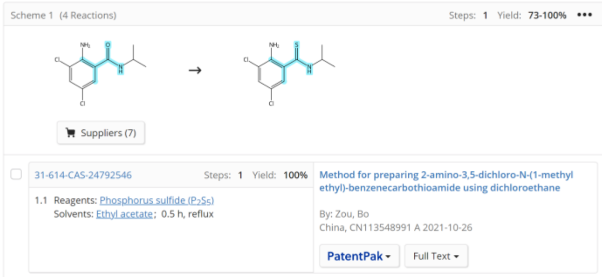	https://scifinder-n.cas.org/search/reaction/6405cc2e0e98827b63fe8a5a/1
Step 3	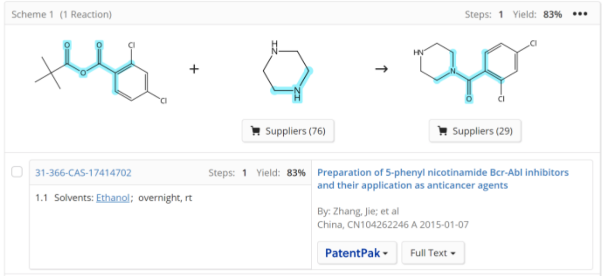	https://scifinder-n.cas.org/search/reaction/6405cca20e98827b63fe939b/1
Step 4	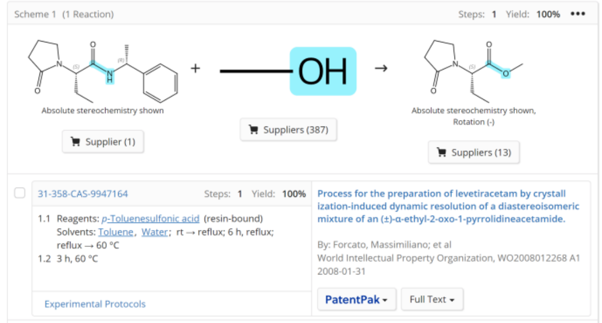	https://scifinder-n.cas.org/search/reaction/6405cccf0e98827b63fe97a3/1
Route 26<br>Step 1	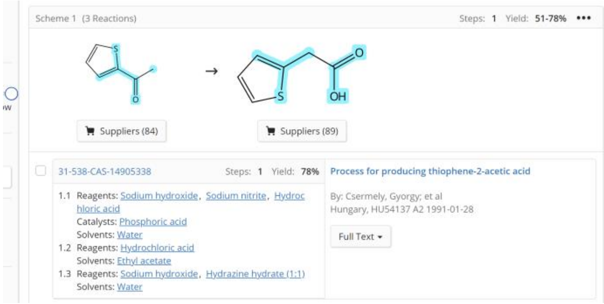	https://scifinder-n.cas.org/search/reaction/6405ced80e98827b63fec47e/1

Route 27<br>Step 1	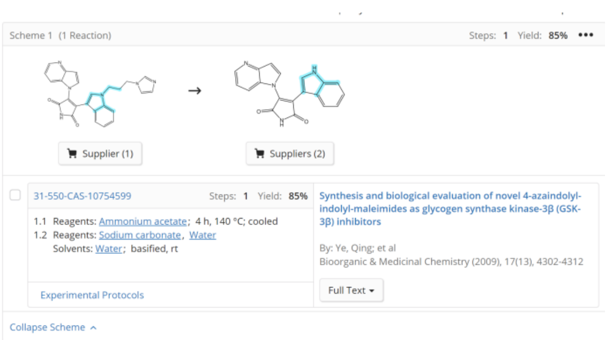	https://scifinder-n.cas.org/search/reaction/6405cf3e0e98827b63fecf5c/1
Step 2	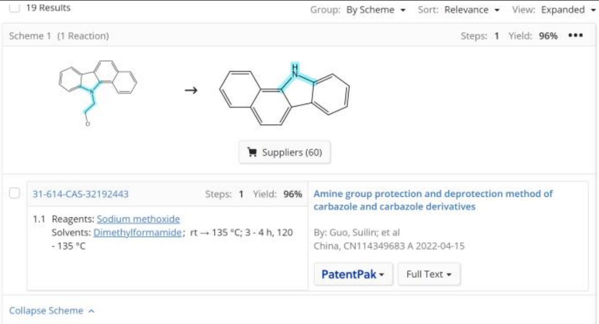	https://scifinder-n.cas.org/search/reaction/6405cf740e98827b63fed3b6/1
Route 28<br>Step 1	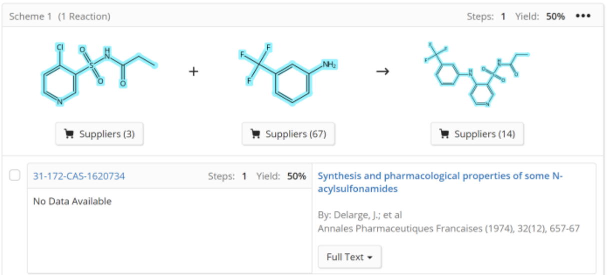	https://scifinder-n.cas.org/search/reaction/6405d0300e98827b63fee3cb/1
Step 2	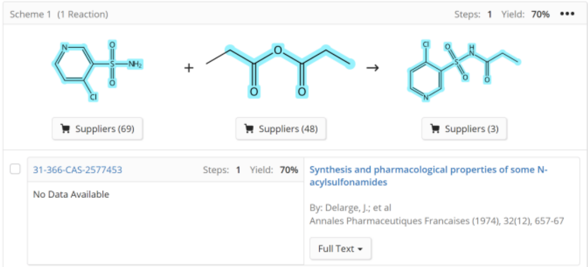	https://scifinder-n.cas.org/search/reaction/6405d0620e98827b63fee897/1
Route 29<br>Step 1	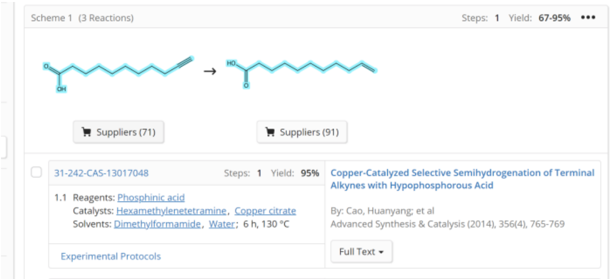	https://scifinder-n.cas.org/search/reaction/6405d0bd0e98827b63fef04f/1

Route 30<br>Step 1	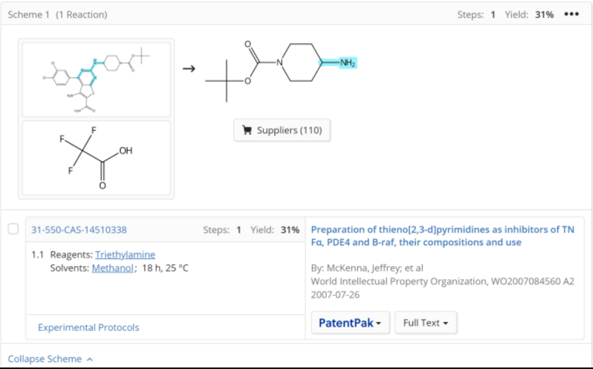	https://scifinder-n.cas.org/search/reaction/6405d3010e98827b63ff1c93/1
Step 2	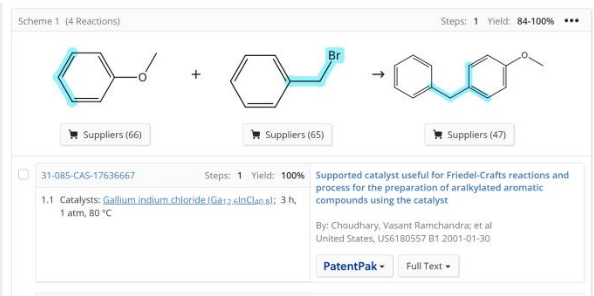	https://scifinder-n.cas.org/search/reaction/6405d3510e98827b63ff2268/1
Step 3	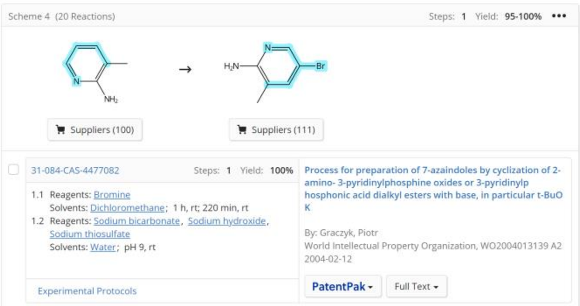	https://scifinder-n.cas.org/search/reaction/6405d5860e98827b63ff4a38/1
Route 31<br>Step 1	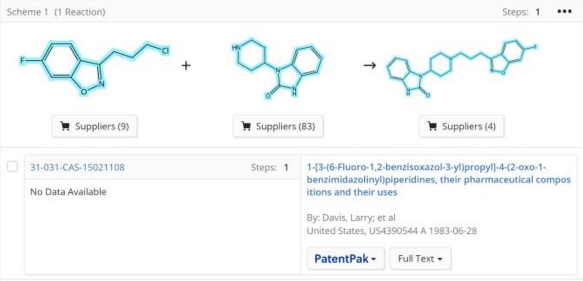	https://scifinder-n.cas.org/search/reaction/6405d8940e98827b63ff80e4/1
Route 32<br>Step 1	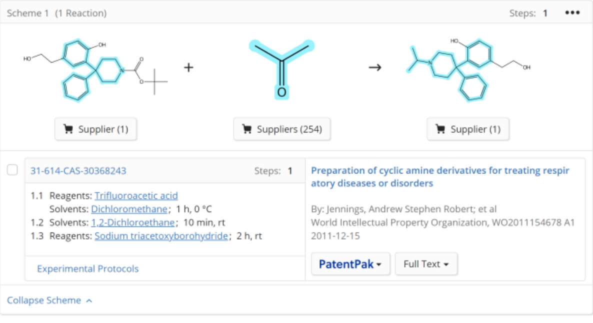	https://scifinder-n.cas.org/search/reaction/6405d8d40e98827b63ff8596/1
Route 33<br>Step 1	Null	
Route 34	Null	

Route 35<br>Step 1	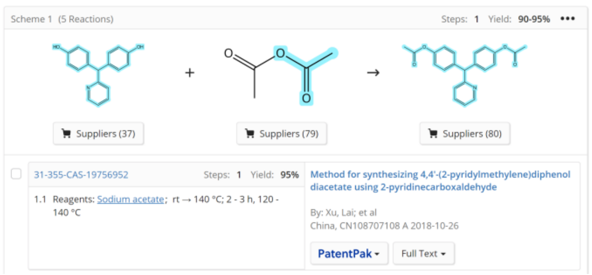	https://scifinder-n.cas.org/search/reaction/64069b4a0e98827b6309c531/1
Step 2	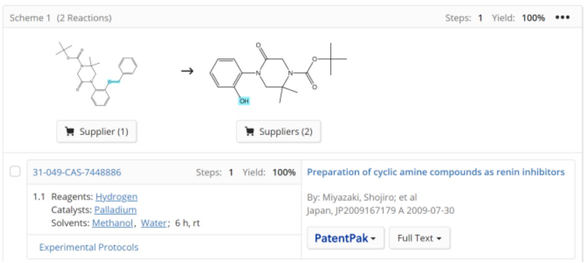	https://scifinder-n.cas.org/search/reaction/64069bf30e98827b6309d3ca/1
Step 3	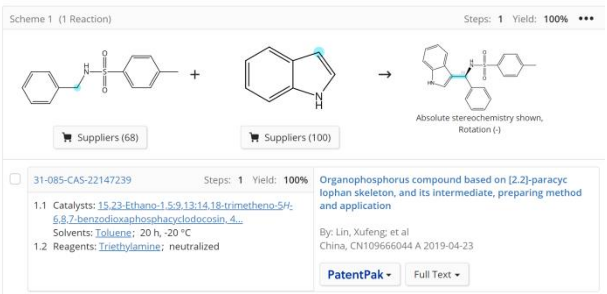	https://scifinder-n.cas.org/search/reaction/64069ccf0e98827b6309e59b/1
Route 36<br>Step 1	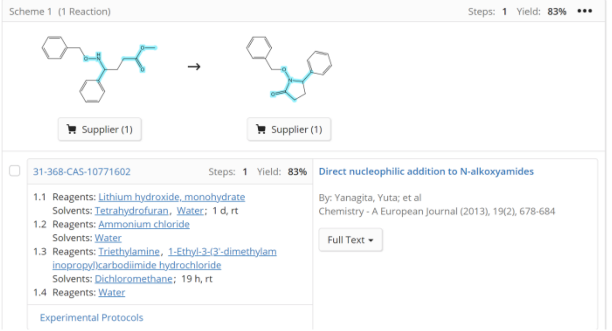	https://scifinder-n.cas.org/search/reaction/64069d110e98827b6309ea5b/1
Step 2	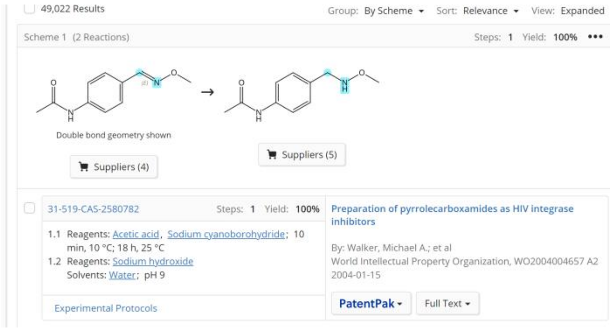	https://scifinder-n.cas.org/search/reaction/64069d850e98827b6309f339/1

Step 3	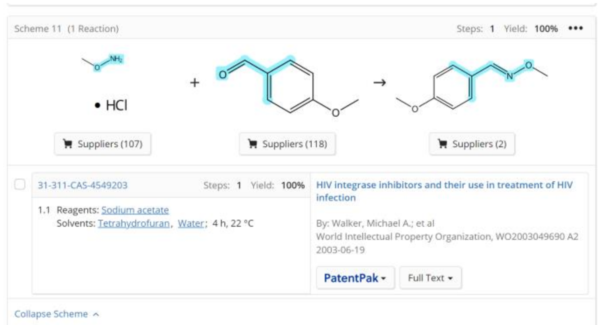	https://scifinder-n.cas.org/search/reaction/64069e000e98827b6309fcd2/1
Step 4	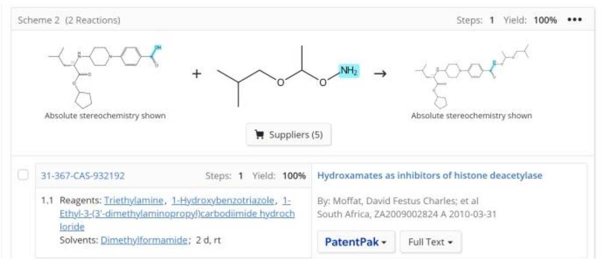	https://scifinder-n.cas.org/search/reaction/64069e3d0e98827b630a00a2/1
Step 5	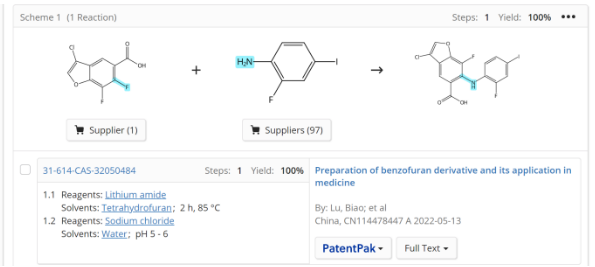	https://scifinder-n.cas.org/search/reaction/64069e9a0e98827b630a081e/1
Route 37<br>Step 1	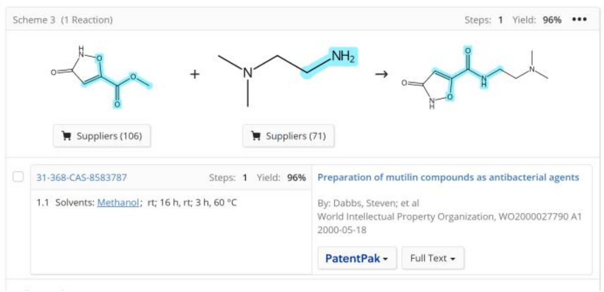	https://scifinder-n.cas.org/search/reaction/64069f100e98827b630a1063/1
Route 38<br>Step 1	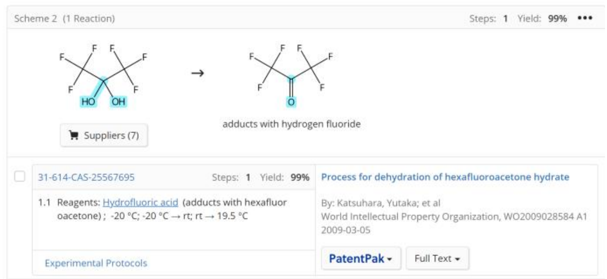	https://scifinder-n.cas.org/search/reaction/6406a1550e98827b630a40d8/1
Step 2	Null	

<table>
<tr><td>Route 39
Step 1</td><td>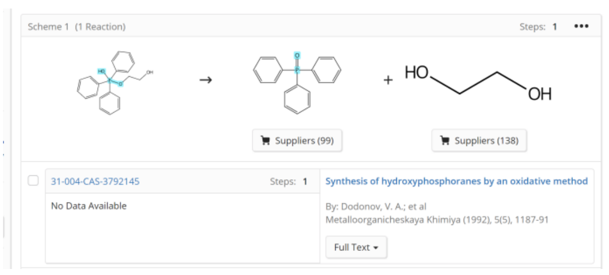
</td><td>https://scifinder-n.cas.org/search/reaction/6406a1ef0e98827b630a4d4c/1</td></tr>
<tr><td>Step 2</td><td>Null</td><td></td></tr>
<tr><td>Route 40
Step 1</td><td>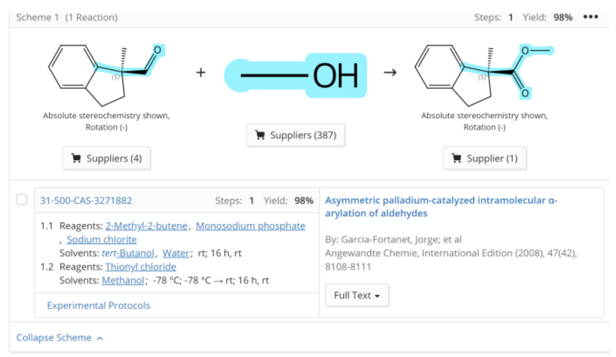
</td><td>https://scifinder-n.cas.org/search/reaction/6406a2b10e98827b630a5c21/1</td></tr>
<tr><td>Route 41
Step 1</td><td>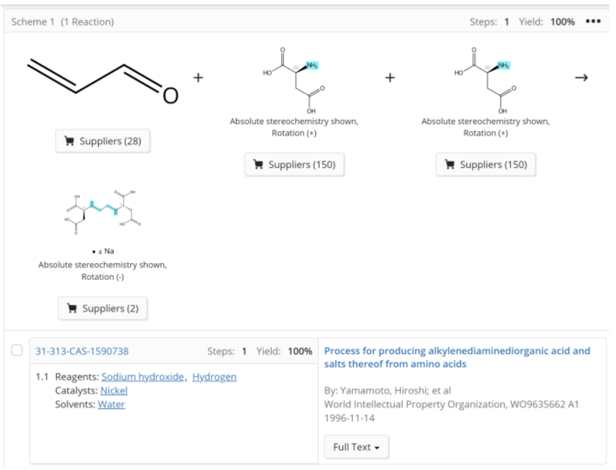
</td><td>https://scifinder-n.cas.org/search/reaction/6406a4270e98827b630a7abb/1</td></tr>
<tr><td>Route 42
Step 1</td><td>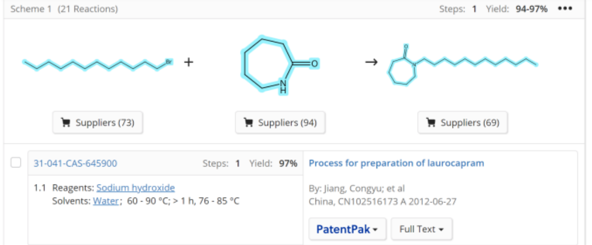
</td><td>https://scifinder-n.cas.org/search/reaction/6406a5660e98827b630a9217/1</td></tr>
<tr><td>Route 43
Step 1</td><td>Null</td><td></td></tr>
<tr><td>Step 2</td><td>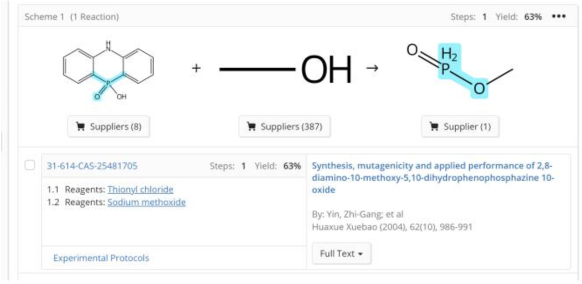
</td><td>https://scifinder-n.cas.org/search/reaction/6406a6000e98827b630a9e3a/1</td></tr>
</table>

Route 44<br>Step 1	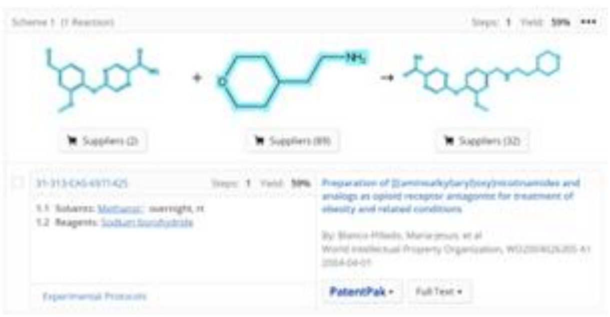	https://scifinder-n.cas.org/search/reaction/640729582b901c23fd16a807/1
Step 2	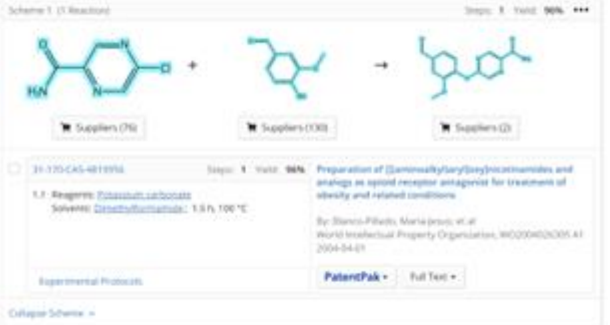	https://scifinder-n.cas.org/search/reaction/640729862b901c23fd16ab50/1
Route 45<br>Step 1	Null	
Route 46<br>Step 1	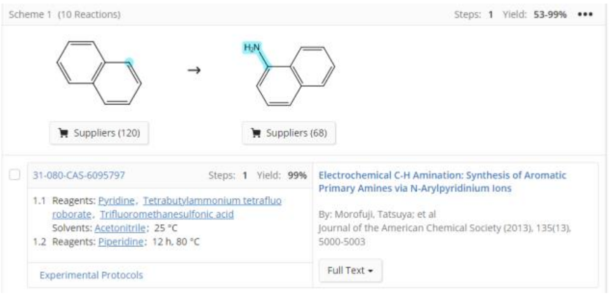	https://scifinder-n.cas.org/search/reaction/64073ce415f126035f20ffab/1
Route 47<br>Step 1	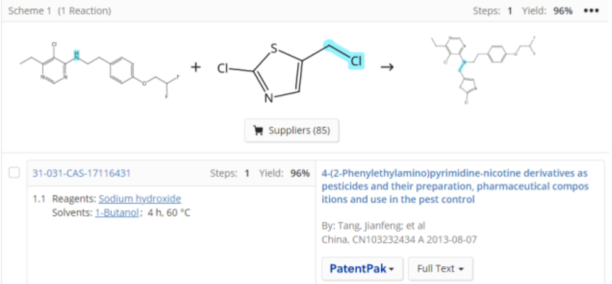	https://scifinder-n.cas.org/search/reaction/64073c5d15f126035f20f4fc/1
Route 48<br>Step 1	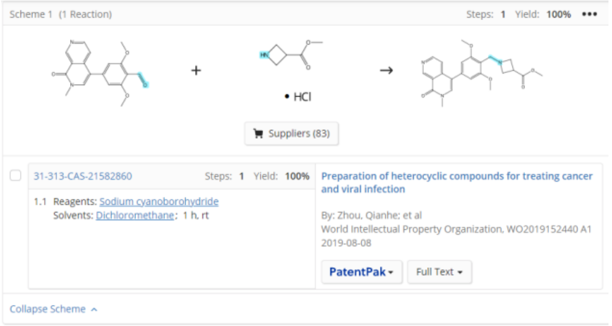	https://scifinder-n.cas.org/search/reaction/64073d1a15f126035f210346/1

<table>
<tr><td>Route 49
Step 1</td><td>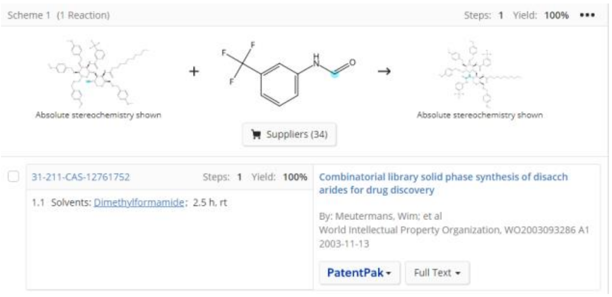
</td><td>https://scifinder-n.cas.org/search/reaction/64073d5915f126035f2107b6/1</td></tr>
<tr><td>Route 50
Step 1</td><td>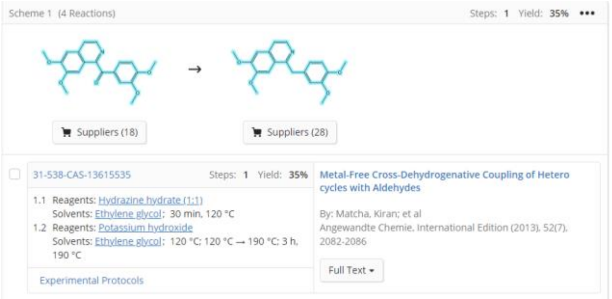
</td><td>https://scifinder-n.cas.org/search/reaction/64073db615f126035f210dcc/1</td></tr>
<tr><td>Route 51
Step 1</td><td>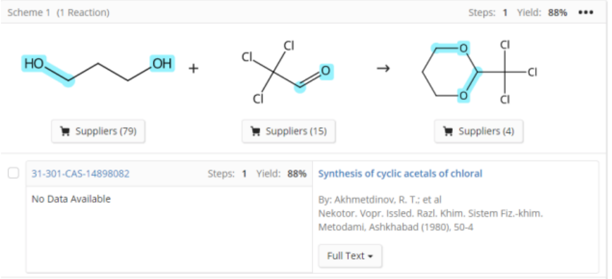
</td><td>https://scifinder-n.cas.org/search/reaction/64073e1d15f126035f211457/1</td></tr>
<tr><td>Route 52
Step 1</td><td>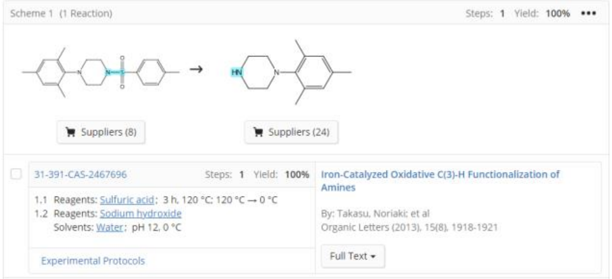
</td><td>https://scifinder-n.cas.org/search/reaction/64073e5f15f126035f211947/1</td></tr>
<tr><td>Step 2</td><td>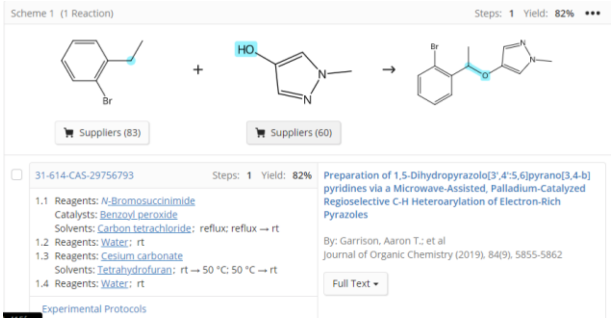
</td><td>https://scifinder-n.cas.org/search/reaction/64073ea115f126035f211e1b/1</td></tr>
</table>

Route 53<br>Step 1	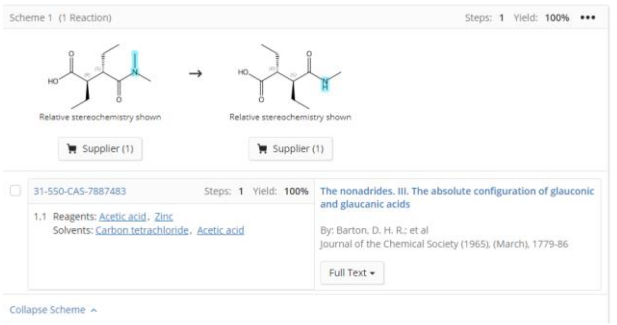	https://scifinder-n.cas.org/search/reaction/64073f2d15f126035f212a49/1
Step 2	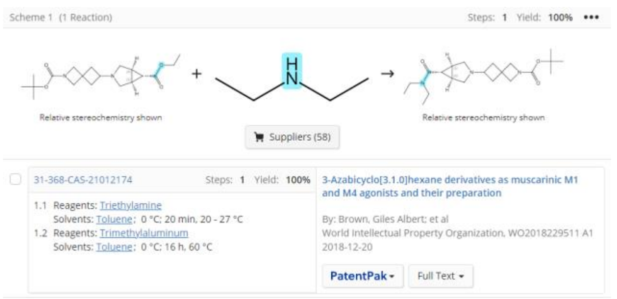	https://scifinder-n.cas.org/search/reaction/64073f8f15f126035f213338/1
Route 54<br>Step 1	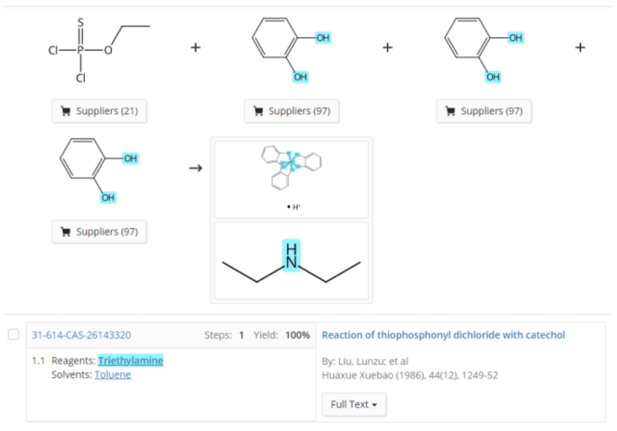	https://scifinder-n.cas.org/search/reaction/64073fbd15f126035f213625/1
Step 2	Null	
Route 55<br>Step 1	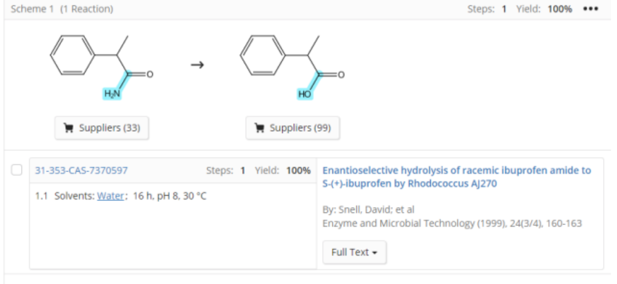	https://scifinder-n.cas.org/search/reaction/6407402315f126035f213d57/1

<table>
<tr>
<td>Route 56
Step 1</td>
<td>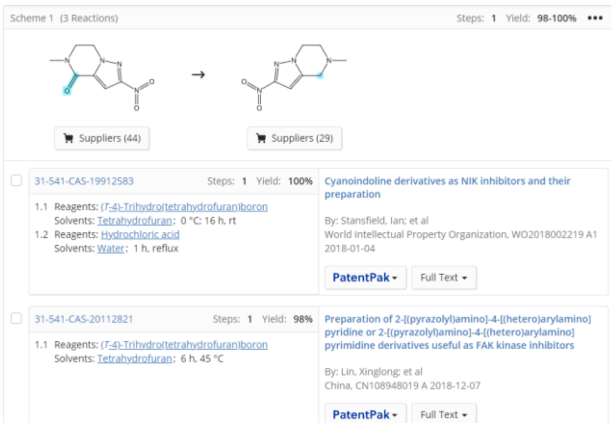
</td>
<td>https://scifinder-n.cas.org/search/reaction/6407407715f126035f2144be/1</td>
</tr>
<tr>
<td>Step 2</td>
<td>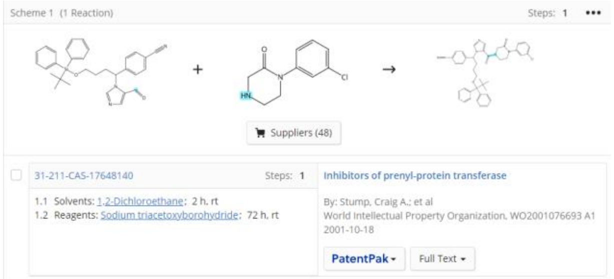
</td>
<td>https://scifinder-n.cas.org/search/reaction/640740bd15f126035f214b4e/1</td>
</tr>
<tr>
<td>Route 57
Step 1</td>
<td>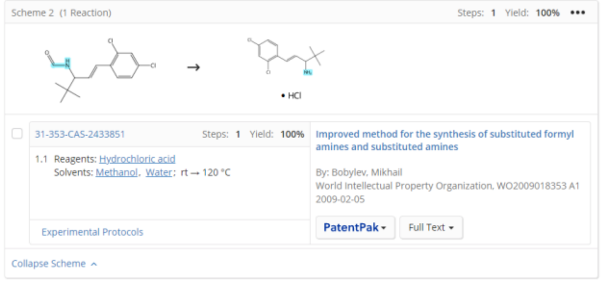
</td>
<td>https://scifinder-n.cas.org/search/reaction/640740f415f126035f215051/1</td>
</tr>
<tr>
<td>Step 2</td>
<td>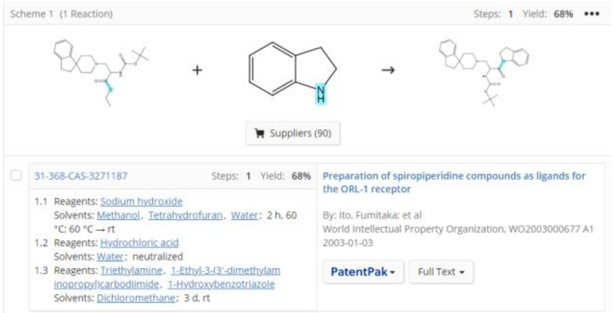
</td>
<td>https://scifinder-n.cas.org/search/reaction/6407416f15f126035f2158ef/1</td>
</tr>
<tr>
<td>Route 58
Step 1</td>
<td>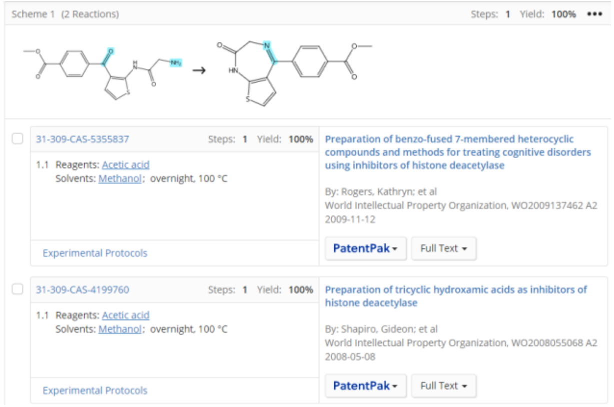
</td>
<td>https://scifinder-n.cas.org/search/reaction/640741b515f126035f215ecc/1</td>
</tr>
</table>

<table>
<tr>
<td>Step 2</td>
<td>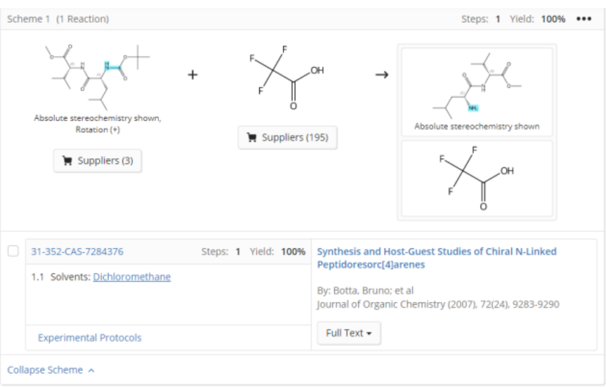
</td>
<td>https://scifinder-n.cas.org/search/reaction/640741fb15f126035f2163f6/1</td>
</tr>
<tr>
<td>Step 3</td>
<td>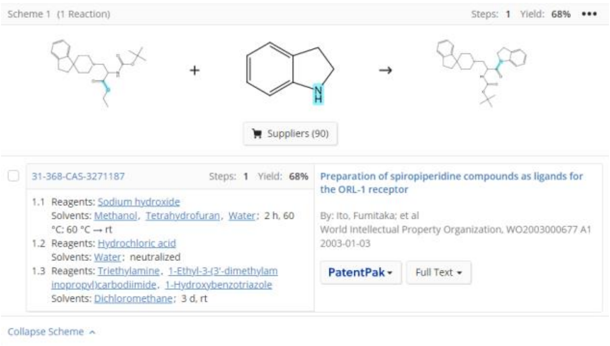
</td>
<td>https://scifinder-n.cas.org/search/reaction/6407424015f126035f216963/1</td>
</tr>
<tr>
<td>Route 59
Step 1</td>
<td>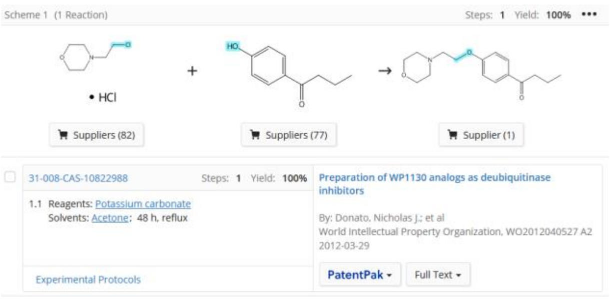
</td>
<td>https://scifinder-n.cas.org/search/reaction/640731e69bdefb0477012104/1</td>
</tr>
<tr>
<td>Route 60
Step 1</td>
<td>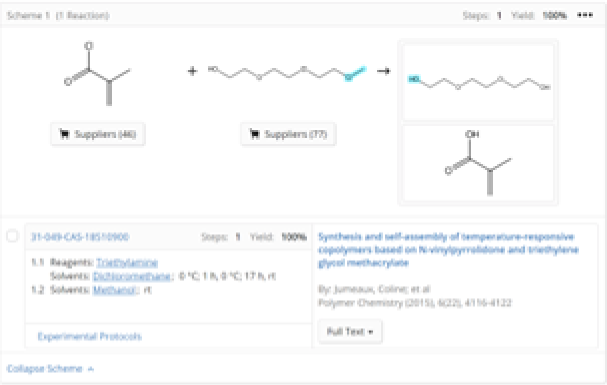
</td>
<td>https://scifinder-n.cas.org/search/reaction/6407341b2b901c23fd1767dd/1</td>
</tr>
</table>

Step 2	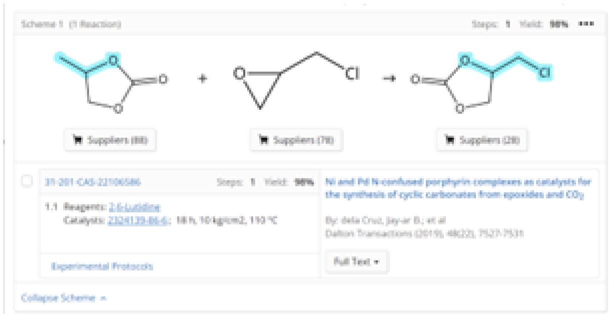	https://scifinder-n.cas.org/search/reaction/640734982b901c23fd176e98/1
Route 61<br>Step 1	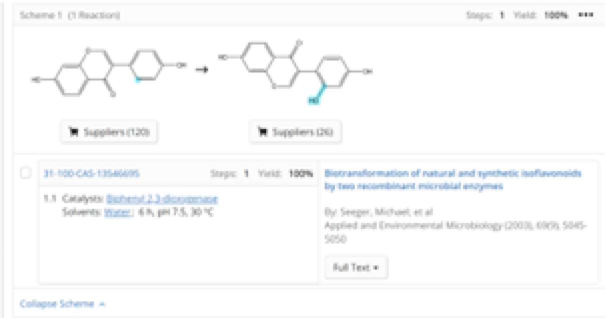	https://scifinder-n.cas.org/search/reaction/640735302b901c23fd1776de/1
Route 62<br>Step 1	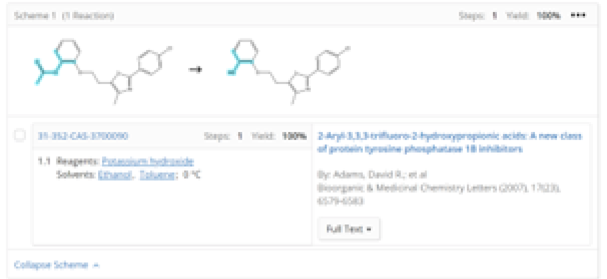	https://scifinder-n.cas.org/search/reaction/6407359b2b901c23fd177e13/1
Route 63<br>Step 1	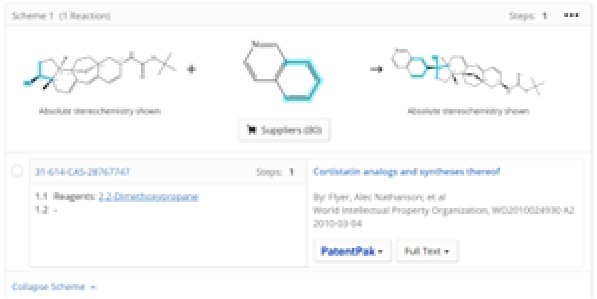	https://scifinder-n.cas.org/search/reaction/640735ed2b901c23fd178365/1
Step 2	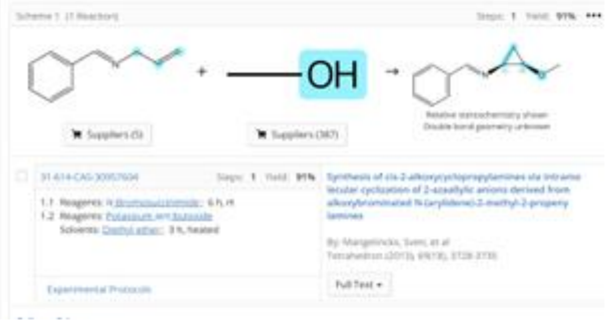	https://scifinder-n.cas.org/search/reaction/6407364c2b901c23fd178bb6/1

Route 64<br>Step 1	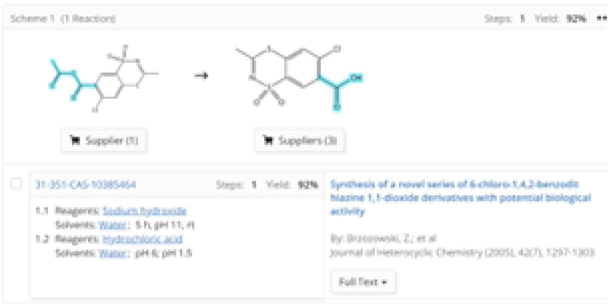	https://scifinder-n.cas.org/search/reaction/640736eb2b901c23fd179967/1
Step 2	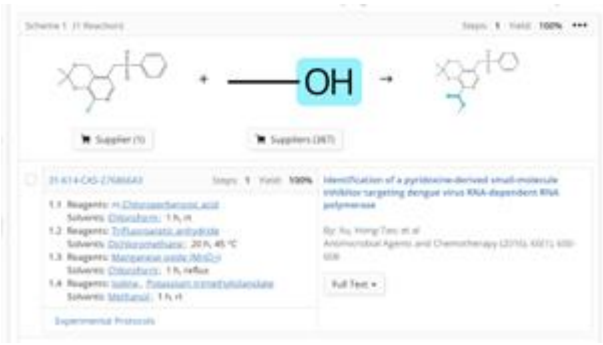	https://scifinder-n.cas.org/search/reaction/6407371a2b901c23fd179dc0/1
Step 3	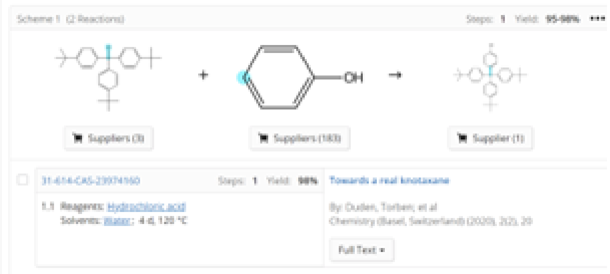	https://scifinder-n.cas.org/search/reaction/6407379b2b901c23fd17a7c4/1
Route 65<br>Step 1	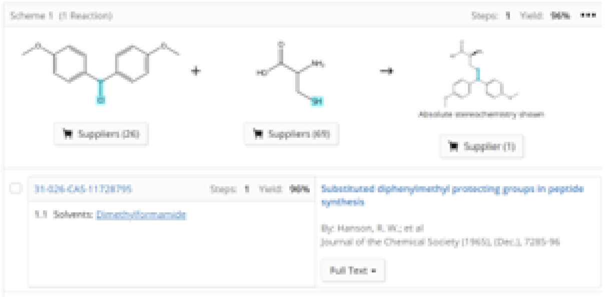	https://scifinder-n.cas.org/search/reaction/640737c22b901c23fd17ab20/1
Step 2	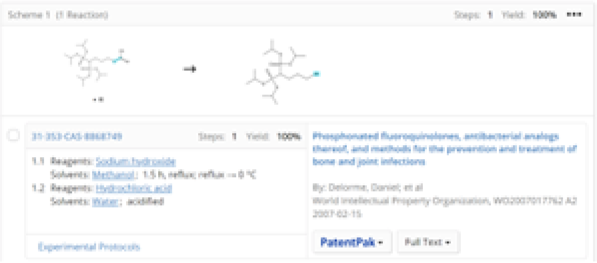	https://scifinder-n.cas.org/search/reaction/640737e32b901c23fd17ae00/1

Step 3	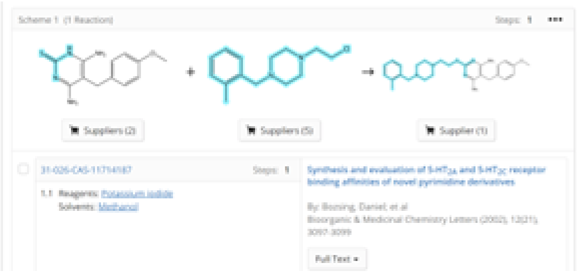	https://scifinder-n.cas.org/search/reaction/6407380b2b901c23fd17b1aa/1
Route 66<br>Step 1	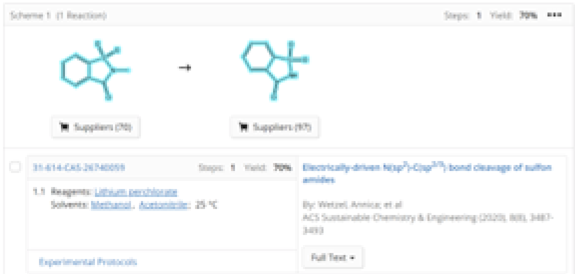	https://scifinder-n.cas.org/search/reaction/640738362b901c23fd17b503/1
Route 67<br>Step 1	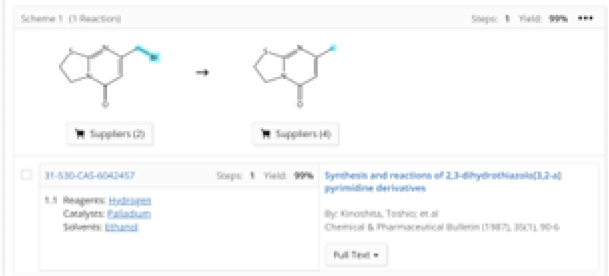	https://scifinder-n.cas.org/search/reaction/6407386a2b901c23fd17bacc/1
Step 2	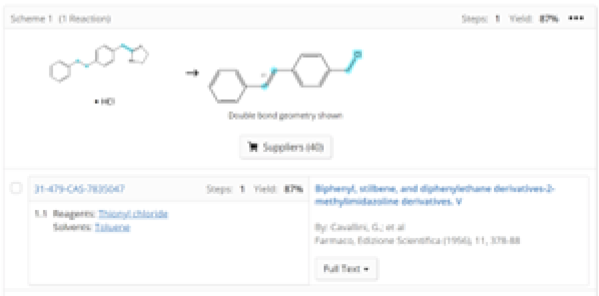	https://scifinder-n.cas.org/search/reaction/6407388b2b901c23fd17bdce/1
Route 68<br>Step 1	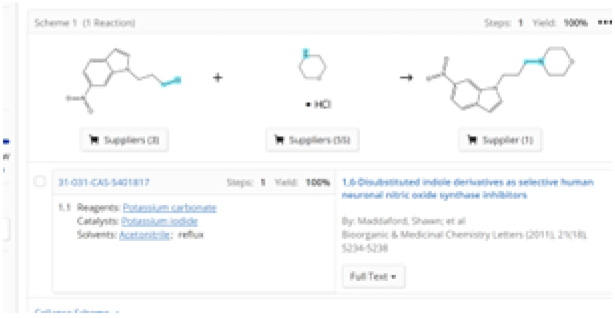	https://scifinder-n.cas.org/search/reaction/640738aa2b901c23fd17c068/1
Route 69<br>Step 1	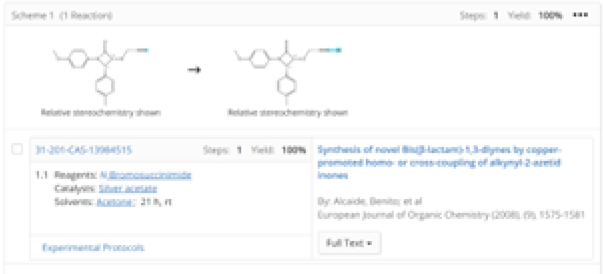	https://scifinder-n.cas.org/search/reaction/6407390c2b901c23fd17c822/1

Route 70<br>Step 1	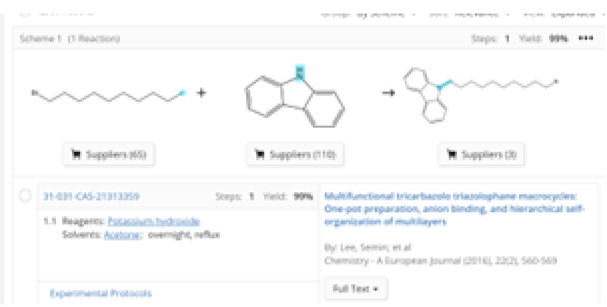	https://scifinder-n.cas.org/search/reaction/6407399f2b901c23fd17d3ab/1
Route 71<br>Step 1	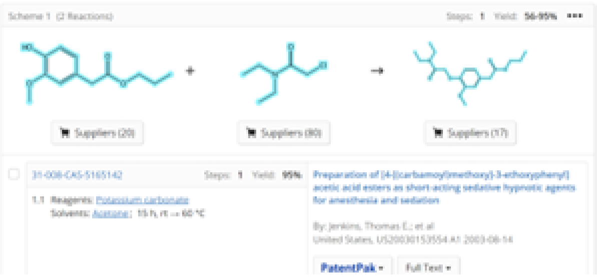	https://scifinder-n.cas.org/search/reaction/640739d32b901c23fd17d7da/1
Step 2	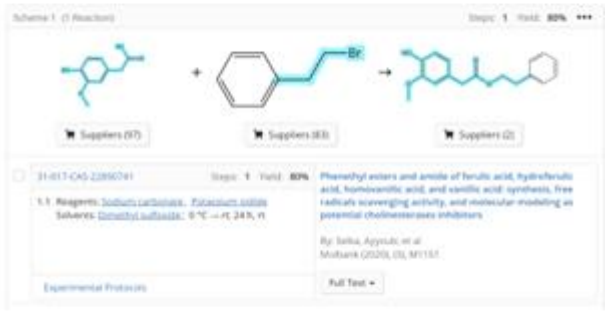	https://scifinder-n.cas.org/search/reaction/64073a042b901c23fd17db7d/1
Route 72<br>Step 1	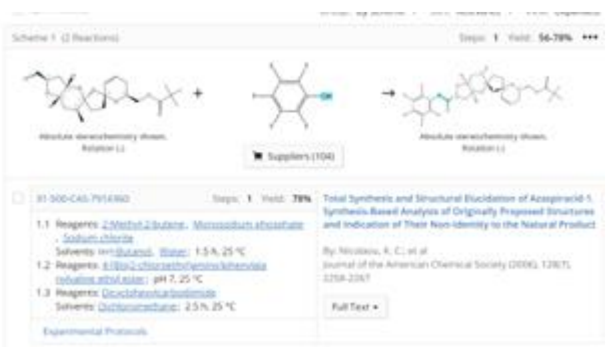	https://scifinder-n.cas.org/search/reaction/64073adc2b901c23fd17eb2a/1
Route 73<br>Step 1	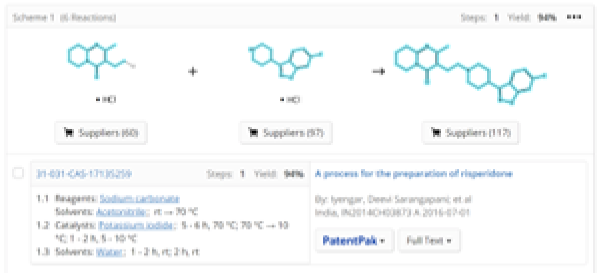	https://scifinder-n.cas.org/search/reaction/64073b0c2b901c23fd17ee4b/1

<table>
<tr>
<td>Route 74
Step 1</td>
<td>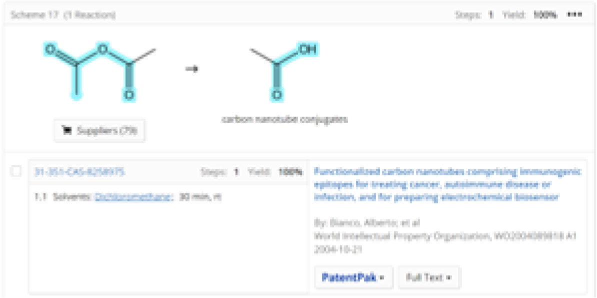
</td>
<td>https://scifinder-n.cas.org/search/reaction/64073c8d2b901c23fd180ab2/1</td>
</tr>
<tr>
<td>Step 2</td>
<td>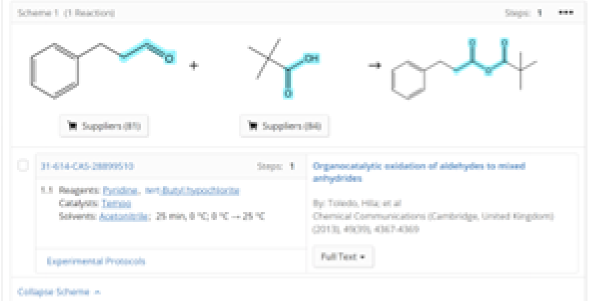
</td>
<td>https://scifinder-n.cas.org/search/reaction/64073d062b901c23fd1812e8/1</td>
</tr>
<tr>
<td>Step 3</td>
<td>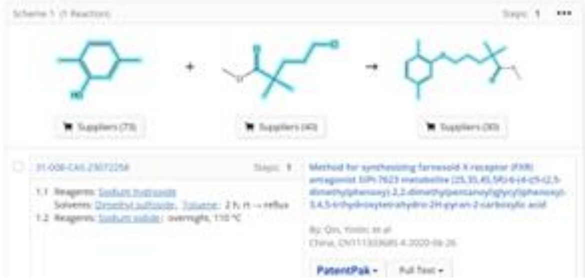</td>
<td>https://scifinder-n.cas.org/search/reaction/64073d352b901c23fd181666/1</td>
</tr>
<tr>
<td>Route 75
Step 1</td>
<td>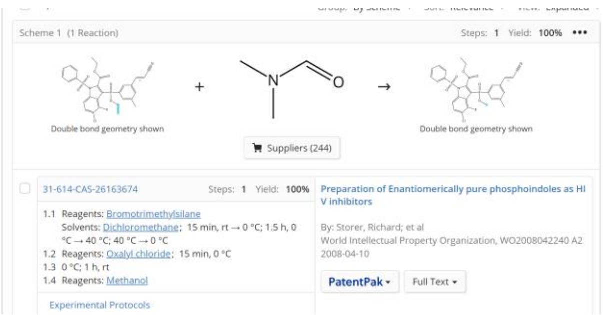
</td>
<td>https://scifinder-n.cas.org/search/reaction/640745833136c60ac9448417/1</td>
</tr>
<tr>
<td>Step 2</td>
<td>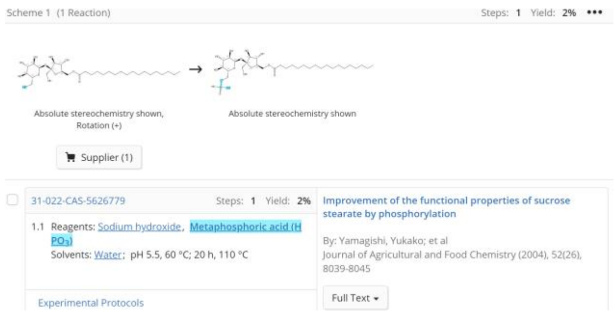
</td>
<td>https://scifinder-n.cas.org/search/reaction/640745a53136c60ac944866d/1</td>
</tr>
</table>

Step 3	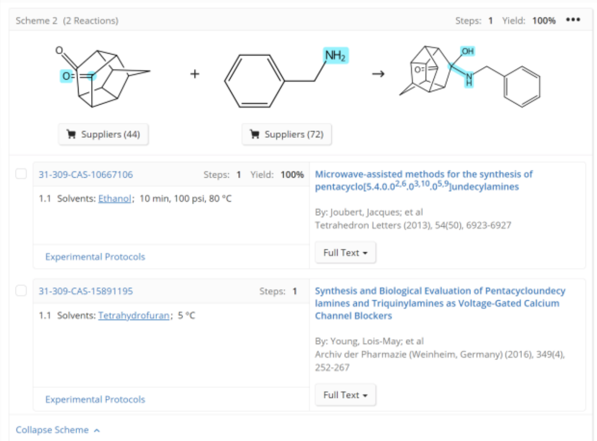	https://scifinder-n.cas.org/search/reaction/640745f63136c60ac9448c96/1
Route 76<br>Step 1	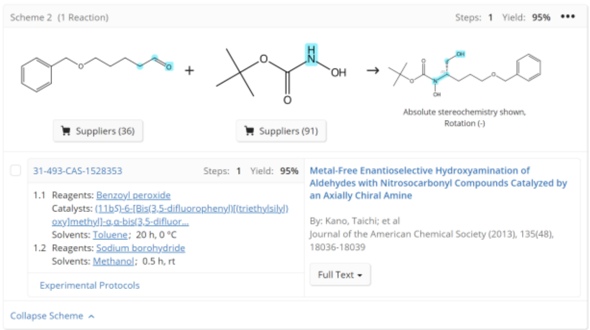	https://scifinder-n.cas.org/search/reaction/640746673136c60ac94495c4/1
Route 77<br>Step 1	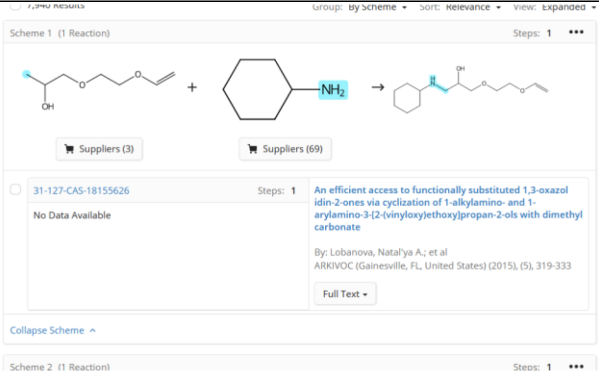	https://scifinder-n.cas.org/search/reaction/6407465b9bdefb04770285d4/1
Step 2	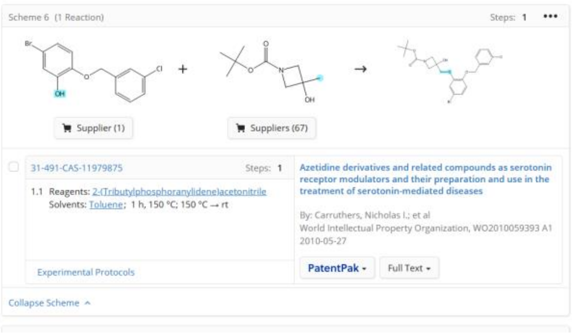	https://scifinder-n.cas.org/search/reaction/640746929bdefb0477028927/1
Route 78<br>Step 1	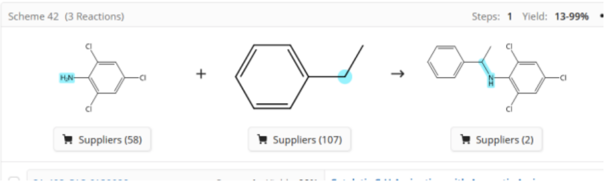	https://scifinder-n.cas.org/search/reaction/640745b89bdefb0477027bd5/3

<table>
<tr><td>Route 79
Step 1</td><td>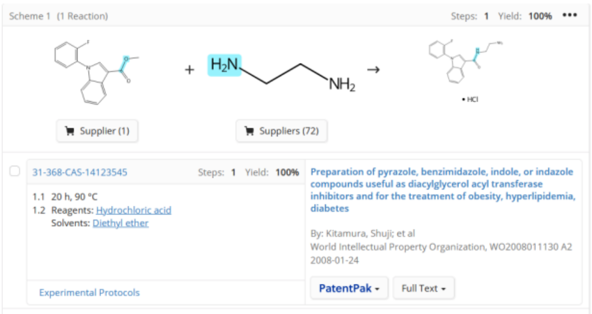</td><td>https://scifinder-n.cas.org/search/reaction/640745239bdefb0477027181/1</td></tr>
<tr><td>Step 2</td><td>Null</td><td></td></tr>
<tr><td>Route 80
Step 1</td><td>Null</td><td></td></tr>
<tr><td>Route 81
Step 1</td><td>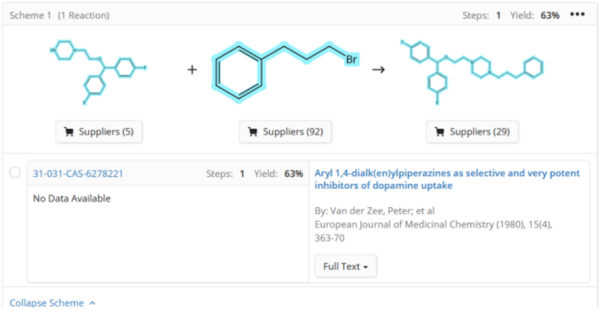</td><td>https://scifinder-n.cas.org/search/reaction/640744909bdefb04770269b7/1</td></tr>
<tr><td>Route 82
Step 1</td><td>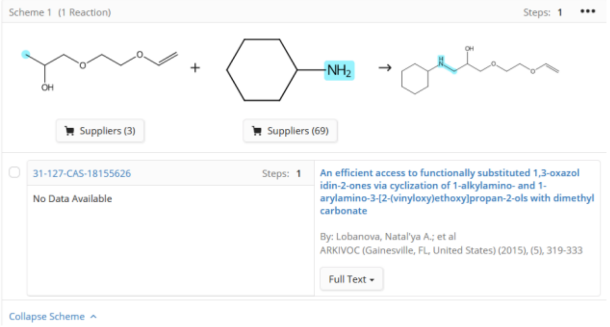</td><td>https://scifinder-n.cas.org/search/reaction/640744639bdefb0477026769/1</td></tr>
<tr><td>Route 83
Step 1</td><td>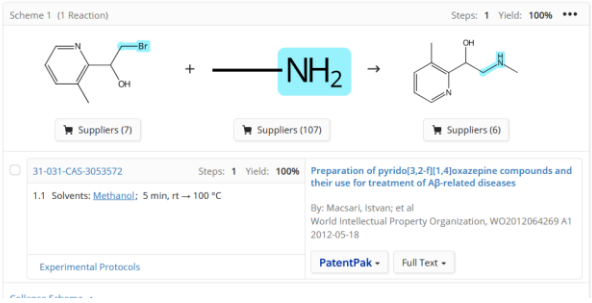</td><td>https://scifinder-n.cas.org/search/reaction/640744359bdefb04770264b5/1</td></tr>
<tr><td>Route 84
Step 1</td><td>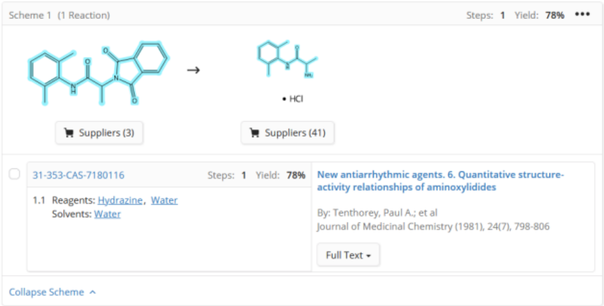</td><td>https://scifinder-n.cas.org/search/reaction/640743d59bdefb0477025f45/1</td></tr>
</table>

Route 85<br>Step 1	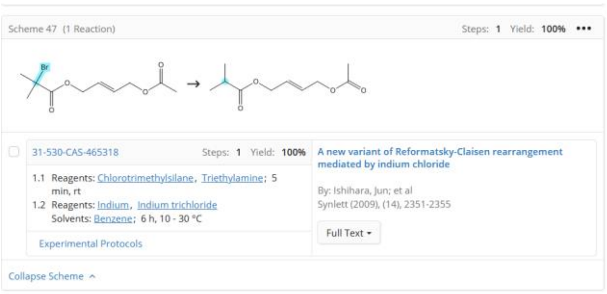	https://scifinder-n.cas.org/search/reaction/640742e09bdefb0477024f45/3
Step 2	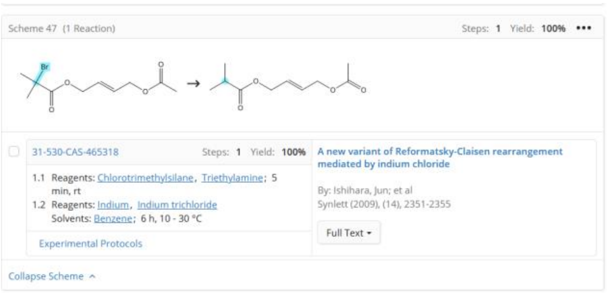	https://scifinder-n.cas.org/search/reaction/640742e09bdefb0477024f45/3
Step 3	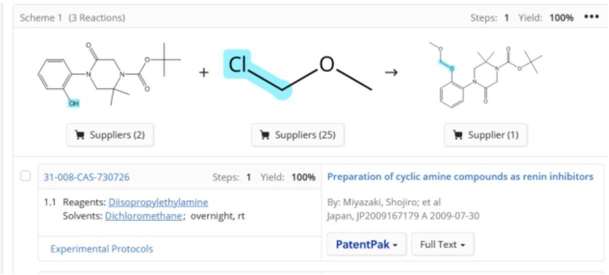	https://scifinder-n.cas.org/search/reaction/640742439bdefb04770241ef/1
Step 4	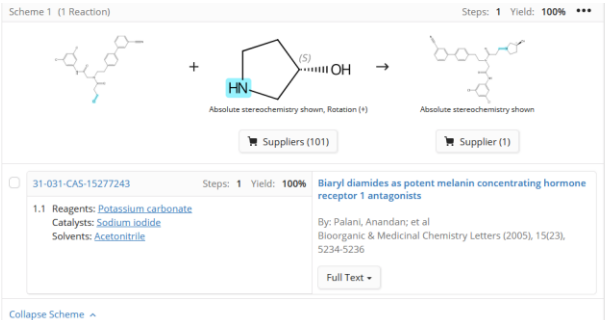	https://scifinder-n.cas.org/search/reaction/640742209bdefb0477023fa2/1
Route 86<br>Step 1	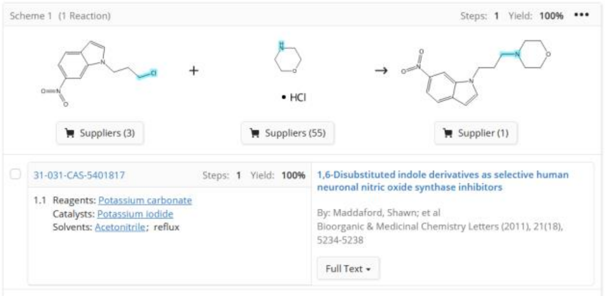	https://scifinder-n.cas.org/search/reaction/640740e59bdefb0477022a63/1
Step 2		https://scifinder-n.cas.org/search/reaction/640741129bdefb0477022df9/1

<table>
<tr><td></td><td>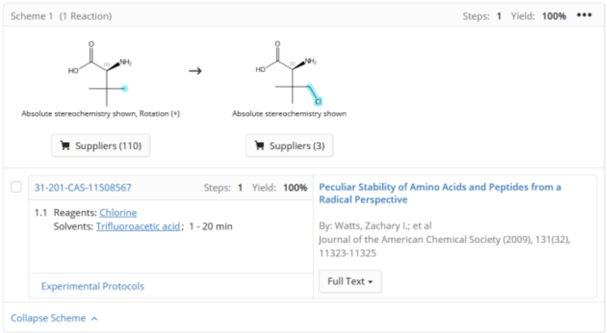
</td><td></td></tr>
<tr><td>Route 87
Step 1</td><td>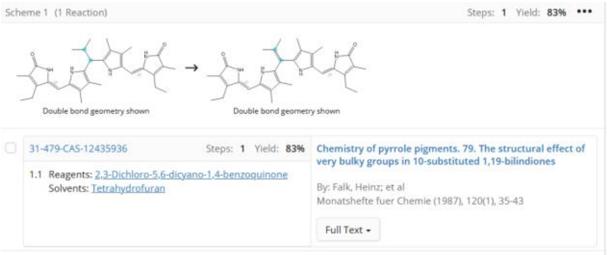
</td><td>https://scifinder-n.cas.org/search/reaction/640740ab9bdefb04770226cc/1</td></tr>
<tr><td>Step 2</td><td>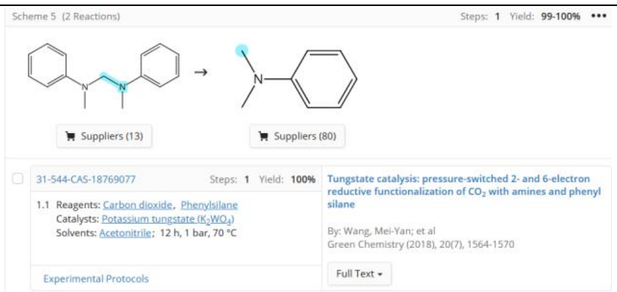
</td><td>https://scifinder-n.cas.org/search/reaction/640740459bdefb0477021fa3/1</td></tr>
<tr><td>Step 3</td><td>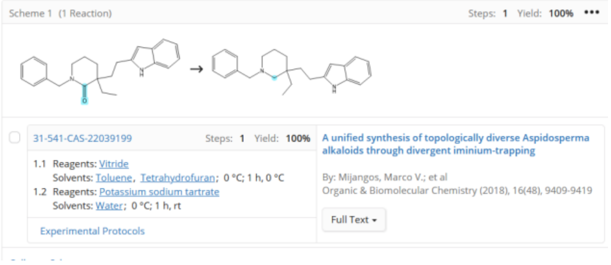
</td><td>https://scifinder-n.cas.org/search/reaction/640740199bdefb0477021ce0/1</td></tr>
<tr><td>Step 4</td><td>Null</td><td></td></tr>
<tr><td>Route 88
Step 1</td><td>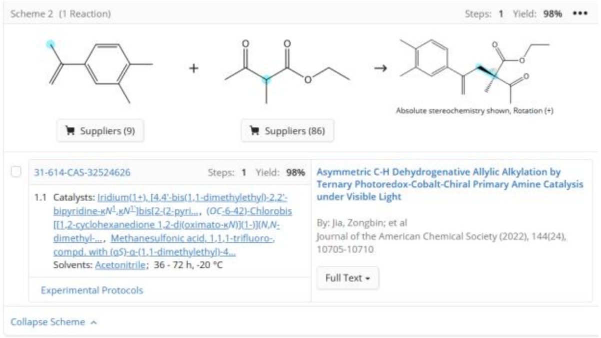
</td><td>https://scifinder-n.cas.org/search/reaction/64073f549bdefb047702113a/1</td></tr>
</table>

<table>
<tr><td>Route 89
Step 1</td><td>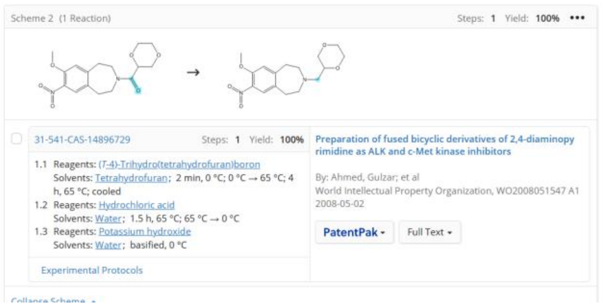
</td><td>https://scifinder-n.cas.org/search/reaction/64073ec79bdefb0477020931/1</td></tr>
<tr><td>Step 2</td><td>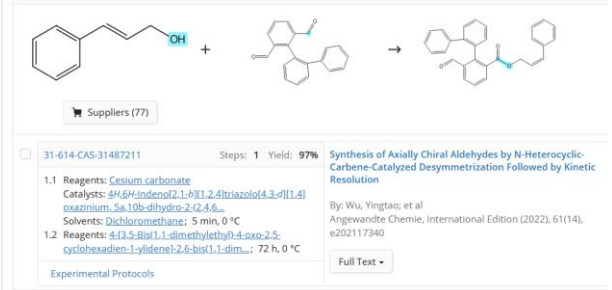
</td><td>https://scifinder-n.cas.org/search/reaction/64073ea59bdefb04770206b4/1</td></tr>
<tr><td>Step 3</td><td>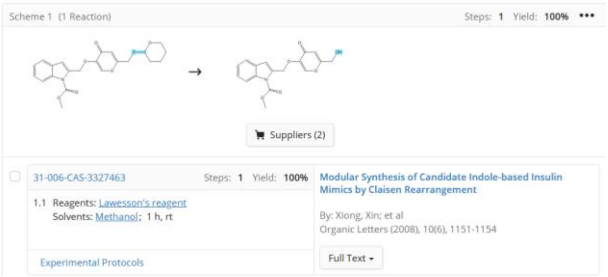
</td><td>https://scifinder-n.cas.org/search/reaction/64073e5d9bdefb04770202bc/1</td></tr>
<tr><td>Step 4</td><td>Null</td><td></td></tr>
<tr><td>Route 90
Step 1</td><td>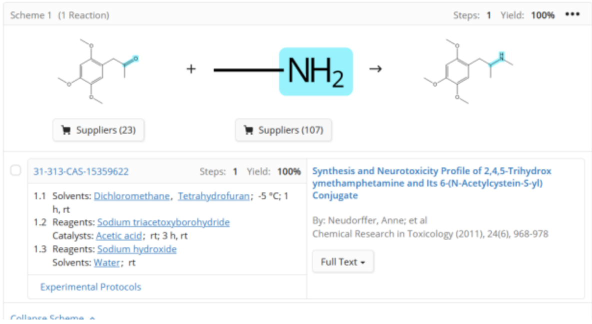
</td><td>https://scifinder-n.cas.org/search/reaction/64073cd79bdefb047701ebfe/1</td></tr>
<tr><td>Route 91
Step 1</td><td>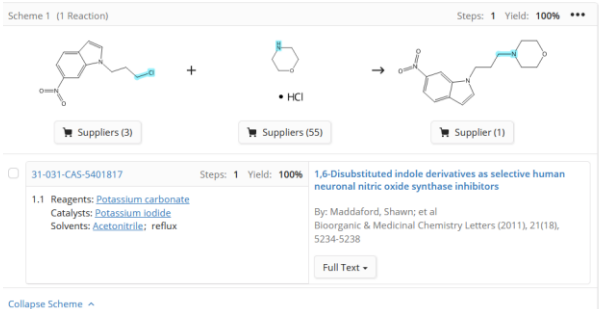
</td><td>https://scifinder-n.cas.org/search/reaction/64073c229bdefb047701e05e/1</td></tr>
</table>

Route 92<br>Step 1	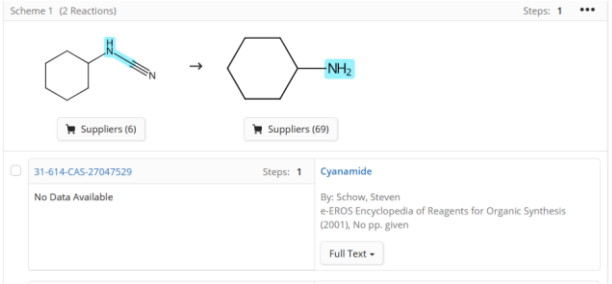	https://scifinder-n.cas.org/search/reaction/64073bdb9bdefb047701dc21/1
Route 93<br>Step 1	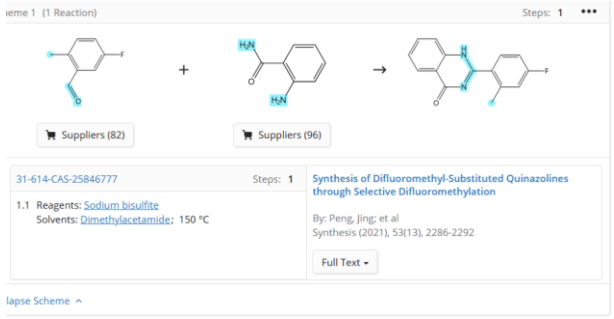	https://scifinder-n.cas.org/search/reaction/64073b2a9bdefb047701d09b/1
Route 94<br>Step 1	Null	
Step 2	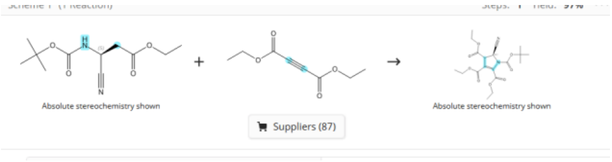	https://scifinder-n.cas.org/search/reaction/640738679bdefb047701a1e0/1
Route 95<br>Step 1	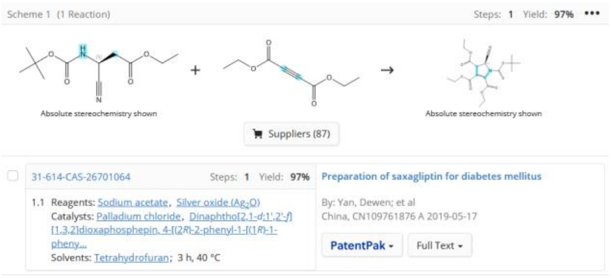	https://scifinder-n.cas.org/search/reaction/640738679bdefb047701a1e0/1
Step 2	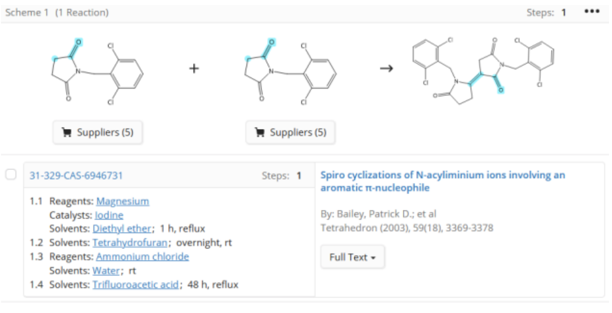	https://scifinder-n.cas.org/search/reaction/640737e59bdefb04770197e0/1
Route 96<br>Step 1	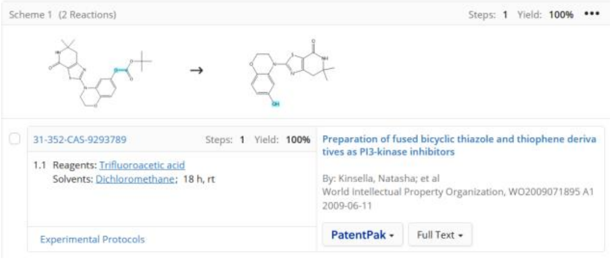	https://scifinder-n.cas.org/search/reaction/640737349bdefb0477018b37/1

Step 2	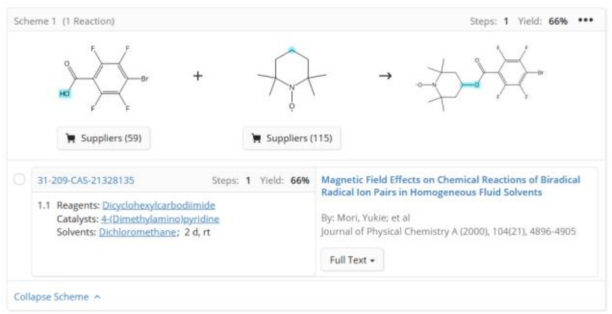	https://scifinder-n.cas.org/search/reaction/6407376e9bdefb0477018f20/1
Route 97<br>Step 1	Null	
Step 2	Null	
Route 98<br>Step 1	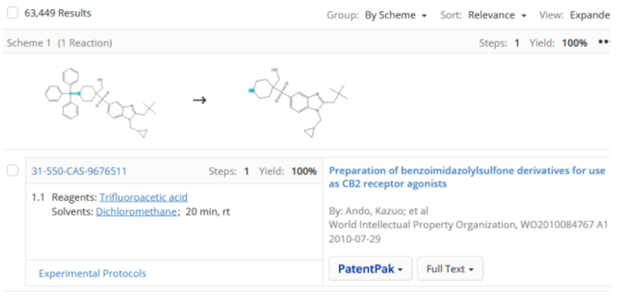	https://scifinder-n.cas.org/search/reaction/640735569bdefb04770169ba/1
Step 2	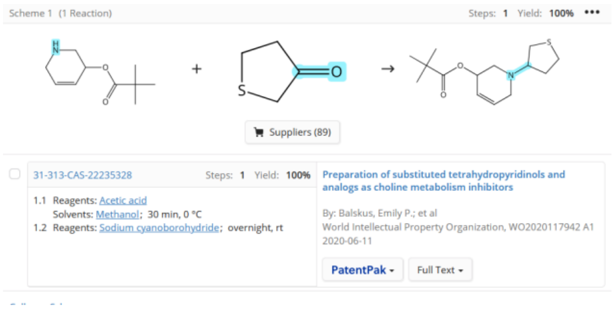	https://scifinder-n.cas.org/search/reaction/640735ae9bdefb04770170a2/1
Step 3	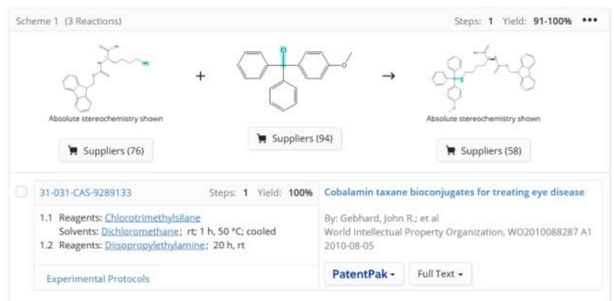	https://scifinder-n.cas.org/search/reaction/640735ed9bdefb04770175b0/1
Route 99<br>Step 1	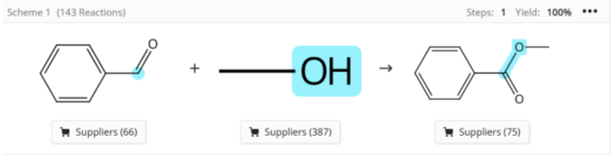	https://scifinder-n.cas.org/search/reaction/640732d09bdefb04770132ae/1

Step 2	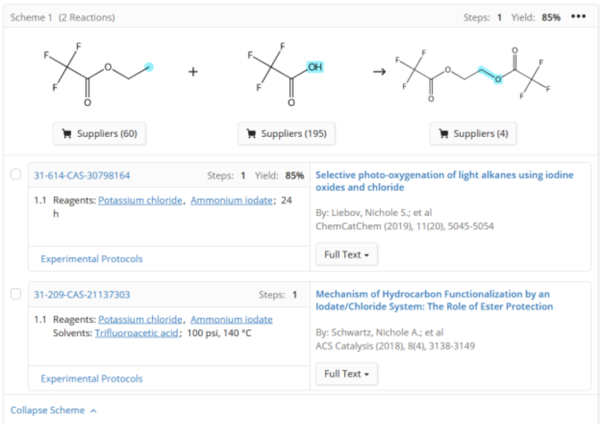	https://scifinder-n.cas.org/search/reaction/640734429bdefb04770154c7/1
Step 3	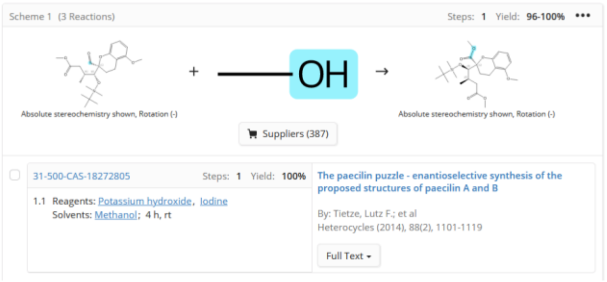	https://scifinder-n.cas.org/search/reaction/640734bd9bdefb0477015ca5/1
Route 100<br>Step 1	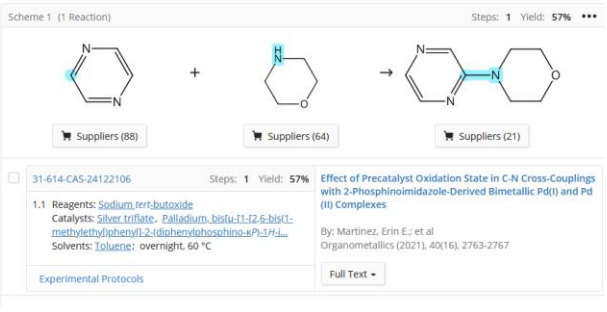	https://scifinder-n.cas.org/search/reaction/64072fe79bdefb047700ffa5/1
Route 101<br>Step 1	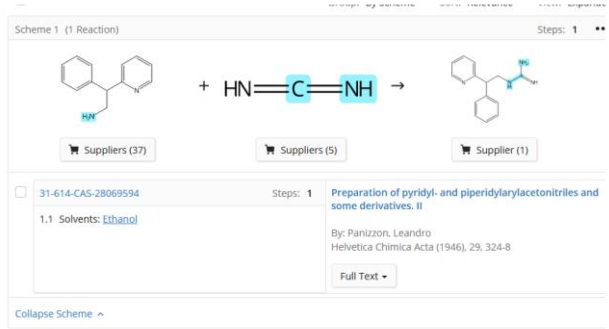	https://scifinder-n.cas.org/search/reaction/64072eb59bdefb047700eb2f/1
