# Supplementary material for: Retrosynthesis prediction with an interpretable deep-learning framework based on molecular assembly tasks
Source: Nat Commun. 2023 Oct 3;14:6155. doi: 10.1038/s41467-023-41698-5 (PMC10547708; doi:10.1038/s41467-023-41698-5)
Supplement: Supplementary file 5 — Supplementary Data 2 [file 41467_2023_41698_MOESM5_ESM.pdf]

```
|Route 1|-------------------------------------------------------------------------------------------------------
|Step  1|-------------------------------------------------------------------------------------------------------
Retrosynthesis:
O=C(N[C@H]1N=C(c2ccccc2F)c2cccc3c2N(CC3)C1=O)c1cc2ccccc2[nH]1>>O=Cc1cc2ccccc2[nH]1.NC(N)C(=O)N1CCc2cccc(C(=O)c3ccccc3F)c21
Reaction:
O=Cc1cc2ccccc2[nH]1.NC(N)C(=O)N1CCc2cccc(C(=O)c3ccccc3F)c21>>O=C(N[C@H]1N=C(c2ccccc2F)c2cccc3c2N(CC3)C1=O)c1cc2ccccc2[nH]1
```

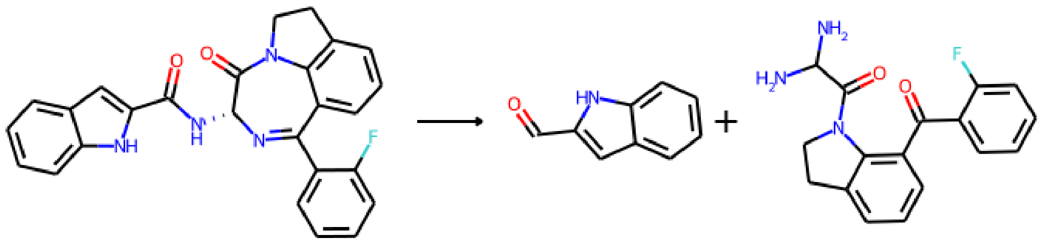

```
|Step  2|-------------------------------------------------------------------------------------------------------
Retrosynthesis:
NC(N)C(=O)N1CCc2cccc(C(=O)c3ccccc3F)c21>>NC(C(=O)N1CCc2cccc(C(=O)c3ccccc3F)c21)N1C(=O)c2ccccc2C1=O
Reaction:
NC(C(=O)N1CCc2cccc(C(=O)c3ccccc3F)c21)N1C(=O)c2ccccc2C1=O>>NC(N)C(=O)N1CCc2cccc(C(=O)c3ccccc3F)c21
```

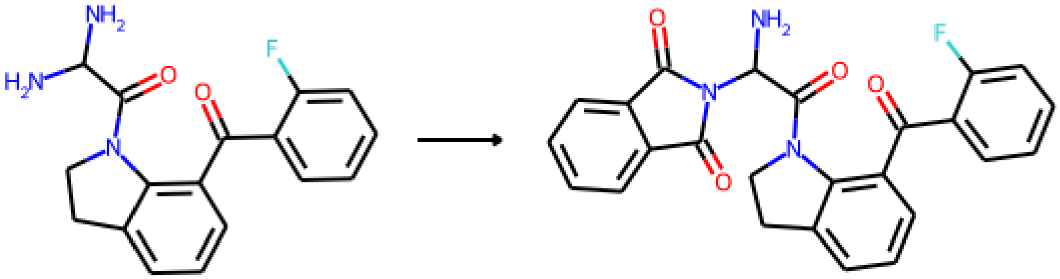


```
|Step  3|------------------------------------------------------------------------------------------------------
Retrosynthesis:
NC(C(=O)N1CCc2cccc(C(=O)c3ccccc3F)c21)N1C(=O)c2ccccc2C1=O>>NC(=O)OCc1ccccc1.O=Cc1ccccc1F.O=C1c2ccccc2C(=O)N1CC(=O)N1CCc2ccccc21
Reaction:
NC(=O)OCc1ccccc1.O=Cc1ccccc1F.O=C1c2ccccc2C(=O)N1CC(=O)N1CCc2ccccc21>>NC(C(=O)N1CCc2cccc(C(=O)c3ccccc3F)c21)N1C(=O)c2ccccc2C1=O
```

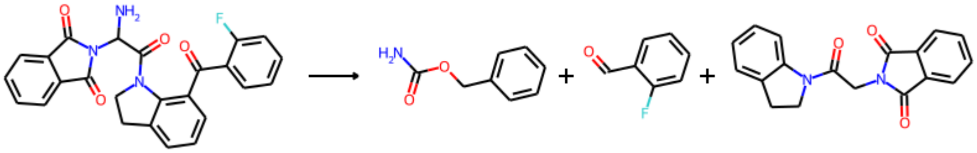

```
|Route 2|------------------------------------------------------------------------------------------------------
|Step  1|------------------------------------------------------------------------------------------------------
Retrosynthesis:
Cn1c(CCCC(=O)O)nc2cc(N(CCCl)CCCl)ccc21>>Cn1c(CCCC=O)nc2cc(N(CCCl)CCCl)ccc21.O
Reaction:
Cn1c(CCCC=O)nc2cc(N(CCCl)CCCl)ccc21.O>>Cn1c(CCCC(=O)O)nc2cc(N(CCCl)CCCl)ccc21
```

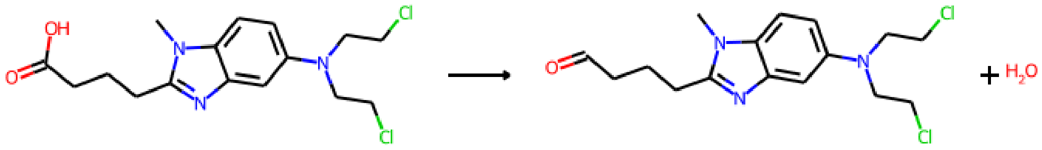


```
|Step  2|-----------------------------------------------------------------------------
-------------------------
Retrosynthesis:
Cn1c(CCCC=O)nc2cc(N(CCCl)CCCl)ccc21>>COC(=O)CCCc1nc2cc(N(CCCl)CCCl)ccc2n1C
Reaction: COC(=O)CCCc1nc2cc(N(CCCl)CCCl)ccc2n1C>>Cn1c(CCCC=O)nc2cc(N(CCCl)CCCl)ccc21
```

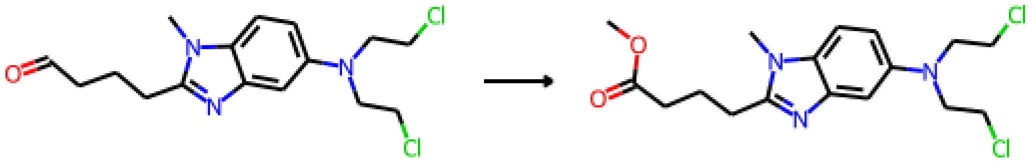


```
|Route 3|-----------------------------------------------------------------------------
-------------------------
|Step  1|-----------------------------------------------------------------------------
-------------------------
Retrosynthesis: CC(C)N(CC[C@H](c1ccccc1)c1cc(CO)ccc1O)C(C)C>>CC(C)N(CC[C@H]
(c1ccccc1)c1cc(CO)ccc1O[Si](C)(C)C(C)(C)C)C(C)C
Reaction: CC(C)N(CC[C@H](c1ccccc1)c1cc(CO)ccc1O[Si](C)(C)C(C)
(C)C)C(C)C>>CC(C)N(CC[C@H](c1ccccc1)c1cc(CO)ccc1O)C(C)C
```

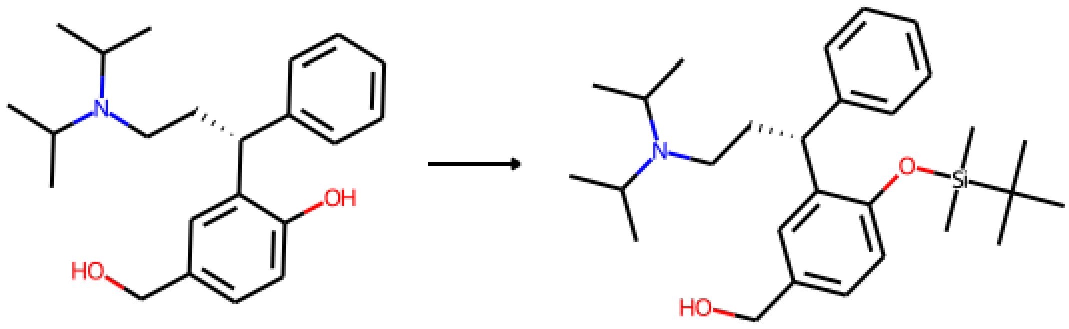

```
|Route 4|-------------------------------------------------------------------------------------------------------
|Step  1|-------------------------------------------------------------------------------------------------------
Retrosynthesis: N=C(NC(=N)N1CCCC1)Nc1ccc(OC(F)(F)F)cc1>>N#CNc1ccc(OC(F)(F)F)cc1.N=C(N)N1CCCC1
Reaction: N#CNc1ccc(OC(F)(F)F)cc1.N=C(N)N1CCCC1>>N=C(NC(=N)N1CCCC1)Nc1ccc(OC(F)(F)F)cc1
```

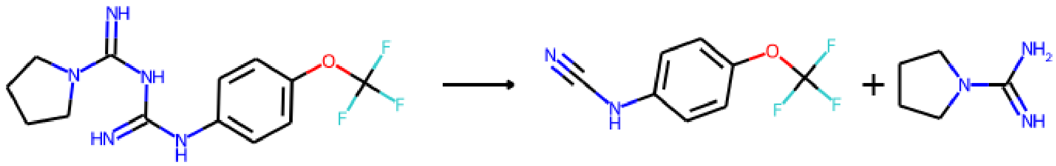

```
|Route 5|-------------------------------------------------------------------------------------------------------
|Step  1|-------------------------------------------------------------------------------------------------------
Retrosynthesis: Cn1cc(-c2ccc(S(=O)(=O)[C@@H]3C[C@@H](C(=O)NC4(C#N)CC4)N(C(=O)C4(C(F)(F)F)CC4)C3)c(C(F)(F)F)c2)cn1>>Cn1cc(B2OC(C)(C)C(C)(C)O2)cn1.N#CC1(NC(=O)[C@@H]2C[C@@H](S(=O)(=O)c3ccc(Cl)cc3C(F)(F)F)CN2C(=O)C2(C(F)(F)F)CC2)CC1
Reaction: Cn1cc(B2OC(C)(C)C(C)(C)O2)cn1.N#CC1(NC(=O)[C@@H]2C[C@@H](S(=O)(=O)c3ccc(Cl)cc3C(F)(F)F)CN2C(=O)C2(C(F)(F)F)CC2)CC1>>Cn1cc(-c2ccc(S(=O)(=O)[C@@H]3C[C@@H](C(=O)NC4(C#N)CC4)N(C(=O)C4(C(F)(F)F)CC4)C3)c(C(F)(F)F)c2)cn1
```

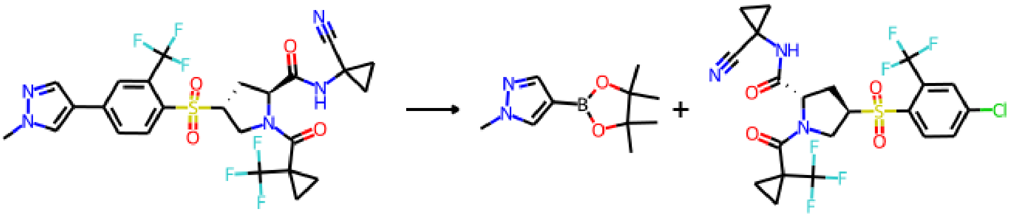

```
|Step  2|--------------------------------------------------------------------------
-------------------------
Retrosynthesis: N#CC1(NC(=O)[C@@H]2C[C@@H](S(=O)(=O)c3ccc(Cl)cc3C(F)
(F)F)CN2C(=O)C2(C(F)(F)F)CC2)CC1>>N#CC1(NC(=O)[C@@H]2C[C@@H](S(=O)
(=O)c3ccc(Cl)cc3C(F)(F)F)CN2)CC1.O=C(Cl)C1(C(F)(F)F)CC1
Reaction: N#CC1(NC(=O)[C@@H]2C[C@@H](S(=O)(=O)c3ccc(Cl)cc3C(F)
(F)F)CN2)CC1.O=C(Cl)C1(C(F)(F)F)CC1>>N#CC1(NC(=O)[C@@H]2C[C@@H](S(=O)
(=O)c3ccc(Cl)cc3C(F)(F)F)CN2C(=O)C2(C(F)(F)F)CC2)CC1
```

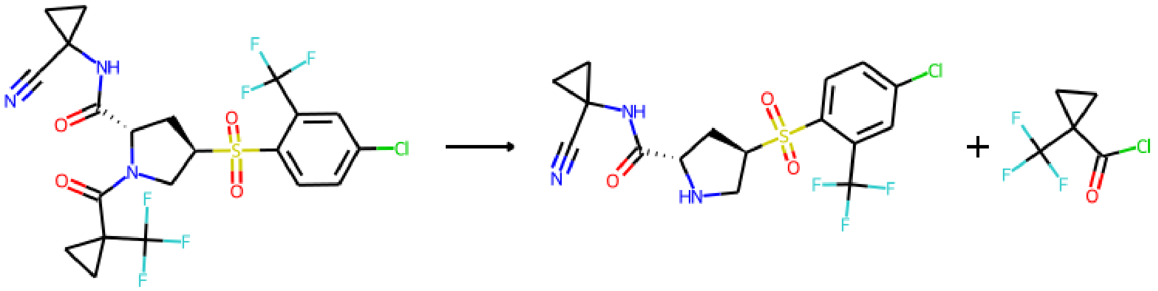

```
|Step  3|--------------------------------------------------------------------------
-------------------------
Retrosynthesis: N#CC1(NC(=O)[C@@H]2C[C@@H](S(=O)(=O)c3ccc(Cl)cc3C(F)
(F)F)CN2)CC1>>N#CC1(NC(=O)[C@@H]2C[C@@H](S(=O)(=O)c3ccc(Cl)cc3C(F)(F)F)CN2C(=O)C(F)
(F)F)CC1
Reaction: N#CC1(NC(=O)[C@@H]2C[C@@H](S(=O)(=O)c3ccc(Cl)cc3C(F)(F)F)CN2C(=O)C(F)
(F)F)CC1>>N#CC1(NC(=O)[C@@H]2C[C@@H](S(=O)(=O)c3ccc(Cl)cc3C(F)(F)F)CN2)CC1
```

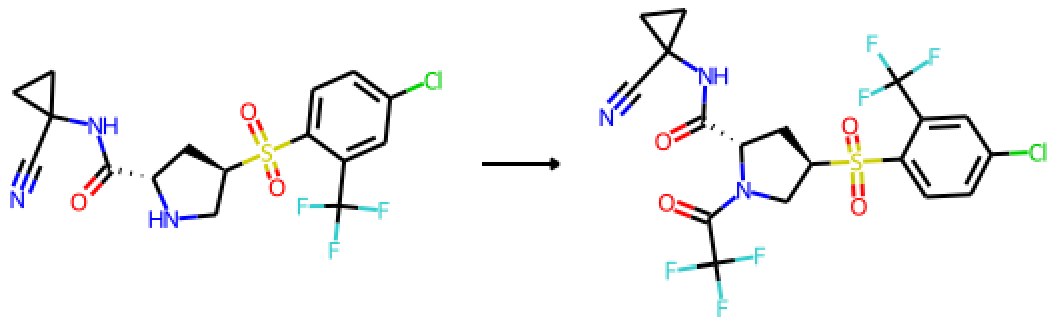

```
|Step  4|----------------------------------------------------------------------------
-------------------------
Retrosynthesis: N#CC1(NC(=O)[C@@H]2C[C@@H](S(=O)(=O)c3ccc(Cl)cc3C(F)
(F)F)CN2C(=O)C(F)(F)F)CC1>>O=C(O)[C@@H]1C[C@@H](S(=O)(=O)c2ccc(Cl)cc2C(F)
(F)F)CN1C(=O)C(F)(F)F.N#CC1(N)CC1
Reaction: O=C(O)[C@@H]1C[C@@H](S(=O)(=O)c2ccc(Cl)cc2C(F)(F)F)CN1C(=O)C(F)
(F)F.N#CC1(N)CC1>>N#CC1(NC(=O)[C@@H]2C[C@@H](S(=O)(=O)c3ccc(Cl)cc3C(F)
(F)F)CN2C(=O)C(F)(F)F)CC1
```

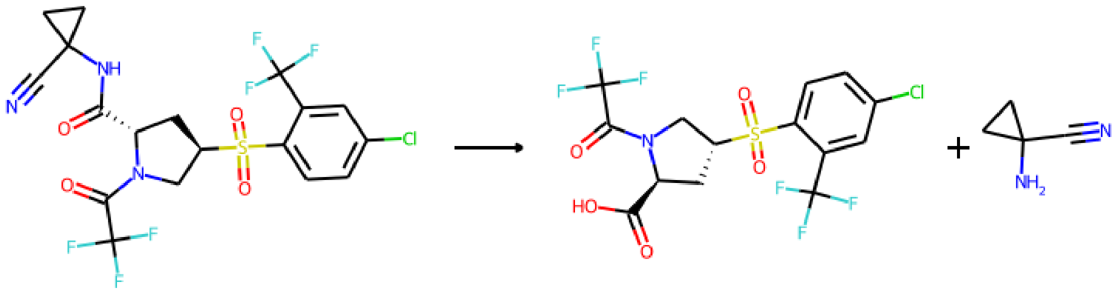

```
|Step  5|----------------------------------------------------------------------------
-------------------------
Retrosynthesis: O=C(O)[C@@H]1C[C@@H](S(=O)(=O)c2ccc(Cl)cc2C(F)(F)F)CN1C(=O)C(F)
(F)F>>O=S(=O)=O.FC(F)(F)c1cccc(Cl)c1.O=C(O)[C@@H]1CCCN1C(=O)C(F)(F)F
Reaction: O=S(=O)=O.FC(F)(F)c1cccc(Cl)c1.O=C(O)[C@@H]1CCCN1C(=O)C(F)(F)F>>O=C(O)
[C@@H]1C[C@@H](S(=O)(=O)c2ccc(Cl)cc2C(F)(F)F)CN1C(=O)C(F)(F)F
```

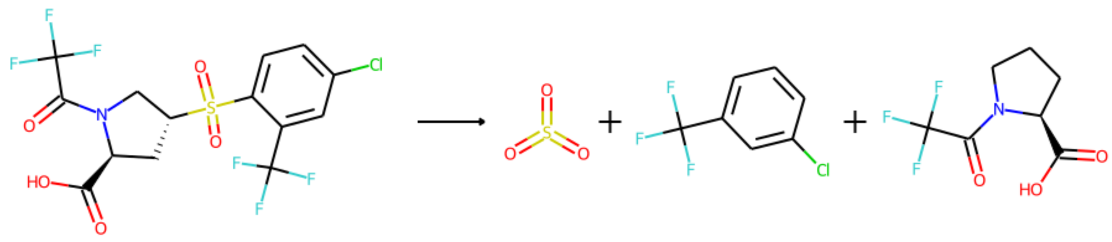


```
|Route 6|---------------------------------------------------------------------------
-------------------------
|Step  1|---------------------------------------------------------------------------
-------------------------
Retrosynthesis: COc1ccc2c3c1O[C@H]1C[C@@H]
(O)C=C[C@@]31CCN(C)C2>>COc1ccc2c3c1O[C@H]1CC(=O)C=C[C@@]31CCN(C)C2
Reaction: COc1ccc2c3c1O[C@H]1CC(=O)C=C[C@@]31CCN(C)C2>>COc1ccc2c3c1O[C@H]1C[C@@H]
(O)C=C[C@@]31CCN(C)C2
```

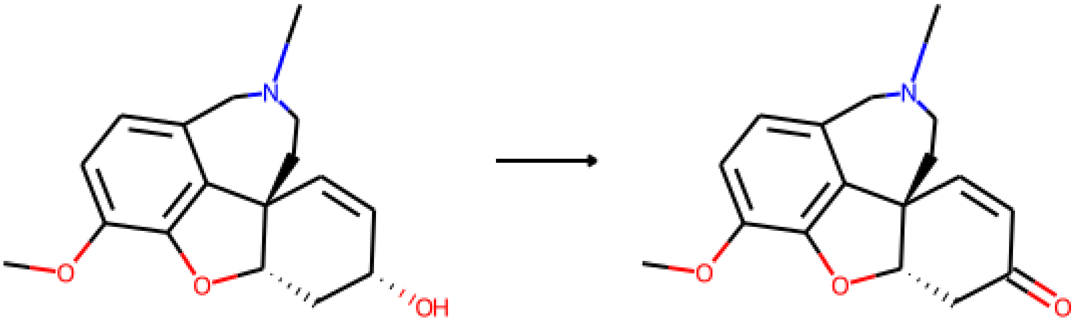


```
|Route 7|---------------------------------------------------------------------------
-------------------------
|Step  1|---------------------------------------------------------------------------
-------------------------
Retrosynthesis: CCC(=O)Oc1ccc2c(c1)CC[C@@H]1[C@@H]2CC[C@]2(C)[C@@H]
(OC(=O)CC)CC[C@@H]12>>C[C@]12CC[C@@H]3c4ccc(O)cc4CC[C@H]3[C@@H]1CC[C@@H]2O.CCC=O.C=C
OC(=O)CC
Reaction:
C[C@]12CC[C@@H]3c4ccc(O)cc4CC[C@H]3[C@@H]1CC[C@@H]2O.CCC=O.C=COC(=O)CC>>CCC(=O)Oc1cc
c2c(c1)CC[C@@H]1[C@@H]2CC[C@]2(C)[C@@H](OC(=O)CC)CC[C@@H]12
```

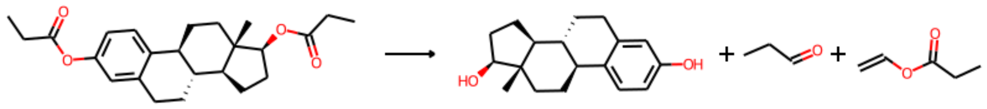

```
|Route 8|--------------------------------------------------------------
---------------------
|Step  1|--------------------------------------------------------------
---------------------
Retrosynthesis: CCCn1c(=O)c2nc([C@H]3C[C@H]4C[C@@H]3[C@@H]3O[C@H]43)
[nH]c2n(CCC)c1=O>>CCCn1c(N)c(NC(=O)
[C@H]2C[C@H]3C[C@@H]2[C@@H]2O[C@@H]23)c(=O)n(CCC)c1=O
Reaction: CCCn1c(N)c(NC(=O)
[C@H]2C[C@H]3C[C@@H]2[C@@H]2O[C@@H]23)c(=O)n(CCC)c1=O>>CCCn1c(=O)c2nc([C@H]3C[C@H]4C
[C@@H]3[C@@H]3O[C@H]43)[nH]c2n(CCC)c1=O
```

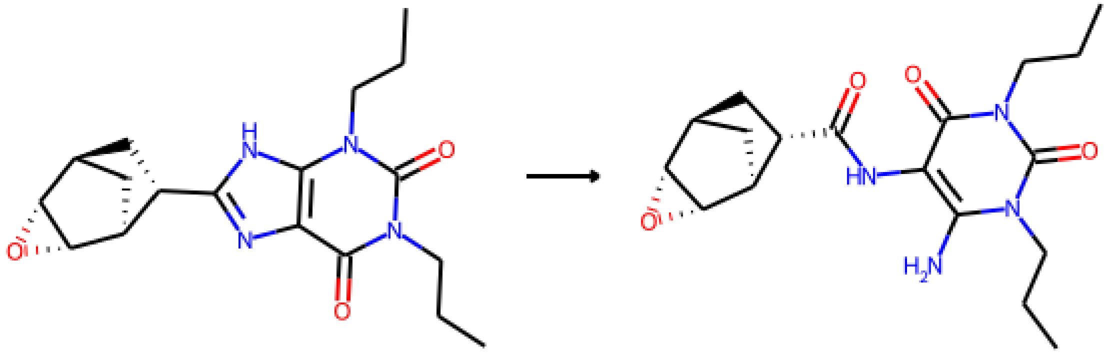

```
|Step  2|--------------------------------------------------------------
---------------------
Retrosynthesis: CCCn1c(N)c(NC(=O)
[C@H]2C[C@H]3C[C@@H]2[C@@H]2O[C@@H]23)c(=O)n(CCC)c1=O>>CCCn1c(N)c(N)c(=O)n(CCC)c1=O.
COC(=O)[C@H]1C[C@H]2C[C@@H]1[C@@H]1O[C@@H]12
Reaction: CCCn1c(N)c(N)c(=O)n(CCC)c1=O.COC(=O)
[C@H]1C[C@H]2C[C@@H]1[C@@H]1O[C@@H]12>>CCCn1c(N)c(NC(=O)
[C@H]2C[C@H]3C[C@@H]2[C@@H]2O[C@@H]23)c(=O)n(CCC)c1=O
```

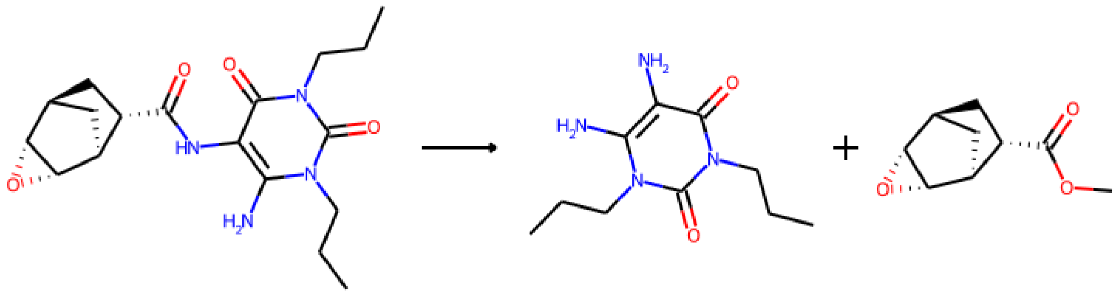


```
|Step  3|--------------------------------------------------------------------------------------------------------
Retrosynthesis: COC(=O)[C@H]1C[C@H]2C[C@@H]1[C@@H]1O[C@@H]12>>C1CO1.COC(=O)C1CCCC1
Reaction: C1CO1.COC(=O)C1CCCC1>>COC(=O)[C@H]1C[C@H]2C[C@@H]1[C@@H]1O[C@@H]12
```

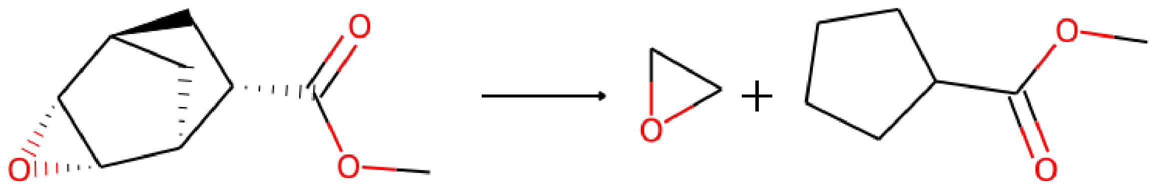


```
|Route 9|--------------------------------------------------------------------------------------------------------
|Step  1|--------------------------------------------------------------------------------------------------------
Retrosynthesis:
O=C1CCC(N2C(=O)c3ccccc3C2=O)C(=O)N1>>NC1CCC(=O)NC1=O.O=C1OC(=O)c2ccccc21
Reaction: NC1CCC(=O)NC1=O.O=C1OC(=O)c2ccccc21>>O=C1CCC(N2C(=O)c3ccccc3C2=O)C(=O)N1
```

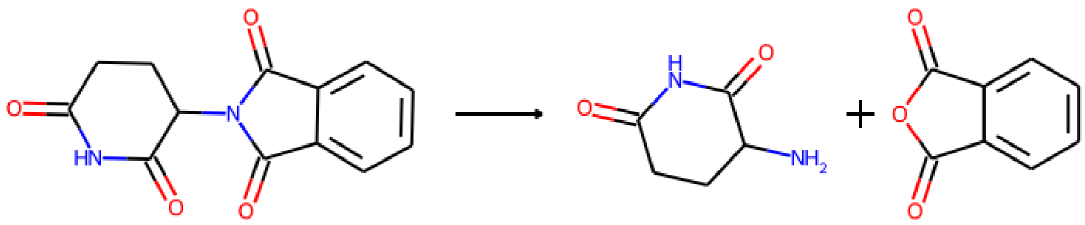

```
|Route 10|--------------------------------------------------------------------------------
--------------------------
|Step  1|---------------------------------------------------------------------------------
-------------------------
Retrosynthesis: CC1(NC2=NS(=O)(=O)c3sc(Cl)cc3N2)CC1>>COC(Nc1cc(Cl)sc1S(N)
(=O)=O)NC1(C)CC1
Reaction: COC(Nc1cc(Cl)sc1S(N)(=O)=O)NC1(C)CC1>>CC1(NC2=NS(=O)(=O)c3sc(Cl)cc3N2)CC1
```

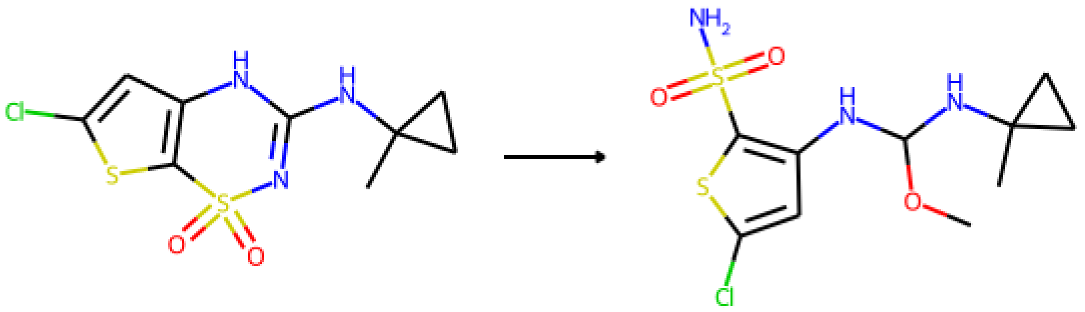

```
|Step  2|---------------------------------------------------------------------------------
-------------------------
Retrosynthesis: COC(Nc1cc(Cl)sc1S(N)(=O)=O)NC1(C)CC1>>CC1(NC#N)CC1.CO.NS(=O)
(=O)c1ccc(Cl)s1
Reaction: CC1(NC#N)CC1.CO.NS(=O)(=O)c1ccc(Cl)s1>>COC(Nc1cc(Cl)sc1S(N)
(=O)=O)NC1(C)CC1
```

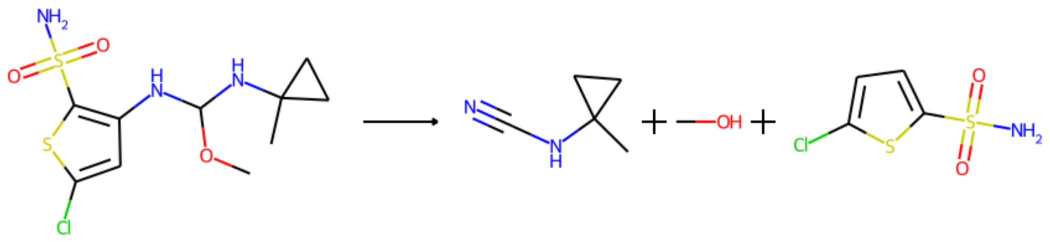

```
|Route 11|--------------------------------------------------------------------------------------------------------
|Step  1|--------------------------------------------------------------------------------------------------------
Retrosynthesis: CNC(C)CC1CCCCC1>>CC(=O)CC1CCCCC1.CN
Reaction: CC(=O)CC1CCCCC1.CN>>CNC(C)CC1CCCCC1
```

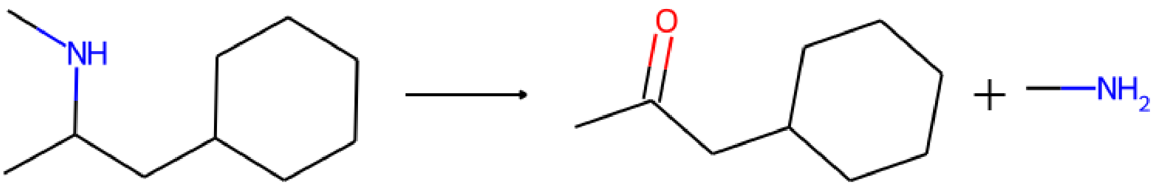

```
|Route 12|--------------------------------------------------------------------------------------------------------
|Step  1|--------------------------------------------------------------------------------------------------------
Retrosynthesis:
CCN(CC)CCOC(=O)C(c1ccccc1)C1CCCCC1>>O=C(O)C(c1ccccc1)C1CCCCC1.CCN(CC)CC
Reaction: O=C(O)C(c1ccccc1)C1CCCCC1.CCN(CC)CC>>CCN(CC)CCOC(=O)C(c1ccccc1)C1CCCCC1
```

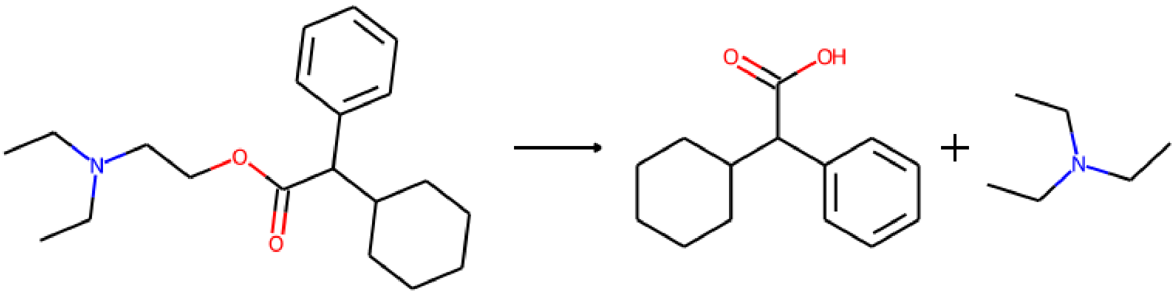

```
|Route 13|--------------------------------------------------------------------------------------------------------
|Step  1|--------------------------------------------------------------------------------------------------------
Retrosynthesis: C=C(CN(C)C)C(Cc1ccccc1)(OC(=O)CC)c1ccccc1>>C=C(CN(C)C)C(O)(Cc1ccccc1)c1ccccc1.CCC=O
Reaction: C=C(CN(C)C)C(O)(Cc1ccccc1)c1ccccc1.CCC=O>>C=C(CN(C)C)C(Cc1ccccc1)(OC(=O)CC)c1ccccc1
```

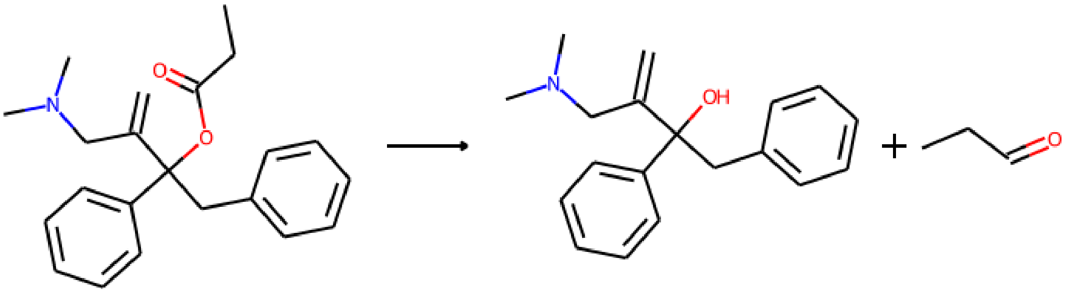

```
|Step  2|--------------------------------------------------------------------------------------------------------
Retrosynthesis: C=C(CN(C)C)C(O)(Cc1ccccc1)c1ccccc1>>CC(CN(C)C)C(=O)c1ccccc1.ClCc1ccccc1
Reaction: CC(CN(C)C)C(=O)c1ccccc1.ClCc1ccccc1>>C=C(CN(C)C)C(O)(Cc1ccccc1)c1ccccc1
```

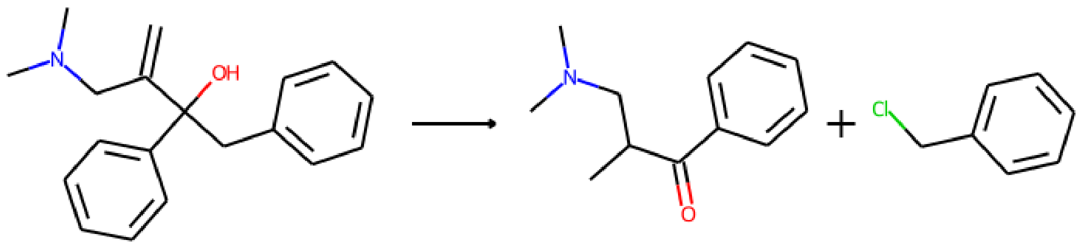


```
|Route 14|------------------------------------------------------------------------------------------------------
|Step  1|-----------------------------------------------------------------------------------------------------
Retrosynthesis: C=CCC1(C(C)CCC)C(=O)N=C(S)NC1=O>>C=CCC(C(Br)Br)(C(C)CCC)C(O)(O)N=C(N)S.O
Reaction: C=CCC(C(Br)Br)(C(C)CCC)C(O)(O)N=C(N)S.O>>C=CCC1(C(C)CCC)C(=O)N=C(S)NC1=O
```

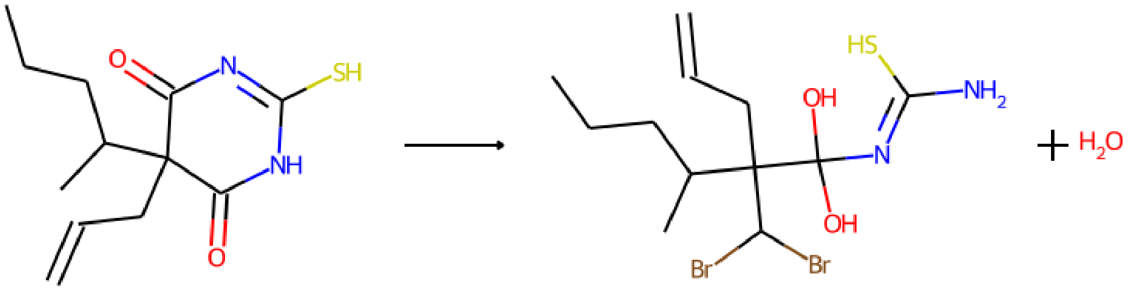


```
|Step  2|-----------------------------------------------------------------------------------------------------
Retrosynthesis: C=CCC(C(Br)Br)(C(C)CCC)C(O)(O)N=C(N)S>>BrC(Br)Br.C=CCCC(C)CCC.OCO.N=C(N)S
Reaction: BrC(Br)Br.C=CCCC(C)CCC.OCO.N=C(N)S>>C=CCC(C(Br)Br)(C(C)CCC)C(O)(O)N=C(N)S
```

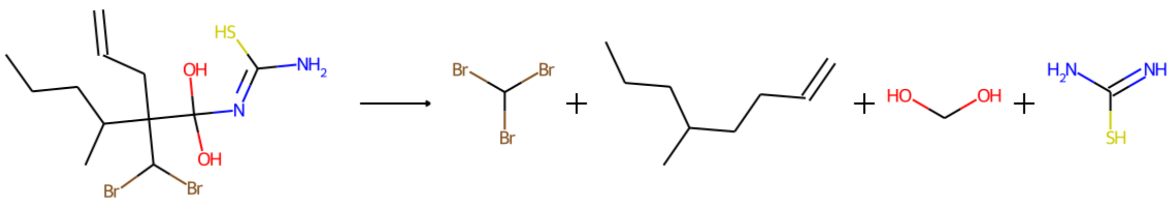

```
|Route 15|--------------------------------------------------------------------------
--------------------------
|Step  1|--------------------------------------------------------------------------
-------------------------
Retrosynthesis: CC(C)(C)NCC(O)COc1ccccc1OCC1CCCO1>>CC(C)
(C)N.ClCC1CCCO1.CC(O)COc1ccccc1O
Reaction: CC(C)(C)N.ClCC1CCCO1.CC(O)COc1ccccc1O>>CC(C)(C)NCC(O)COc1ccccc1OCC1CCCO1
```

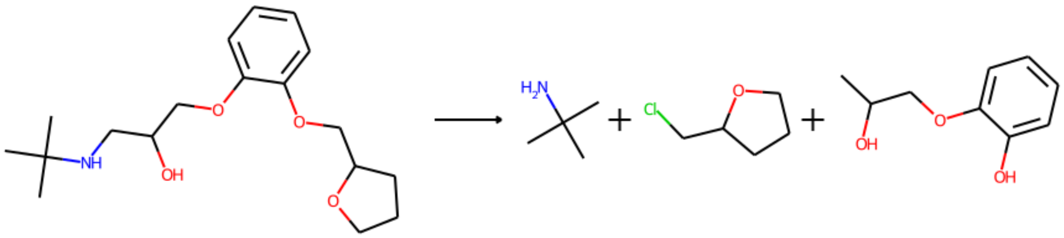


```
|Route 16|--------------------------------------------------------------------------
--------------------------
|Step  1|--------------------------------------------------------------------------
-------------------------
Retrosynthesis: CN1CCC(COC(=O)C(O)(c2ccccc2)c2ccccc2)C1>>CCOC(=O)C(O)
(c1ccccc1)c1ccccc1.CN1CCC(CO)C1
Reaction: CCOC(=O)C(O)(c1ccccc1)c1ccccc1.CN1CCC(CO)C1>>CN1CCC(COC(=O)C(O)
(c2ccccc2)c2ccccc2)C1
```

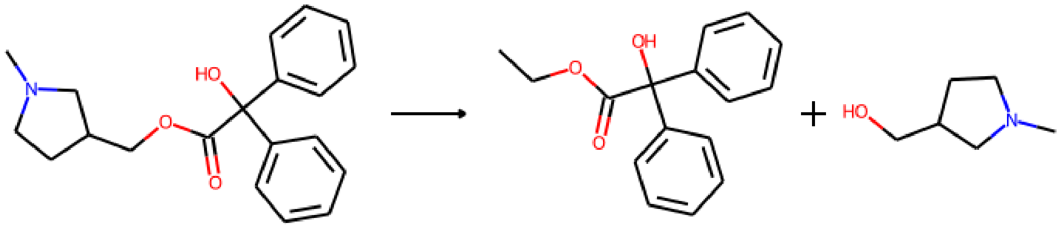

```
|Route 17|--------------------------------------------------------------------------------
--------------------------
|Step  1|---------------------------------------------------------------------------------
-------------------------
Retrosynthesis: COc1cc(N)c(Cl)cc1C(=O)N1CCN(Cc2ccc3c(c2)OCO3)CC1>>COc1cc(NC(=O)C(F)
(F)F)c(Cl)cc1C(=O)N1CCN(Cc2ccc3c(c2)OCO3)CC1
Reaction: COc1cc(NC(=O)C(F)
(F)F)c(Cl)cc1C(=O)N1CCN(Cc2ccc3c(c2)OCO3)CC1>>COc1cc(N)c(Cl)cc1C(=O)N1CCN(Cc2ccc3c(c
2)OCO3)CC1
```

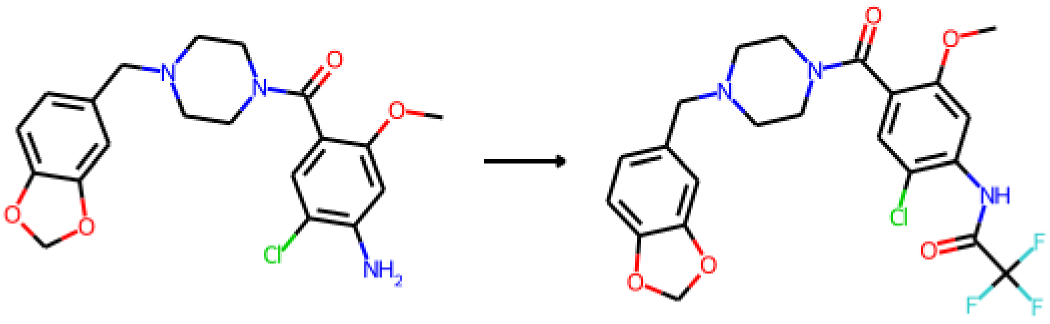

```
|Step  2|---------------------------------------------------------------------------------
-------------------------
Retrosynthesis: COc1cc(NC(=O)C(F)
(F)F)c(Cl)cc1C(=O)N1CCN(Cc2ccc3c(c2)OCO3)CC1>>COc1cc(NC(=O)C(F)
(F)F)c(Cl)cc1C(=O)N1CCNCC1.BrCc1ccc2c(c1)OCO2
Reaction: COc1cc(NC(=O)C(F)
(F)F)c(Cl)cc1C(=O)N1CCNCC1.BrCc1ccc2c(c1)OCO2>>COc1cc(NC(=O)C(F)
(F)F)c(Cl)cc1C(=O)N1CCN(Cc2ccc3c(c2)OCO3)CC1
```

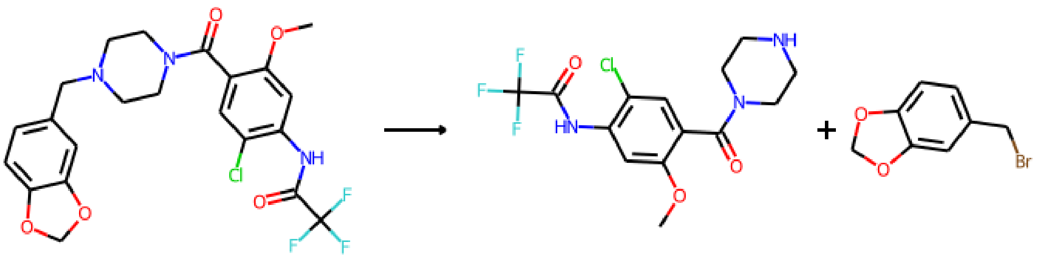

```
|Step  3|-------------------------------------------------------------------------------------------------------
Retrosynthesis: COc1cc(NC(=O)C(F)
(F)F)c(Cl)cc1C(=O)N1CCNCC1>>C1CNCCN1.COC(=O)c1cc(Cl)c(NC(=O)C(F)(F)F)cc1OC
Reaction: C1CNCCN1.COC(=O)c1cc(Cl)c(NC(=O)C(F)(F)F)cc1OC>>COc1cc(NC(=O)C(F)
(F)F)c(Cl)cc1C(=O)N1CCNCC1
```

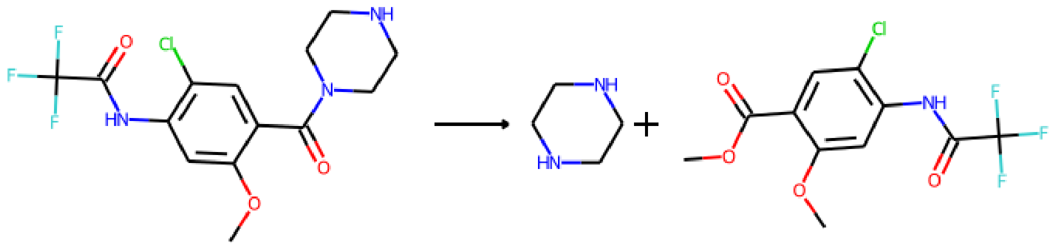

```
|Route 18|--------------------------------------------------------------------------------------------------------
|Step  1|-------------------------------------------------------------------------------------------------------
Retrosynthesis:
O=C(c1ccc(CN2CCOCC2)cc1)N1CCN(C2CC2)CC1>>Cc1ccc(C(=O)N2CCN(C3CC3)CC2)cc1.C1COCCN1
Reaction:
Cc1ccc(C(=O)N2CCN(C3CC3)CC2)cc1.C1COCCN1>>O=C(c1ccc(CN2CCOCC2)cc1)N1CCN(C2CC2)CC1
```

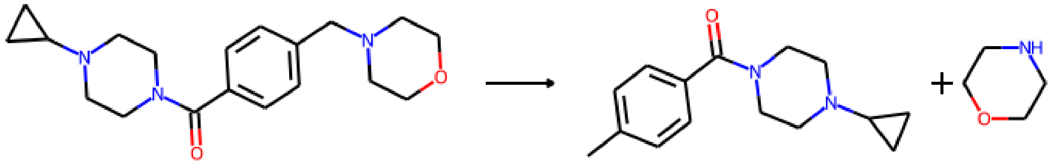

```
|Route 19|--------------------------------------------------------------------------
--------------------------
|Step  1|---------------------------------------------------------------------------
-------------------------
Retrosynthesis:
COc1cc2nc(N3CCN(C(=O)C4COc5ccccc5O4)CC3)nc(N)c2cc1OC>>COc1cc2nc(N3CCNCC3)nc(N)c2cc1O
C.CCOC(=O)C1COc2ccccc2O1
Reaction:
COc1cc2nc(N3CCNCC3)nc(N)c2cc1OC.CCOC(=O)C1COc2ccccc2O1>>COc1cc2nc(N3CCN(C(=O)C4COc5c
cccc5O4)CC3)nc(N)c2cc1OC
```

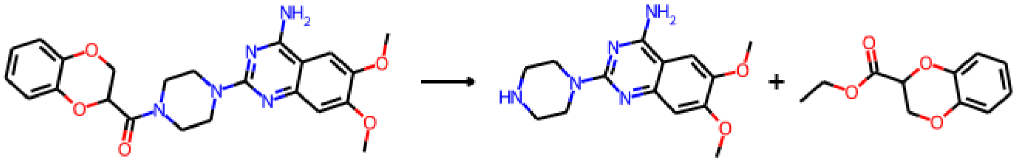

```
|Route 20|--------------------------------------------------------------------------
--------------------------
|Step  1|---------------------------------------------------------------------------
-------------------------
Retrosynthesis: Cn1cc2c(NC3=NCCN3)cccc2n1>>C.Cn1cc2c(N)cccc2n1.NCCNS(=O)(=O)O
Reaction: C.Cn1cc2c(N)cccc2n1.NCCNS(=O)(=O)O>>Cn1cc2c(NC3=NCCN3)cccc2n1
```

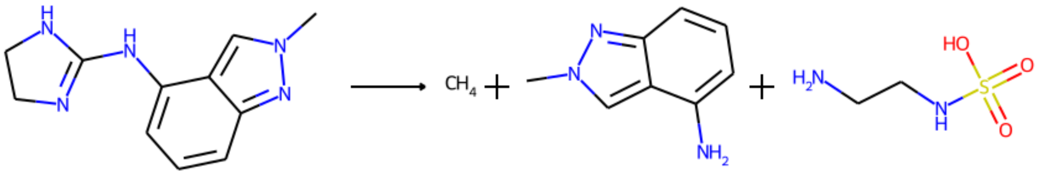

```
|Step  2|-------------------------------------------------------------------------
-------------------------
Retrosynthesis: NCCNS(=O)(=O)O>>O=[SH](=O)O.CNCCN
Reaction: O=[SH](=O)O.CNCCN>>NCCNS(=O)(=O)O
```

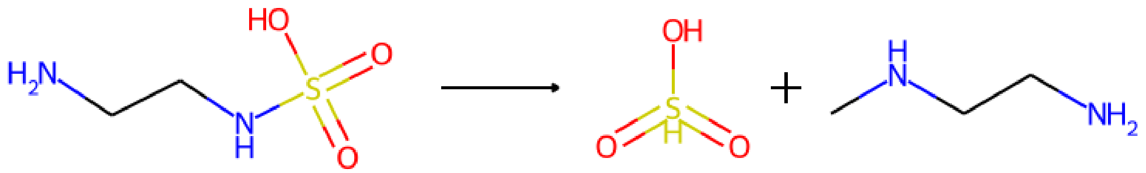

```
|Route 21|------------------------------------------------------------------------
--------------------------
|Step  1|-------------------------------------------------------------------------
-------------------------
Retrosynthesis: CC(C)(C)NCC(O)COc1cccc2c1CCC(=O)N2>>CC(C)
(C)N.O=C1CCc2c(cccc2OCC(O)CCl)N1
Reaction: CC(C)(C)N.O=C1CCc2c(cccc2OCC(O)CCl)N1>>CC(C)(C)NCC(O)COc1cccc2c1CCC(=O)N2
```

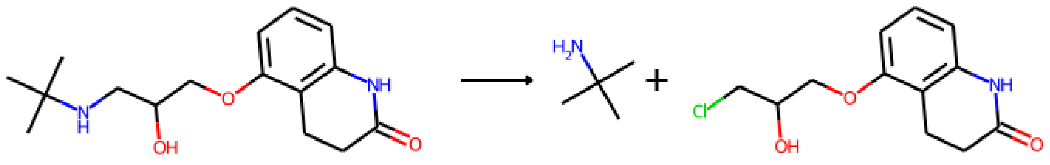

```
|Step  2|-------------------------------------------------------------------------
-------------------------
Retrosynthesis: O=C1CCc2c(cccc2OCC(O)CCl)N1>>OC(CCl)CCl.O=C1CCc2c(O)cccc2N1
Reaction: OC(CCl)CCl.O=C1CCc2c(O)cccc2N1>>O=C1CCc2c(cccc2OCC(O)CCl)N1
```

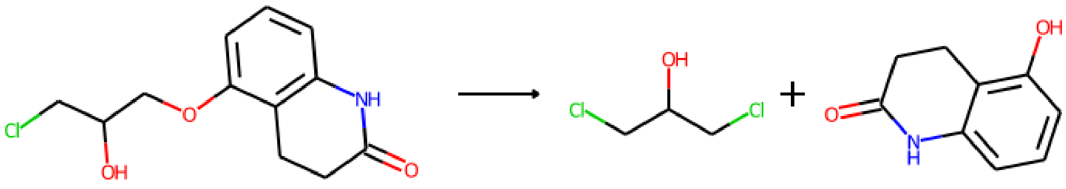


```
|Route 22|-----------------------------------------------------------------------------------------------------
|Step  1|------------------------------------------------------------------------------------------------------
Retrosynthesis:
CCOC(=O)c1[nH]c2cc(OC)c(OC)cc2c1CCN1CCN(c2ccccc2)CC1>>CCOC(=O)c1[nH]c2cc(OC)c(OC)cc2c1CC=O.c1ccc(N2CCNCC2)cc1
Reaction:
CCOC(=O)c1[nH]c2cc(OC)c(OC)cc2c1CC=O.c1ccc(N2CCNCC2)cc1>>CCOC(=O)c1[nH]c2cc(OC)c(OC)cc2c1CCN1CCN(c2ccccc2)CC1
```

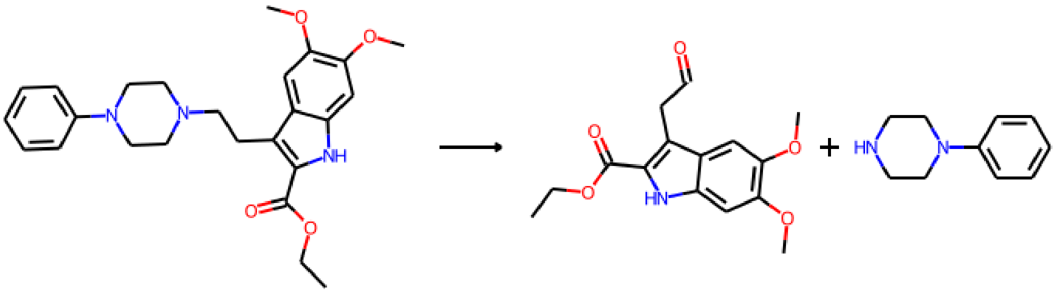


```
|Step  2|------------------------------------------------------------------------------------------------------
Retrosynthesis: CCOC(=O)c1[nH]c2cc(OC)c(OC)cc2c1CC=O>>CCOC(=O)Cc1c(C(=O)OCC)[nH]c2cc(OC)c(OC)cc12
Reaction: CCOC(=O)Cc1c(C(=O)OCC)[nH]c2cc(OC)c(OC)cc12>>CCOC(=O)c1[nH]c2cc(OC)c(OC)cc2c1CC=O
```

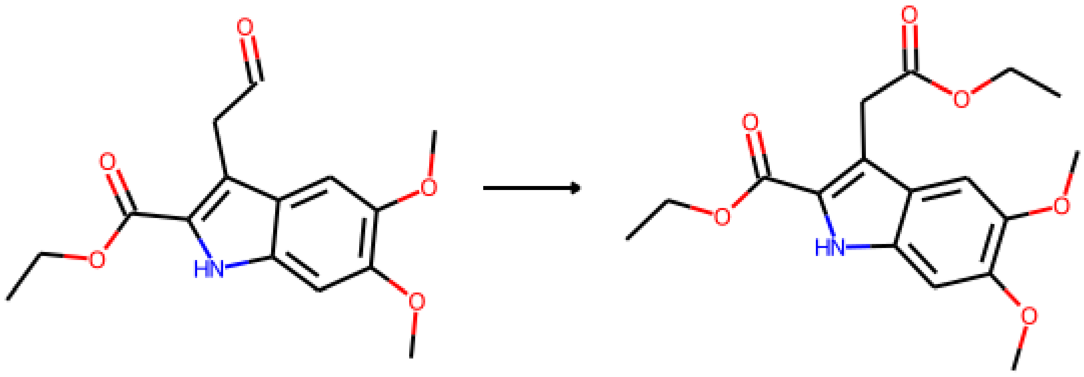

```
|Step  3|-------------------------------------------------------------------------
-------------------------
Retrosynthesis: CCOC(=O)Cc1c(C(=O)OCC)
[nH]c2cc(OC)c(OC)cc12>>COc1ccc(N)cc1OC.CCOC(=O)CC(=O)CC(=O)OCC
Reaction: COc1ccc(N)cc1OC.CCOC(=O)CC(=O)CC(=O)OCC>>CCOC(=O)Cc1c(C(=O)OCC)
[nH]c2cc(OC)c(OC)cc12
```

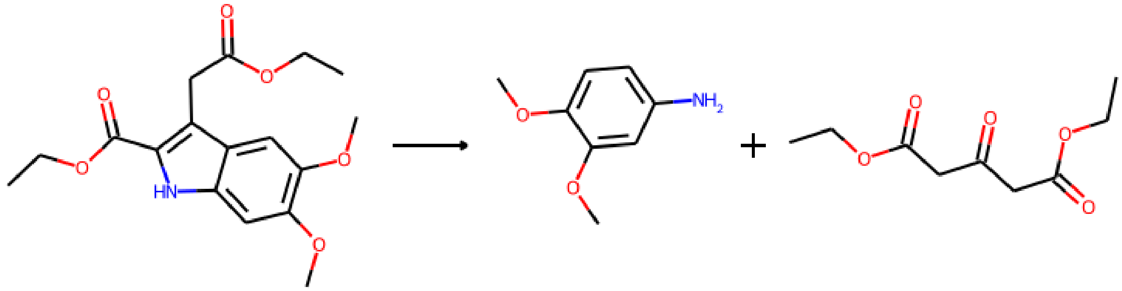


```
|Route 23|------------------------------------------------------------------------
--------------------------
|Step  1|-------------------------------------------------------------------------
-------------------------
Retrosynthesis: CN(C)CCc1c[nH]c2ccc(CS(=O)
(=O)N3CCCC3)cc12>>CN(C)CCC:C:N(C(=O)O)c1ccc(CS(=O)(=O)N2CCCC2)cc1
Reaction: CN(C)CCC:C:N(C(=O)O)c1ccc(CS(=O)
(=O)N2CCCC2)cc1>>CN(C)CCc1c[nH]c2ccc(CS(=O)(=O)N3CCCC3)cc12
```

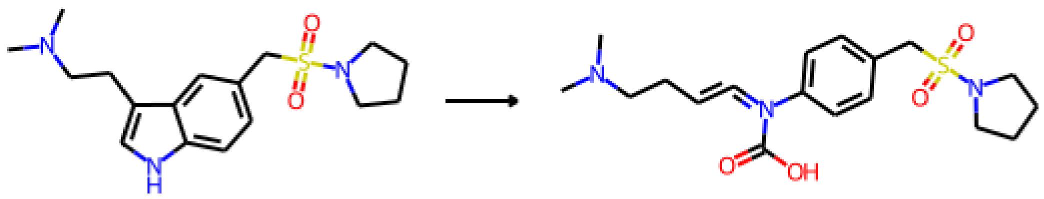

```
|Step  2|---------------------------------------------------------------------------------------------------
Retrosynthesis: CN(C)CCC:C:N(C(=O)O)c1ccc(CS(=O)
(=O)N2CCCC2)cc1>>CN(C)CCC:C:N(c1ccc(CS(=O)(=O)N2CCCC2)cc1)C(O)O
Reaction: CN(C)CCC:C:N(c1ccc(CS(=O)
(=O)N2CCCC2)cc1)C(O)O>>CN(C)CCC:C:N(C(=O)O)c1ccc(CS(=O)(=O)N2CCCC2)cc1
```

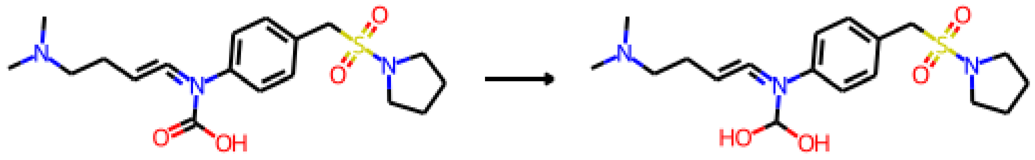

```
|Step  3|---------------------------------------------------------------------------------------------------
Retrosynthesis: CN(C)CCC:C:N(c1ccc(CS(=O)
(=O)N2CCCC2)cc1)C(O)O>>CCCCN(C)C.OCO.Nc1ccc(CS(=O)(=O)N2CCCC2)cc1
Reaction: CCCCN(C)C.OCO.Nc1ccc(CS(=O)(=O)N2CCCC2)cc1>>CN(C)CCC:C:N(c1ccc(CS(=O)
(=O)N2CCCC2)cc1)C(O)O
```

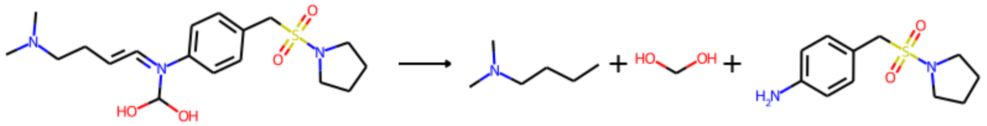


```
|Route 24|-----------------------------------------------------------------------------
--------------------------
|Step  1|------------------------------------------------------------------------------
-------------------------
Retrosynthesis: CCOP(=S)(OCC)O/N=C(\C#N)c1ccccc1>>N#C/C(=N\O)c1ccccc1.CCOP(=S)
(Cl)OCC
Reaction: N#C/C(=N\O)c1ccccc1.CCOP(=S)(Cl)OCC>>CCOP(=S)(OCC)O/N=C(\C#N)c1ccccc1
```

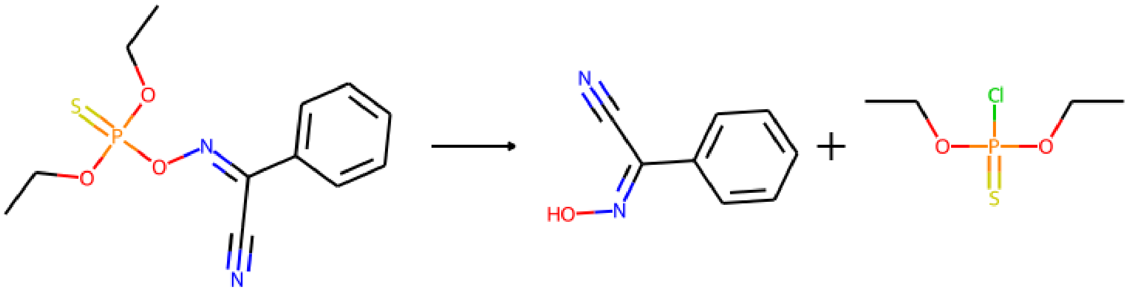


```
|Route 25|-----------------------------------------------------------------------------
--------------------------
|Step  1|------------------------------------------------------------------------------
-------------------------
Retrosynthesis: COc1ccc2c(C(=S)N(C)CC(=O)O)cccc2c1C(F)
(F)F>>O.COc1ccc2c(C(=S)N(C)CCOC(C)=O)cccc2c1C(F)(F)F
Reaction: O.COc1ccc2c(C(=S)N(C)CCOC(C)=O)cccc2c1C(F)
(F)F>>COc1ccc2c(C(=S)N(C)CC(=O)O)cccc2c1C(F)(F)F
```

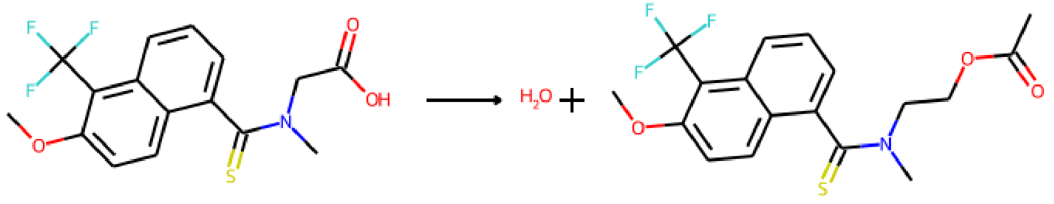

```
|Step  2|---------------------------------------------------------------------------
-------------------------
Retrosynthesis: COc1ccc2c(C(=S)N(C)CCOC(C)=O)cccc2c1C(F)
(F)F>>COc1ccc2c(C(=O)N(C)CCOC(C)=O)cccc2c1C(F)(F)F.COc1ccc(P2(=S)SP(=S)
(c3ccc(OC)cc3)S2)cc1
Reaction: COc1ccc2c(C(=O)N(C)CCOC(C)=O)cccc2c1C(F)(F)F.COc1ccc(P2(=S)SP(=S)
(c3ccc(OC)cc3)S2)cc1>>COc1ccc2c(C(=S)N(C)CCOC(C)=O)cccc2c1C(F)(F)F
```

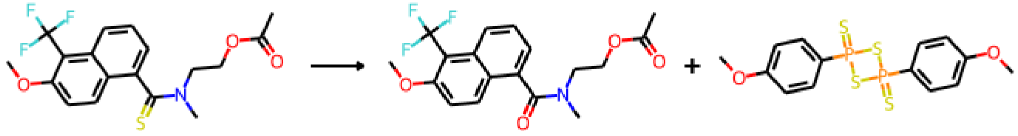

```
|Step  3|---------------------------------------------------------------------------
-------------------------
Retrosynthesis: COc1ccc2c(C(=O)N(C)CCOC(C)=O)cccc2c1C(F)
(F)F>>COc1ccc2c(C(=O)OC(C)=O)cccc2c1C(F)(F)F.CNCCOC(C)=O
Reaction: COc1ccc2c(C(=O)OC(C)=O)cccc2c1C(F)
(F)F.CNCCOC(C)=O>>COc1ccc2c(C(=O)N(C)CCOC(C)=O)cccc2c1C(F)(F)F
```

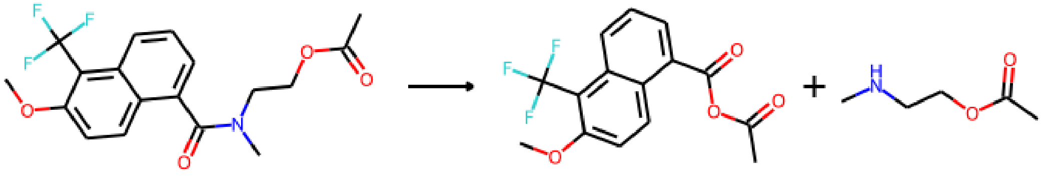


```
|Step  4|---------------------------------------------------------------------------
-------------------------
Retrosynthesis: COc1ccc2c(C(=O)OC(C)=O)cccc2c1C(F)(F)F>>COc1ccc2c(C(=O)O)cccc2c1C(F)
(F)F.CC(N)=O
Reaction: COc1ccc2c(C(=O)O)cccc2c1C(F)
(F)F.CC(N)=O>>COc1ccc2c(C(=O)OC(C)=O)cccc2c1C(F)(F)F
```

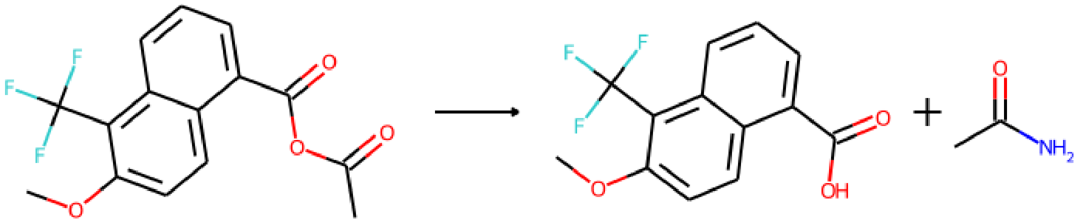


```
|Route 26|--------------------------------------------------------------------------
--------------------------
|Step  1|---------------------------------------------------------------------------
-------------------------
Retrosynthesis: Cc1c(CC(=O)O)sc2ccc(Cl)cc12>>CC(=O)c1sc2ccc(Cl)cc2c1C.O
Reaction: CC(=O)c1sc2ccc(Cl)cc2c1C.O>>Cc1c(CC(=O)O)sc2ccc(Cl)cc12
```

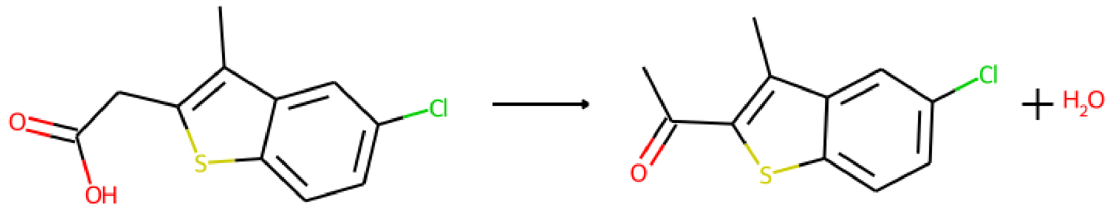


```
|Route 27|------------------------------------------------------------------------------
--------------------------
|Step  1|-------------------------------------------------------------------------------
-------------------------
Retrosynthesis:
CCCC1(CC(=O)O)OCCc2c1[nH]c1ccccc21>>CCCC1(CC(=O)O)OCCc2c1n(CC)c1ccccc21
Reaction: CCCC1(CC(=O)O)OCCc2c1n(CC)c1ccccc21>>CCCC1(CC(=O)O)OCCc2c1[nH]c1ccccc21
```

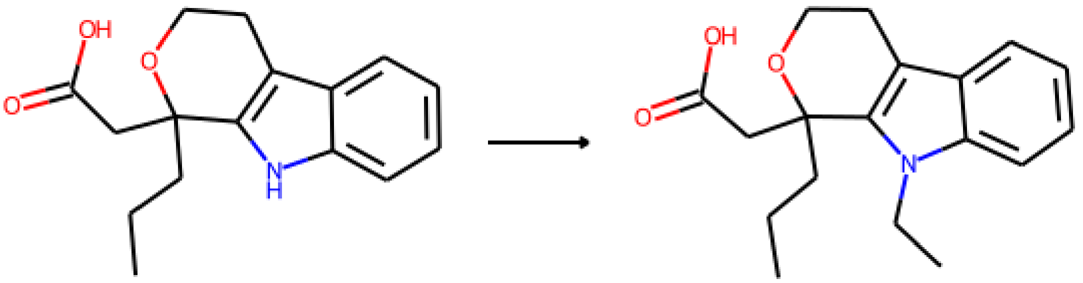


```
|Step  2|-------------------------------------------------------------------------------
-------------------------
Retrosynthesis:
CCCC1(CC(=O)O)OCCc2c1n(CC)c1ccccc21>>CCCCCC(=O)O.CCn1cc(CCO)c2ccccc21
Reaction: CCCCCC(=O)O.CCn1cc(CCO)c2ccccc21>>CCCC1(CC(=O)O)OCCc2c1n(CC)c1ccccc21
```

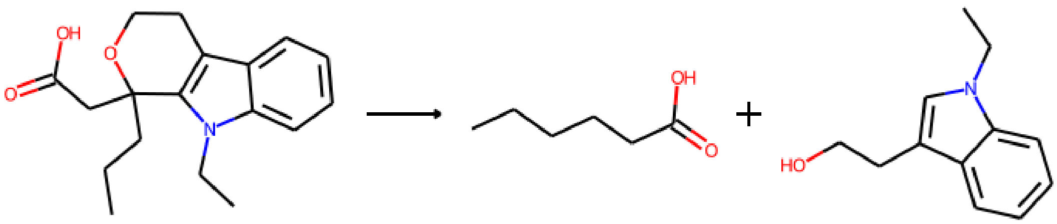

```
|Route 28|------------------------------------------------------------------------------------------------------
|Step  1|-----------------------------------------------------------------------------------------------------
Retrosynthesis: CCC(=O)NS(=O)(=O)c1cnccc1Nc1cccc(C(F)(F)F)c1>>CCC(=O)NS(=O)(=O)c1cnccc1Cl.Nc1cccc(C(F)(F)F)c1
Reaction: CCC(=O)NS(=O)(=O)c1cnccc1Cl.Nc1cccc(C(F)(F)F)c1>>CCC(=O)NS(=O)(=O)c1cnccc1Nc1cccc(C(F)(F)F)c1
```

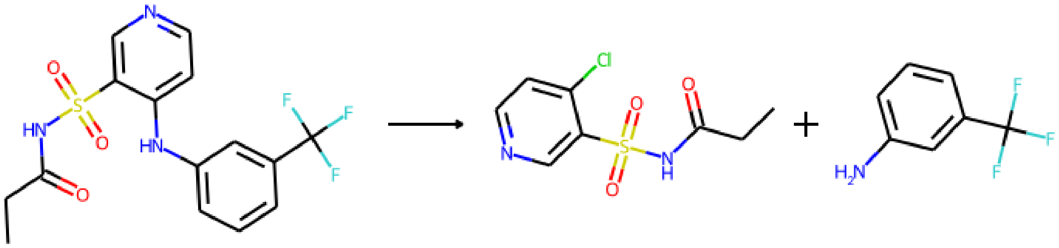

```
|Step  2|-----------------------------------------------------------------------------------------------------
Retrosynthesis: CCC(=O)NS(=O)(=O)c1cnccc1Cl>>CCC(=O)OC(C)=O.NS(=O)(=O)c1cnccc1Cl
Reaction: CCC(=O)OC(C)=O.NS(=O)(=O)c1cnccc1Cl>>CCC(=O)NS(=O)(=O)c1cnccc1Cl
```

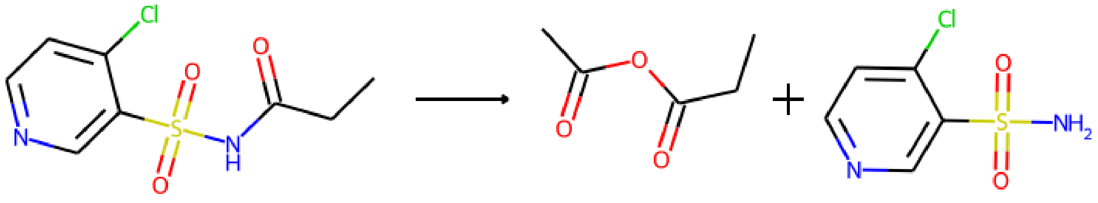

```
|Route 29|--------------------------------------------------------------------------
--------------------------
|Step  1|--------------------------------------------------------------------------
-------------------------
Retrosynthesis: C=CCCCCCCCCC(=O)O>>C#CCCCCCCCCC(=O)O
Reaction: C#CCCCCCCCCC(=O)O>>C=CCCCCCCCCC(=O)O
```

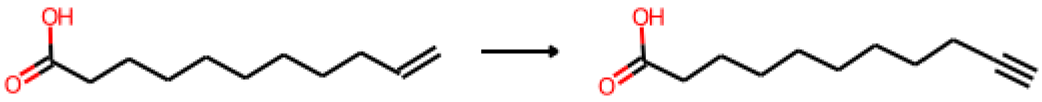

```
|Route 30|--------------------------------------------------------------------------
--------------------------
|Step  1|--------------------------------------------------------------------------
-------------------------
Retrosynthesis:
Cc1ccc(Cc2cnc(NCCCCc3ncc(Br)cc3C)nc2O)cn1>>Cc1ccc(Cc2cnc(C)nc2O)cn1.Cc1cc(Br)cnc1CCC
CN
Reaction:
Cc1ccc(Cc2cnc(C)nc2O)cn1.Cc1cc(Br)cnc1CCCCN>>Cc1ccc(Cc2cnc(NCCCCc3ncc(Br)cc3C)nc2O)c
n1
```

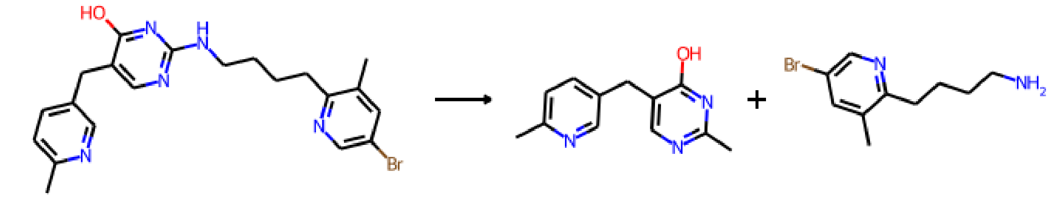

```
|Step  2|--------------------------------------------------------------------------
-------------------------
Retrosynthesis: Cc1ccc(Cc2cnc(C)nc2O)cn1>>Cc1nccc(O)n1.Cc1ccc(CCl)cn1
Reaction: Cc1nccc(O)n1.Cc1ccc(CCl)cn1>>Cc1ccc(Cc2cnc(C)nc2O)cn1
```

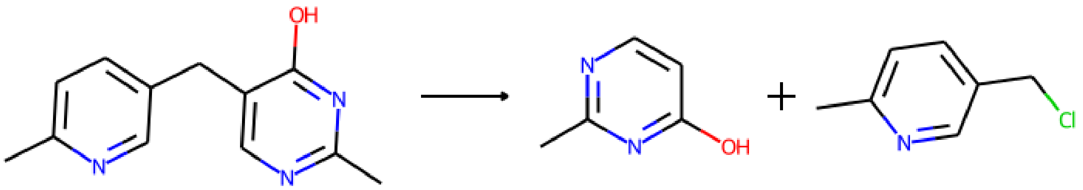

```
|Step  3|--------------------------------------------------------------------------
-------------------------
Retrosynthesis: Cc1cc(Br)cnc1CCCCN>>Br.Cc1cccnc1CCCCN
Reaction: Br.Cc1cccnc1CCCCN>>Cc1cc(Br)cnc1CCCCN
```

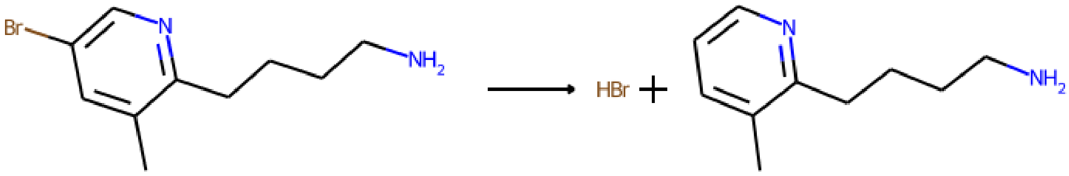

```
|Route 31|--------------------------------------------------------------------------------------------------------
|Step  1|--------------------------------------------------------------------------------------------------------
Retrosynthesis: 
O=c1[nH]c2ccccc2n1C1CCN(CCCc2noc3cc(F)ccc23)CC1>>O=c1[nH]c2ccccc2n1C1CCNCC1.Fc1ccc2c(CCCCl)noc2c1
Reaction: 
O=c1[nH]c2ccccc2n1C1CCNCC1.Fc1ccc2c(CCCCl)noc2c1>>O=c1[nH]c2ccccc2n1C1CCN(CCCc2noc3cc(F)ccc23)CC1
```

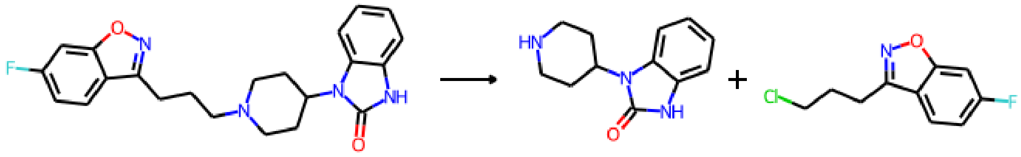

```
|Route 32|--------------------------------------------------------------------------------------------------------
|Step  1|--------------------------------------------------------------------------------------------------------
Retrosynthesis: CC(C)N1CCC(c2ccccc2)(c2ccccc2)CC1>>c1ccc(C2(c3ccccc3)CCNCC2)cc1.CC(C)=O
Reaction: c1ccc(C2(c3ccccc3)CCNCC2)cc1.CC(C)=O>>CC(C)N1CCC(c2ccccc2)(c2ccccc2)CC1
```

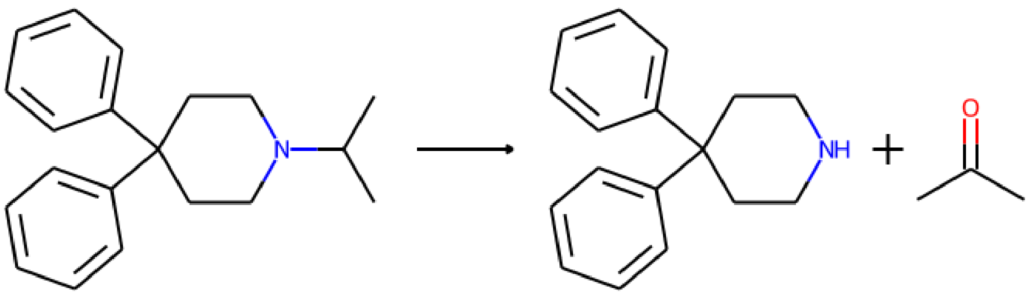

```
|Route 33|--------------------------------------------------------------------------
--------------------------
|Step  1|---------------------------------------------------------------------------
-------------------------
Retrosynthesis:
COC(=O)C1=C(C)NC(C)=C(C(=O)OC)C1c1ccccc1OC(F)F>>COC(=O)C=C(C)N.CC.COC(=O)CC(=O)c1ccc
cc1OC(F)F
Reaction:
COC(=O)C=C(C)N.CC.COC(=O)CC(=O)c1ccccc1OC(F)F>>COC(=O)C1=C(C)NC(C)=C(C(=O)OC)C1c1ccc
cc1OC(F)F
```

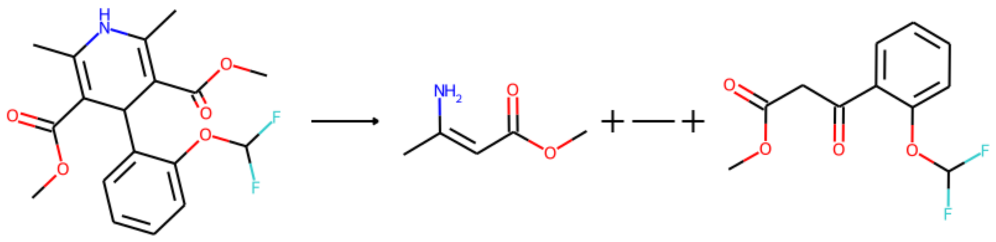

```
|Route 34|--------------------------------------------------------------------------
--------------------------
|Step  1|---------------------------------------------------------------------------
-------------------------
Retrosynthesis: CCON(CC)C(=S)S>>S.CCO.CCN=C=S
Reaction: S.CCO.CCN=C=S>>CCON(CC)C(=S)S
```

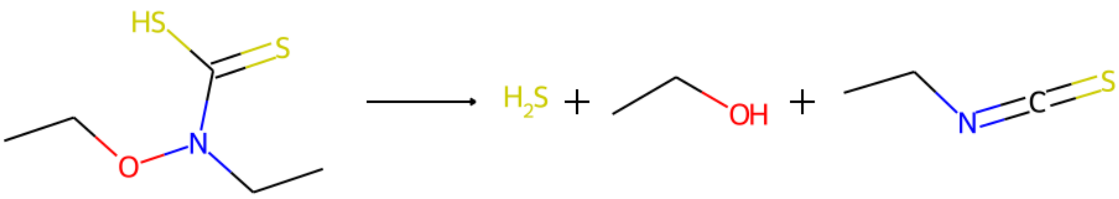

```
|Route 35|--------------------------------------------------------------------------
--------------------------
|Step  1|---------------------------------------------------------------------------
-------------------------
Retrosynthesis:
CC(=O)Oc1ccc(C(c2ccc(OC(C)=O)cc2)c2ccccn2)cc1>>Oc1ccc(C(c2ccc(O)cc2)c2ccccn2)cc1.CC(
=O)O
Reaction:
Oc1ccc(C(c2ccc(O)cc2)c2ccccn2)cc1.CC(=O)O>>CC(=O)Oc1ccc(C(c2ccc(OC(C)=O)cc2)c2ccccn2
)cc1
```

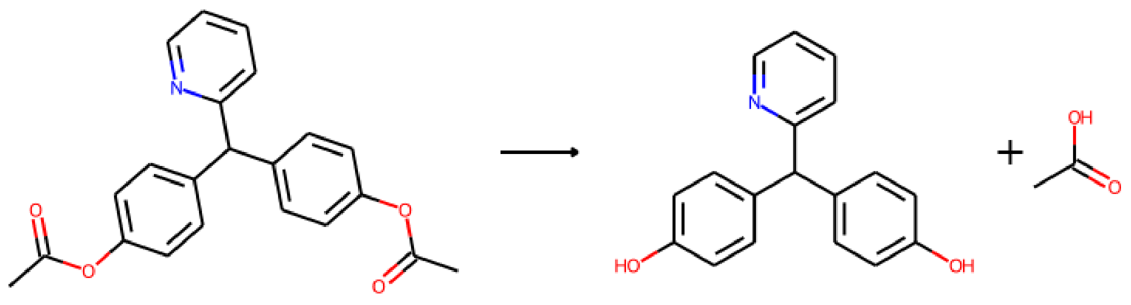

```
|Step  2|---------------------------------------------------------------------------
-------------------------
Retrosynthesis:
Oc1ccc(C(c2ccc(O)cc2)c2ccccn2)cc1>>Oc1ccc(C(c2ccc(OCc3ccccc3)cc2)c2ccccn2)cc1
Reaction:
Oc1ccc(C(c2ccc(OCc3ccccc3)cc2)c2ccccn2)cc1>>Oc1ccc(C(c2ccc(O)cc2)c2ccccn2)cc1
```

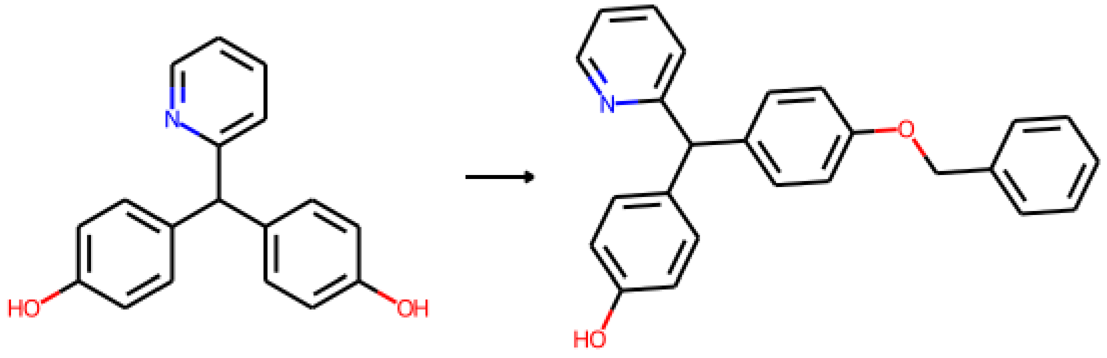

```
|Step  3|---------------------------------------------------------------------------
-------------------------
Retrosynthesis:
Oc1ccc(C(c2ccc(OCc3ccccc3)cc2)c2ccccn2)cc1>>Oc1ccc(Cc2ccc(OCc3ccccc3)cc2)cc1.c1ccncc
1
Reaction:
Oc1ccc(Cc2ccc(OCc3ccccc3)cc2)cc1.c1ccncc1>>Oc1ccc(C(c2ccc(OCc3ccccc3)cc2)c2ccccn2)cc
1
```

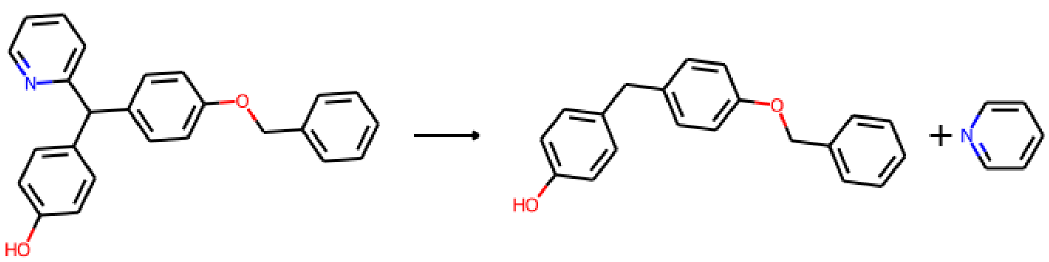

```
|Route 36|--------------------------------------------------------------------------
--------------------------
|Step  1|---------------------------------------------------------------------------
-------------------------
Retrosynthesis:
O=C(NOCCO)c1cc(CN2OCCCC2=O)c(F)c(F)c1Nc1ccc(I)cc1F>>COC(=O)CCCONCc1cc(C(=O)NOCCO)c(N
c2ccc(I)cc2F)c(F)c1F
Reaction:
COC(=O)CCCONCc1cc(C(=O)NOCCO)c(Nc2ccc(I)cc2F)c(F)c1F>>O=C(NOCCO)c1cc(CN2OCCCC2=O)c(F
)c(F)c1Nc1ccc(I)cc1F
```

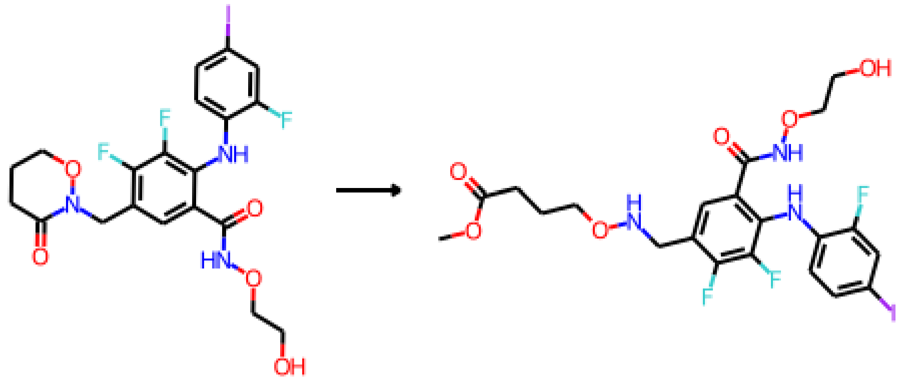

```
|Step  2|--------------------------------------------------------------------------
-------------------------
Retrosynthesis:
COC(=O)CCCONCc1cc(C(=O)NOCCO)c(Nc2ccc(I)cc2F)c(F)c1F>>COC(=O)CCCON=Cc1cc(C(=O)NOCCO)
c(Nc2ccc(I)cc2F)c(F)c1F
Reaction:
COC(=O)CCCON=Cc1cc(C(=O)NOCCO)c(Nc2ccc(I)cc2F)c(F)c1F>>COC(=O)CCCONCc1cc(C(=O)NOCCO)
c(Nc2ccc(I)cc2F)c(F)c1F
```

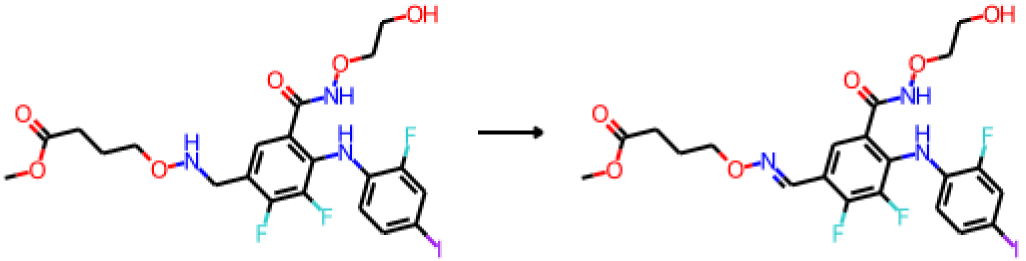

```
|Step  3|--------------------------------------------------------------------------
-------------------------
Retrosynthesis:
COC(=O)CCCON=Cc1cc(C(=O)NOCCO)c(Nc2ccc(I)cc2F)c(F)c1F>>O=Cc1cc(C(=O)NOCCO)c(Nc2ccc(I
)cc2F)c(F)c1F.COC(=O)CCCON
Reaction:
O=Cc1cc(C(=O)NOCCO)c(Nc2ccc(I)cc2F)c(F)c1F.COC(=O)CCCON>>COC(=O)CCCON=Cc1cc(C(=O)NOC
CO)c(Nc2ccc(I)cc2F)c(F)c1F
```

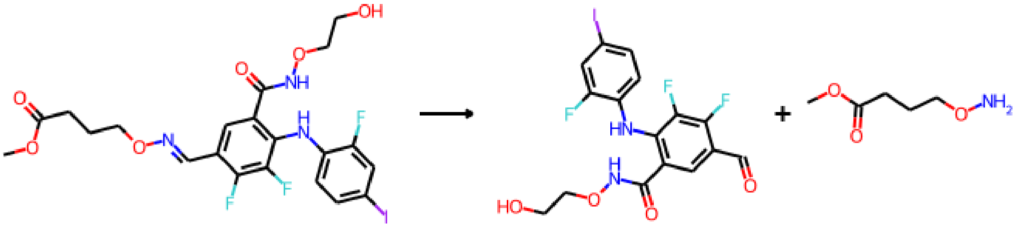

```
|Step  4|-------------------------------------------------------------------------------------------------------
Retrosynthesis:
O=Cc1cc(C(=O)NOCCO)c(Nc2ccc(I)cc2F)c(F)c1F>>O=Cc1cc(C(=O)O)c(Nc2ccc(I)cc2F)c(F)c1F.NOCCO
Reaction:
O=Cc1cc(C(=O)O)c(Nc2ccc(I)cc2F)c(F)c1F.NOCCO>>O=Cc1cc(C(=O)NOCCO)c(Nc2ccc(I)cc2F)c(F)c1F
```

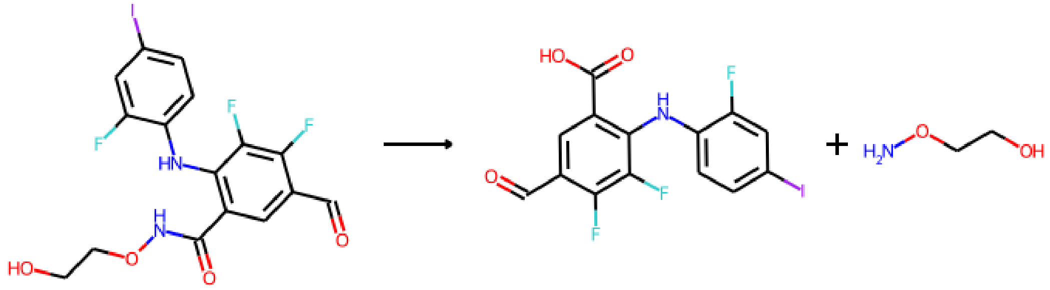

```
|Step  5|-------------------------------------------------------------------------------------------------------
Retrosynthesis:
O=Cc1cc(C(=O)O)c(Nc2ccc(I)cc2F)c(F)c1F>>O=Cc1cc(C(=O)O)c(F)c(F)c1F.Nc1ccc(I)cc1F
Reaction:
O=Cc1cc(C(=O)O)c(F)c(F)c1F.Nc1ccc(I)cc1F>>O=Cc1cc(C(=O)O)c(Nc2ccc(I)cc2F)c(F)c1F
```

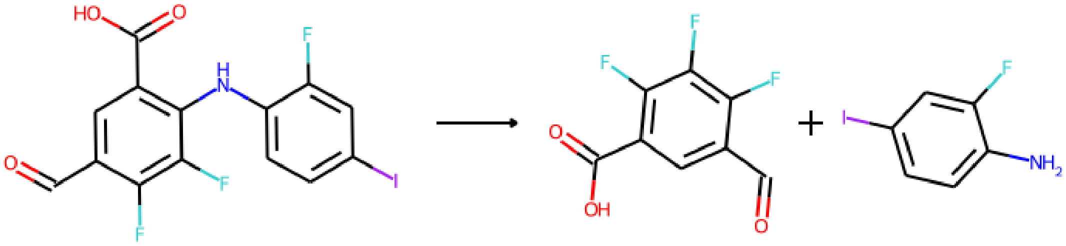

```
|Route 37|--------------------------------------------------------------------------
--------------------------
|Step  1|---------------------------------------------------------------------------
-------------------------
Retrosynthesis:
COc1ccc2[nH]cc(CCNC(=O)c3cc(=O)cco3)c2c1>>COC(=O)c1cc(=O)cco1.COc1ccc2[nH]cc(CCN)c2c
1
Reaction:
COC(=O)c1cc(=O)cco1.COc1ccc2[nH]cc(CCN)c2c1>>COc1ccc2[nH]cc(CCNC(=O)c3cc(=O)cco3)c2c
1
```

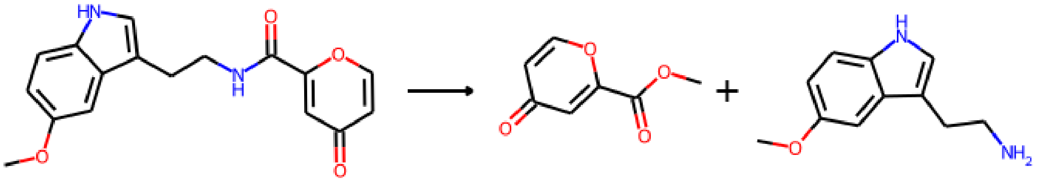

```
|Route 38|--------------------------------------------------------------------------
--------------------------
|Step  1|---------------------------------------------------------------------------
-------------------------
Retrosynthesis: CCCCN(CCN(CC)CC)C(=O)c1cccc2ccccc12>>CCCCN(CCN(CC)CC)C(O)
(ON1C(=O)CCC1=O)c1cccc2ccccc12
Reaction: CCCCN(CCN(CC)CC)C(O)
(ON1C(=O)CCC1=O)c1cccc2ccccc12>>CCCCN(CCN(CC)CC)C(=O)c1cccc2ccccc12
```

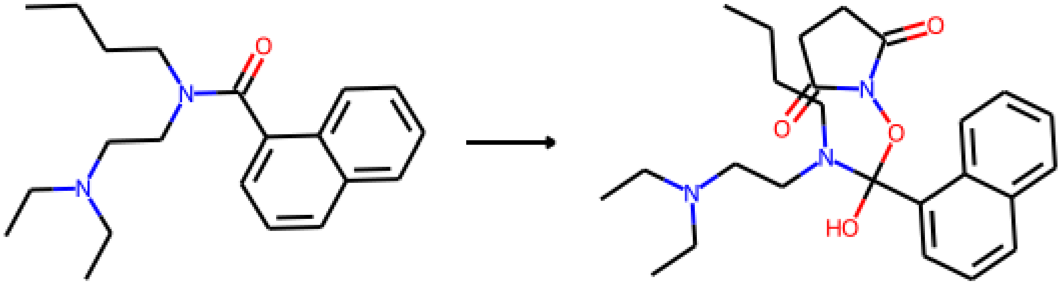

```
|Step  2|--------------------------------------------------------------------------------------------------------
Retrosynthesis: CCCCN(CCN(CC)CC)C(O)(ON1C(=O)CCC1=O)c1cccc2ccccc12>>O=C1CCC(=O)N1O.CCCC.CCN(CC)CCNC(=O)c1cccc2ccccc12
Reaction: O=C1CCC(=O)N1O.CCCC.CCN(CC)CCNC(=O)c1cccc2ccccc12>>CCCCN(CCN(CC)CC)C(O)(ON1C(=O)CCC1=O)c1cccc2ccccc12
```

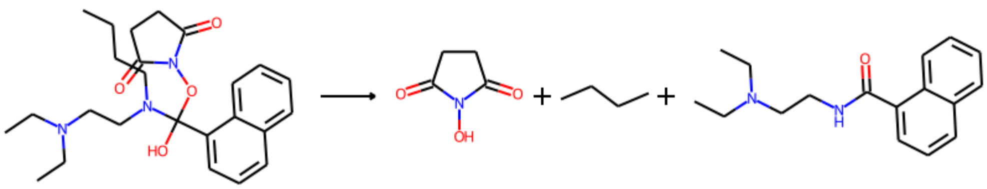

```
|Route 39|--------------------------------------------------------------------------------------------------------
|Step  1|--------------------------------------------------------------------------------------------------------
Retrosynthesis: CCOP(=O)(OCC)OP(=O)(OCC)OCC>>CCOP(=O)(OCC)OP(O)(OCC)(OCC)OS(=O)(=O)c1ccc(C)cc1
Reaction: CCOP(=O)(OCC)OP(O)(OCC)(OCC)OS(=O)(=O)c1ccc(C)cc1>>CCOP(=O)(OCC)OP(=O)(OCC)OCC
```

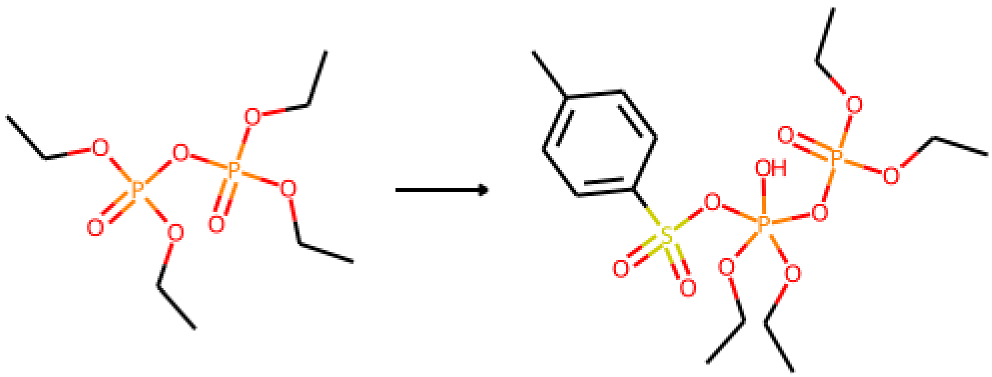

```
|Step  2|---------------------------------------------------------------------------
-------------------------
Retrosynthesis: CCOP(=O)(OCC)OP(O)(OCC)(OCC)OS(=O)(=O)c1ccc(C)cc1>>Cc1ccc(S(=O)
(=O)O)cc1.CCOP(=O)(O)OCC.CCOP(=O)(Cl)OCC
Reaction: Cc1ccc(S(=O)(=O)O)cc1.CCOP(=O)(O)OCC.CCOP(=O)(Cl)OCC>>CCOP(=O)(OCC)OP(O)
(OCC)(OCC)OS(=O)(=O)c1ccc(C)cc1
```

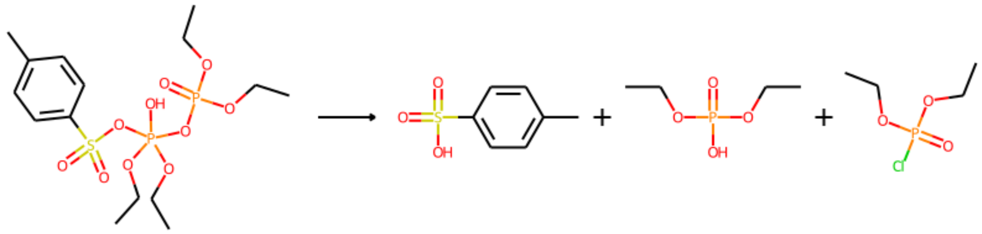

```
|Route 40|---------------------------------------------------------------------------
--------------------------
|Step  1|---------------------------------------------------------------------------
-------------------------
Retrosynthesis: CCN(CC)CCOCCOC(=O)C(CC)(CC)c1ccccc1>>CCC(C=O)
(CC)c1ccccc1.CCN(CC)CCOCCO
Reaction: CCC(C=O)(CC)c1ccccc1.CCN(CC)CCOCCO>>CCN(CC)CCOCCOC(=O)C(CC)(CC)c1ccccc1
```

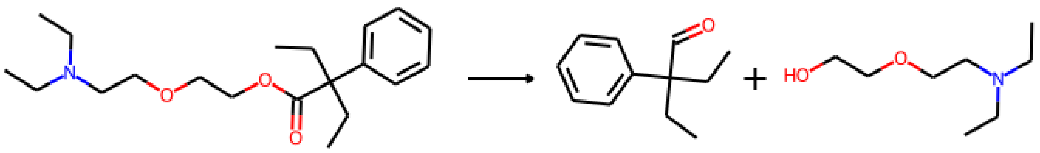

```
|Route 41|----------------------------------------------------------------------------------------------------
|Step  1|----------------------------------------------------------------------------------------------------
Retrosynthesis: CCC(=O)N(c1ccccc1)C1(COC)CCN(CCn2nnn(CC)c2=O)CC1>>CC.CCn1nn[nH]c1=O.CCC(=O)N(c1ccccc1)C1(COC)CCNCC1
Reaction: CC.CCn1nn[nH]c1=O.CCC(=O)N(c1ccccc1)C1(COC)CCNCC1>>CCC(=O)N(c1ccccc1)C1(COC)CCN(CCn2nnn(CC)c2=O)CC1
```

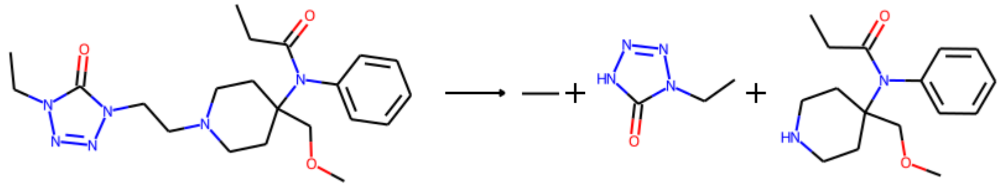

```
|Route 42|----------------------------------------------------------------------------------------------------
|Step  1|----------------------------------------------------------------------------------------------------
Retrosynthesis: CCCCCCCCCCCCN1CCCCCC1=O>>CCCCCCCCCCCCBr.O=C1CCCCCN1
Reaction: CCCCCCCCCCCCBr.O=C1CCCCCN1>>CCCCCCCCCCCCN1CCCCCC1=O
```

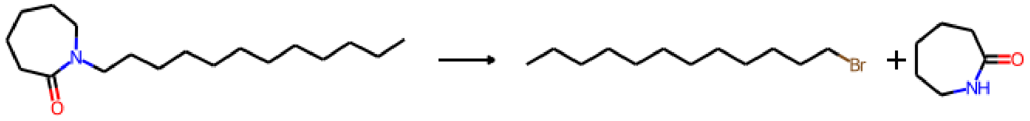

```
|Route 43|-----------------------------------------------------------------------------------------------------
|Step  1|----------------------------------------------------------------------------------------------------
Retrosynthesis: O=P(O)(NCCBr)NCCBr>>O[PH4].NCCBr.O=C=O
Reaction: O[PH4].NCCBr.O=C=O>>O=P(O)(NCCBr)NCCBr
```

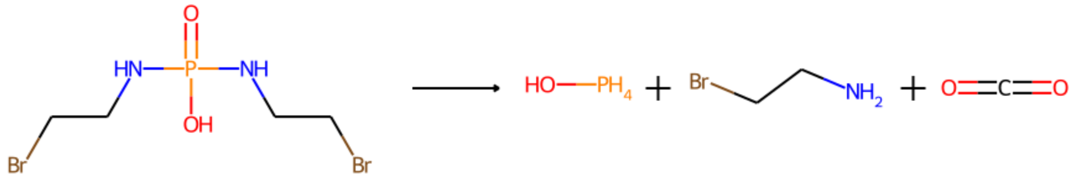

```
|Step  2|----------------------------------------------------------------------------------------------------
Retrosynthesis: O[PH4]>>C[PH](C)=O
Reaction: C[PH](C)=O>>O[PH4]
```

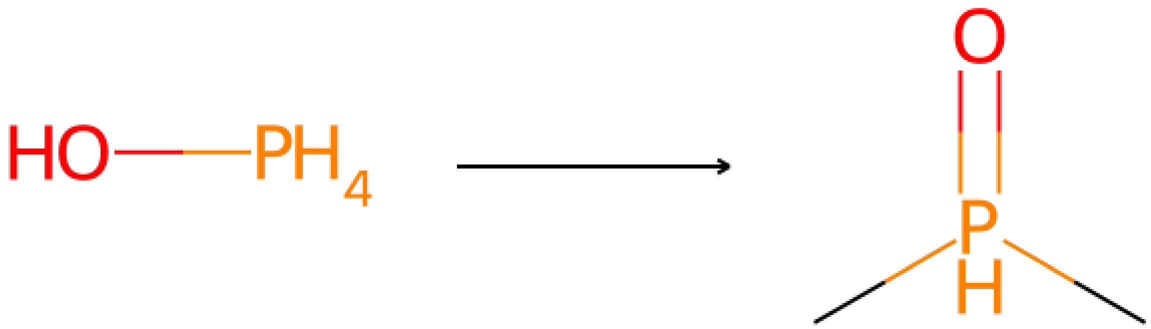

```
|Route 44|--------------------------------------------------------------------------------------------------
|Step  1|--------------------------------------------------------------------------------------------------
Retrosynthesis:
COc1cc(CNCCC2CCOCC2)ccc1Oc1cnc(C(N)=O)cn1>>COc1cc(C=O)ccc1Oc1cnc(C(N)=O)cn1.NCCC1CCOCC1
Reaction:
COc1cc(C=O)ccc1Oc1cnc(C(N)=O)cn1.NCCC1CCOCC1>>COc1cc(CNCCC2CCOCC2)ccc1Oc1cnc(C(N)=O)cn1
```

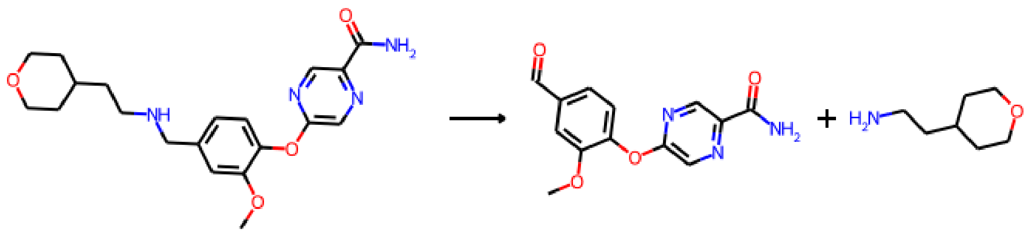

```
|Step  2|--------------------------------------------------------------------------------------------------
Retrosynthesis:
COc1cc(C=O)ccc1Oc1cnc(C(N)=O)cn1>>NC(=O)c1cnc(Cl)cn1.COc1cc(C=O)ccc1O
Reaction: NC(=O)c1cnc(Cl)cn1.COc1cc(C=O)ccc1O>>COc1cc(C=O)ccc1Oc1cnc(C(N)=O)cn1
```

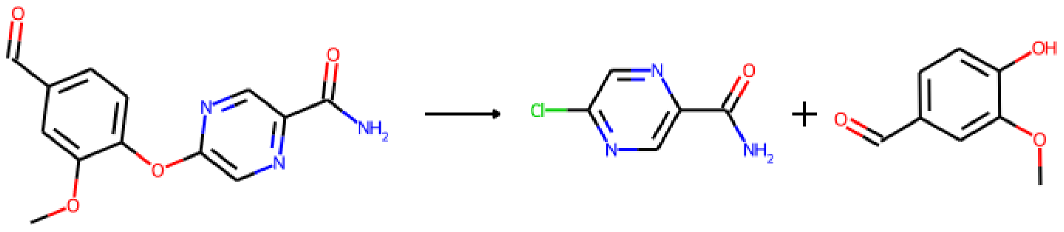

```
|Route 45|-------------------------------------------------------------------------------------------------------
|Step  1|-------------------------------------------------------------------------------------------------------
Retrosynthesis: CC1CCCC(C)N1NC(=O)c1ccc(Cl)c(S(N)(=O)=O)c1>>CC1=NCCCC1.CCl.NC(=O)c1ccc(Cl)c(S(N)(=O)=O)c1
Reaction: CC1=NCCCC1.CCl.NC(=O)c1ccc(Cl)c(S(N)(=O)=O)c1>>CC1CCCC(C)N1NC(=O)c1ccc(Cl)c(S(N)(=O)=O)c1
```

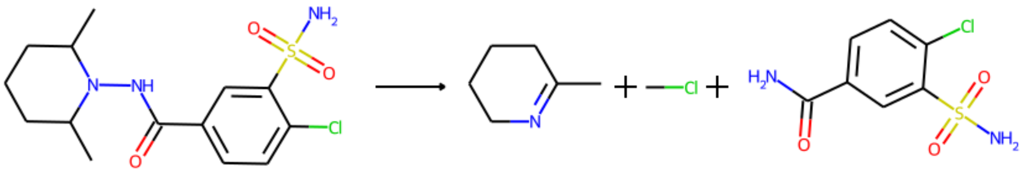

```
|Route 46|-------------------------------------------------------------------------------------------------------
|Step  1|-------------------------------------------------------------------------------------------------------
Retrosynthesis: Nc1c2c(nc3ccccc13)CCCC2>>N.c1ccc2nc3c(cc2c1)CCCC3
Reaction: N.c1ccc2nc3c(cc2c1)CCCC3>>Nc1c2c(nc3ccccc13)CCCC2
```

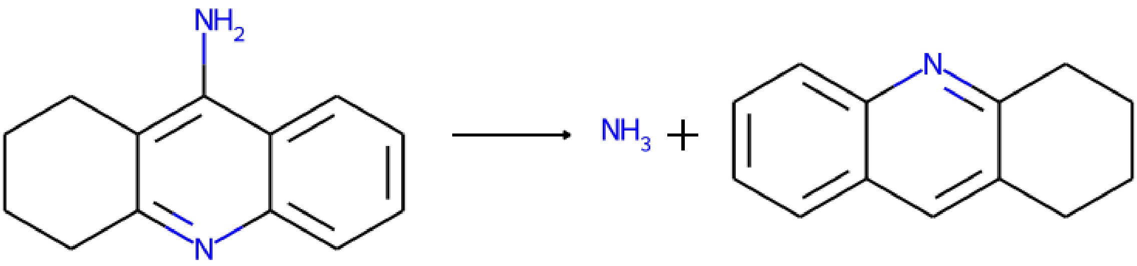

```
|Route 47|-----------------------------------------------------------------------------------------------------------
|Step  1|------------------------------------------------------------------------------------------------------------
Retrosynthesis: CN(C)CCN(Cc1cccs1)c1ccccn1>>CN(C)CCNc1ccccn1.ClCc1cccs1
Reaction: CN(C)CCNc1ccccn1.ClCc1cccs1>>CN(C)CCN(Cc1cccs1)c1ccccn1
```

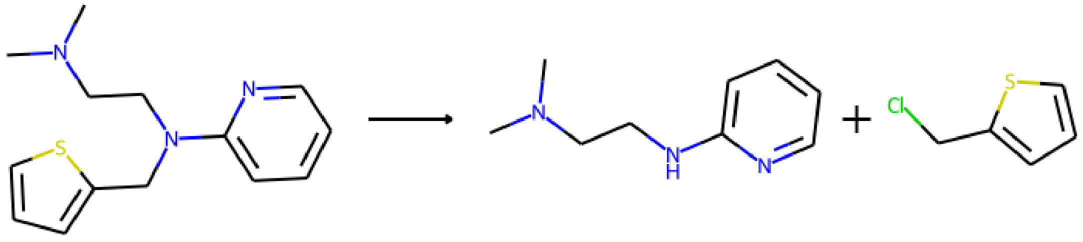

```
|Route 48|-----------------------------------------------------------------------------------------------------------
|Step  1|------------------------------------------------------------------------------------------------------------
Retrosynthesis:
Cc1cncc(CN2CCC(=C3c4ccc(Cl)cc4CCc4cccnc43)CC2)c1>>Clc1ccc2c(c1)CCc1cccnc1C2=C1CCNCC1
.Cc1cncc(C=O)c1
Reaction:
Clc1ccc2c(c1)CCc1cccnc1C2=C1CCNCC1.Cc1cncc(C=O)c1>>Cc1cncc(CN2CCC(=C3c4ccc(Cl)cc4CCc
4cccnc43)CC2)c1
```

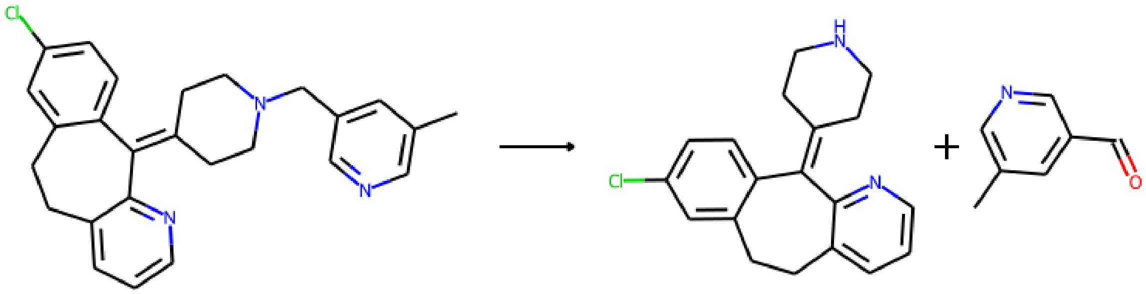

```
|Route 49|-----------------------------------------------------------------------------------------------------------
|Step  1|----------------------------------------------------------------------------------------------------------
Retrosynthesis: Cc1cccc(C)c1NC(=O)NCCCNC(C)C>>CC(C)NCCCN.Cc1cccc(C)c1NC=O
Reaction: CC(C)NCCCN.Cc1cccc(C)c1NC=O>>Cc1cccc(C)c1NC(=O)NCCCNC(C)C
```

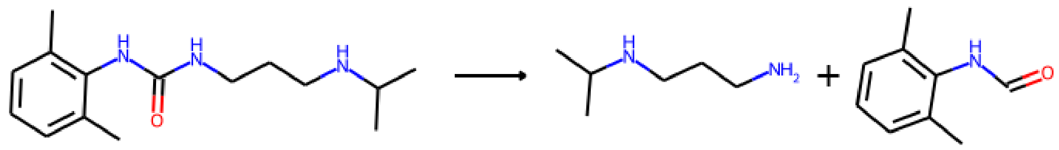

```
|Route 50|-----------------------------------------------------------------------------------------------------------
|Step  1|----------------------------------------------------------------------------------------------------------
Retrosynthesis:
COc1ccc(Cc2nccc3cc(OC)c(OC)cc23)cc1OC>>COc1ccc(C(=O)c2nccc3cc(OC)c(OC)cc23)cc1OC
Reaction:
COc1ccc(C(=O)c2nccc3cc(OC)c(OC)cc23)cc1OC>>COc1ccc(Cc2nccc3cc(OC)c(OC)cc23)cc1OC
```

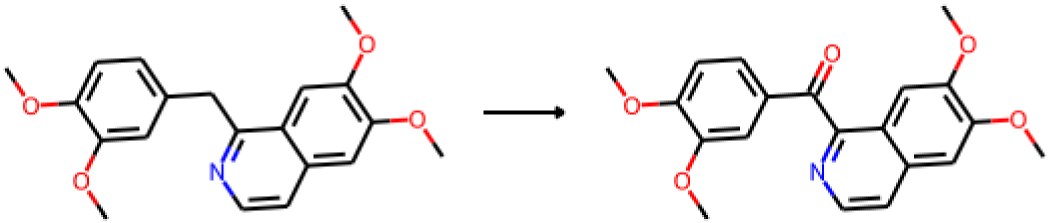

```
|Route 51|--------------------------------------------------------------------------
--------------------------
|Step  1|---------------------------------------------------------------------------
-------------------------
Retrosynthesis: OCC1(CO)COC(C(Cl)(Cl)Cl)OC1>>O=CC(Cl)(Cl)Cl.OCC(CO)(CO)CO
Reaction: O=CC(Cl)(Cl)Cl.OCC(CO)(CO)CO>>OCC1(CO)COC(C(Cl)(Cl)Cl)OC1
```

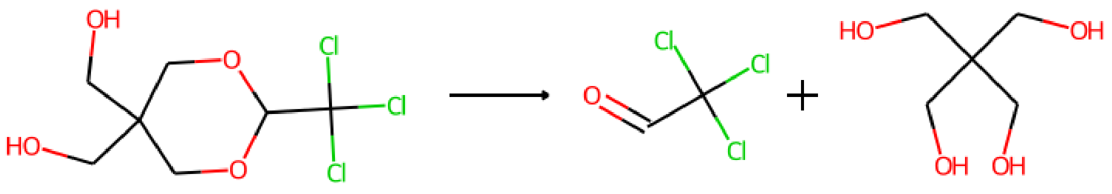

```
|Route 52|--------------------------------------------------------------------------
--------------------------
|Step  1|---------------------------------------------------------------------------
-------------------------
Retrosynthesis: CCOc1ccccc1OC(c1ccccc1)C1CNCCO1>>CCOc1ccccc1OC(c1ccccc1)C1CN(S(=O)
(=O)c2ccccc2)CCO1
Reaction: CCOc1ccccc1OC(c1ccccc1)C1CN(S(=O)
(=O)c2ccccc2)CCO1>>CCOc1ccccc1OC(c1ccccc1)C1CNCCO1
```

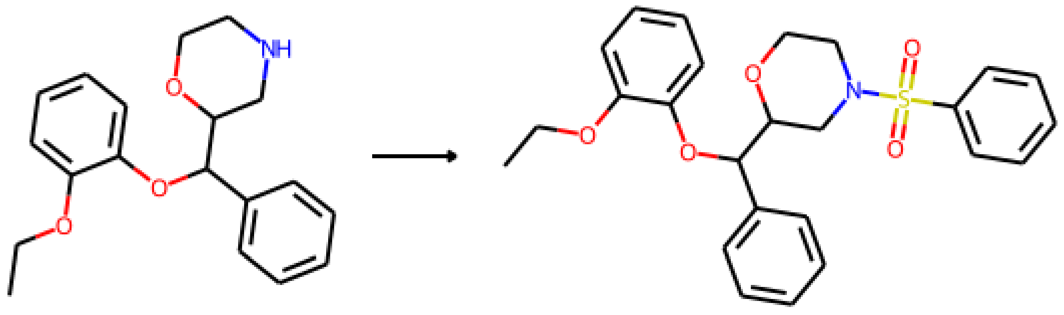

```
|Step  2|---------------------------------------------------------------------------
-------------------------
Retrosynthesis: CCOc1ccccc1OC(c1ccccc1)C1CN(S(=O)
(=O)c2ccccc2)CCO1>>CCOc1ccccc1O.O=S(=O)(c1ccccc1)N1CCOC(Cc2ccccc2)C1
Reaction: CCOc1ccccc1O.O=S(=O)
(c1ccccc1)N1CCOC(Cc2ccccc2)C1>>CCOc1ccccc1OC(c1ccccc1)C1CN(S(=O)(=O)c2ccccc2)CCO1
```

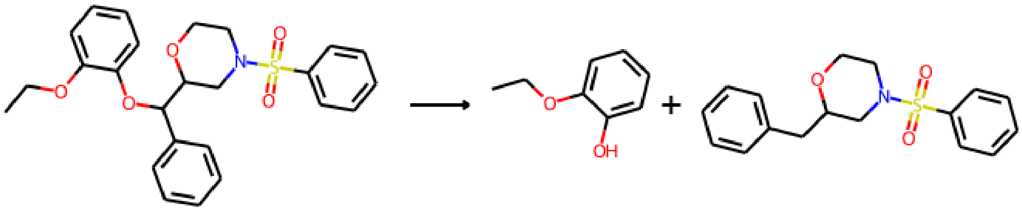

```
|Route 53|--------------------------------------------------------------------------
--------------------------
|Step  1|---------------------------------------------------------------------------
-------------------------
Retrosynthesis: COc1ccc2cccc(CCNC(C)=O)c2c1>>COc1ccc2cccc(CCN(C)C(C)=O)c2c1
Reaction: COc1ccc2cccc(CCN(C)C(C)=O)c2c1>>COc1ccc2cccc(CCNC(C)=O)c2c1
```

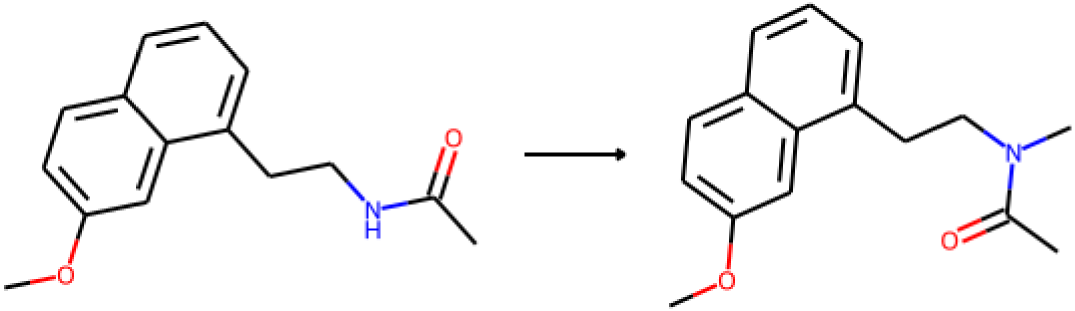

```
|Step  2|---------------------------------------------------------------------------
-------------------------
Retrosynthesis: COc1ccc2cccc(CCN(C)C(C)=O)c2c1>>CCOC(C)=O.CNCCc1cccc2ccc(OC)cc12
Reaction: CCOC(C)=O.CNCCc1cccc2ccc(OC)cc12>>COc1ccc2cccc(CCN(C)C(C)=O)c2c1
```

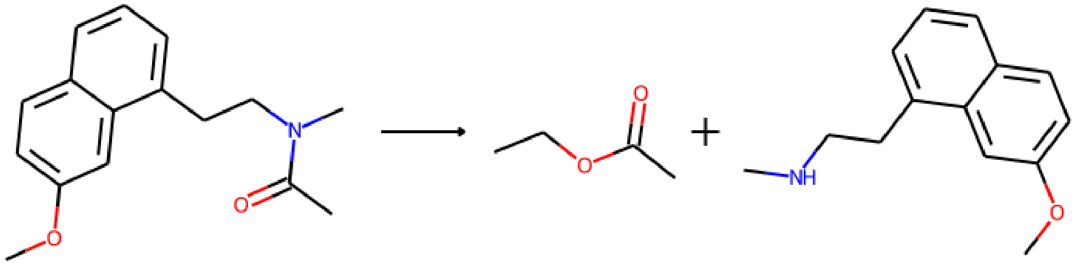

```
|Route 54|--------------------------------------------------------------------------
--------------------------
|Step  1|---------------------------------------------------------------------------
-------------------------
Retrosynthesis:
CCN(CC)C(=O)c1ccc(C2=CC3(CCNCC3)Oc3cccc(O)c32)cc1>>CCN1CCC2(C=C(c3ccc(C(=O)N(CC)CC)c
c3)c3c(O)cccc3O2)CC1
Reaction:
CCN1CCC2(C=C(c3ccc(C(=O)N(CC)CC)cc3)c3c(O)cccc3O2)CC1>>CCN(CC)C(=O)c1ccc(C2=CC3(CCNC
C3)Oc3cccc(O)c32)cc1
```

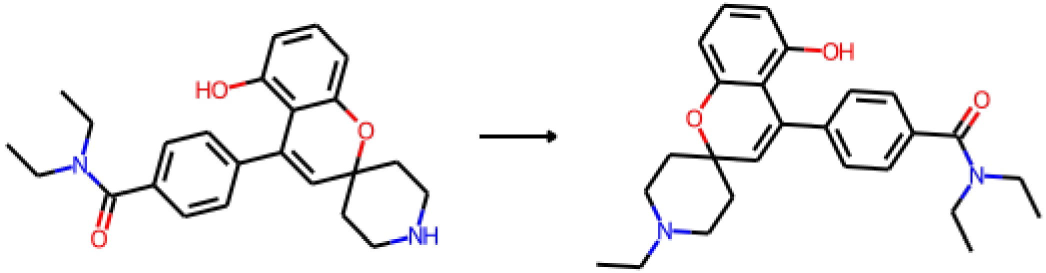

```
|Step  2|---------------------------------------------------------------------------------------------------
Retrosynthesis: CCN1CCC2(C=C(c3ccc(C(=O)N(CC)CC)cc3)c3c(O)cccc3O2)CC1>>CCN1CCC(C)
(O)CC1.CCN(CC)C(=O)c1ccc(C=O)cc1.Oc1ccccc1
Reaction: CCN1CCC(C)
(O)CC1.CCN(CC)C(=O)c1ccc(C=O)cc1.Oc1ccccc1>>CCN1CCC2(C=C(c3ccc(C(=O)N(CC)CC)cc3)c3c(
O)cccc3O2)CC1
```

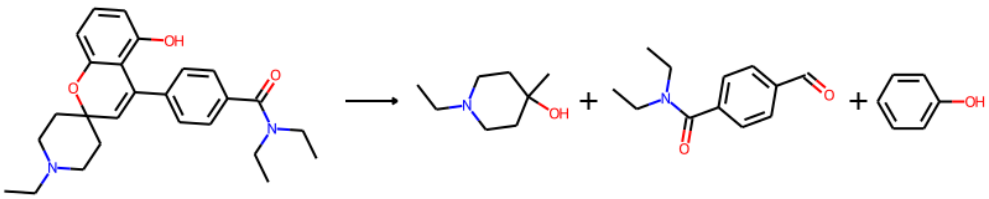

```
|Route 55|----------------------------------------------------------------------------------------------------
|Step  1|---------------------------------------------------------------------------------------------------
Retrosynthesis:
O=C(O)Cc1cc(I)c(Oc2cc(I)c(O)c(I)c2)c(I)c1>>O.NC(=O)Cc1cc(I)c(Oc2cc(I)c(O)c(I)c2)c(I)
c1
Reaction:
O.NC(=O)Cc1cc(I)c(Oc2cc(I)c(O)c(I)c2)c(I)c1>>O=C(O)Cc1cc(I)c(Oc2cc(I)c(O)c(I)c2)c(I)
c1
```

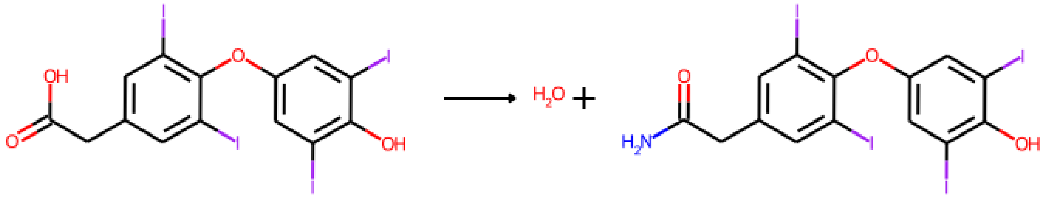

```
|Route 56|--------------------------------------------------------------------------------------------------------
|Step  1|--------------------------------------------------------------------------------------------------------
Retrosynthesis:
N#Cc1ccc(Cn2cncc2CN2CCN(c3cccc(Cl)c3)C(=O)C2)cc1>>N#Cc1ccc(Cn2cncc2C(=O)N2CCN(c3cccc(Cl)c3)C(=O)C2)cc1
Reaction:
N#Cc1ccc(Cn2cncc2C(=O)N2CCN(c3cccc(Cl)c3)C(=O)C2)cc1>>N#Cc1ccc(Cn2cncc2CN2CCN(c3cccc(Cl)c3)C(=O)C2)cc1
```

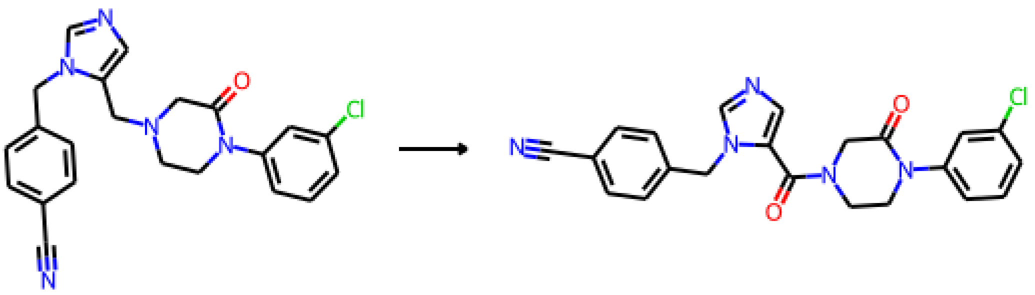

```
|Step  2|--------------------------------------------------------------------------------------------------------
Retrosynthesis:
N#Cc1ccc(Cn2cncc2C(=O)N2CCN(c3cccc(Cl)c3)C(=O)C2)cc1>>O=C1CNCCN1c1cccc(Cl)c1.N#Cc1ccc(Cn2cncc2C=O)cc1
Reaction:
O=C1CNCCN1c1cccc(Cl)c1.N#Cc1ccc(Cn2cncc2C=O)cc1>>N#Cc1ccc(Cn2cncc2C(=O)N2CCN(c3cccc(Cl)c3)C(=O)C2)cc1
```

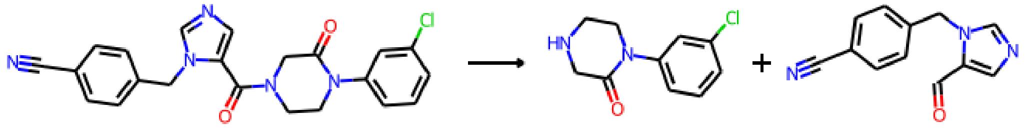

```
|Route 57|--------------------------------------------------------------------------------------------------------
|Step  1|--------------------------------------------------------------------------------------------------------
Retrosynthesis:
CN(C(=O)CNC(=O)CN)c1ccc(Cl)cc1C(=O)c1ccccc1Cl>>CN(C(=O)CNC(=O)CNC=O)c1ccc(Cl)cc1C(=O)c1ccccc1Cl
Reaction:
CN(C(=O)CNC(=O)CNC=O)c1ccc(Cl)cc1C(=O)c1ccccc1Cl>>CN(C(=O)CNC(=O)CN)c1ccc(Cl)cc1C(=O)c1ccccc1Cl
```

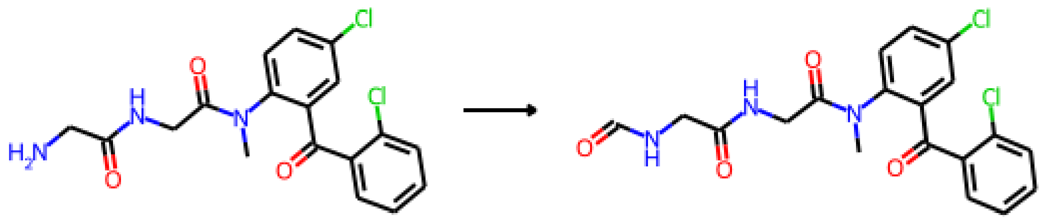

```
|Step  2|--------------------------------------------------------------------------------------------------------
Retrosynthesis:
CN(C(=O)CNC(=O)CNC=O)c1ccc(Cl)cc1C(=O)c1ccccc1Cl>>COC(=O)CNC(=O)CNC=O.CNc1ccc(Cl)cc1C(=O)c1ccccc1Cl
Reaction:
COC(=O)CNC(=O)CNC=O.CNc1ccc(Cl)cc1C(=O)c1ccccc1Cl>>CN(C(=O)CNC(=O)CNC=O)c1ccc(Cl)cc1C(=O)c1ccccc1Cl
```

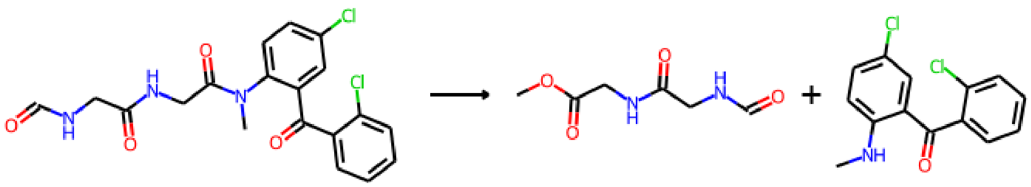

```
|Route 58|------------------------------------------------------------------------------------------------------
|Step  1|-------------------------------------------------------------------------------------------------------
Retrosynthesis:
CCN(CC)CCN1C(=O)CN=C(c2ccccc2F)c2cc(Cl)ccc21>>CCN(CC)CCN(C(=O)CN)c1ccc(Cl)cc1C(=O)c1ccccc1F
Reaction:
CCN(CC)CCN(C(=O)CN)c1ccc(Cl)cc1C(=O)c1ccccc1F>>CCN(CC)CCN1C(=O)CN=C(c2ccccc2F)c2cc(Cl)ccc21
```

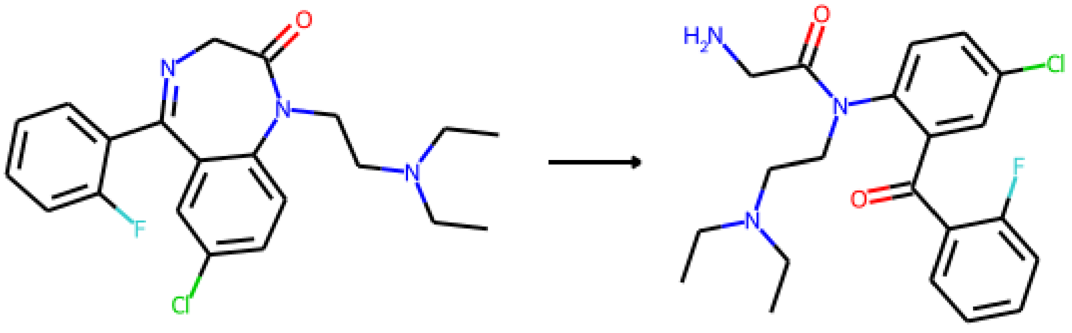

```
|Step  2|-------------------------------------------------------------------------------------------------------
Retrosynthesis:
CCN(CC)CCN(C(=O)CN)c1ccc(Cl)cc1C(=O)c1ccccc1F>>CCN(CC)CCN(C(=O)CNC(=O)OC(C)(C)C)c1ccc(Cl)cc1C(=O)c1ccccc1F
Reaction: CCN(CC)CCN(C(=O)CNC(=O)OC(C)(C)C)c1ccc(Cl)cc1C(=O)c1ccccc1F>>CCN(CC)CCN(C(=O)CN)c1ccc(Cl)cc1C(=O)c1ccccc1F
```

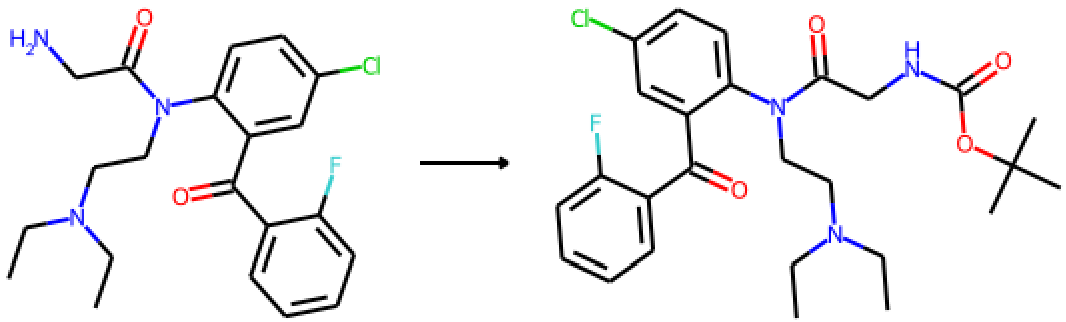

```
|Step  3|--------------------------------------------------------------------------
-------------------------
Retrosynthesis: CCN(CC)CCN(C(=O)CNC(=O)OC(C)
(C)C)c1ccc(Cl)cc1C(=O)c1ccccc1F>>CCN(CC)CCNc1ccc(Cl)cc1C(=O)c1ccccc1F.COC(=O)CNC(=O)
OC(C)(C)C
Reaction: CCN(CC)CCNc1ccc(Cl)cc1C(=O)c1ccccc1F.COC(=O)CNC(=O)OC(C)
(C)C>>CCN(CC)CCN(C(=O)CNC(=O)OC(C)(C)C)c1ccc(Cl)cc1C(=O)c1ccccc1F
```

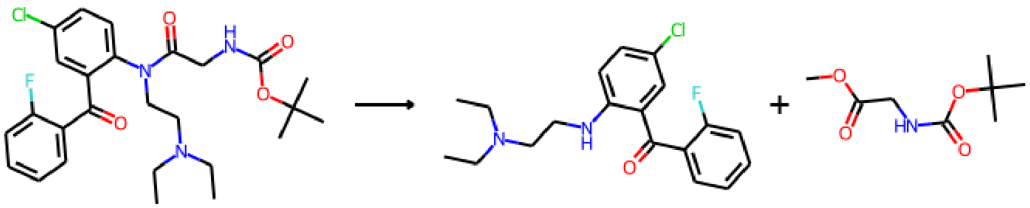

```
|Step  4|--------------------------------------------------------------------------
-------------------------
Retrosynthesis:
CCN(CC)CCNc1ccc(Cl)cc1C(=O)c1ccccc1F>>CCN(CC)CCNc1ccc(Cl)cc1.O=Cc1ccccc1F
Reaction: CCN(CC)CCNc1ccc(Cl)cc1.O=Cc1ccccc1F>>CCN(CC)CCNc1ccc(Cl)cc1C(=O)c1ccccc1F
```

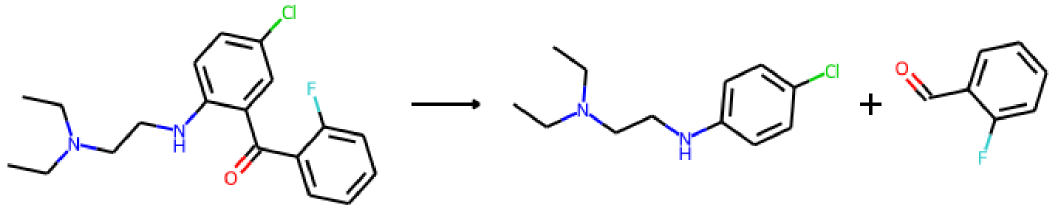

```
|Route 59|--------------------------------------------------------------------------
--------------------------
|Step  1|---------------------------------------------------------------------------
-------------------------
Retrosynthesis: CCN(CC)CCOc1ccccc1OC>>CCN(CC)CCBr.COc1ccccc1O
Reaction: CCN(CC)CCBr.COc1ccccc1O>>CCN(CC)CCOc1ccccc1OC
```

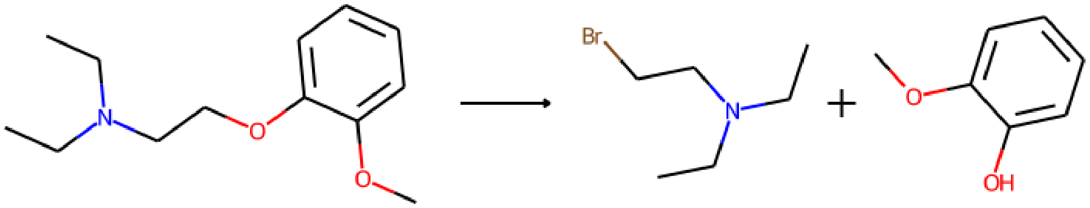

```
|Route 60|--------------------------------------------------------------------------
--------------------------
|Step  1|---------------------------------------------------------------------------
-------------------------
Retrosynthesis: OC(CI)CI>>COC(CI)CI
Reaction: COC(CI)CI>>OC(CI)CI
```

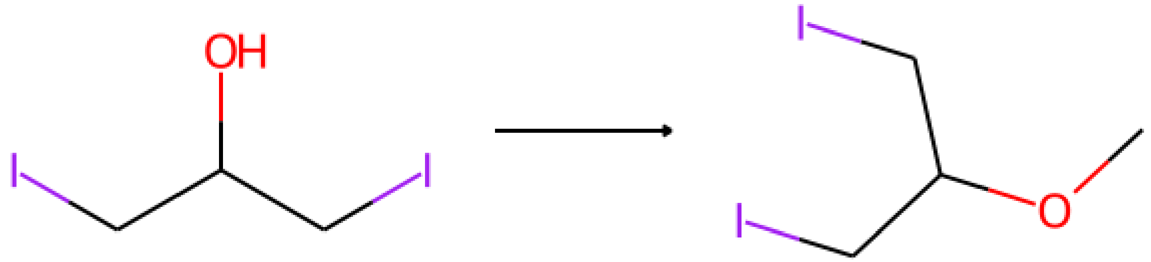

```
|Step  2|---------------------------------------------------------------------------
-------------------------
Retrosynthesis: COC(CI)CI>>CI.COC(C)CI
Reaction: CI.COC(C)CI>>COC(CI)CI
```

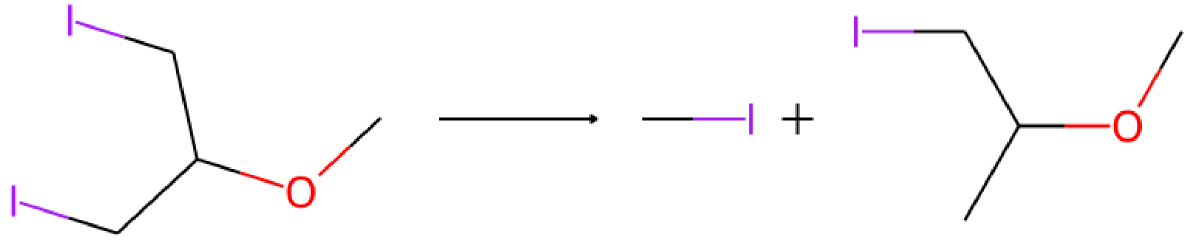

```
|Route 61|--------------------------------------------------------------------------
--------------------------
|Step  1|---------------------------------------------------------------------------
-------------------------
Retrosynthesis: O=C1c2cccc(O)c2C(=O)c2c(O)cccc21>>O.O=C1c2ccccc2C(=O)c2c(O)cccc21
Reaction: O.O=C1c2ccccc2C(=O)c2c(O)cccc21>>O=C1c2cccc(O)c2C(=O)c2c(O)cccc21
```

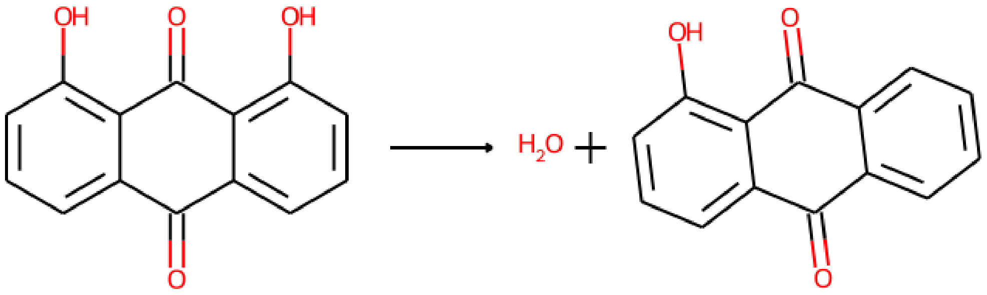

```
|Route 62|--------------------------------------------------------------------------
--------------------------
|Step  1|---------------------------------------------------------------------------
-------------------------
Retrosynthesis: O=C(O)c1ccccc1O.OCCN(CCO)CCO>>OCCN(CCO)CCO.CC(=O)Oc1ccccc1C(=O)O
Reaction: OCCN(CCO)CCO.CC(=O)Oc1ccccc1C(=O)O>>O=C(O)c1ccccc1O.OCCN(CCO)CCO
```

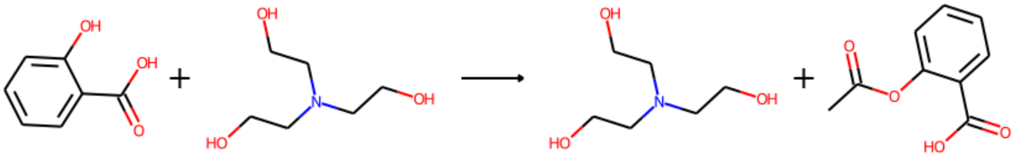

```
|Route 63|--------------------------------------------------------------------------
--------------------------
|Step  1|---------------------------------------------------------------------------
-------------------------
Retrosynthesis: COC1(c2ccccc2)C2CCCC1CN(C)C2>>c1ccccc1.COC1C2CCCC1CN(C)C2
Reaction: c1ccccc1.COC1C2CCCC1CN(C)C2>>COC1(c2ccccc2)C2CCCC1CN(C)C2
```

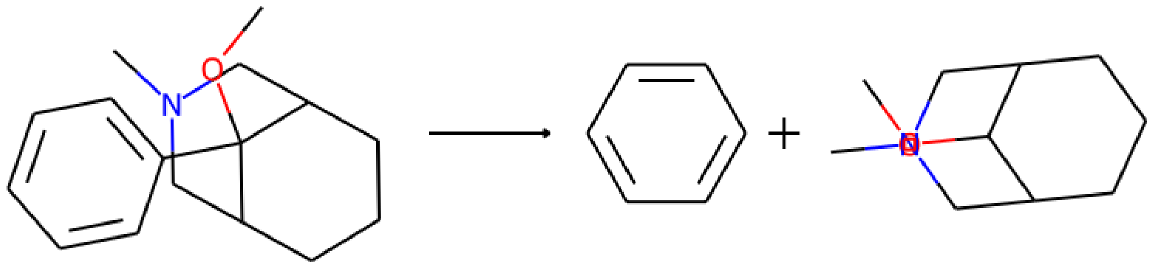

```
|Step  2|-------------------------------------------------------------------------------------------------------
Retrosynthesis: COC1C2CCCC1CN(C)C2>>CO.CN(C)CC1C=CCCC1
Reaction: CO.CN(C)CC1C=CCCC1>>COC1C2CCCC1CN(C)C2
```

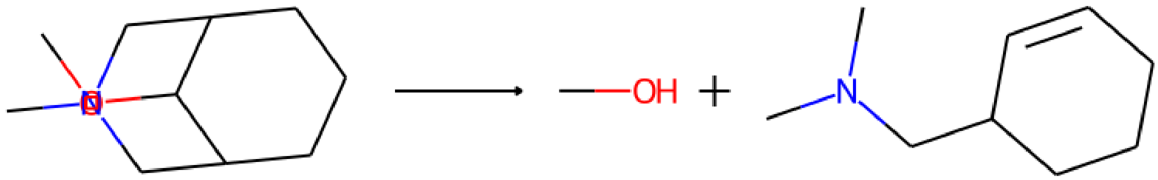


```
|Route 64|-------------------------------------------------------------------------------------------------------
|Step  1|-------------------------------------------------------------------------------------------------------
Retrosynthesis: O=C(O)c1ccc(C(c2ccccc2)(c2ccccc2)c2ccccc2)cn1>>CC(=O)OC(=O)c1ccc(C(c2ccccc2)(c2ccccc2)c2ccccc2)cn1
Reaction: CC(=O)OC(=O)c1ccc(C(c2ccccc2)(c2ccccc2)c2ccccc2)cn1>>O=C(O)c1ccc(C(c2ccccc2)(c2ccccc2)c2ccccc2)cn1
```

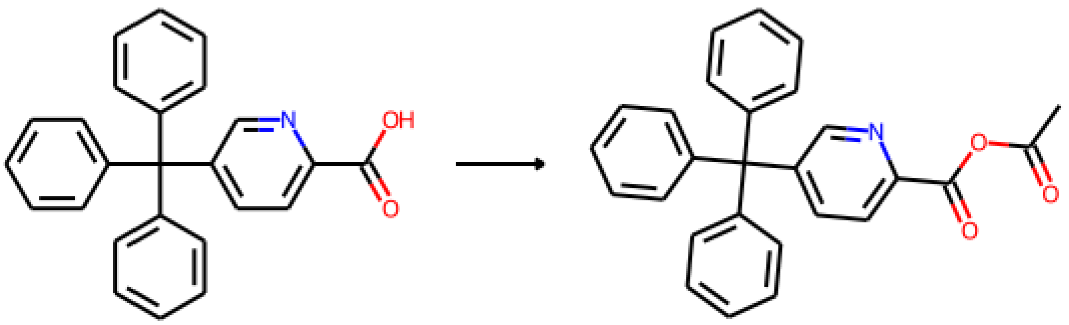

```
|Step  2|---------------------------------------------------------------------------
-------------------------
Retrosynthesis: CC(=O)OC(=O)c1ccc(C(c2ccccc2)
(c2ccccc2)c2ccccc2)cn1>>Cc1ccc(C(c2ccccc2)(c2ccccc2)c2ccccc2)cn1.O.CC(=O)O
Reaction: Cc1ccc(C(c2ccccc2)
(c2ccccc2)c2ccccc2)cn1.O.CC(=O)O>>CC(=O)OC(=O)c1ccc(C(c2ccccc2)
(c2ccccc2)c2ccccc2)cn1
```

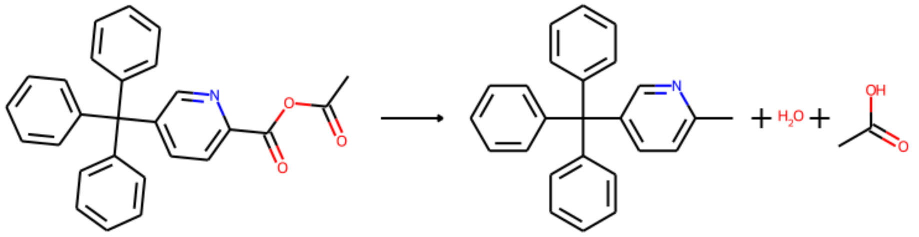

```
|Step  3|---------------------------------------------------------------------------
-------------------------
Retrosynthesis: Cc1ccc(C(c2ccccc2)(c2ccccc2)c2ccccc2)cn1>>ClC(c1ccccc1)
(c1ccccc1)c1ccccc1.Cc1ccccn1
Reaction: ClC(c1ccccc1)(c1ccccc1)c1ccccc1.Cc1ccccn1>>Cc1ccc(C(c2ccccc2)
(c2ccccc2)c2ccccc2)cn1
```

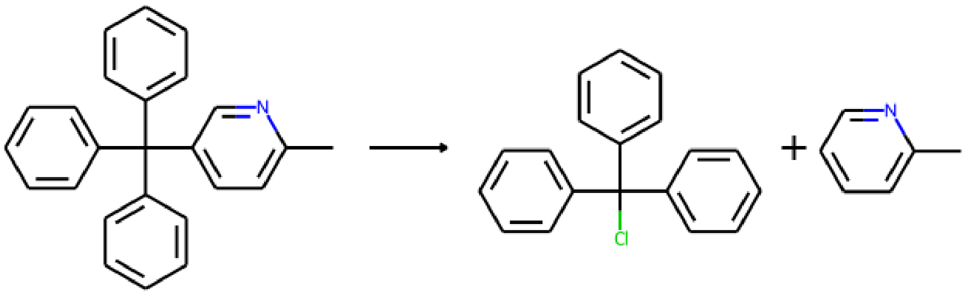

```
|Route 65|---------------------------------------------------------------------------
--------------------------
|Step  1|----------------------------------------------------------------------------
-------------------------
Retrosynthesis:
Cc1ccccc1CN1CCN(CCSC(c2ccccc2)c2ccccc2Cl)CC1>>Clc1ccccc1C(Cl)c1ccccc1.Cc1ccccc1CN1CC
N(CCS)CC1
Reaction:
Clc1ccccc1C(Cl)c1ccccc1.Cc1ccccc1CN1CCN(CCS)CC1>>Cc1ccccc1CN1CCN(CCSC(c2ccccc2)c2ccc
cc2Cl)CC1
```

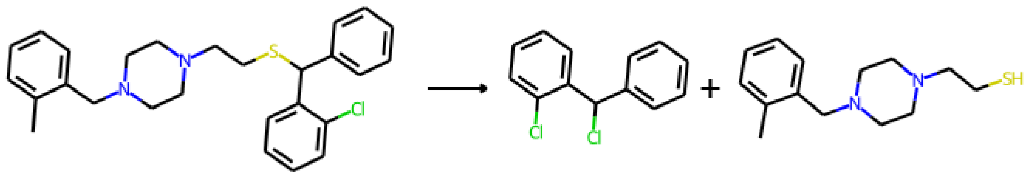

```
|Step  2|----------------------------------------------------------------------------
-------------------------
Retrosynthesis: Cc1ccccc1CN1CCN(CCS)CC1>>Cc1ccccc1CN1CCN(CCSC(=N)N)CC1
Reaction: Cc1ccccc1CN1CCN(CCSC(=N)N)CC1>>Cc1ccccc1CN1CCN(CCS)CC1
```

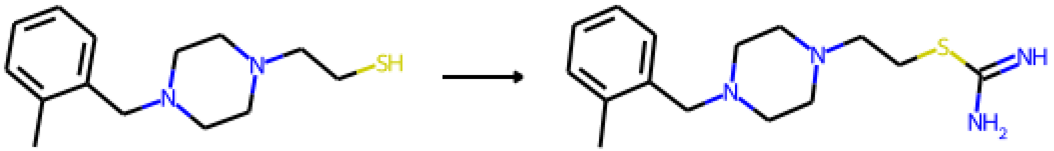

```
|Step  3|---------------------------------------------------------------------------
-------------------------
Retrosynthesis: Cc1ccccc1CN1CCN(CCSC(=N)N)CC1>>Cc1ccccc1CN1CCN(CCCl)CC1.NC(N)=S
Reaction: Cc1ccccc1CN1CCN(CCCl)CC1.NC(N)=S>>Cc1ccccc1CN1CCN(CCSC(=N)N)CC1
```

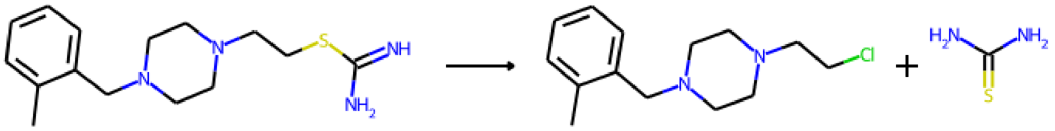

```
|Route 66|--------------------------------------------------------------------------
--------------------------
|Step  1|---------------------------------------------------------------------------
-------------------------
Retrosynthesis: O=C1NS(=O)(=O)c2ccccc21>>CN1C(=O)c2ccccc2S1(=O)=O
Reaction: CN1C(=O)c2ccccc2S1(=O)=O>>O=C1NS(=O)(=O)c2ccccc21
```

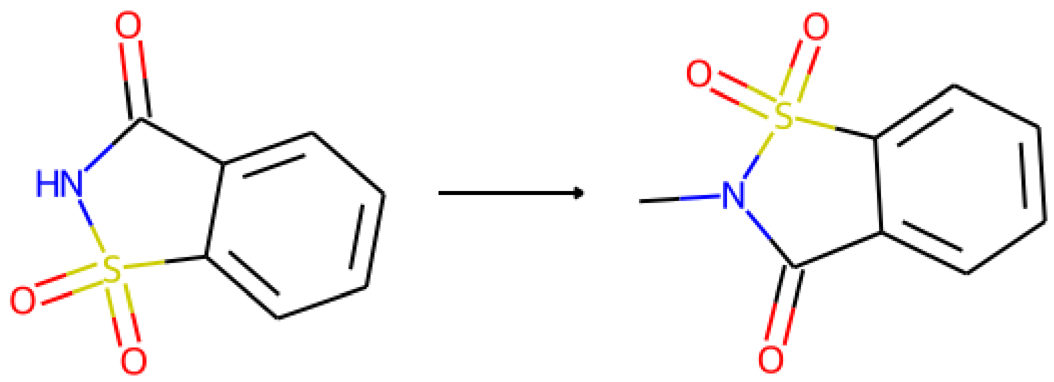

```
|Route 67|--------------------------------------------------------------------------
--------------------------
|Step  1|---------------------------------------------------------------------------
-------------------------
Retrosynthesis: Cc1cc(C)c(=O)[nH]n1>>Cc1cc(CCl)n[nH]c1=O
Reaction: Cc1cc(CCl)n[nH]c1=O>>Cc1cc(C)c(=O)[nH]n1
```

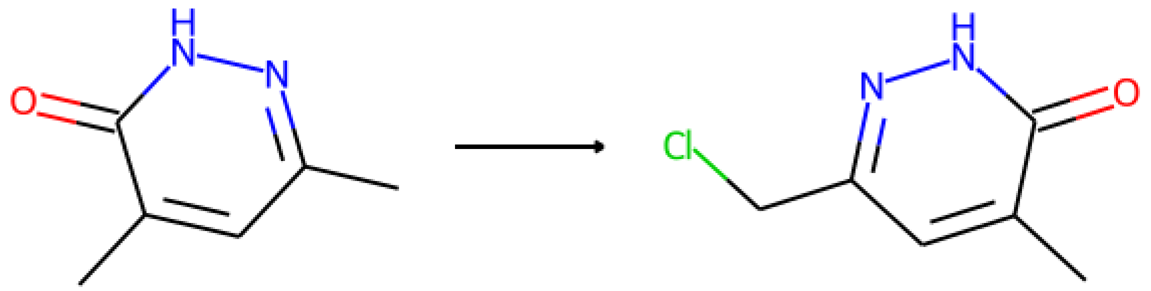

```
|Step  2|---------------------------------------------------------------------------
-------------------------
Retrosynthesis: Cc1cc(CCl)n[nH]c1=O>>Cc1n[nH]c(=O)c(C)c1C.ClCl
Reaction: Cc1n[nH]c(=O)c(C)c1C.ClCl>>Cc1cc(CCl)n[nH]c1=O
```

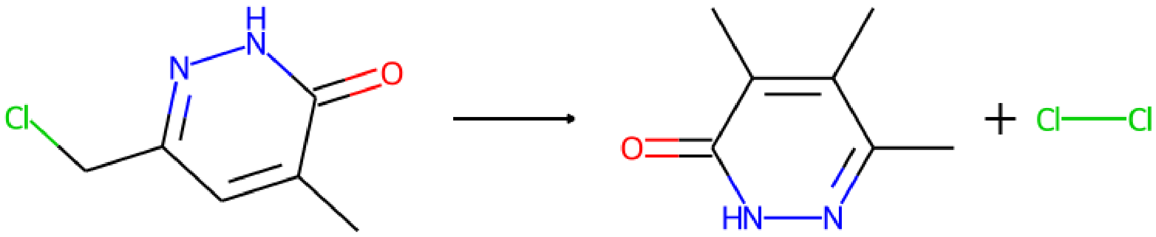

```
|Route 68|-----------------------------------------------------------------------------------------------------------
|Step  1|-----------------------------------------------------------------------------------------------------------
Retrosynthesis:
c1ccc(C(c2ccccc2)N2CCN(CCCC3(c4ccccc4)OCCO3)CC2)cc1>>c1ccc(C(c2ccccc2)N2CCNCC2)cc1.B
rCCCC1(c2ccccc2)OCCO1
Reaction:
c1ccc(C(c2ccccc2)N2CCNCC2)cc1.BrCCCC1(c2ccccc2)OCCO1>>c1ccc(C(c2ccccc2)N2CCN(CCCC3(c
4ccccc4)OCCO3)CC2)cc1
```

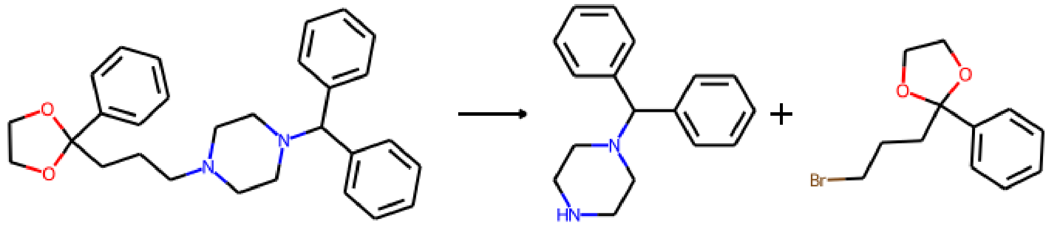

```
|Route 69|-----------------------------------------------------------------------------------------------------------
|Step  1|-----------------------------------------------------------------------------------------------------------
Retrosynthesis: CCCCNC(=O)OCC#CI>>C#CCOC(=O)NCCCC.I
Reaction: C#CCOC(=O)NCCCC.I>>CCCCNC(=O)OCC#CI
```

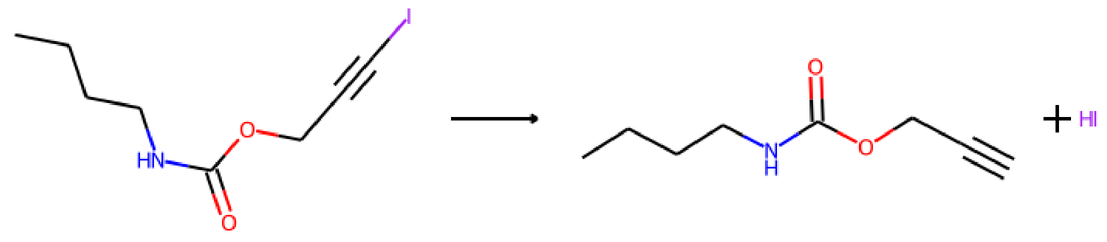

```
|Route 70|------------------------------------------------------------------------------------------------------------
|Step  1|-----------------------------------------------------------------------------------------------------------
Retrosynthesis:
Cn1c(=O)c2c(ncn2CC2SCCS2)n(C)c1=O>>Cn1c(=O)c2[nH]cnc2n(C)c1=O.CC1SCCS1
Reaction: Cn1c(=O)c2[nH]cnc2n(C)c1=O.CC1SCCS1>>Cn1c(=O)c2c(ncn2CC2SCCS2)n(C)c1=O
```

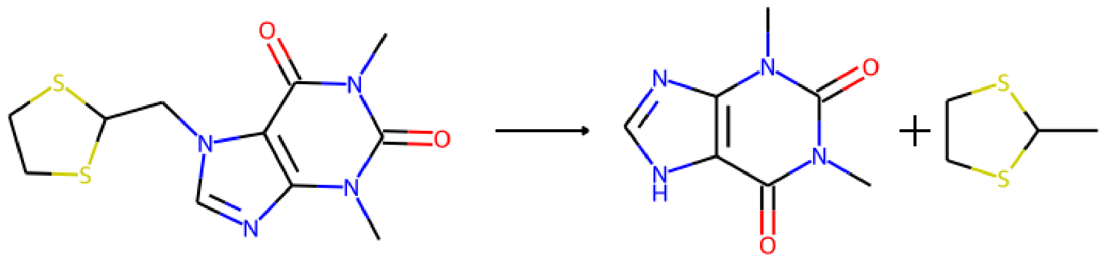

```
|Route 71|------------------------------------------------------------------------------------------------------------
|Step  1|-----------------------------------------------------------------------------------------------------------
Retrosynthesis:
CCCOC(=O)Cc1ccc(OCC(=O)N(CC)CC)c(OC)c1>>CCN(CC)C(=O)CCl.CCCOC(=O)Cc1ccc(O)c(OC)c1
Reaction:
CCN(CC)C(=O)CCl.CCCOC(=O)Cc1ccc(O)c(OC)c1>>CCCOC(=O)Cc1ccc(OCC(=O)N(CC)CC)c(OC)c1
```

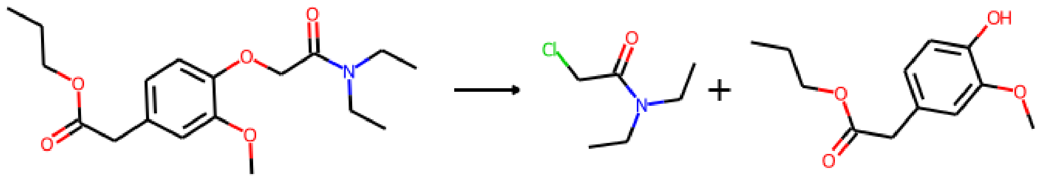

```
|Step  2|--------------------------------------------------------------------------------
-------------------------
Retrosynthesis: CCCOC(=O)Cc1ccc(O)c(OC)c1>>COc1cc(CC(=O)O)ccc1O.CCCBr
Reaction: COc1cc(CC(=O)O)ccc1O.CCCBr>>CCCOC(=O)Cc1ccc(O)c(OC)c1
```

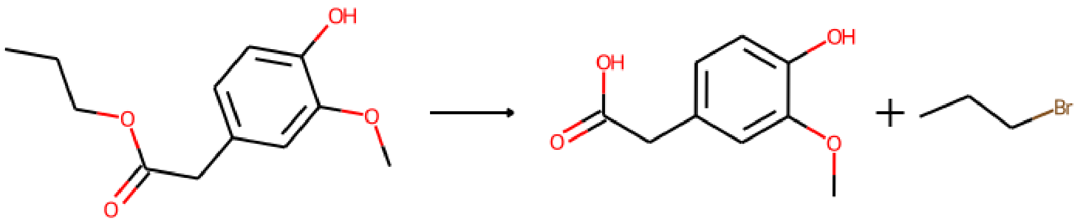

```
|Route 72|-------------------------------------------------------------------------------
--------------------------
|Step  1|--------------------------------------------------------------------------------
-------------------------
Retrosynthesis: Cc1c(C)c2c(c(C)c1OC(=O)C(C)(C)Oc1ccc(Cl)cc1)CCC(C)
(CCCC(C)CCCC(C)CCCC(C)C)O2>>CC(C)(C=O)Oc1ccc(Cl)cc1.Cc1c(C)c2c(c(C)c1O)CCC(C)
(CCCC(C)CCCC(C)CCCC(C)C)O2
Reaction: CC(C)(C=O)Oc1ccc(Cl)cc1.Cc1c(C)c2c(c(C)c1O)CCC(C)
(CCCC(C)CCCC(C)CCCC(C)C)O2>>Cc1c(C)c2c(c(C)c1OC(=O)C(C)(C)Oc1ccc(Cl)cc1)CCC(C)
(CCCC(C)CCCC(C)CCCC(C)C)O2
```

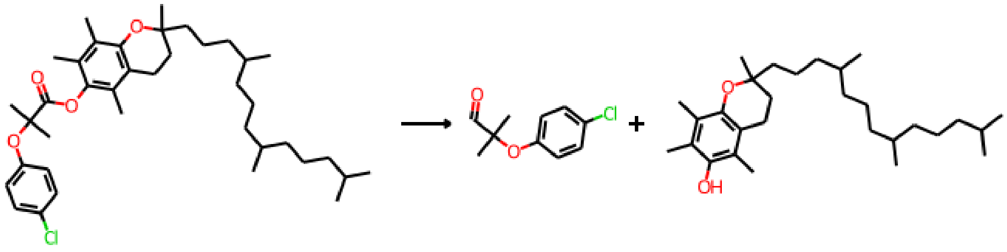


```
|Route 73|--------------------------------------------------------------------------
--------------------------
|Step  1|--------------------------------------------------------------------------
-------------------------
Retrosynthesis:
Cc1nc2n(c(=O)c1CCN1CCC(c3noc4cc(F)ccc34)CC1)CCCC2>>Fc1ccc2c(C3CCNCC3)noc2c1.CCc1c(C)
nc2n(c1=O)CCCC2
Reaction:
Fc1ccc2c(C3CCNCC3)noc2c1.CCc1c(C)nc2n(c1=O)CCCC2>>Cc1nc2n(c(=O)c1CCN1CCC(c3noc4cc(F)
ccc34)CC1)CCCC2
```

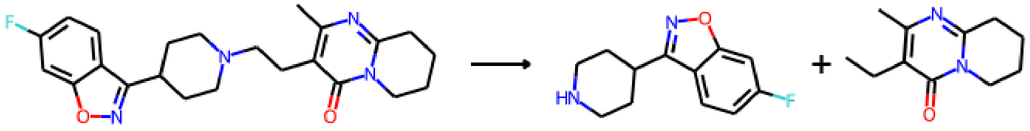


```
|Route 74|--------------------------------------------------------------------------
--------------------------
|Step  1|--------------------------------------------------------------------------
-------------------------
Retrosynthesis: Cc1ccc(C)c(OCCCC(C)(C)C(=O)O)c1>>CC(=O)OC(=O)C(C)(C)CCCOc1cc(C)ccc1C
Reaction: CC(=O)OC(=O)C(C)(C)CCCOc1cc(C)ccc1C>>Cc1ccc(C)c(OCCCC(C)(C)C(=O)O)c1
```

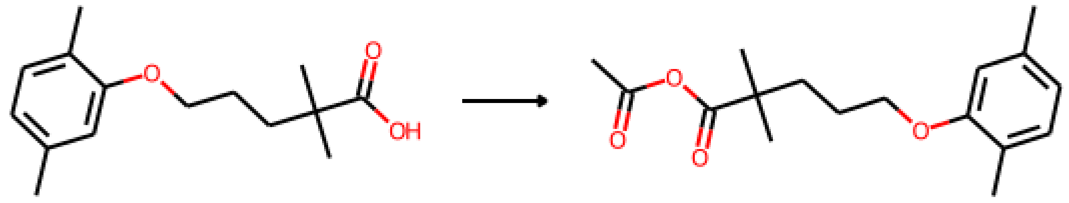

```
|Step  2|----------------------------------------------------------------------------------
-------------------------
Retrosynthesis: CC(=O)OC(=O)C(C)(C)CCCOc1cc(C)ccc1C>>Cc1ccc(C)c(OCCCC(C)
(C)C=O)c1.CC(=O)O
Reaction: Cc1ccc(C)c(OCCCC(C)(C)C=O)c1.CC(=O)O>>CC(=O)OC(=O)C(C)(C)CCCOc1cc(C)ccc1C
```

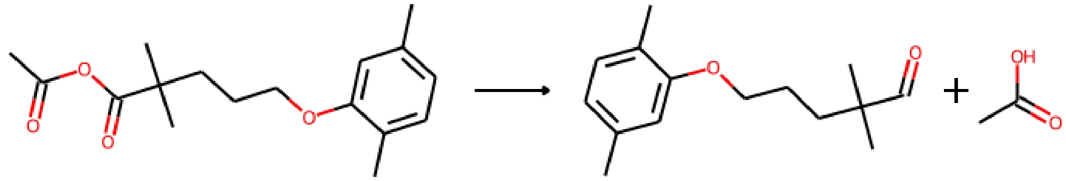

```
|Step  3|----------------------------------------------------------------------------------
-------------------------
Retrosynthesis: Cc1ccc(C)c(OCCCC(C)(C)C=O)c1>>CC(C)(C=O)CCCCl.Cc1ccc(C)c(O)c1
Reaction: CC(C)(C=O)CCCCl.Cc1ccc(C)c(O)c1>>Cc1ccc(C)c(OCCCC(C)(C)C=O)c1
```

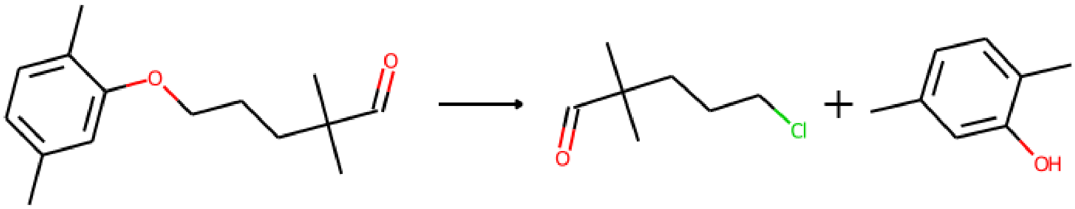


```
|Route 75|----------------------------------------------------------------------------
--------------------------
|Step  1|-----------------------------------------------------------------------------
-------------------------
Retrosynthesis: COc1cc(Nc2ncc(F)c(Nc3ccc4c(n3)N(COP(=O)(O)O)C(=O)C(C)
(C)O4)n2)cc(OC)c1OC>>CCCCCCCCCCCCCCC(OP(=O)(O)O)N1C(=O)C(C)
(C)Oc2ccc(Nc3nc(Nc4cc(OC)c(OC)c(OC)c4)ncc3F)nc21
Reaction: CCCCCCCCCCCCCCC(OP(=O)(O)O)N1C(=O)C(C)
(C)Oc2ccc(Nc3nc(Nc4cc(OC)c(OC)c(OC)c4)ncc3F)nc21>>COc1cc(Nc2ncc(F)c(Nc3ccc4c(n3)N(CO
P(=O)(O)O)C(=O)C(C)(C)O4)n2)cc(OC)c1OC
```

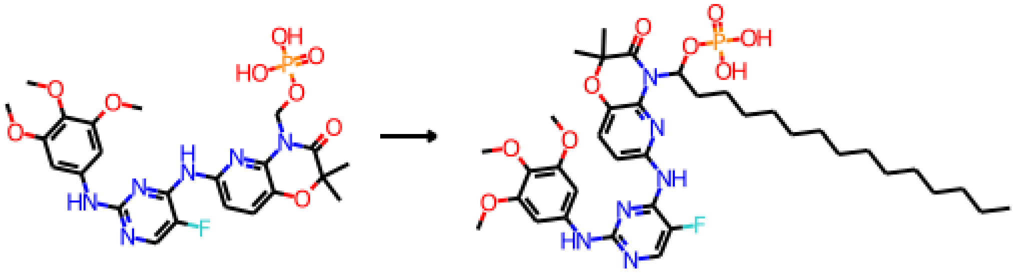


```
|Step  2|-----------------------------------------------------------------------------
-------------------------
Retrosynthesis: CCCCCCCCCCCCCCC(OP(=O)(O)O)N1C(=O)C(C)
(C)Oc2ccc(Nc3nc(Nc4cc(OC)c(OC)c(OC)c4)ncc3F)nc21>>O=P(=O)O.CCCCCCCCCCCCCCC(O)N1C(=O)
C(C)(C)Oc2ccc(Nc3nc(Nc4cc(OC)c(OC)c(OC)c4)ncc3F)nc21
Reaction: O=P(=O)O.CCCCCCCCCCCCCCC(O)N1C(=O)C(C)
(C)Oc2ccc(Nc3nc(Nc4cc(OC)c(OC)c(OC)c4)ncc3F)nc21>>CCCCCCCCCCCCCCC(OP(=O)
(O)O)N1C(=O)C(C)(C)Oc2ccc(Nc3nc(Nc4cc(OC)c(OC)c(OC)c4)ncc3F)nc21
```

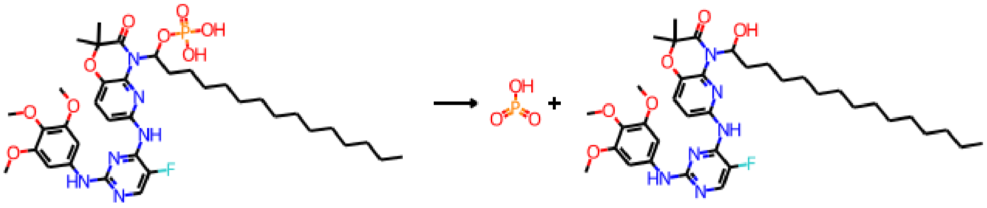

```
|Step  3|--------------------------------------------------------------------------------------------------
Retrosynthesis: CCCCCCCCCCCCCCC(O)N1C(=O)C(C)(C)Oc2ccc(Nc3nc(Nc4cc(OC)c(OC)c(OC)c4)ncc3F)nc21>>CCCCCCCCCCCCCCC=O.COc1cc(Nc2ncc(F)c(Nc3ccc4c(n3)NC(=O)C(C)(C)O4)n2)cc(OC)c1OC
Reaction: CCCCCCCCCCCCCCC=O.COc1cc(Nc2ncc(F)c(Nc3ccc4c(n3)NC(=O)C(C)(C)O4)n2)cc(OC)c1OC>>CCCCCCCCCCCCCCC(O)N1C(=O)C(C)(C)Oc2ccc(Nc3nc(Nc4cc(OC)c(OC)c(OC)c4)ncc3F)nc21
```

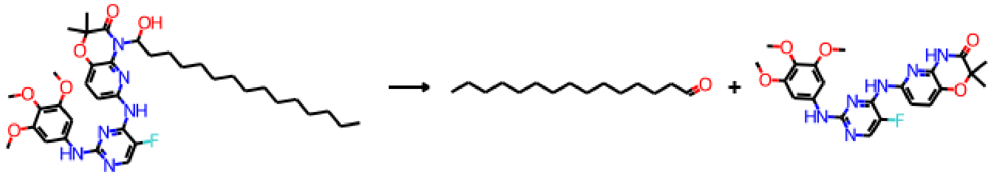

```
|Route 76|--------------------------------------------------------------------------------------------------
|Step  1|--------------------------------------------------------------------------------------------------
Retrosynthesis: CCCCCCCN(CC)CCCC(O)c1ccc(NS(C)(=O)=O)cc1>>CCCCCCCNCC.CCCC(=O)c1ccc(NS(C)(=O)=O)cc1
Reaction: CCCCCCCNCC.CCCC(=O)c1ccc(NS(C)(=O)=O)cc1>>CCCCCCCN(CC)CCCC(O)c1ccc(NS(C)(=O)=O)cc1
```

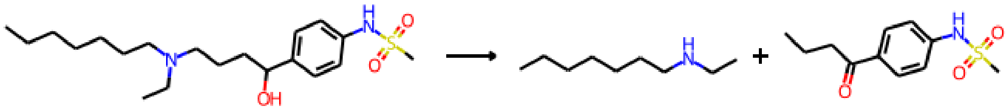

```
|Route 77|----------------------------------------------------------------------------------------------------
|Step  1|---------------------------------------------------------------------------------------------------
Retrosynthesis:
COc1ccccc1OCCNCC(O)COc1cccc2[nH]c3ccccc3c12>>COc1ccccc1OCCN.CC(O)COc1cccc2[nH]c3cccc
c3c12
Reaction:
COc1ccccc1OCCN.CC(O)COc1cccc2[nH]c3ccccc3c12>>COc1ccccc1OCCNCC(O)COc1cccc2[nH]c3cccc
c3c12
```

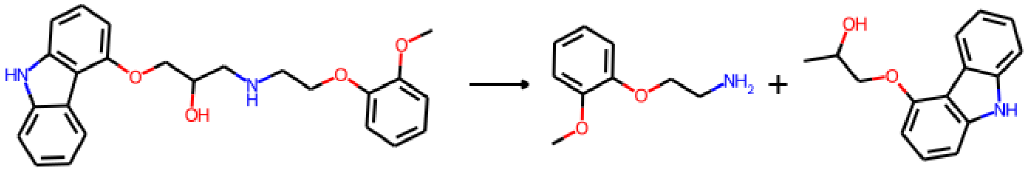

```
|Step  2|---------------------------------------------------------------------------------------------------
Retrosynthesis: CC(O)COc1cccc2[nH]c3ccccc3c12>>Oc1cccc2[nH]c3ccccc3c12.CC(C)O
Reaction: Oc1cccc2[nH]c3ccccc3c12.CC(C)O>>CC(O)COc1cccc2[nH]c3ccccc3c12
```

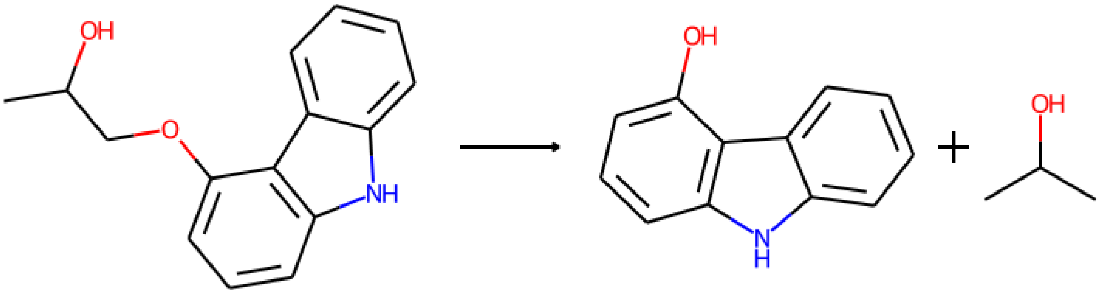

```
|Route 78|--------------------------------------------------------------------------
--------------------------
|Step  1|---------------------------------------------------------------------------
-------------------------
Retrosynthesis: CC(Cc1ccccc1)NCc1ccccc1Cl>>CCCc1ccccc1.NCc1ccccc1Cl
Reaction: CCCc1ccccc1.NCc1ccccc1Cl>>CC(Cc1ccccc1)NCc1ccccc1Cl
```

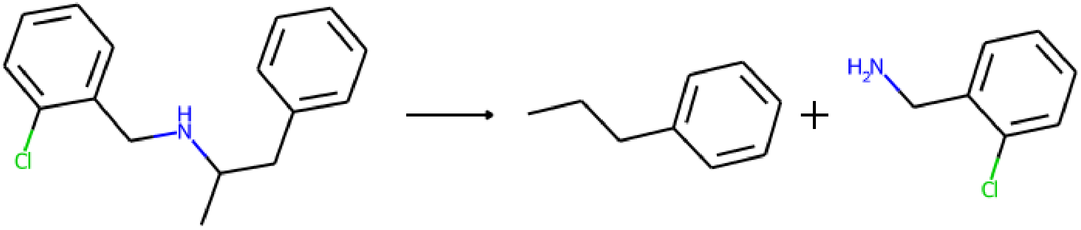

```
|Route 79|--------------------------------------------------------------------------
--------------------------
|Step  1|---------------------------------------------------------------------------
-------------------------
Retrosynthesis:
COc1cc2[nH]nnc2cc1C(=O)NC1CCN(Cc2ccccc2)CC1>>CCOC(=O)c1cc2nn[nH]c2cc1OC.NC1CCN(Cc2cc
ccc2)CC1
Reaction:
CCOC(=O)c1cc2nn[nH]c2cc1OC.NC1CCN(Cc2ccccc2)CC1>>COc1cc2[nH]nnc2cc1C(=O)NC1CCN(Cc2cc
ccc2)CC1
```

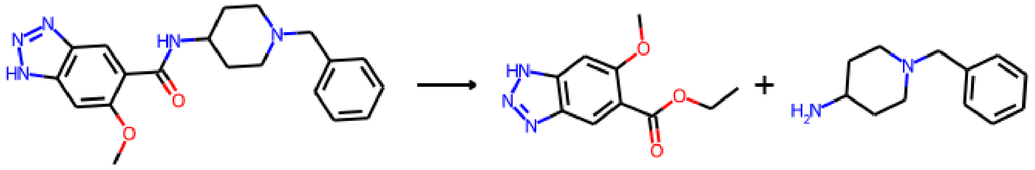


```
|Step  2|--------------------------------------------------------------------------
-------------------------
Retrosynthesis: CCOC(=O)c1cc2nn[nH]c2cc1OC>>CCOC(=O)c1ccc(NC(C)=O)cc1OC.NN
Reaction: CCOC(=O)c1ccc(NC(C)=O)cc1OC.NN>>CCOC(=O)c1cc2nn[nH]c2cc1OC
```

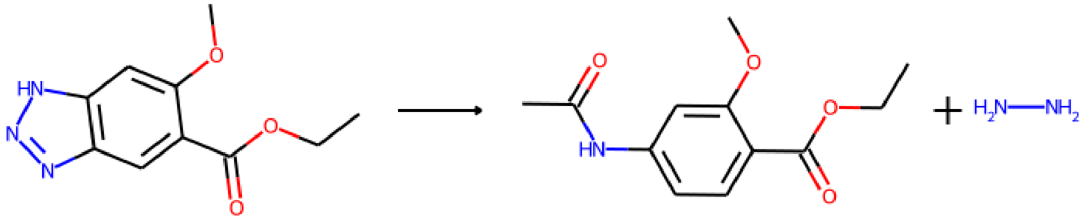


```
|Route 80|-------------------------------------------------------------------------
--------------------------
|Step  1|--------------------------------------------------------------------------
-------------------------
Retrosynthesis: CCCCN1CCCC1=Nc1ccc(Cl)c(Cl)c1>>Nc1ccc(Cl)c(Cl)c1.CCCC=NCCCC
Reaction: Nc1ccc(Cl)c(Cl)c1.CCCC=NCCCC>>CCCCN1CCCC1=Nc1ccc(Cl)c(Cl)c1
```

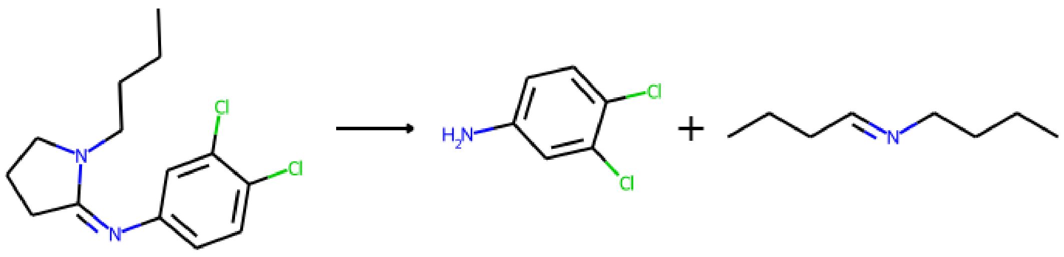


```
|Route 81|--------------------------------------------------------------------------
--------------------------
|Step  1|---------------------------------------------------------------------------
-------------------------
Retrosynthesis:
Fc1ccc(C(OCCN2CCN(CCCc3ccccc3)CC2)c2ccc(F)cc2)cc1>>Fc1ccc(C(OCCN2CCNCC2)c2ccc(F)cc2)
cc1.BrCCCc1ccccc1
Reaction:
Fc1ccc(C(OCCN2CCNCC2)c2ccc(F)cc2)cc1.BrCCCc1ccccc1>>Fc1ccc(C(OCCN2CCN(CCCc3ccccc3)CC
2)c2ccc(F)cc2)cc1
```

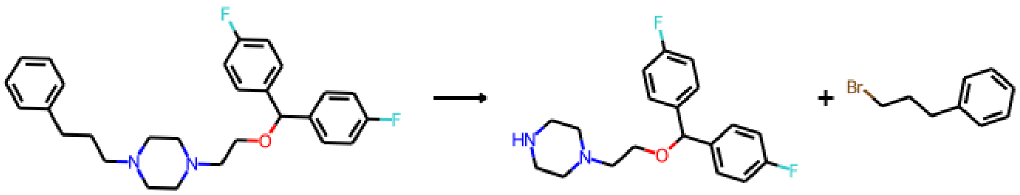


```
|Route 82|--------------------------------------------------------------------------
--------------------------
|Step  1|---------------------------------------------------------------------------
-------------------------
Retrosynthesis: CSc1ccccc1OCC(O)CNC(C)C>>CSc1ccccc1OCC(C)O.CC(C)N
Reaction: CSc1ccccc1OCC(C)O.CC(C)N>>CSc1ccccc1OCC(O)CNC(C)C
```

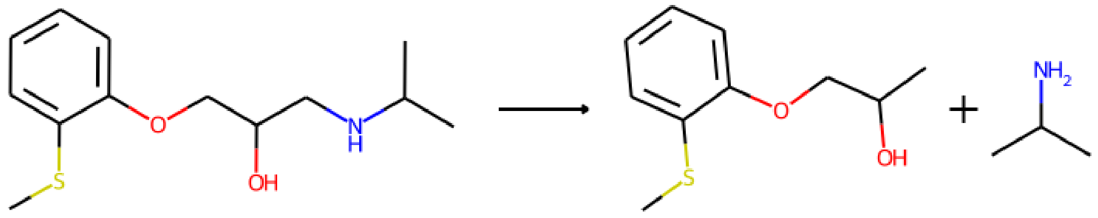

```
|Route 83|---------------------------------------------------------------------------
--------------------------
|Step  1|----------------------------------------------------------------------------
-------------------------
Retrosynthesis: CC(C)(CO)NCC(O)c1ccccc1>>CC(C)(N)CO.OC(CCl)c1ccccc1
Reaction: CC(C)(N)CO.OC(CCl)c1ccccc1>>CC(C)(CO)NCC(O)c1ccccc1
```

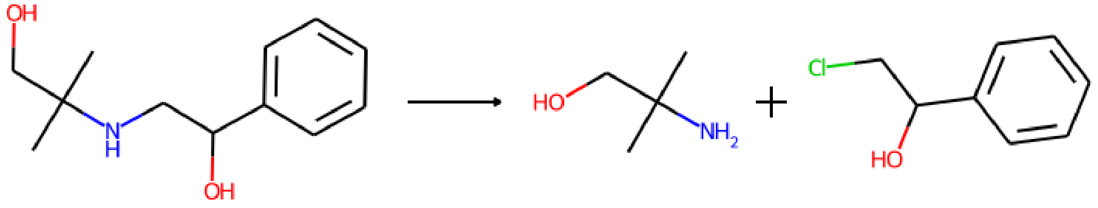

```
|Route 84|---------------------------------------------------------------------------
--------------------------
|Step  1|----------------------------------------------------------------------------
-------------------------
Retrosynthesis: Cc1cccc(C)c1NC(=O)C(C)N>>Cc1cccc(C)c1NC(=O)C(C)N1C(=O)c2ccccc2C1=O
Reaction: Cc1cccc(C)c1NC(=O)C(C)N1C(=O)c2ccccc2C1=O>>Cc1cccc(C)c1NC(=O)C(C)N
```

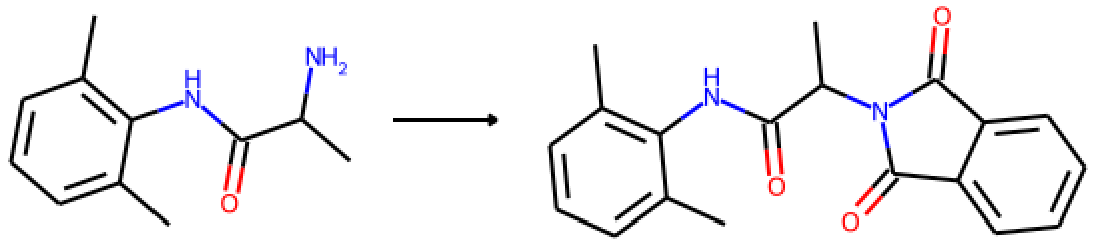

```
|Route 85|---------------------------------------------------------------------------
--------------------------
|Step  1|---------------------------------------------------------------------------
-------------------------
Retrosynthesis: CCCCOc1c(N)cccc1C(=O)OCCN(CC)CC>>CCCCOc1c(N)cccc1C(=O)OC(Cl)CN(CC)CC
Reaction: CCCCOc1c(N)cccc1C(=O)OC(Cl)CN(CC)CC>>CCCCOc1c(N)cccc1C(=O)OCCN(CC)CC
```

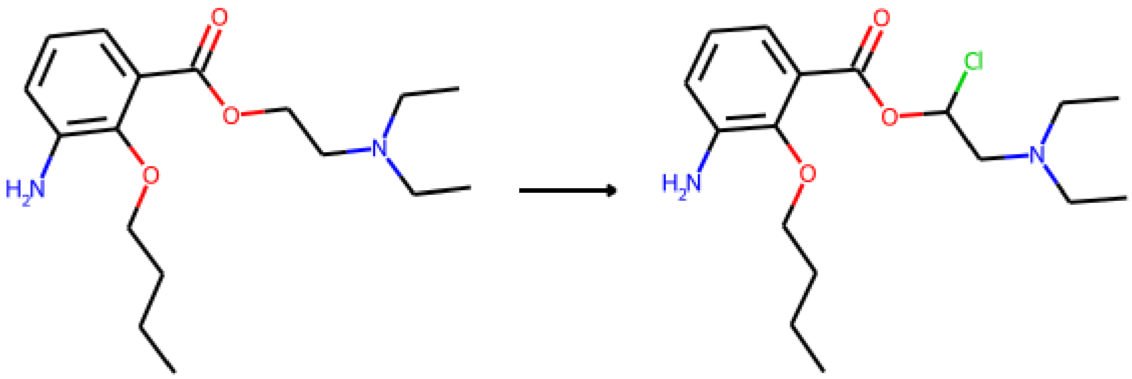


```
|Step  2|---------------------------------------------------------------------------
-------------------------
Retrosynthesis: CCCCOc1c(N)cccc1C(=O)OC(Cl)CN(CC)CC>>CCCCOc1c(N)cccc1C(=O)OC(Cl)
(Cl)CN(CC)CC
Reaction: CCCCOc1c(N)cccc1C(=O)OC(Cl)
(Cl)CN(CC)CC>>CCCCOc1c(N)cccc1C(=O)OC(Cl)CN(CC)CC
```

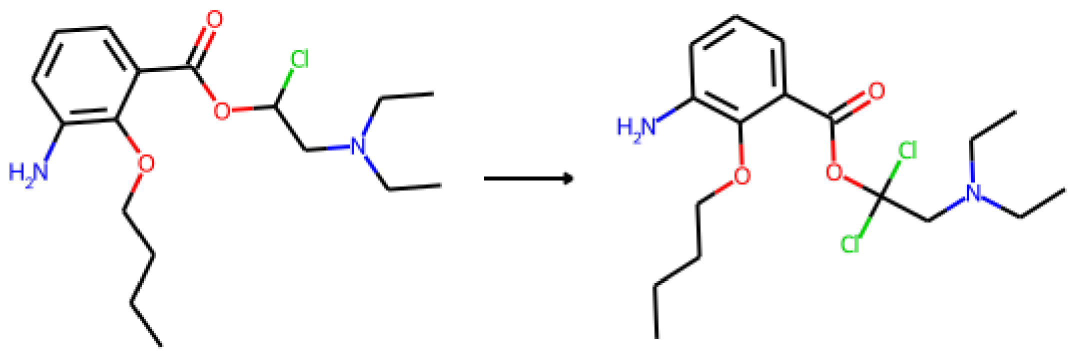

```
|Step  3|-----------------------------------------------------------------------------
-------------------------
Retrosynthesis: CCCCOc1c(N)cccc1C(=O)OC(Cl)(Cl)CN(CC)CC>>CCN(CC)CC(Cl)
(Cl)Cl.CCCCOc1c(N)cccc1C(=O)O
Reaction: CCN(CC)CC(Cl)(Cl)Cl.CCCCOc1c(N)cccc1C(=O)O>>CCCCOc1c(N)cccc1C(=O)OC(Cl)
(Cl)CN(CC)CC
```

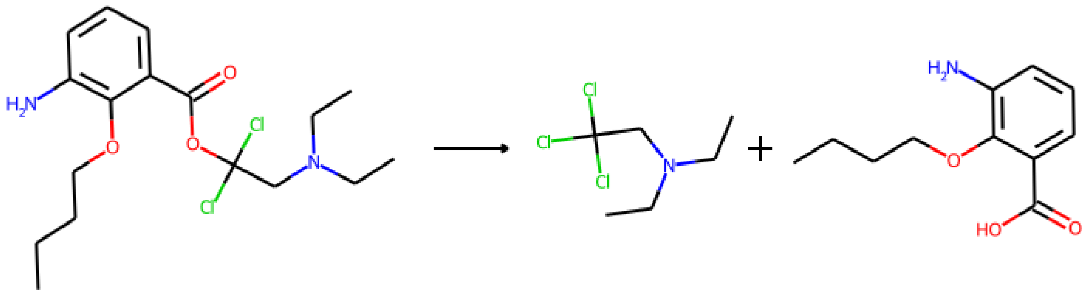

```
|Step  4|-----------------------------------------------------------------------------
-------------------------
Retrosynthesis: CCN(CC)CC(Cl)(Cl)Cl>>CCNCC.ClCC(Cl)(Cl)Cl
Reaction: CCNCC.ClCC(Cl)(Cl)Cl>>CCN(CC)CC(Cl)(Cl)Cl
```

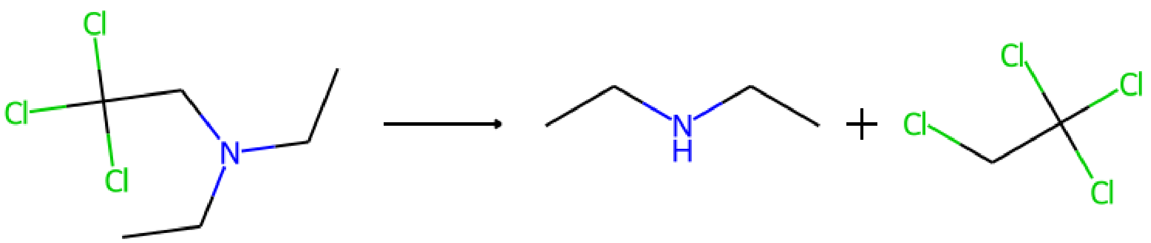

```
|Route 86|----------------------------------------------------------------------------------------------------
|Step  1|---------------------------------------------------------------------------------------------------
Retrosynthesis:
O=c1[nH]c2ccccc2c(=O)n1CCCN1CCN(c2ccccc2)CC1>>O=c1[nH]c2ccccc2c(=O)n1CCCCl.c1ccc(N2CCNCC2)cc1
Reaction:
O=c1[nH]c2ccccc2c(=O)n1CCCCl.c1ccc(N2CCNCC2)cc1>>O=c1[nH]c2ccccc2c(=O)n1CCCN1CCN(c2ccccc2)CC1
```

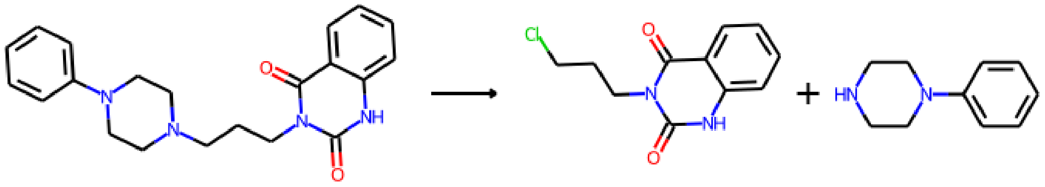

```
|Step  2|---------------------------------------------------------------------------------------------------
Retrosynthesis: O=c1[nH]c2ccccc2c(=O)n1CCCCl>>CCCn1c(=O)[nH]c2ccccc2c1=O.Cl
Reaction: CCCn1c(=O)[nH]c2ccccc2c1=O.Cl>>O=c1[nH]c2ccccc2c(=O)n1CCCCl
```

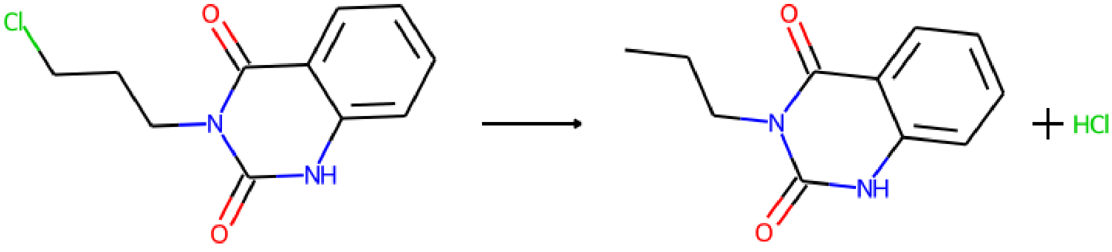


```
|Route 87|-------------------------------------------------------------------------------------------------------
|Step  1|--------------------------------------------------------------------------------------------------------
Retrosynthesis:
CCN1CCC(=C2c3ccccc3CCc3ccccc32)C1C>>CCN1CCC(C2c3ccccc3CCc3ccccc32)C1C
Reaction: CCN1CCC(C2c3ccccc3CCc3ccccc32)C1C>>CCN1CCC(=C2c3ccccc3CCc3ccccc32)C1C
```

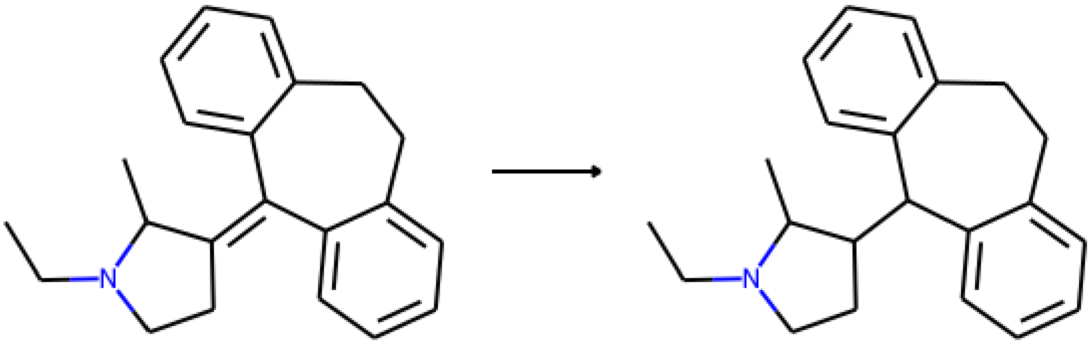


```
|Step  2|--------------------------------------------------------------------------------------------------------
Retrosynthesis:
CCN1CCC(C2c3ccccc3CCc3ccccc32)C1C>>CCN1CCC(C2c3ccccc3CCc3ccccc32)C1(C)N(C)C
Reaction:
CCN1CCC(C2c3ccccc3CCc3ccccc32)C1(C)N(C)C>>CCN1CCC(C2c3ccccc3CCc3ccccc32)C1C
```

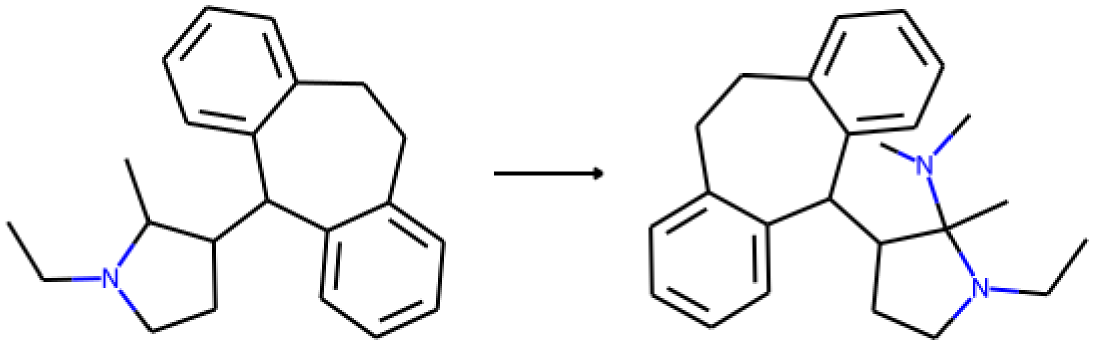

```
|Step  3|---------------------------------------------------------------------------------------------------
Retrosynthesis:
CCN1CCC(C2c3ccccc3CCc3ccccc32)C1(C)N(C)C>>CC(=O)N1CCC(C2c3ccccc3CCc3ccccc32)C1(C)N(C)C
Reaction:
CC(=O)N1CCC(C2c3ccccc3CCc3ccccc32)C1(C)N(C)C>>CCN1CCC(C2c3ccccc3CCc3ccccc32)C1(C)N(C)C
```

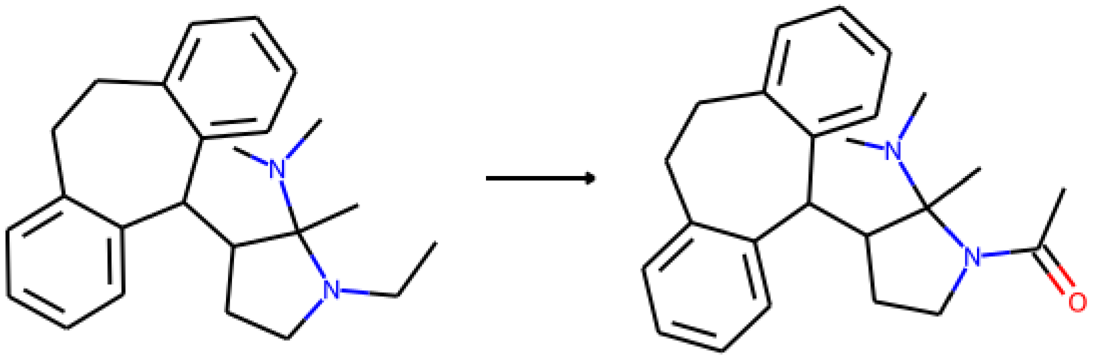

```
|Step  4|---------------------------------------------------------------------------------------------------
Retrosynthesis:
CC(=O)N1CCC(C2c3ccccc3CCc3ccccc32)C1(C)N(C)C>>c1ccc2c(c1)CCc1ccccc1C2.CC(=O)N1CCCC1C.CNC
Reaction:
c1ccc2c(c1)CCc1ccccc1C2.CC(=O)N1CCCC1C.CNC>>CC(=O)N1CCC(C2c3ccccc3CCc3ccccc32)C1(C)N(C)C
```

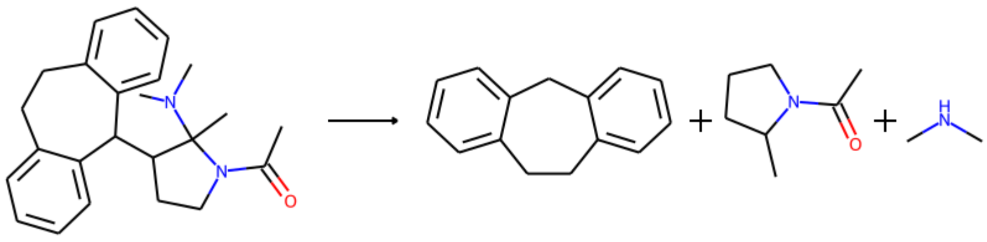

```
|Route 88|--------------------------------------------------------------------------------------------------------
|Step  1|--------------------------------------------------------------------------------------------------------
Retrosynthesis: C=CCC1(C(C)C)C(=O)NC(=O)NC1=O>>CC(C)C1C(=O)NC(=O)NC1=O.C=CC
Reaction: CC(C)C1C(=O)NC(=O)NC1=O.C=CC>>C=CCC1(C(C)C)C(=O)NC(=O)NC1=O
```

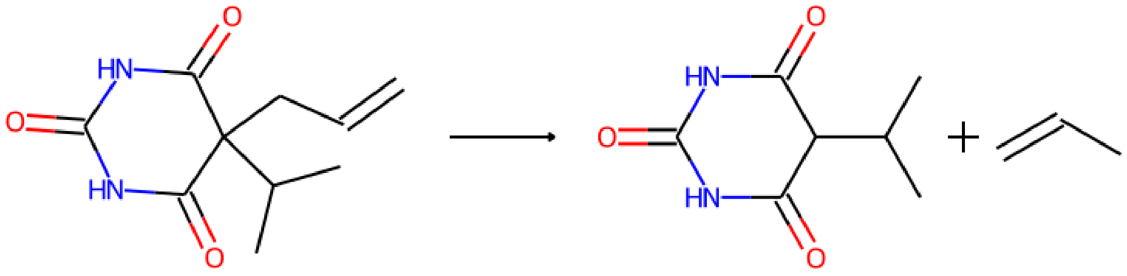

```
|Route 89|--------------------------------------------------------------------------------------------------------
|Step  1|--------------------------------------------------------------------------------------------------------
Retrosynthesis: CCC(C)(CN(C)C)OC(=O)c1ccccc1>>CCC(C)(OC(=O)c1ccccc1)C(=O)N(C)C
Reaction: CCC(C)(OC(=O)c1ccccc1)C(=O)N(C)C>>CCC(C)(CN(C)C)OC(=O)c1ccccc1
```

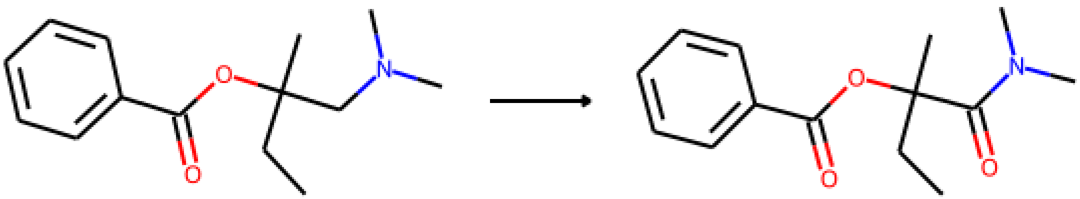

```
|Step  2|---------------------------------------------------------------------------
-------------------------
Retrosynthesis: CCC(C)(OC(=O)c1ccccc1)C(=O)N(C)C>>CCC(C)(O)C(=O)N(C)C.O=Cc1ccccc1
Reaction: CCC(C)(O)C(=O)N(C)C.O=Cc1ccccc1>>CCC(C)(OC(=O)c1ccccc1)C(=O)N(C)C
```

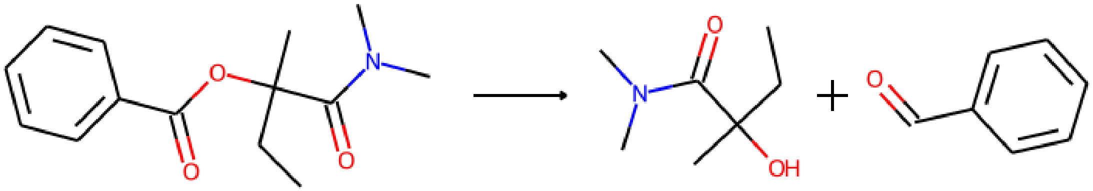

```
|Step  3|---------------------------------------------------------------------------
-------------------------
Retrosynthesis: CCC(C)(O)C(=O)N(C)C>>CCOC(C)OC(C)(CC)C(=O)N(C)C
Reaction: CCOC(C)OC(C)(CC)C(=O)N(C)C>>CCC(C)(O)C(=O)N(C)C
```

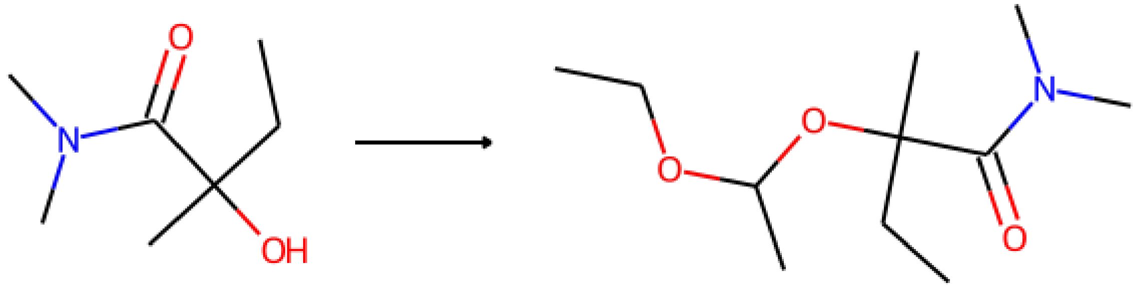

```
|Step  4|-------------------------------------------------------------------------------
-------------------------
Retrosynthesis: CCOC(C)OC(C)(CC)C(=O)N(C)C>>CC.CCOCC.CC(O)C(=O)N(C)C
Reaction: CC.CCOCC.CC(O)C(=O)N(C)C>>CCOC(C)OC(C)(CC)C(=O)N(C)C
```

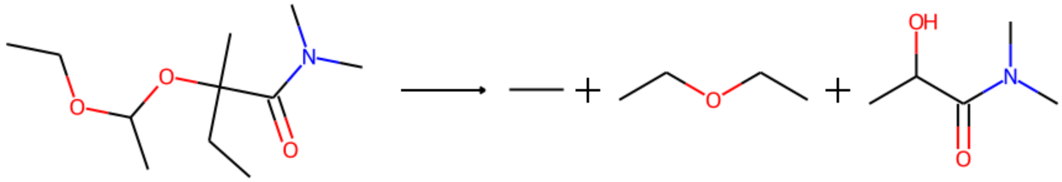

```
|Route 90|------------------------------------------------------------------------------
--------------------------
|Step  1|-------------------------------------------------------------------------------
-------------------------
Retrosynthesis:
CC(Cc1ccc(O)cc1)NCC(O)c1cc(O)cc(O)c1>>CC(=O)Cc1ccc(O)cc1.NCC(O)c1cc(O)cc(O)c1
Reaction:
CC(=O)Cc1ccc(O)cc1.NCC(O)c1cc(O)cc(O)c1>>CC(Cc1ccc(O)cc1)NCC(O)c1cc(O)cc(O)c1
```

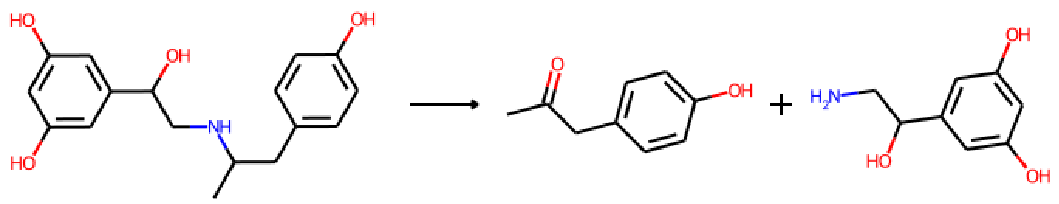

```
|Route 91|--------------------------------------------------------------------------------------------
|Step  1|---------------------------------------------------------------------------------------------
Retrosynthesis: OC1(c2ccc(Cl)c(C(F)(F)F)c2)CCN(CCCC(c2ccc(F)cc2)c2ccc(F)cc2)CC1>>OC1(c2ccc(Cl)c(C(F)(F)F)c2)CCNCC1.Fc1ccc(C(CCCCl)c2ccc(F)cc2)cc1
Reaction: OC1(c2ccc(Cl)c(C(F)(F)F)c2)CCNCC1.Fc1ccc(C(CCCCl)c2ccc(F)cc2)cc1>>OC1(c2ccc(Cl)c(C(F)(F)F)c2)CCN(CCCC(c2ccc(F)cc2)c2ccc(F)cc2)CC1
```

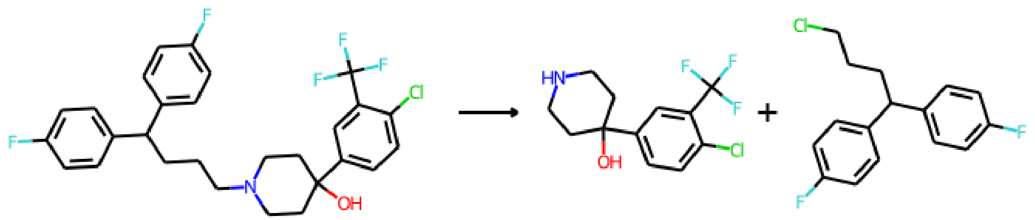

```
|Route 92|--------------------------------------------------------------------------------------------
|Step  1|---------------------------------------------------------------------------------------------
Retrosynthesis: NCCN>>N#CNCCN
Reaction: N#CNCCN>>NCCN
```

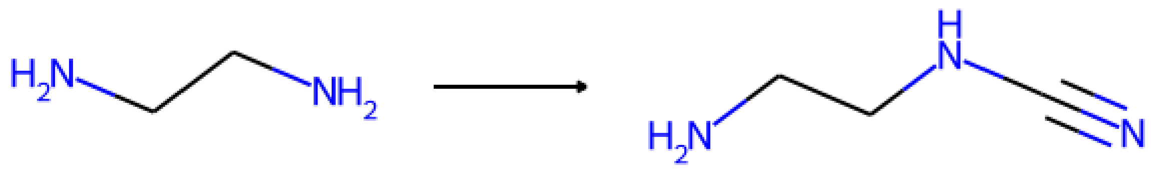

```
|Route 93|--------------------------------------------------------------------------
--------------------------
|Step  1|--------------------------------------------------------------------------
-------------------------
Retrosynthesis:
CCc1cccc(N(C)C(=N)Nc2cccc3ccccc23)c1>>Nc1cccc2ccccc12.CC(=O)c1cccc(N(C)C)c1.N
Reaction:
Nc1cccc2ccccc12.CC(=O)c1cccc(N(C)C)c1.N>>CCc1cccc(N(C)C(=N)Nc2cccc3ccccc23)c1
```

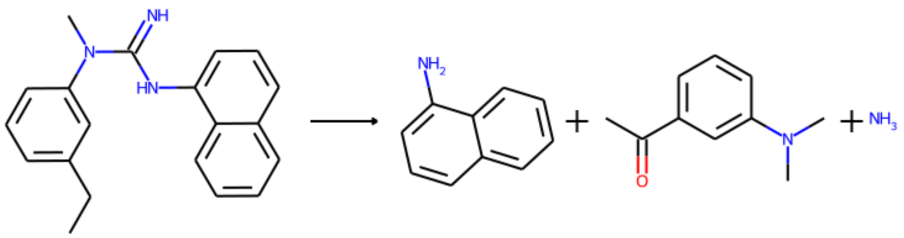

```
|Route 94|--------------------------------------------------------------------------
--------------------------
|Step  1|--------------------------------------------------------------------------
-------------------------
Retrosynthesis: CCc1nncn1C1CCCCC1>>CCC(N):N(C#N)C1CCCCC1
Reaction: CCC(N):N(C#N)C1CCCCC1>>CCc1nncn1C1CCCCC1
```

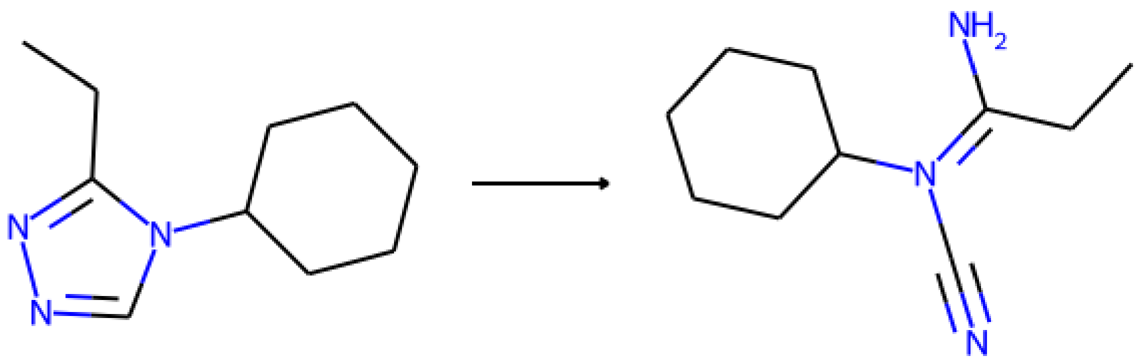

```
|Step  2|--------------------------------------------------------------------------------------------------------
Retrosynthesis: CCC(N):N(C#N)C1CCCCC1>>CCCOCC.N#CNC1CCCCC1.N
Reaction: CCCOCC.N#CNC1CCCCC1.N>>CCC(N):N(C#N)C1CCCCC1
```

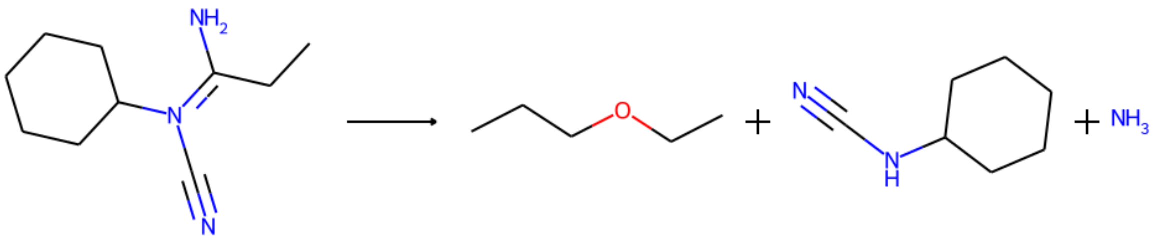

```
|Route 95|--------------------------------------------------------------------------------------------------------
|Step  1|--------------------------------------------------------------------------------------------------------
Retrosynthesis:
COC(=O)C1=C(C)NC(C)=C(C(=O)OC)C1c1ccccc1OC(F)F>>COC(=O)C(Cc1ccccc1OC(F)F)=C(C)N.CC#CC(=O)OC
Reaction:
COC(=O)C(Cc1ccccc1OC(F)F)=C(C)N.CC#CC(=O)OC>>COC(=O)C1=C(C)NC(C)=C(C(=O)OC)C1c1ccccc1OC(F)F
```

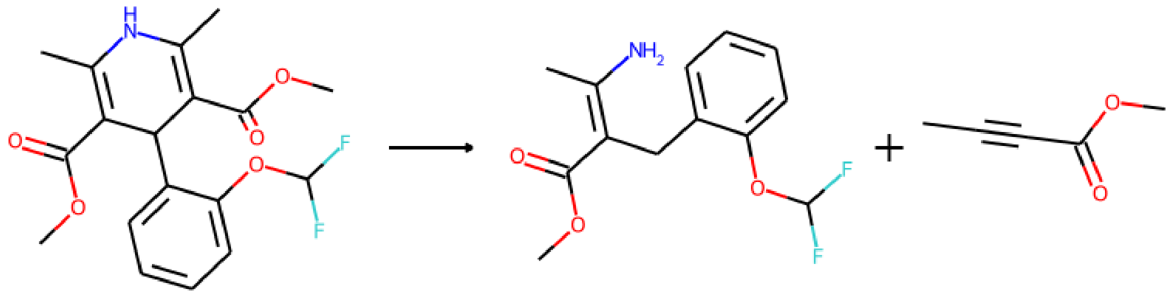

```
|Step  2|-------------------------------------------------------------------------
--------------------------
Retrosynthesis: COC(=O)C(Cc1ccccc1OC(F)F)=C(C)N>>CC(N)=O.COC(=O)CCc1ccccc1OC(F)F
Reaction: CC(N)=O.COC(=O)CCc1ccccc1OC(F)F>>COC(=O)C(Cc1ccccc1OC(F)F)=C(C)N
```

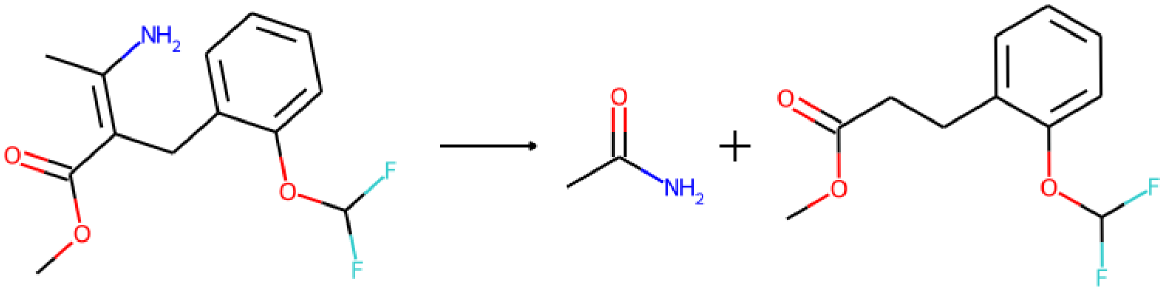

```
|Route 96|------------------------------------------------------------------------
---------------------------
|Step  1|-------------------------------------------------------------------------
--------------------------
Retrosynthesis: CC1CC(OC(=O)c2ccccc2O)CC(C)(C)C1>>COC(=O)Oc1ccccc1C(=O)OC1CC(C)CC(C)
(C)C1
Reaction: COC(=O)Oc1ccccc1C(=O)OC1CC(C)CC(C)(C)C1>>CC1CC(OC(=O)c2ccccc2O)CC(C)(C)C1
```

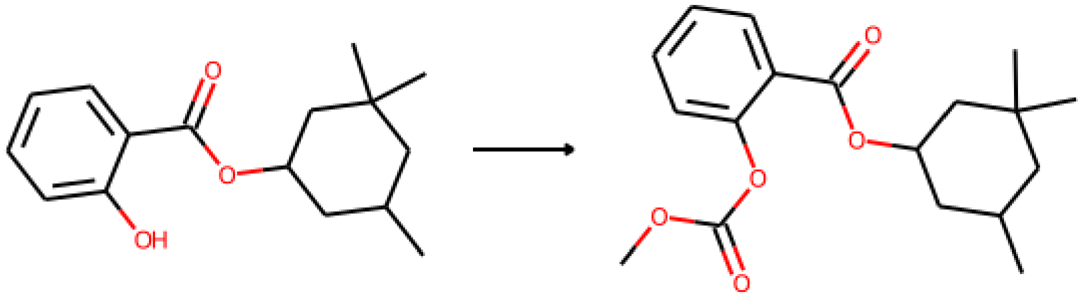

```
|Step  2|-------------------------------------------------------------------------
-------------------------
Retrosynthesis: COC(=O)Oc1ccccc1C(=O)OC1CC(C)CC(C)(C)C1>>CC1CCCC(C)
(C)C1.COC(=O)Oc1ccccc1C(=O)O
Reaction: CC1CCCC(C)(C)C1.COC(=O)Oc1ccccc1C(=O)O>>COC(=O)Oc1ccccc1C(=O)OC1CC(C)CC(C)
(C)C1
```

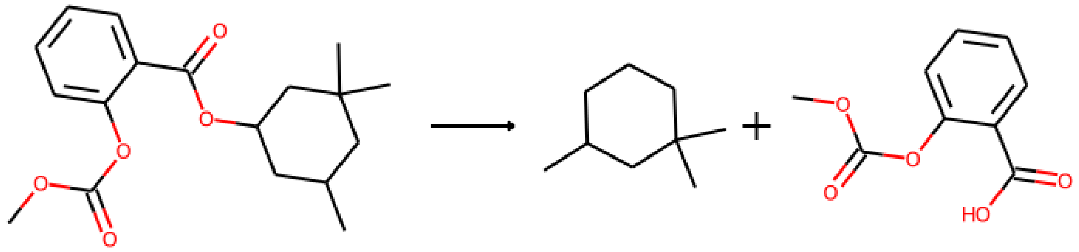

```
|Route 97|------------------------------------------------------------------------
--------------------------
|Step  1|-------------------------------------------------------------------------
-------------------------
Retrosynthesis: OCC(Br)(Br)Br>>O=S(=O)(O)OCC(Br)(Br)Br
Reaction: O=S(=O)(O)OCC(Br)(Br)Br>>OCC(Br)(Br)Br
```

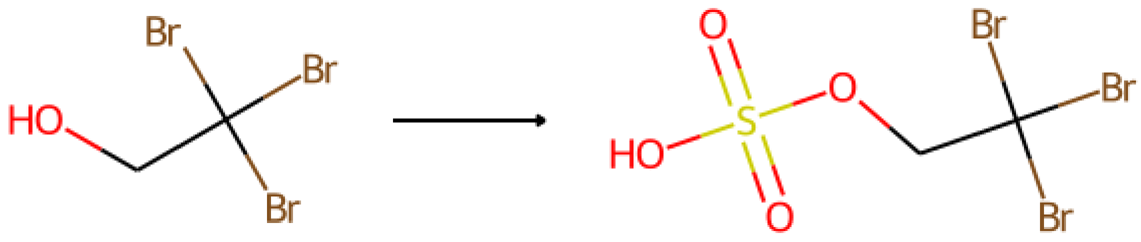

```
|Step  2|----------------------------------------------------------------------------
-------------------------
Retrosynthesis: O=S(=O)(O)OCC(Br)(Br)Br>>O=C(O)C(Br)(Br)Br.O=[SH](=O)O
Reaction: O=C(O)C(Br)(Br)Br.O=[SH](=O)O>>O=S(=O)(O)OCC(Br)(Br)Br
```

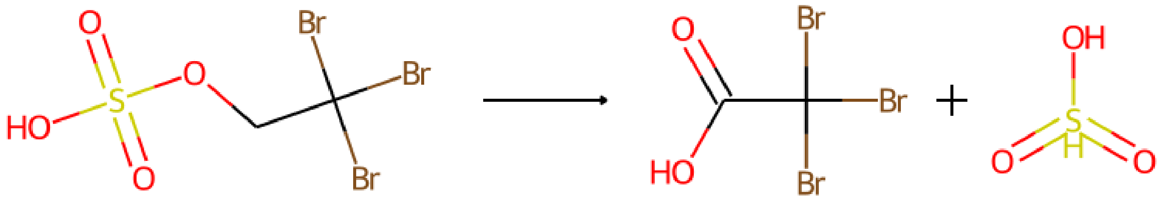

```
|Route 98|---------------------------------------------------------------------------
--------------------------
|Step  1|----------------------------------------------------------------------------
-------------------------
Retrosynthesis: CC(C)NCC(O)c1cc(O)cc(O)c1>>CC(C)N(CC(O)c1cc(O)cc(O)c1)C(c1ccccc1)
(c1ccccc1)c1ccccc1
Reaction: CC(C)N(CC(O)c1cc(O)cc(O)c1)C(c1ccccc1)
(c1ccccc1)c1ccccc1>>CC(C)NCC(O)c1cc(O)cc(O)c1
```

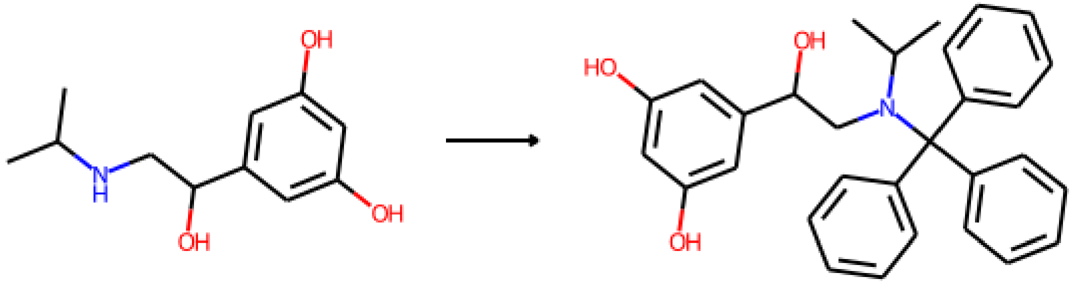

```
|Step  2|--------------------------------------------------------------------------------
-------------------------
Retrosynthesis: CC(C)N(CC(O)c1cc(O)cc(O)c1)C(c1ccccc1)
(c1ccccc1)c1ccccc1>>Oc1cc(O)cc(C(O)CNC(c2ccccc2)(c2ccccc2)c2ccccc2)c1.CC(C)=O
Reaction: Oc1cc(O)cc(C(O)CNC(c2ccccc2)
(c2ccccc2)c2ccccc2)c1.CC(C)=O>>CC(C)N(CC(O)c1cc(O)cc(O)c1)C(c1ccccc1)
(c1ccccc1)c1ccccc1
```

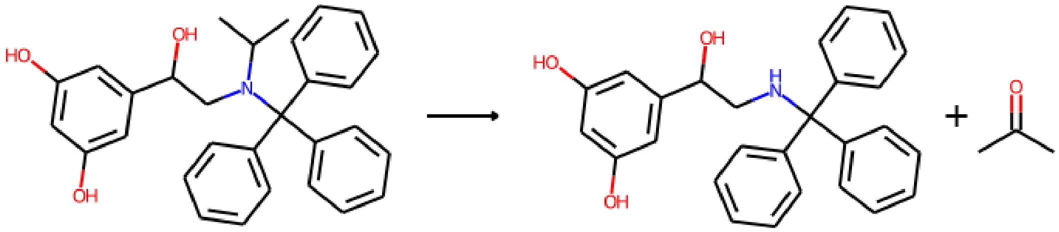

```
|Step  3|--------------------------------------------------------------------------------
-------------------------
Retrosynthesis: Oc1cc(O)cc(C(O)CNC(c2ccccc2)
(c2ccccc2)c2ccccc2)c1>>NCC(O)c1cc(O)cc(O)c1.ClC(c1ccccc1)(c1ccccc1)c1ccccc1
Reaction: NCC(O)c1cc(O)cc(O)c1.ClC(c1ccccc1)
(c1ccccc1)c1ccccc1>>Oc1cc(O)cc(C(O)CNC(c2ccccc2)(c2ccccc2)c2ccccc2)c1
```

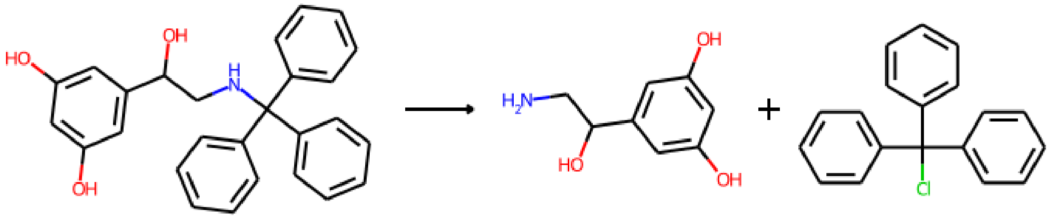

```
|Route 99|------------------------------------------------------------------------------
--------------------------
|Step  1|------------------------------------------------------------------------------
-------------------------
Retrosynthesis: CC(=O)Oc1ccccc1C(=O)OC(COC(=O)C(C)(C)Oc1ccc(Cl)cc1)COC(=O)C(C)
(C)Oc1ccc(Cl)cc1>>CC(=O)Oc1ccccc1C=O.CC(C)(Oc1ccc(Cl)cc1)C(=O)OCC(O)COC(=O)C(C)
(C)Oc1ccc(Cl)cc1
Reaction: CC(=O)Oc1ccccc1C=O.CC(C)(Oc1ccc(Cl)cc1)C(=O)OCC(O)COC(=O)C(C)
(C)Oc1ccc(Cl)cc1>>CC(=O)Oc1ccccc1C(=O)OC(COC(=O)C(C)(C)Oc1ccc(Cl)cc1)COC(=O)C(C)
(C)Oc1ccc(Cl)cc1
```

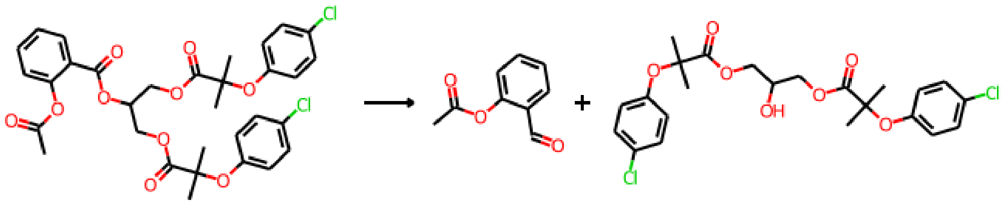

```
|Step  2|------------------------------------------------------------------------------
-------------------------
Retrosynthesis: CC(C)(Oc1ccc(Cl)cc1)C(=O)OCC(O)COC(=O)C(C)(C)Oc1ccc(Cl)cc1>>CC(C)
(Oc1ccc(Cl)cc1)C(=O)O.CC(O)COC(=O)C(C)(C)Oc1ccc(Cl)cc1
Reaction: CC(C)(Oc1ccc(Cl)cc1)C(=O)O.CC(O)COC(=O)C(C)(C)Oc1ccc(Cl)cc1>>CC(C)
(Oc1ccc(Cl)cc1)C(=O)OCC(O)COC(=O)C(C)(C)Oc1ccc(Cl)cc1
```

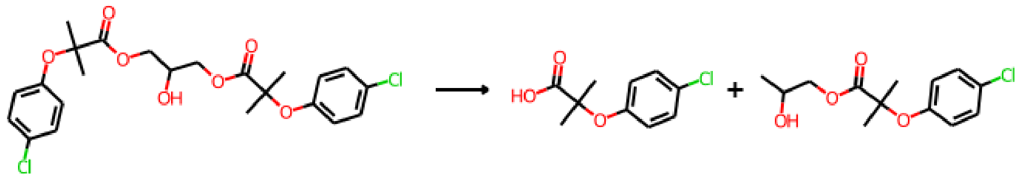


```
|Step  3|--------------------------------------------------------------------------
-------------------------
Retrosynthesis: CC(O)COC(=O)C(C)(C)Oc1ccc(Cl)cc1>>CC(O)CO.CC(C)(C=O)Oc1ccc(Cl)cc1
Reaction: CC(O)CO.CC(C)(C=O)Oc1ccc(Cl)cc1>>CC(O)COC(=O)C(C)(C)Oc1ccc(Cl)cc1
```

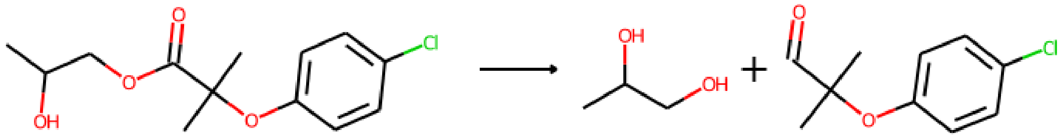


```
|Route 100|------------------------------------------------------------------------
---------------------------
|Step  1|--------------------------------------------------------------------------
-------------------------
Retrosynthesis: Cc1nc(C)c(N(C)C)nc1C>>CNC.Cc1cnc(C)c(C)n1
Reaction: CNC.Cc1cnc(C)c(C)n1>>Cc1nc(C)c(N(C)C)nc1C
```

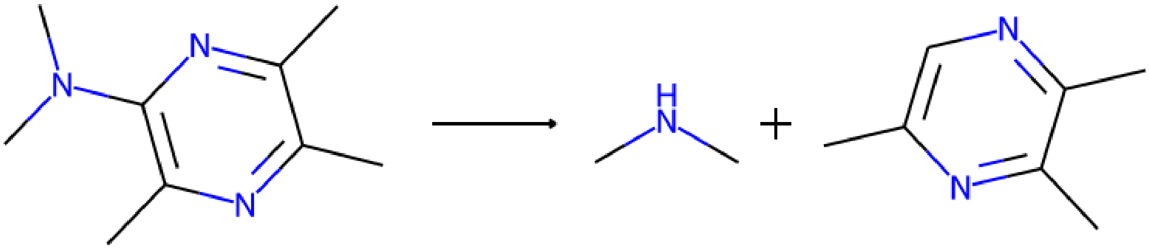


```
|Route 101|----------------------------------------------------------------------------
---------------------------
|Step  1|------------------------------------------------------------------------------
-------------------------
Retrosynthesis: N=C(N)NCC1COC2(CCCCC2)O1>>N=C=N.NCC1COC2(CCCCC2)O1
Reaction: N=C=N.NCC1COC2(CCCCC2)O1>>N=C(N)NCC1COC2(CCCCC2)O1
```

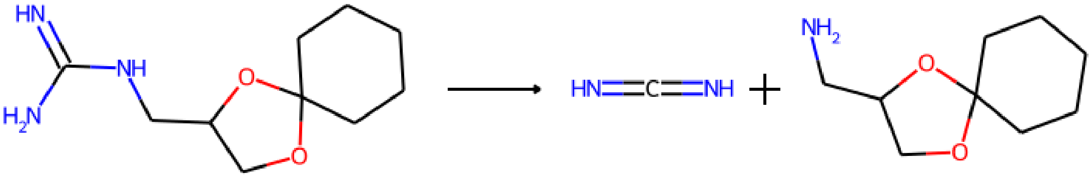


```
step_count
```

```
Counter({3: 13, 2: 28, 1: 53, 5: 2, 4: 5})
```
