# Supplementary material for: Retrosynthesis prediction with an interpretable deep-learning framework based on molecular assembly tasks
Source: Nat Commun. 2023 Oct 3;14:6155. doi: 10.1038/s41467-023-41698-5 (PMC10547708; doi:10.1038/s41467-023-41698-5)
Supplement: Supplementary file 2 — Peer Review File [file 41467_2023_41698_MOESM2_ESM.pdf]

nature portfolio

Peer Review File

Retrosynthesis prediction with an interpretable deep-learning framework based on molecular assembly tasks

Open Access This file is licensed under a Creative Commons Attribution 4.0 International License, which permits use, sharing, adaptation, distribution and reproduction in any medium or format, as long as you give appropriate credit to the original author(s) and the source, provide a link to the Creative Commons license, and indicate if changes were made. In the cases where the authors are anonymous, such as is the case for the reports of anonymous peer reviewers, author attribution should be to 'Anonymous Referee' followed by a clear attribution to the source work. The images or other third party material in this file are included in the article's Creative Commons license, unless indicated otherwise in a credit line to the material. If material is not included in the article's Creative Commons license and your intended use is not permitted by statutory regulation or exceeds the permitted use, you will need to obtain permission directly from the copyright holder. To view a copy of this license, visit http://creativecommons.org/licenses/by/4.0/.

REVIEWER COMMENTS

Reviewer #1 (Remarks to the Author):

The manuscript by Wang et al. presents RetroExplainer, a framework for single-step retrosynthesis, which uses a semi-template/reaction-center based two-stage formulation similar as in GraphRetro. Its main model architectures are based on a graph-Transformer, with additional performance engineering techniques such as contrastive learning and adaptive adjustment of task weights to boost top-k accuracies. However, due to the following major concerns, I recommend rejecting this manuscript.

Major issues with contextualization and motivation:

The summary of related works in the introduction is over-simplifying, if not misleading. The authors do not seem to know exactly what some cited works are doing. As a few examples,

- Lines 43-49: "From the perspective of molecule representations", there are fingerprint-based representations in addition to the mentioned sequence-based and graph-based representations, such as used in Segler et al. and RetroSim. The authors cite both but do not seem to be aware that they use fingerprints and are neither sequence nor graph-based. The authors' definitions of sequence-based approaches as encoder-decoder-based translation, and of graph-based approaches as graph changes, are both problematic. First, this does not include the important family of template-based methods at all. Secondly, there are graph-based translation approaches such as GET and Graph2SMILES, both of which the authors cite too.

- Line 58: "Segler et al." is cited as augmenting Transformers. This is not true. It is one of the most well-known works for template-based retrosynthesis and has nothing to do with Transformers.

- Line 66: (Sequence approaches are) "not guaranteed semantically valid". The authors cite SELFIES but do not seem to know that SELFIES is 100% valid by definition.

- Lines 75-76: "Jin et al. propose WLDN … for retrosynthetic graph learning." This is not true. WLDN was designed for reaction outcome prediction, not retrosynthesis.

The authors claim three intrinsic problems with existing approaches thereafter, but all three are questionable in my opinion.

- Lines 91-95: The authors claim that both sequence and graph approaches are constrained in feature representation learning, not knowing that graph-Transformers similar to their proposed architecture have already been used in GET, Graph2SMILES and GTA.

- Lines 95-97: The authors question the explainability of existing methods, but without discussing template-based methods which are the most interpretable approaches (since templates can be linked to literature precedents). Also, the proposed framework is not fundamentally different from existing semi-template methods.

- Lines 97-99: The authors question the accessibility of reactants from single-step retrosynthesis, which really questions this very work too. There is a single paragraph describing multi-step extension, yet without addressing the accessibility question.

Major issues with results:

- According to the associated codes (https://github.com/wangyu-sd/RetroExplainer. I have to look up the code myself as the link provided in the manuscript gives 404), the featurization is done with the "smile_to_mol_info()" function, but it does not canonicalize the test SMILES with atom mappings removed. This will lead to information leak [1-3] that has spuriously boosted test accuracy (by up to 15% for RetroXpert). Without proper canonicalization, any results with RetroExplainer on USPTO_50K are questionable.

- Line 185: RetroExplainer results are compared with "runner-up Transformer-based method G2GT". However, from Table 1, the runner-up is R-SMILES.

- Lines 461-474: The authors extend RetroExplainer for multi-step planning but is missing lots of details. For example, is the value network for Retro* retrained? How do you do the "literature search" and how are "similar reactions" defined? The manuscript referes to SI Section 11 but I have no idea which that is as the SI is not numbered.

Major issues with novelty:

In general, RetroExplainer seems to be a mega-amalgamation of know formulation/technique, including

- Graph-Transformers, as have been done with Graphormer, GET, GTA and Graph2SMILES

- Semi-template-based formulation, almost exactly following GraphRetro, except some additional model is trained for leaving group connection

- Joint-training of reaction center predictions and leaving group matching, as have been done in Retroformer [4].

- Contrastive learning, as have been done in GLN, Dual-TF/TB and Lin et al. [5]

The claim of energy-based and mechanism-like decision making is exaggerated. The "energies" seem to be merely probability outputs from the models, and the synthon-based formulation is in little way close to reaction mechanisms, which at least involve some electron flow [6, 7].

Major issues with presentation and grammar:

The styling needs improvement as it is disrupting at this moment. Please consider having some native English speaker to proofread the manuscript. Just a few examples:

- Line 5: Why do you abbreviate the names of the corresponding authors?

- Lines 32-33: "RetroExplainer discovers 101 pathways in which 86.9% reactions can be found similar reaction patterns." Not sure what the second part means.

- In Table 1, different methods are tagged with "T" or "G" or single/double asterisks, without any clear order. Please consider reorganizing, for example into groups of methods.

- Line 222 says the decision process is "six stages" but Line 628 says it has "five distinct actions".

- Line 293 refers to Fig 3a-c but those figures are showing totally different information from what's discussed. Plus, Figure 3 refers to some "MechRetro" model which comes from nowhere.

- Lines 387-389: the authors try to conclude based on how "discrete the distribution is", but there is no definition of what discreteness is anywhere.

- Please number the SI.

[1] https://github.com/uta-smile/RetroXpert/issues/10#issuecomment-803699582

[2] https://openreview.net/forum?id=rMbLORc8oS¬eId=7BERHx5nZDI

[3] https://openreview.net/forum?id=yGKi6deX8bX¬eId=7lnzOXMqJiy

[4] https://proceedings.mlr.press/v162/wan22a

[5] https://jcheminf.biomedcentral.com/articles/10.1186/s13321-022-00594-8

[6] https://openreview.net/forum?id=r1x4BnCqKX

[7] http://proceedings.mlr.press/v139/bi21a

Reviewer #2 (Remarks to the Author):

The manuscript is the improved version of the preprint titled “MechRetro is a chemical-mechanism-driven graph learning framework for interpretable retrosynthesis prediction and pathway planning”, published on arXiv in October 2022. The manuscript reports the intriguing achievement of the new state-of-the-art performance on several single-step retrosynthesis benchmarks. To achieve that, the authors developed a novel neural architecture for single-step retrosynthesis titled RetroExplainer inspired by graph neural networks and the Transformers. The authors give a detailed specification of the architecture and its submodules and compared it with the single-step retrosynthesis models from the literature in “reaction unknown” and “reaction known” cases. Furthermore, the authors integrated several explainability features in the model as well as coupled it with the Retro* algorithm to showcase its fitness for multi-step synthesis planning. The approach taken by the authors is promising and will be of considerable significance to the field. However, there are some points that we recommend revising.

Main concerns:

1) The volume of the manuscript is inflated by the number of the raised research questions (single-step retrosynthesis, model agnostic explainability, multi-step setting), and the subsections of the paper come in the suboptimal order, which makes it hard to follow the story.

2) While the score achieved with the model is impressive, the weaknesses of the proposed model are not highlighted. For example, the authors report that they train a model with a fixed number of possible leaving groups. Does this mean that the model will fail on a reaction featuring a rare leaving group which did not appear in the training set?

Recommendations:

References

1) Please add the comparison against Chemformer (MolBART) and Retroformer on USPTO to the relevant tables.

Unclear points

1) Line 64: The statement seems unclear and questionable. What do you mean by “implicit symbols” and “information loss”? No structural information is lost in SMILES. Could you please elaborate on it more? Could you support the statement “more computational costs” with a reference?

2) Line 91: Here as well, how do the models suffer from the loss of topological information? A SMILES string contains all the information about the topology of a molecule. Also, sequence-based approaches have been achieving a higher score than graph-based models in both product prediction (Molecular Transformer, Chemformer, etc.) and single-step retrosynthesis (R-SMILES), which demonstrates the lack of such suffering. If I am wrong, please correct me. If you agree, please consider changing the wording to make your point in this line more clear.

3) Line 99: What do you mean by "not accessible"?

4) Line 133: Please consider moving the subsection "The framework of the proposed RetroExplainer" and Fig. 1 to the "Methods" section, because it does not report any results and describes the architecture instead.

5) Line 143: "The resulting hidden molecular representations". Do you mean the learned embedding vectors of atoms and bonds or something else?

6) Line 145: What are "deep neural decision units" exactly? Are those MLPs? Please add a clarification.

7) Line 232: Please provide the pseudocode of the dynamic programming algorithm mentioned here. Otherwise, it is not clear what it does exactly.

8) Line 239-241: It is not clear how your learnable module allows you to generate explanations for predictions made by other models. This claim was not in the MechRetro preprint. The energy-based explanation described in the manuscript seems to require a sequence of graph-editing actions of five types. These types, in turn, appear to be integral to RetroExplainer and its submodules like RCP, LGM, LGC, etc. Please support your claim with an experimental demonstration. If you wouldn't be able to do that, please consider removing this claim (also from lines 114-119).

9) Line 244: Why is the word "incorrect" taken into brackets? Does this word here mean "not matching the ground truth, but correct in principle"?

10) Line 267: Please consider moving the section "Performance in USPTO-50K datasets using Tanimoto similarity splits method" right after the section "Results in USPTO benchmark datasets using the common splitting method" to improve the cohesion of the story.

11) Line 292: What does "attention bias" mean?

12) Line 293: "As illustrated in Fig.3a and 3b, the top-1 accuracy increases and decreases as the max hop increases (from 0 to 5), and it reaches its peak when the max hop equals 4." Figures 3-a and 3-b have no information about the max hop.

13) Line 339. Please consider removing the subsection "Multi-scale expressivity on edge representation" from the text. It describes Figure 4, which is hardly insightful and very hard to read. Arbitrary single examples of attention maps may or may not "explain" a model's prediction.

14) Line 382: The statement is questionable: one t-SNE plot does not guarantee that "these reaction types can further be divided into more advanced classes". The obtained t-SNE maps may not look the same when using different random seeds. Also, please specify the software used to obtain the t-SNE plots.

15) Line 400: Reaction type mutation and APEx seem to be really model agnostic. It seems that the sections in lines 400 and 417 are safe to remove from the manuscript because they are worth a separate paper. The paper raises several research questions, which makes it too long in my opinion, and it would not hurt to remove the mentioned points and elaborate more on them in a separate paper, possibly coupling them with different single-step models and formulating the requirements for these models to be compatible with APEx.

16) Line 417: Where does the APEx algorithm come from? Did the authors invent it? If not, please give an appropriate reference.

17) Line 485: Did you mean a probability distribution by $f\theta$? If yes, please consider denoting it as $p\theta$ to make it clearer.

18) Line 495: Also, synthons are a set of graphs modified by-products according to reaction centers. It seems that some words may be missing. It would be better to rephrase this line and formulate what a synthon is more clearly.

19) Line 534: Could you please provide some examples to support the claim "makes the model unable to distinguish some isomers"?

20) Line 539: Ai is said to belong to two different domains at the same time, which is impossible. Please fix the confusion in the notation.

21) Eq 2: There is no clarification in the text what dk is. While it is obvious for those who are familiar with the Transformer architecture, a broader audience might need clarification.

Typos

1) Fig 1-c: "Dynamatic Adaptive Multi-Task Learning". Did you mean "Dynamic"?

2) Fig 1-d: "Transation state". Did you mean "Transition state"?

3) Fig 1-d: "Macth". Did you mean "Match"?

4) Fig 2-c: "Energy decresed in RCP". Did you mean "decreased"?

5) Line 343: "hot maps". Did you mean "heat maps"?

6) Line 481: A dot before a comma.

7) Line 497: "by attaching leaving group". Did you mean "by attaching a leaving group"?

8) Line 577: "The expressive of RetroExplainer". Did you mean "The expressive power of RetroExplainer"?

9) Line 673: "Decent rate". Did you mean "Descent rate"?

Figures

1) Fig 1-d: What is the difference between E4 and Es? They look identical in the figure.

2) Fig 2-c: It would be beneficial for the readability to increase the contrast in the colors of decision curves, especially those that are close to each other.

3) Fig 3: Features the name "MechRetro" which is never mentioned in the manuscript.

4) Fig 3: Hard to read, seems too cluttered. Please consider splitting it into several separate figures. For example, a-i, j-k, l could be three separate figures. The latter might be better in the supplement. This will also solve the problem with the caption, which otherwise seems haphazard.

5) Fig 4 is very hard to read, and its necessity is questionable. Please consider moving it to the supplement or removing it entirely.

6) Fig 5: The legend is unclear. It is not obvious what classes are represented by numerical labels and why labels 2, 5 and 8 are missing.

7) Fig 8: Please clarify in the caption where the "evidence" (in blue) comes from.

Supplementary information

1) Features the name "MechRetro", which is never mentioned in the main text.

2) The quality of the figures is too low, which hurts readability.

Code

1) The github link provided in the manuscript leads to a page that does not exist. However, it seems that the repository associated with the MechRetro preprint now contains the code for RetroExplainer. Please double-check the links and ensure consistency between the names "MechRetro" and "RetroExplainer" in the repository.

2) Please consider adding a Jupyter notebook demonstrating the usage of the model.

3) Please make sure that the repository contains all the code to reproduce the figures from the manuscript that were generated by matplotlib.

4) Please add an environment file (environment.yml) which lists all the packages installed in the authors' environment with versions. Otherwise, the reproduction of the environment the authors have used can become impossible after some time.

Reviewer #3 (Remarks to the Author):

Overall, this is an excellent paper with noteworthy results. The authors present their RetroExplainer model, which provides transparent and interpretable retrosynthesis predictions. They claim it can offer post-hoc explanations and re-rank predictions made by other models, regardless of the model used. Furthermore, they introduce three new highly effective deep learning modules: MSMS-GT for molecular representation learning, DAMT for balanced multi-task learning, and SACL for fast graph-level contrastive learning. The effectiveness of their approach is demonstrated through experiments on several benchmark datasets.

The paper also features a thorough experimental analysis. The work is significant, as it proposes a novel, comprehensive, and effective technical framework that leverages chemical knowledge in model design. The paper presents many innovative designs.

Regarding the primary purpose of the review:

- The noteworthy results include the RetroExplainer model and the introduction of three new deep learning modules (MSMS-GT, DAMT, and SACL).

- The work will be of significance to the field and related fields, as it presents a novel framework and innovative designs that effectively utilize chemical knowledge.

- The work supports the conclusions and claims, as demonstrated by the experiments conducted on benchmark datasets.

- There do not appear to be any flaws in the data analysis, interpretation, or conclusions.

- The methodology is sound, and the work meets the expected standards in the field.

- The methods section provides sufficient detail for the work to be reproduced.

- The authors have conducted sufficient ablation experiments to demonstrate the effectiveness of the individual modules.

In conclusion, I recommend the publication of this paper in Nature Communications due to its significant contributions to the field, novel framework, and innovative designs.

Minor issues:

In addition to my previous comments, I have a few suggestions for improving the presentation of the paper:

1. Please optimize the overall structure of the paper by placing the most important results and methods in the main body, and moving the less critical information to the supplementary materials. This will help emphasize the key contributions of your work.

Minor issues:

2. In Figure 3, your work is referred to as "MechRetro." Please clarify if this is an alternate name for your model, or if it is a labeling error that should be corrected.

3. I recommend improving the clarity of the images in the paper, as it will enhance the reader's understanding of the presented concepts.

4. There are typographical errors in the paper that should be addressed:

- In Figure B, please correct the typo.

- In Figure D, please correct the typo.

Incorporating these changes will further enhance the quality of the paper. I maintain my recommendation for publication in Nature Communications, given the significance of the contributions and the novel framework and designs presented in the paper.

Responses to Reviewers

Responses to Reviewer #1

Comments:

The manuscript by Wang et al. presents RetroExplainer, a framework for single-step retrosynthesis, which uses a semi-template/reaction-center based two-stage formulation similar as in GraphRetro. Its main model architectures are based on a graph-Transformer, with additional performance engineering techniques such as contrastive learning and adaptive adjustment of task weights to boost top-k accuracies. However, due to the following major concerns, I recommend rejecting this manuscript.

Major issues with contextualization and motivation:

The summary of related works in the introduction is over-simplifying, if not misleading. The authors do not seem to know exactly what some cited works are doing. As a few examples,

- Lines 43-49: "From the perspective of molecule representations", there are fingerprint-based representations in addition to the mentioned sequence-based and graph-based representations, such as used in Segler et al. and RetroSim. The authors cite both but do not seem to be aware that they use fingerprints and are neither sequence nor graph-based.

Author's response:

We would like to apologize for not including fingerprint-based methods in the "*Introduction*" section of our manuscript. In response to the reviewer's suggestion, we have added a paragraph to introduce fingerprint-based methods alongside template-based methods.

Molecular fingerprint-based methods have been classified as graph-based methods because they are essentially calculated based on some graph algorithms. For example, MACCS[1] is a substructure key-based fingerprint method, Daylight[2] fingerprint is based on path-hashing, and ECFP[3] records each node environment from the targeted atom node up to a specified radius, which is similar to

the node aggregation method of current graph neural networks (GNNs). Fingerprint-based methods serve as a procedure for GNNs to obtain graph-level molecular representations, with the difference that fingerprint-based methods are mainly based on topological hashing, whereas GNNs employ a set of learnable parameters.

However, classifying molecular fingerprint-based methods as graph-based methods seems too general due to their crucial impact on the development of current retrosynthesis approaches. Therefore, in the revised manuscript, we have added a passage to introduce fingerprint-based methods, including Segler et al.[4] and RetroSim[5]. The important descriptions of the fingerprint-based methods are listed in the main text as follows:

-Line 67 (from Main text): "*In the early age of data-driven retrosynthesis, researchers primarily focused on developing template-based retrosynthesis approaches that rely on a reaction template to transform products into reactants*[4,6]*. Among these approaches, molecular fingerprints with the multi-layer perceptron are often used to encode molecular products and recommend reasonable templates. For instance, Segler et al.*[4] *utilized extended-connectivity fingerprints (ECFPs)*[3] *with an expansion policy network to guide the template search, whereas Chen et al.*[6] *adopted a strategy similar to a single-step retrosynthesis predictor for their neural-guided multi-step planning. However, the process of constructing reaction templates currently relies on manual encoding or complex subgraph isomorphism, making it difficult to explore potential reaction templates in vast chemical space. To address these issues, template-free and semi-template methods have emerged as promising alternatives, utilizing molecular fingerprints to obtain molecular-level representations. Coley et al.*[5] *used the fingerprinting technique to quantify molecular similarity for ranking candidate precursors to search for appropriate reactants. In addition to molecular fingerprints, existing template-free and semi-template approaches can be generally categorized into two classes: (1) sequence-based approaches*[7-9] *and (2) graph-based approaches*[10-12]*. The three classes of the method mainly differ in the strategies of molecular representations; the molecules are usually represented as linearized strings for sequence-based approaches*[7-9]*, and as molecular graph structures for graph-based approaches*[10-12]*.*"

Comments:

The authors' definitions of sequence-based approaches as encoder-decoder-based translation, and of graph-based approaches as graph changes, are both problematic. First, this does not include the important family of template-based methods at all. Secondly, there are graph-based translation approaches such as GET and Graph2SMILES, both of which the authors cite too.

Author's response:

We apologize for the misinformation caused by the overly narrow definition presented in the text. We have revised the two problematic definitions questioned by the reviewer. We greatly appreciate the valuable feedback provided by the reviewers.

Our initial purpose was to emphasize the methods of molecular representation rather than how they are ultimately predicted. Thus, we have revised the corresponding definitions as follows:

-Line 80: "In addition to molecular fingerprints, existing template-free and semi-template approaches can be generally categorized into two classes: (1) sequence-based approaches[7-9] and (2) graph-based approaches[10-12]. The three classes of the method mainly differ in the strategies of molecular representations; the molecules are usually represented as linearized strings for sequence-based approaches[7-9], and as molecular graph structures for graph-based approaches[10-12]."

-Line 87: "Sequence-based approaches have been used to represent target product molecules using serialized notations, such as SMILES[13]."

-Line 105: "Graph-based approaches are commonly used to represent molecules through graph structures, which are used to predict changes in the target molecule and infer the reactants."

-Line 123: "In addition to the above methods directly modeling graph changes, there are other graph-based approaches that predict graph changes by translating reactants[12,14,15]."

Comments:

- Line 58: "Segler et al." is cited as augmenting Transformers. This is not true. It is one of the most well-known works for template-based retrosynthesis and has nothing to do with Transformers.

Author’s response:

We apologize for incorrectly citing “Tetko et al.” as “Segler et al.,” in which the former used Transformer with SMILES augmentation and the later used symbolic artificial intelligence (AI) and deep neural networks. We have corrected the problem in the revised manuscript. We thank the reviewer for carefully examining our manuscript and pointing out this issue.

Comments:

- Line 66: (Sequence approaches are) “not guaranteed semantically valid”. The authors cite SELFIES but do not seem to know that SELFIES is 100% valid by definition.

Author’s response:

We recognize that SELFIES are semantically 100% valid, but the reviewers' statements were in reference to SMILES. Although the original manuscript did include a description of SELFIES, we had previously removed those sentences for the sake of brevity, which led to a misunderstanding among the reviewers. In order to clarify this misconception, we have re-added the following sentence regarding SELFIES:

-Line 99: “… the SMILES-based molecular representation approach is grammatically strict and not semantically valid, leading to frequent invalid syntaxes.”

Comments:

- Lines 75-76: “Jin et al. propose WLDN … for retrosynthetic graph learning.” This is not true. WLDN was designed for reaction outcome prediction, not retrosynthesis.

Author’s response:

We would like to express our gratitude to the reviewers for their meticulous review. We acknowledge that WLDN is specifically designed for predicting reaction outcomes and was one of the pioneers in proposing the direct description of graph changes for chemical reaction prediction. We apologize for any confusion caused by our previous statements and have modified the corresponding sentence as follows:

-Line 108: "Initially, this idea was used in forward reaction prediction by Jin et al.[16], who proposed using the Weisfeiler–Lehman isomorphism test[17] and graph-learning to predict reaction outcomes."

Comments:

The authors claim three intrinsic problems with existing approaches thereafter, but all three are questionable in my opinion.

- Lines 91-95: The authors claim that both sequence and graph approaches are constrained in feature representation learning, not knowing that graph-Transformers similar to their proposed architecture have already been used in GET, Graph2SMILES and GTA.

Author's response:

While GET, Graph2SMILES, and GTA and our MSMS-GT belong to the Graph Transformer family, our MSMS-GT has distinct characteristics due to several reasons:

1) MSMS-GT encodes topology information by adding positional bias to the attention map in the self-attention mechanism, in which we have designed three important edge blocks: multi-sense edge embedding (encodes σ orbit, π orbit, etc.), multi-hop local embedding (the main idea is that the n-th power of the 0-1 adjacency matrix can represent the n-th order neighbour's relationship), and global embedding (encodes the shortest-path information for each atom pair). Whereas GET takes as input both a sequence representation and a graph representation of the molecule, which seems to be a combination of GNN and Transformer, Graph2SMILES simply adds a shortest path-based global embedding without using the powerful Transformer architecture and multi-hop local embeddings, and GTA takes only sequential representations as input and additionally optimizes a

selective MSE loss related to graph changes, which does not belong to the Graph Transformer family.

2) We provide a view of the Transformer from a GNN perspective, which is instructive for modeling pair-wise relationships (e.g., chemical bonds in a molecule, residue contact map in a protein).

3) Our proposed MSMS-GT predicts graph changes directly, unlike the other three models that output sequential molecular notations.

Comments:

- *Lines 95-97: The authors question the explainability of existing methods, but without discussing template-based methods which are the most interpretable approaches (since templates can be linked to literature precedents).*

Author's response:

We would like to express our gratitude to the reviewer for suggesting the addition of an interpretability comparison between RetroExplainer and template-based models, which we had overlooked. While template-based models may be easier for humans to understand, they still have limitations in comprehending the decision mechanism, explaining counterfactual predictions, and finding quantitative substructural attributions to a more systematic degree. In contrast, our RetroExplainer method has three distinct characteristics that provide interpretable insights, including a transparent decision process, resulting decision energy curve, and substructure quantitative attribution. These features are discussed in detail in the "*RetroExplainer provides interpretable insights*" section of the manuscript. Our decision process is transparent, with each action probability estimated by the corresponding neural module, as explained in the "*Transparent decision process through S_N2 mechanism and deep-learning (DL)-guided molecular assembly*" section and the calculation of decision energy scores in **SI. 3**. Additionally, RetroExplainer generates decision curves that allow users to attribute counterfactual predictions to locate the key process (S-LGM, S-LGC, S-RCP, or HC), as outlined in "*Decision curves uncover the "incorrect" predictions with more insights*." Furthermore, our method offers substructure quantitative

attribution for granular insights by analyzing the percentage of energy contributed by each stage to the final energy score, as discussed in "*Substructure quantitative attribution for granular insights*."

Comments:

Also, the proposed framework is not fundamentally different from existing semi-template methods.

Author's response:

We appreciate the reviewer's comment regarding the difference between RetroExplainer and existing semi-template methods. From the perspective of task modeling, RetroExplainer differs from template-based and semi-template methods. To illustrate this point, we compare RetroExplainer with several existing methods, including direct template-based models, GLN, RetroXpert, G2G, SemiRetro, and GraphRetro:

Given the product graph as $\mathcal{G}_p$, reactant graph set $\{\mathcal{G}_{r;i}\}_{i=1}^{N_r}$ with size of N_r, and template T, the comparisons are as follows:

1) Direct template-based model $p_{\theta_{cls}}$ with learnable parameters θ_{cls} simplifies the retrosynthesis as a multi-classification task on templates:

$$p_{\theta_{cls}}\left(\{\mathcal{G}_{r;i}\}_{i=1}^{N_r}\middle|\mathcal{G}_p\right) = p_{\theta_{cls}}(T|\mathcal{G}_p)f_t\left(\{\mathcal{G}_{r;i}\}_{i=1}^{N_r}\middle|T,\mathcal{G}_p\right), \tag{1}$$

where $f_t(\cdot)$ denotes a deterministic function that transforms the product to reactants given the corresponding template.

2) Integrating more prior knowledge, GLN $p_{\theta_{gln}}$ predicts following a structural pipeline:

$$p_{\theta_{gln}}\left(\{\mathcal{G}_{r;i}\}_{i=1}^{N_r}\middle|\mathcal{G}_p\right) = \underbrace{p_{\theta_{rc}}\left(\{\mathcal{G}_{rc;i}\}_{i=1}^{N_{rc}}\middle|\mathcal{G}_p\right)}_{Reaction\ Center}\underbrace{p_{\theta_t}\left(T\middle|\mathcal{G}_p,\{\mathcal{G}_{rc;i}\}_{i=1}^{N_{rc}}\right)}_{Template\ matching}\underbrace{p_{\theta_r}\left(\{\mathcal{G}_{r;i}\}_{i=1}^{N_r}\middle|T,\mathcal{G}_p\right)}_{Reactant\ matching}, \tag{2}$$

3) RetroXpert and G2G follow a simplified two-step paradigm:

$$p_{\theta_{rx}}\left(\{\mathcal{G}_{r;i}\}_{i=1}^{N_r}\middle|\mathcal{G}_p\right) = \underbrace{p_{\theta_{rc}}\left(\{\mathcal{G}_{rc;i}\}_{i=1}^{N_{rc}}\middle|\mathcal{G}_p\right)}_{Reaction\ Center} \underbrace{f_s\left(\{\mathcal{G}_{s;i}\}_{i=1}^{N_s}|\mathcal{G}_p, \{\mathcal{G}_{rc;i}\}_{i=1}^{N_{rc}}\right)}_{Reaction\ Center\ to\ Synthon} \underbrace{p_{\theta_r}\left(\{\mathcal{G}_{r;i}\}_{i=1}^{N_r}\middle|\{\mathcal{G}_{s;i}\}_{i=1}^{N_s}\right)}_{Synthon\ to\ Reactant}, (3)$$

where $f_s(\cdot)$ denotes a deterministic function that transforms the product according to the predicted reaction center, and $\{\mathcal{G}_{s;i}\}_{i=1}^{N_s}$ represents a set of synthon graphs with size N_s. G2G can be understood as the same as RetroXpert from a general perspective.

4) Directly transforming the synthon to reactants is difficult. Therefore, SemiRetro leverages semi-template for synthon completion, acting more like what GLN does:

$$p_{\theta_{SR}}\left(\{\mathcal{G}_{r;i}\}_{i=1}^{N_r}\middle|\mathcal{G}_p\right) = \underbrace{p_{\theta_{rc}}\left(\{\mathcal{G}_{rc;i}\}_{i=1}^{N_{rc}}\middle|\mathcal{G}_p\right)}_{Reaction\ Center} \underbrace{p_{\theta_{st}}\left(T_{semi}\middle|\mathcal{G}_p, \{\mathcal{G}_{rc;i}\}_{i=1}^{N_{rc}}\right)}_{Semi-Template\ matching} \underbrace{f_{st}\left(\{\mathcal{G}_{r;i}\}_{i=1}^{N_r}\middle|T_{semi}, \mathcal{G}_p\right)}_{Reactant\ matching}, (4)$$

where $f_{st}(\cdot)$ is responsible for the final reactants predicted by the semi-template model and origin product, which is deterministic.

5) GraphRetro introduces the concept of leaving groups, avoiding the direct generation of absent substructure in the product to reduce fitting difficulty:

$$p_{\theta_{gr}}\left(\{\mathcal{G}_{r;i}\}_{i=1}^{N_r}\middle|\mathcal{G}_p\right) = \prod_{i=1}^{N_r}\left(\underbrace{p_{\theta_{rc}}(\mathcal{G}_{rc;i}|\mathcal{G}_p)}_{Reaction\ Center} \underbrace{p_{\theta_{lg}}\left(\mathcal{G}_{lg;i}\middle|\mathcal{G}_p, \mathcal{G}_{rc;i}, \{\mathcal{G}_{lg;k}\}_{k=0}^{i-1}\right)}_{Leaving\ Group} \underbrace{f_r(\mathcal{G}_{r;i}|\mathcal{G}_p, \mathcal{G}_{lg;i})}_{Reactant}\right), (5)$$

where $f_r(\cdot)$ is a hand-coded chemical-rule-based function that generates reactants by predicting the leaving groups and the product.

6) RetroExplainer is trained based on the joint distribution of leaving groups and reaction centers, and the connection function $f_r(\cdot)$ probability is estimated in GraphRetro:

$$p_{\theta_{re}}\left(\{\mathcal{G}_{r;i}\}_{i=1}^{N_r}\middle|\mathcal{G}_{p;m}\right) = \underbrace{p_{\theta_{lgc}}\left(\{\mathcal{G}_{r;j}\}_{j=1}^{N_r}\middle|\mathcal{G}_p, \{\mathcal{G}_{lg;j}\}_{j=1}^{K_l}\right)}_{Leaving\ Group\ Connecting} \underbrace{p_{\theta_{rc\&lg}}\left(\{\mathcal{G}_{rc;i}\}_{i=1}^{K_{rc}}, \{\mathcal{G}_{lg;i}\}_{i=1}^{K_l}\middle|\mathcal{G}_p\right)}_{Reaction\ Center\ \&\ Leaving\ Group}. \quad (6)$$

Therefore, RetroExplainer has its own unique characteristics compared to other deep neural models.

Comments:

- Lines 97-99: The authors question the accessibility of reactants from single-step retrosynthesis, which really questions this very work too. There is a single paragraph describing multi-step extension, yet without addressing the accessibility question.

Author's response:

We appreciate the reviewer's comment regarding the accessibility of reactants from single-step retrosynthesis. We apologize for any confusion caused by our use of the term "accessibility." Our intention was to highlight that the reactants predicted by most of previous one-step retrosynthetic models may not be directly purchasable.

To address this issue, we have developed a multi-step retrosynthetic planning algorithm that integrates with a list of purchasable molecules as the end condition to ensure that the end reactants are "purchasable." We have also revised our manuscript to clarify this point by replacing "accessible" with "purchasable." We hope this clarification will help alleviate any concerns regarding the accessibility of the predicted reactants. The corresponding revision is as follows:

--Line 138: "Most existing approaches focus on the single-step retrosynthesis prediction that enables generating plausible reactants but is perhaps not purchasable and which are usually accompanied by a tedious process of hand-picking predictions. Therefore, the multi-step retrosynthesis prediction with pathway planning from products to accessible reactants is much more meaningful for experimental researchers in practical chemical synthesis."

Comments:

Major issues with results:

- According to the associated codes (https://github.com/wangyu-sd/RetroExplainer. I have to look up the code myself as the link provided in the manuscript gives 404), the featurization is done with the "smile_to_mol_info()" function, but it does not canonicalize the test SMILES with atom mappings removed. This will lead to information leak [1-3] that has spuriously boosted test

accuracy (by up to 15% for RetroXpert). Without proper canonicalization, any results with RetroExplainer on USPTO_50K are questionable.

Author's response:

We appreciate the reviewer's comments regarding potential data leakage in USPTO-50K. We apologize for the inconvenience caused by the outdated code link belonging to MechRetro (our initial release). In the latest version of RetroExplainer, we have addressed this issue by using the "map_id_alignment()" function, which is based on the canonicalization method proposed by RetroXpert. We have also retrained all models using USPTO-50K as the dataset in RetroExplainer.

Compared to MechRetro, RetroExplainer shows a slight drop in top-1 accuracy (from 68.2%/58.0% to 66.8%/57.7%). However, in theory, the MSMS-GT model we used is not affected by data leakage that could cause the majority of atoms in the reaction center to be ranked first. This is because MSMS-GT is a permutation invariant model that does not embed atom order information but only topological information for the molecule. Therefore, the data leakage mentioned by the reviewer is not questionable, and the decrease in accuracy may be due to the negative impact of increasing the model's learnable parameter capacity to improve the top-10 performance. We thank the reviewer for bringing this to our attention.

Comments:

- Line 185: RetroExplainer results are compared with "runner-up Transformer-based method G2GT". However, from Table 1, the runner-up is R-SMILES.

Author's response:

We appreciate the reviewer's comment and apologize for the confusion caused by the mistake in our original manuscript. The comparison we intended to make was between RetroExplainer and R-SMILES, which was the runner-up in our experiments. We have revised the manuscript to reflect this change as follows:

-Line 194: “**Table 1** displays the predictive performance of our RetroExplainer and other existing approaches on the USPTO-50K dataset. The performance was evaluated using the top-*k* exact-match accuracy, with *k* set to 1, 3, 5, and 10. Compared to sequence-based and graph-based approaches, RetroExplainer achieved the optimal level in five out of nine metrics when *k* equals 1, 3, 5, and 1,3 for known and unknown reaction types, respectively. Although our model did not achieve the optimal accuracy when *k* is equal to 10, the accuracy is close to that of the optimal model, with a difference of only 0.2% and 1% compared to LocalRetro. Furthermore, based on the average accuracy of the above two situations (depending on whether the class of reaction is given), our model achieved the highest accuracy, with a difference of 1% and 0.1% compared to the runner-up models, namely LocalRetro for known reaction types and R-SMILES for unknown reaction types, respectively.”

Comments:

- Lines 461-474: The authors extend RetroExplainer for multi-step planning but is missing lots of details. For example, is the value network for Retro retrained? How do you do the “literature search” and how are “similar reactions” defined? The manuscript referes to SI Section 11 but I have no idea which that is as the SI is not numbered.*

Author’s response:

We apologize for uploading an incorrect version of the supporting information that should have been displayed in **SI. 6**. We have modified the problem to address the reviewer's questions. The corresponding details are:

--Line S195: “We provided a convenient interface that integrated the efficient Retro*[6] algorithm to guarantee the end reactants are purchasable. In detail, we developed two versions based on Retro*-0 and Retro* in which we merely replaced the origin cost scores as our energy scores. Considering the difficulty in extracting and accessing the synthesis route dataset based on the USPTO-FULL, we directly adopted the original pretrained neural parameters when using the Retro* version. Additionally, we used about 231 million commercially available building blocks collected from eMolecules[18] to determine whether the end reactants are purchasable.”

Comments:

Major issues with novelty:

In general, RetroExplainer seems to be a mega-amalgamation of know formulation/technique,

Author's response:

We appreciate the reviewer's attention to our proposed RetroExplainer module. While some of the general concepts, such as Graph Transformer, multi-task learning, constative learning, and leaving groups, may be similar to those used in previous works, our ablation studies demonstrate that each technique contributes to the final performance, highlighting their essential and effective roles in the overall framework. We have also made efforts to optimize the implementation of these techniques to ensure that they are tailored to the specific purposes of RetroExplainer. Thank you for your valuable feedback.

Comments:

including

- *Graph-Transformers, as have been done with Graphormer, GET, GTA and Graph2SMILES*

Author's response:

We acknowledge that our initial inspiration for molecular encoding comes from Graphormer, which is designed for a molecular property prediction task. However, MSMS-GT is an upgraded version compared to Graphormer, with differences in the multi-sense embedding and multi-hop local embedding modules, which bring more topological information. As for the differences compared to GET, GTA, and Graph2SMILES, please refer to our previous response to the comment above: "*- Lines 91-95: The authors claim that both sequence and graph approaches are*

constrained in feature representation learning, not knowing that graph-Transformers similar to their proposed architecture have already been used in GET, Graph2SMILES and GTA.".

Comments:

- *Semi-template-based formulation, almost exactly following GraphRetro, except some additional model is trained for leaving group connection*

Author's response:

We appreciate the reviewer's comment. The concept of leaving groups in our work is similar to what GraphRetro proposed, except that we have made some differences to avoid some inherent defects in the GraphRetro to be generalized enough for larger datasets (USPTO-FULL and USPTO-MIT):

1) For improvement in the design of leaving groups, we directly record the type message of the attached bond in the leaving group to avoid ambiguity caused by inference only from the valency rule. For instance, leaving groups "-O-," "=O," and "-OH" in our setting is three different leaving groups, which are considered identically as "O" in GraphRetro.

2) For the multi-leaving situation, GraphRetro needs to iteratively run its model more than once, greatly reducing the model performance; while our model is able to make a connection at the same time. For instance, RetroExplainer can directly predict two leaving groups, "-I" and "-Br," and it connects them through the learnable connection module, whereas GraphRetro needs to predict "-I" or "-Br" first and then predict another leaving group.

3) For the leaving group connection, the connection rule in GraphRetro relies heavily on manual coding, which can be hard work for more complicated datasets. By contrast, RetroExplainer provides a more flexible module to automatically learn these connection rules.

4) From the modeling view, RetroExplainer is different from GraphRetro according to the comparison between **Eq. (5)** and **Eq. (6)**.

Overall, the leaving groups in our work are not simply a copy of the concept proposed by GraphRetro, but rather an improved version in comparison.

Comments:

- *Joint-training of reaction center predictions and leaving group matching, as have been done in Retroformer [4]*

Author's response:

We thank the reviewer for their comment on which Retroformer has already introduced the concept of joint learning. However, we need to stress that our contributions in multi-task learning (or joint learning) include not just introducing it to the retrosynthesis task but proposing a novel and effective module that dynamically assigns weight to adaptively balance the different tasks according to their learning difficulty and the numerical scale of their losses. More details can be seen in the ***Dynamic adaptive multi-task learning (DAMT)*** paragraph in the manuscript**.** Additionally, Retroformer simply added the multiple losses without considering the coefficients of different tasks for better optimization.

Comments:

- *Contrastive learning, as have been done in GLN, Dual-TF/TB and Lin et al. [5]*

Author's response:

We appreciate the reviewer's comment on the novelty of contrastive learning. As the reviewer acknowledges, we do not claim that we are the first to introduce contrastive learning for the retrosynthesis task, but we propose a structure-aware contrastive learning (SACL) strategy for the prediction of leaving groups, which is different in both purpose and implementation. The comparisons are as follows:

1) GLN: The maximum log-likelihood estimation (MLE) is used in GLN to optimize the neuralized parameters, which can be viewed as a contrastive learning form where the expectation of positive pairs is maximized while that of negative pairs corresponding to partition function $Z(\cdot)$ is minimized.

2) Dual-TF/TB: The penalization of KL-divergence is used to constrain the consistency between the forward model $p(X)p(y|X)$ and the backward direction $p(y|X)$, which is similar to the core idea of contrastive learning to some extent.

3) Lin et al.: The loss function has conceptual similarities to contrastive learning.

4) Our work: The SACL is developed to guarantee RetroExplainer perceives the structure of leaving groups in the training phase, where the positive samples are those with the same leaving groups and the negative pair is any pair with a different leaving group.

Comments:

The claim of energy-based and mechanism-like decision making is exaggerated. The "energies" seem to be merely probability outputs from the models, and the synthon-based formulation is in little way close to reaction mechanisms, which at least involve some electron flow [6, 7].

Author's response:

We appreciate the reviewer's feedback regarding the use of the terms "energies" and "mechanism-like decision-making" in our paper.

Regarding "energies," we acknowledge that the term is typically associated with precise quantum chemical calculations, which are computationally expensive and time-consuming. Due to these limitations, we use data-driven probability outputs from our models as a substitute for energy scores, like other works[19]. While this approach is not as precise as quantum chemical calculations, it allows us to quickly score a large number of events, making our model more practical for real-world applications. Additionally, our use of energy scores provides our model with the flexibility to score any event within the user-defined scope, improving the interpretability of our predictions.

Regarding "mechanism-like decision making," we agree that our synthon-based formulation is not a complete representation of reaction mechanisms, which involve electron flow. Therefore, we have rephrased it as a “molecular assembly process” to describe our decision-making process.

If the reviewer has any suggestions for alternative terminology, we would be happy to consider them. We appreciate the opportunity to clarify our approach and hope our response addresses the reviewer's concerns.

Comments:

Major issues with presentation and grammar:

The styling needs improvement as it is disrupting at this moment. Please consider having some native English speaker to proofread the manuscript. Just a few examples:

Author’s response:

We apologize for any disruptions caused by language issues in our manuscript. We understand the importance of clear and concise communication in scientific writing and have taken steps to address this concern. Specifically, we have asked a native English speaker to proofread our manuscript and improve its overall style and readability. We hope this will improve the quality of our writing and make it easier for readers to understand our research. We appreciate the reviewer's feedback and will continue to strive for clear and effective communication in our work.

Comments:

- *Line 5: Why do you abbreviate the names of the corresponding authors?*

Author’s response:

Thank you for bringing to our attention the issue with the authorship signatures in our manuscript. We apologize for the mistake of abbreviating the names of the corresponding authors and have revised them to their full names. We appreciate the reviewer's attention to detail and strive to ensure the accuracy of all aspects of our work.

Comments:

- Lines 32-33: “RetroExplainer discovers 101 pathways in which 86.9% reactions can be found similar reaction patterns.” Not sure what the second part means.

Author’s response:

We thank the reviewer for pointing out the question. We have modified this sentence as follows:

“RetroExplainer has identified 101 pathways, in which 86.9% of the single reactions correspond to those already reported in the literature.”

Comments:

- In Table 1, different methods are tagged with “T” or “G” or single/double asterisks, without any clear order. Please consider reorganizing, for example into groups of methods.

Author’s response:

We thank the reviewer for their helpful suggestion regarding the organization of comparative approaches in **Table 1**. We recognize the need for a clear and organized presentation of our data and have taken steps to address this concern. Specifically, we have reorganized the comparative approaches into three distinct groups: 1) sequence-based, 2) graph-based, and 3) other-based, such as fingerprints. We believe this new organization will make it easier for readers to understand the similarities and differences between the different methods. We appreciate the reviewer's feedback

and will continue to strive for clarity and organization in our work. The corresponding modified tables are as follows:

Table R1. Performance of our RetroExplainer and the state-of-the-art methods on USPTO-50K benchmarks.

Model		Top-*k* accuracy (%)								
		Reaction class known					Reaction class unknown			
	k =	1	3	5	10		1	3	5	10
**Fingerprint-based**										
RetroSim[5]		52.9	73.8	81.2	88.1		37.3	54.7	63.3	74.1
NeuralSym[20]		55.3	76.0	81.4	85.1		44.4	65.3	72.4	78.9
**Sequence-based**										
SCROP[21]		59.0	74.8	78.1	81.1		43.7	60.0	65.2	68.7
LV-Transformer[22]		-	-	-	-		40.5	65.1	72.8	79.4
AutoSynRoute[23]		-	-	-	-		43.1	64.6	71.8	78.7
TiedTransformer[24]		-	-	-	-		47.1	67.1	73.1	76.3
MolBART[25]		-	-	-	-	-	55.6	-	74.2	80.9
Retroformer[26]		64.0	82.5	86.7	90.2		53.2	71.7	76.6	82.1
RetroPrime[27]		64.8	81.6	85.0	86.9		51.4	70.8	74.0	76.1
R-SMILES[28]		-	-	-	-		56.3	**79.2**	**86.2**	91.0
LocalRetro[29]		63.9	86.8	92.4	**96.0**		53.4	77.5	85.9	**92.4**

**Graph-based**								
GLN[30]	64.2	79.1	85.2	90.0	52.5	69.0	75.6	83.7
G2Gs[11]	61.0	81.3	86.0	88.7	48.9	67.6	72.5	75.5
G2GT[12]	-	-	-	-	54.1	69.9	74.5	77.7
GTA[10]	-	-	-	-	51.1	67.6	73.8	80.1
GraphRetro[31]	63.9	81.5	85.2	88.1	53.7	68.3	72.2	75.5
Graph2SMILES[15]	-	-	-	-	52.9	66.5	70.0	72.9
RetroXpert[32]	62.1	75.8	78.5	80.9	50.4	61.1	62.3	63.4
GET[14]	57.4	71.3	74.8	77.4	44.9	58.8	62.4	65.9
DualTF[19]	65.7	81.9	84.7	85.9	53.6	70.7	74.6	77.0
RetroExplainer (**Ours**)	**66.8**	**88.0**	**92.5**	95.8	**57.7**	**79.2**	84.8	91.4

Comments:

- *Line 222 says the decision process is "six stages" but Line 628 says it has "five distinct actions".*

Author's response:

We appreciate the reviewer's keen attention to detail and for bringing to our attention the inconsistency in our description of the decision-making process. We have carefully reviewed our manuscript and have made the necessary revisions to ensure consistency between the methods and

results sections. Specifically, we have modified the corresponding statement in the methods section to match the description in the results section. We thank the reviewer for their helpful feedback and will continue to strive for accuracy and clarity in our work.

Comments:

- Line 293 refers to Fig 3a-c but those figures are showing totally different information from what's discussed. Plus, Figure 3 refers to some "MechRetro" model which comes from nowhere.

Author's response:

We appreciate the reviewer's attention to detail and for bringing to our attention the mistake in our reference to the figures in our manuscript. We apologize for the confusion caused by referring to the wrong figure numbers and also for the lack of clarity regarding the "MechRetro" model. We have made the necessary revisions and have updated the figure references to **Fig. R1**. We thank the reviewer for their helpful feedback and will continue to strive for accuracy and clarity in our work.

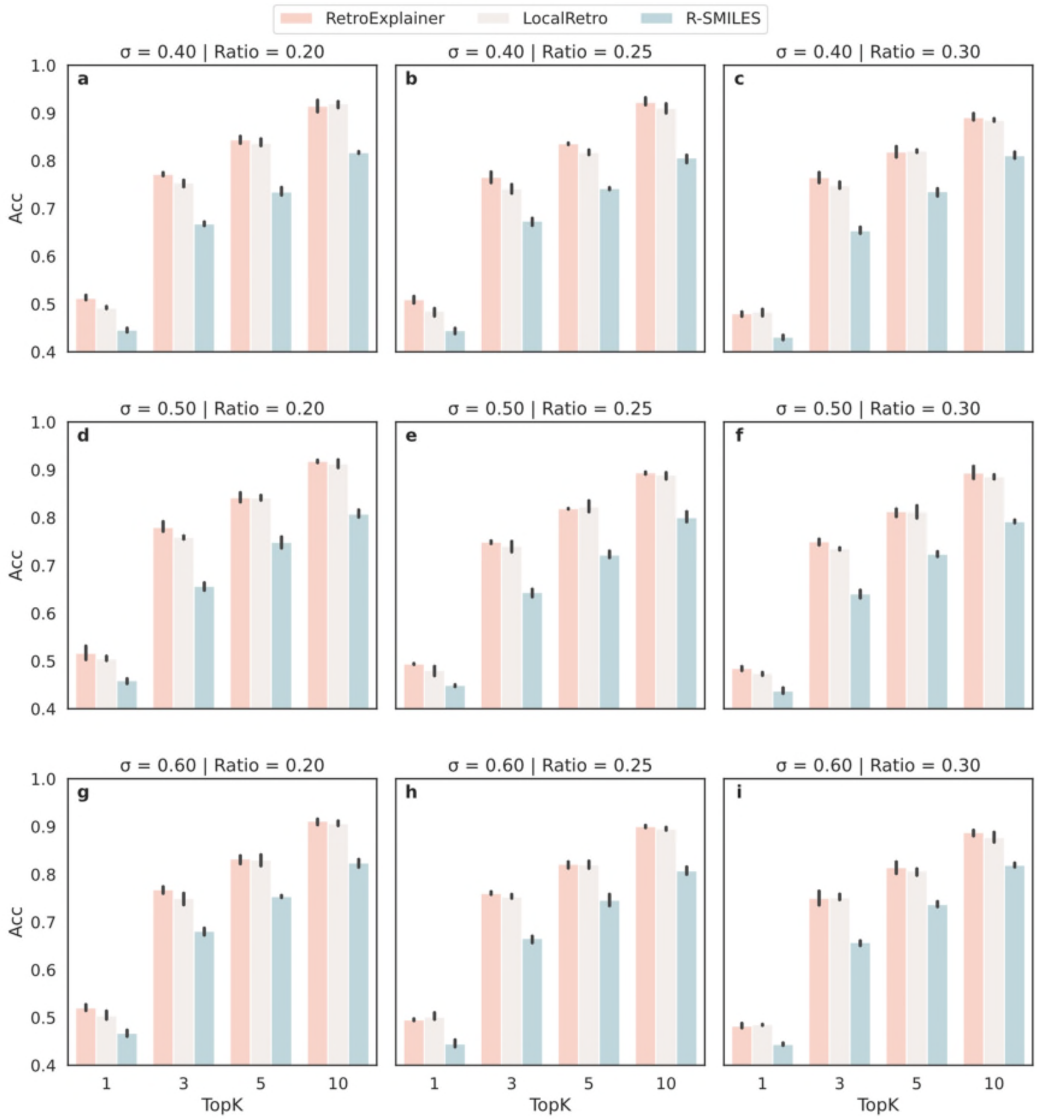

Fig. R1. Performance comparison on the USPTO-50K dataset with Tanimoto similarity splits. **a–i.** Represent the top-k accuracies of our RetroExplainer and the existing methods on the USPTO-50K dataset under different similarity thresholds (i.e., 0.2, 0.25, and 0.3) and different degrees of splitting ratio (i.e., 0.2, 0.25, and 0.3), respectively.

Comments:

- Lines 387-389: the authors try to conclude based on how "discrete the distribution is", but there is no definition of what discreteness is anywhere.

Author’s response:

We appreciate the reviewer’s feedback and for pointing out the lack of definition for "discreteness" in our manuscript. We apologize for the oversight and have made the necessary revisions to address this issue. Specifically, we have included the Davies–Bouldin (DB) Index as a clustering index to justify the discreteness of the distribution. We have also modified the corresponding statements in the manuscript to read as follows: "Notably, according to the Davies–Bouldin (DB) Index, with the reaction type as the label, the more similar the distributions are, the higher the accuracy of the task (from 81.6% to 91.2% on RCP and from 65.4% to 73.2% on LGM). The revised version can be seen in **Fig. R2.**" We thank the reviewer for their helpful feedback and will continue to strive for accuracy and clarity in our work.

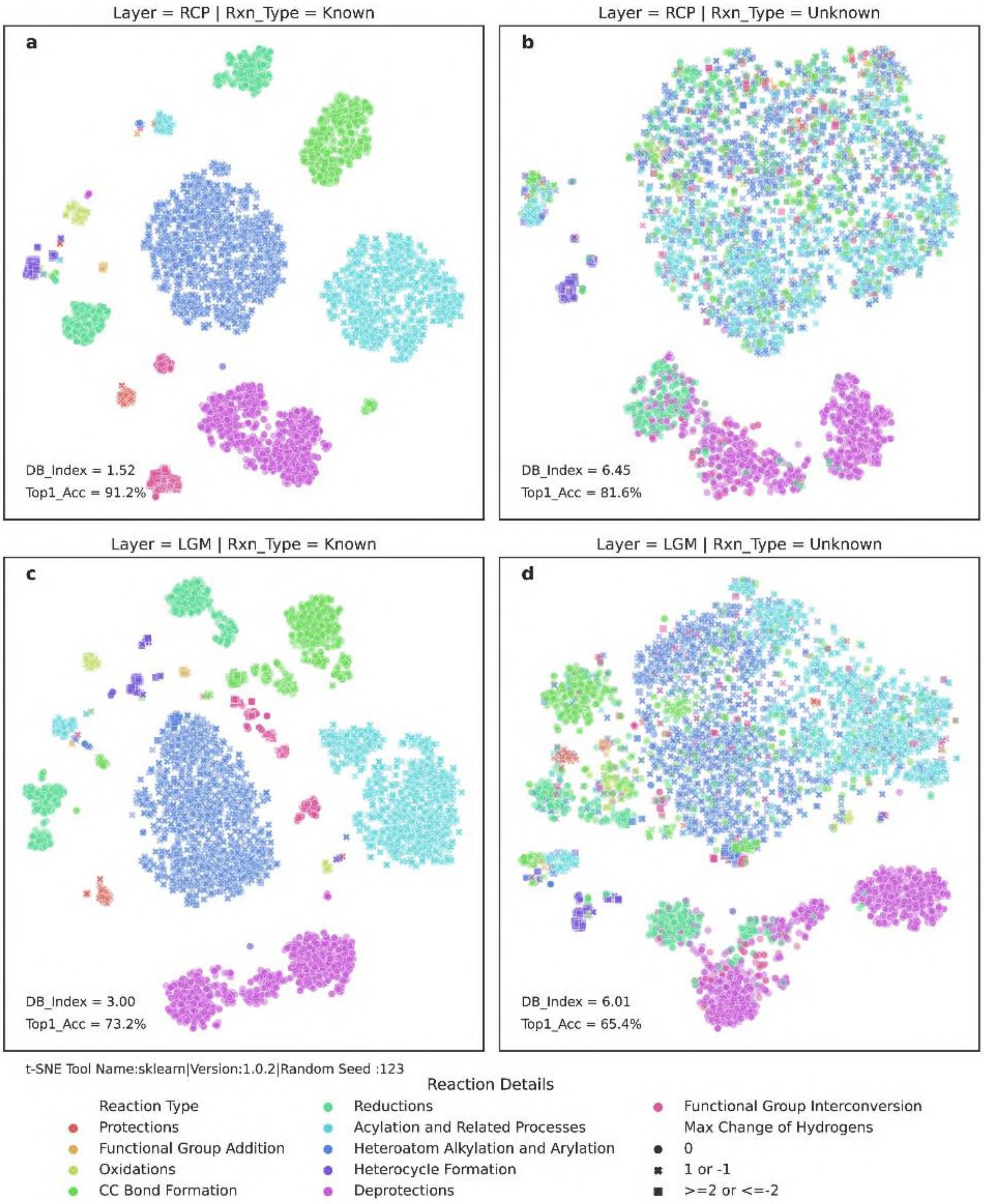

Fig. R2. Distributions of t-SNE from hidden layers of RetroExplainer. a and b. Hidden features are extracted according to (1) whether the reaction type is known and (2) where the hidden features are from (RCP layer or LGM layer), which are then compressed into two dimensions by t-SNE tools. Different colors denote different reaction types, and different styles are determined by the maximum number of hydrogen changes.

Comments:

- *Please number the SI.*

Author's response:

We appreciate the reviewer's suggestion to number the Supplementary Information (SI) in our manuscript. We apologize for the oversight and for any inconvenience this may have caused. We have now numbered the SI in the revised version of our manuscript. Thank you for bringing this to our attention, and we will ensure that all future versions of the manuscript are properly numbered for ease of reference.

Responses to Reviewer #2

Comments:

The manuscript is the improved version of the preprint titled "MechRetro is a chemical-mechanism-driven graph learning framework for interpretable retrosynthesis prediction and pathway planning", published on arXiv in October 2022. The manuscript reports the intriguing achievement of the new state-of-the-art performance on several single-step retrosynthesis benchmarks. To achieve that, the authors developed a novel neural architecture for single-step retrosynthesis titled RetroExplainer inspired by graph neural networks and the Transformers. The authors give a detailed specification of the architecture and its submodules and compared it with the single-step retrosynthesis models from the literature in "reaction unknown" and "reaction known" cases. Furthermore, the authors integrated several explainability features in the model as well as coupled it with the Retro* algorithm to showcase its fitness for multi-step synthesis planning. The approach taken by the authors is promising and will be of considerable significance to the field. However, there are some points that we recommend revising.

Main concerns:

1) The volume of the manuscript is inflated by the number of the raised research questions (single-step retrosynthesis, model agnostic explainability, multi-step setting), and the subsections of the paper come in the suboptimal order, which makes it hard to follow the story.

Author's response:

We appreciate the reviewer's insightful feedback on our manuscript. We agree that the number of research questions raised in the manuscript may have contributed to its inflated volume and suboptimal organization. To address this, we have carefully reviewed the research questions and reduced their number to focus on the most critical and relevant aspects. We have also moved four relatively unrelated sections to the Supplementary Information (SI), including the following:

1. *"Ablation studies on scale information and augmentation strategy of MSMS-GT"* in SI. 1,
2. *"Illustration for multi-scale expressivity on edge representation"* in SI. 2,

3. *“Atom-perturbation-based explainability (APEx) and reaction type tracing analysis”* in SI. 15,
4. *“Reaction type mutation experiment and directed retrosynthesis prediction”* in SI. 17**.**

These changes will help decrease the overall volume of the manuscript while maintaining its core value.

Furthermore, we have restructured the subsections of the manuscript to ensure a more logical and coherent flow, as suggested by the reviewer. Specifically, we have reorganized the sections in the following order:

1. The section "*Performance comparison on USPTO benchmark dataset*" now includes three subsections to evaluate the performance of RetroExplainer.

2. The section "*RetroExplainer provides interpretable insight*" now discusses three aspects of interpretability, including transparent decision-making processes, decision curves from a general view to discover counterfactual predictions, and a newly added section for quantitative attribution in a substructure level which provides more granular insights.

3. We have also added a new section, "*Energy-based process enables effective re-ranking of existing approaches for improved predictions*," to verify the ranking ability of our model, as suggested by the reviewer. "*Extending RetroExplainer to retrosynthesis pathway planning*," corresponding to the multi-step retrosynthesis. "*Influence of reaction types*," discussing the effect of reaction type.

4. A newly added section, "*Limitations*," where we discuss the shortcomings of our model and potential ideas to improve our work for further research in the future.

We believe these changes will make it easier for readers to follow the narrative and understand the research findings more effectively. We thank the reviewer for their helpful feedback and constructive criticism, which has greatly improved the quality and clarity of our manuscript.

Comments:

2) While the score achieved with the model is impressive, the weaknesses of the proposed model are not highlighted. For example, the authors report that they train a model with a fixed number of possible leaving groups. Does this mean that the model will fail on a reaction featuring a rare leaving group which did not appear in the training set?

Author's response:

We appreciate the reviewer's feedback on our manuscript and their concern about the limitations of our proposed model. We agree that it is important to highlight the weaknesses of the model, in addition to its impressive performance.

As the reviewer pointed out, the model we trained has a fixed number of possible leaving groups. While this limitation is mentioned in the manuscript, we acknowledge that we could have provided more details on the possible impact of this limitation on the model's performance. We have now expanded on this limitation in the "Limitations" section of the manuscript, where we discuss the potential challenges that may arise when the model encounters rare leaving groups that were not present in the training set.

We also discuss other limitations of the model in this section, including the flexibility of the decision process and the ability to produce more detailed predictions. We hope this discussion will help readers understand the potential limitations of our model and the areas where further research is needed.

We appreciate the reviewer's constructive criticism, which has helped us to improve the clarity and completeness of our manuscript.

Comments:

Recommendations:

References

1) Please add the comparison against Chemformer (MolBART) and Retroformer on USPTO to the relevant tables.

Author's response:

We appreciate the reviewer's feedback on our manuscript and their suggestion to include comparison against Chemformer (MolBART) and Retroformer on USPTO in the relevant tables. We have now added the comparison results against MolBART and Retroformer to **Table 1** in the manuscript.

However, we would like to point out that we have not found any reported results on larger datasets (such as USPTO-FULL or USPTO-MIT) by MolBART and Retroformer. We have searched for this information extensively but could not find any relevant results. If the reviewer has any additional information on this matter, we would be grateful if they could provide it to us.

We appreciate the reviewer's suggestion, which has helped us to improve the manuscript and provide a more comprehensive comparison of our model with other state-of-the-art models.

Comments:

Unclear points

1) Line 64: The statement seems unclear and questionable. What do you mean by "implicit symbols" and "information loss"? No structural information is lost in SMILES. Could you please elaborate on it more? Could you support the statement "more computational costs" with a reference?

Author's response:

We appreciate the reviewer's feedback on our manuscript and their request for further clarification on our statement about the limitations of SMILES. We apologize for the lack of clarity in our original statement and will address the reviewer's concerns point-by-point:

1. "Implicit symbols": While it is true that SMILES does not lose any structural information of the molecule, it can be challenging for deep-learning models to interpret the linearized representations used in SMILES compared with graph-based models, where the molecular topology is directly contained in the natural molecular graph structure. For example, some symbols in SMILES, such as "(...)" to denote a branch of the molecular backbone, "C1...C1" to denote a circle, and "=" to denote a double bond, are implicit in representing a molecular structure compared with explicit graph structures. This can make it more difficult for deep-learning models to learn and interpret the structural information contained in SMILES.

2. "Information loss": We acknowledge that our original statement about information loss in SMILES was imprecise. What we meant was that more prior information can be embedded through graph-based models, such as molecular hybridization, node degree, Gasteiger charge, and even 3D coordinates, which cannot be encoded directly into a sequence-based model like SMILES. This is because structural symbols are mixed with atomic symbols in a linear representation, making it difficult to embed additional information beyond the basic molecular structure. Therefore, compared to the graph-based models, sequence-based models suffer from information loss problems in prior knowledge embeddings.

3. "Computational costs": We apologize for not being able to provide a reference to support our statement about the computational costs associated with SMILES. We have decided to remove this statement from the manuscript.

We hope that this explanation clarifies our original statement about the limitations of SMILES and provides a more accurate and detailed description of the challenges associated with using SMILES as a molecular representation. We appreciate the reviewer's feedback, which has helped us to improve the manuscript.

Additionally, to clarify the sentence clearer, we modified the original sentence as follows:

"… the linearized molecule representations like SMILES are difficult to explore the direct structural information and atomic properties that are crucial for retrosynthesis analysis."

Comments:

2) Line 91: Here as well, how do the models suffer from the loss of topological information? A SMILES string contains all the information about the topology of a molecule. Also, sequence-based approaches have been achieving a higher score than graph-based models in both product prediction (Molecular Transformer, Chemformer, etc.) and single-step retrosynthesis (R-SMILES), which demonstrates the lack of such suffering. If I am wrong, please correct me. If you agree, please consider changing the wording to make your point in this line more clear.

Author's response:

We appreciate the reviewer's feedback on our manuscript and their request for clarification on our statement about the loss of topological information in sequence-based approaches. We agree with the reviewer that SMILES strings contain all the information about the topology of a molecule. We apologize for any confusion our original statement may have caused.

To address the reviewer's concerns, we have revised the statement to read: "Sequence-based approaches suffer from the loss of prior information of molecules." We believe that this revised statement more accurately reflects the limitations of sequence-based approaches and the challenges associated with encoding additional prior information beyond the basic molecular structure.

We appreciate the reviewer's feedback, which has helped us to improve the clarity and accuracy of our manuscript.

Comments:

3) Line 99: What do you mean by "not accessible"?

Author's response:

We appreciate the reviewer's feedback on our manuscript and their request for clarification on our use of the term "not accessible" in line 99. We apologize for any confusion caused by this wording

and would like to clarify that our intention was to convey that the reactants predicted by most previous one-step retrosynthetic models may not be directly purchasable.

In our approach, we have provided a convenient interface that integrates an efficient multi-step retrosynthetic planning algorithm with a list of purchasable molecules as the end condition to ensure that the end reactants are "purchasable" and, therefore, accessible to experimental chemists. We have replaced our original term "accessible" with "purchasable" to convey our intended meaning more clearly.

Comments:

4) Line 133: Please consider moving the subsection "The framework of the proposed RetroExplainer" and Fig. 1 to the "Methods" section, because it does not report any results and describes the architecture instead.

Author's response:

We sincerely thank the reviewer for the suggestion to move the subsection "The framework of the proposed RetroExplainer." We agree with the reviewer that this section does not report any results and instead describes the architecture of our proposed framework.

To address the reviewer's concerns, we have moved this subsection and the accompanying figure to the "*Methods*" section in the revised manuscript.

We appreciate the opportunity to revise our manuscript and make these improvements based on the reviewer's comments.

Comments:

5) Line 143: "The resulting hidden molecular representations". Do you mean the learned embedding vectors of atoms and bonds or something else?

Author's response:

The mentioned molecular representations are exactly the learned embedding atom and bond embeddings. To make it clearer, we modified the sentence as follows:

-Line 621: "The molecular vectors resulted from former two encoders are blended via the multi-head attention (MHA) mechanism."

Comments:

6) Line 145: What are "deep neural decision units" exactly? Are those MLPs? Please add a clarification.

Author's response:

We apologize for not including more details on "deep neural decision units." In our model, each deep neural decision unit is composed of a separate MSMS-GT layer and a multi-layer perceptron (MLP). The detailed architecture can be referred to in **Fig. 1b** (*right*). Additionally, to clarify, we modified "deep neural decision units" as "specific task heads."

Comments:

7) Line 232: Please provide the pseudocode of the dynamic programming algorithm mentioned here. Otherwise, it is not clear what it does exactly.

Author's response:

We apologize for the unclear statement about the dynamic programming algorithm mentioned by the reviewer. We have attached this pseudocode to **SI. 3**.

Comments:

8) Line 239-241: It is not clear how your learnable module allows you to generate explanations for predictions made by other models. This claim was not in the MechRetro preprint. The energy-based explanation described in the manuscript seems to require a sequence of graph-editing actions of five types. These types, in turn, appear to be integral to RetroExplainer and its submodules like RCP, LGM, LGC, etc. Please support your claim with an experimental demonstration. If you wouldn't be able to do that, please consider removing this claim (also from lines 114-119).

Author's response:

We appreciate the concern raised by the reviewer regarding the unsubstantiated explanations for predictions made by other models. Our intention was to point out that RCP, LGM, and LGC can not only make optimal decisions but also give an evaluation of the likelihood scores in a fairly wide range. To evaluate the enquired solutions provided by other models or human experts, we only need to identify these relevant events in advance, and then according to these events, the energy scores are given by the three task-decision heads to generate the explanations and scoring of the enquired.

To verify the ranking ability of RetroExplainer, we have added a section, "*Energy-based process enables effective re-ranking of existing approaches for improved predictions*," with corresponding pseudocode in **SI. 5**, demonstrating the feasibility of RetroExplainer.

Comments:

9) Line 244: Why is the word "incorrect" taken into brackets? Does this word here mean "not matching the ground truth, but correct in principle"?

Author’s response:

We appreciate the reviewer's feedback on our manuscript and their question regarding the use of brackets around the word "incorrect" in line 244. We agree with the reviewer's interpretation that in this context, the word "incorrect" refers to predictions that are chemically reasonable but do not match the ground truth recorded in the dataset.

To clarify our meaning, we have added a comment in the revised manuscript explaining the use of brackets around the word "incorrect" in this context. The comment now reads: "may make 'incorrect' predictions (chemically reasonable but not matching the ground truth recorded in the dataset)".

Comments:

10) Line 267: Please consider moving the section “Performance in USPTO-50K datasets using Tanimoto similarity splits method” right after the section “Results in USPTO benchmark datasets using the common splitting method” to improve the cohesion of the story.

Author’s response:

We have moved the section “*Performance in USPTO-50K datasets using the Tanimoto similarity splitting method*” right after the section “*Results in USPTO benchmark datasets using the common splitting method*.” We thank the reviewer for their helpful feedback and for helping us to make these improvements to our manuscript.

Comments:

11) Line 292: What does “attention bias” mean?

Author's response:

To clarify, the "attention bias" refers to the attention mechanism used in the Transformer architecture, which assigns weights to different parts of the input sequence based on their relevance to the output. In our manuscript, we describe how the attention mechanism can be biased toward certain parts of the input sequence, which can affect the performance of the model.

To provide further context and clarification, we have added a reference to **Eq. (9.1)** in the manuscript, which describes the attention mechanism and its use in the Transformer architecture. We hope this will help clarify the meaning of "attention bias" and its relevance to our study.

Comments:

12) Line 293: "As illustrated in Fig.3a and 3b, the top-1 accuracy increases and decreases as the max hop increases (from 0 to 5), and it reaches its peak when the max hop equals 4." Figures 3-a and 3-b have no information about the max hop.

Author's response:

We have corrected the wrongly referred description "**Fig. 3a** and **3b**" as "**Fig. 3k** and **3j**." We apologize for any misleading reference this error has caused. The corresponding figure was modified as **Fig. R3**.

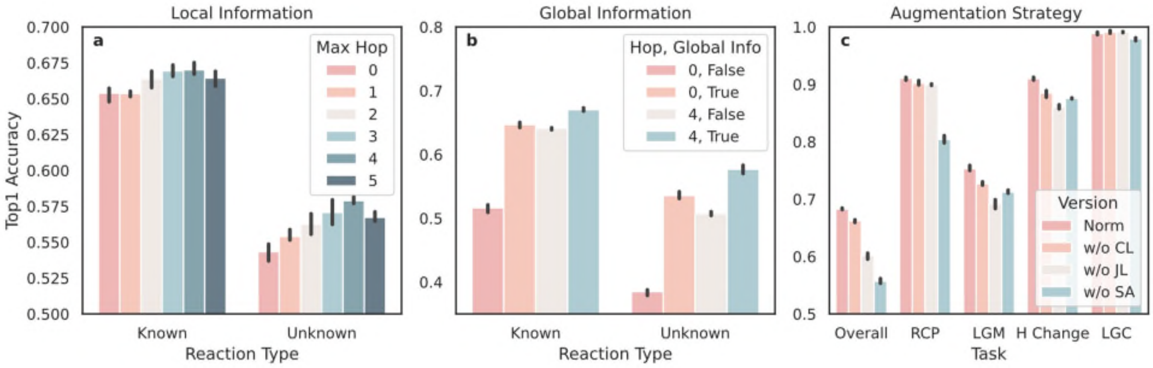

Fig. R3. a and **b. Ablation studies of scale information on known and unknown conditions for reaction types.** We mainly focus on the maximum hop (max hop) of the local item, with the max hop changing to 0 or 4; we remove the global item to verify the validity. With the increasing value of the max hop, the top-1 accuracy increases and decreases. When max hop equals 4, the top-1 accuracy reaches maximum. **c. Ablation studies on different augment strategies for overall prediction and three subtasks.** We consider four conditions to compare: (1) normal version and max hop set to 4, (2) removing contrastive strategy, (3) not adopting adaptive coefficient in multi-task learning, (4) removing all shared layers and training each subtask individually.

Comments:

13) Line 339. Please consider removing the subsection "Multi-scale expressivity on edge representation" from the text. It describes Figure 4, which is hardly insightful and very hard to read. Arbitrary single examples of attention maps may or may not "explain" a model's prediction.

Author's response:

We thank the reviewer for their valuable comment. We expanded this subsection to show that MSMS-GT focuses on the molecular structure at both local and global scales through a case study, which explains why MSMS-GT is better than the previous model in molecular representations. However, this section may not be easily understood by readers and is insufficient to prove the expressivity of MSMS-GT. Thus, we have moved this section to **SI. 2**. and placed a corresponding reference in the subsection about scale information as a simple illustration.

Comments:

14) Line 382: The statement is questionable: one t-SNE plot does not guarantee that "these reaction types can further be divided into more advanced classes". The obtained t-SNE maps may

not look the same when using different random seeds. Also, please specify the software used to obtain the t-SNE plots.

Author's response:

We thank the reviewer for this comment. To support the questionable statements, we introduce the maximum change of the hydrogens categorized into 0, the absolute value (AV) equal to 1, and the AV greater than 1 (i.e., three conditions). The corresponding statements are modified as: "…, which means some reaction types can be further divided into more advanced classes given the specific task. For instance, functional group interconversion (FGI) is divided into two clusters in **Fig. 5a** and three clusters in **Fig. 5c** depending on their maximum change in the number of attached hydrogens from products to reactants." In addition, to reduce the effect of randomness on the clusters, we additionally provide nine t-SNE embedding figures using different random seeds ranging from 124 to 132, where the used tool with its version information is displayed. The resulting figures are renamed as **Fig. R4–Fig. R13**.

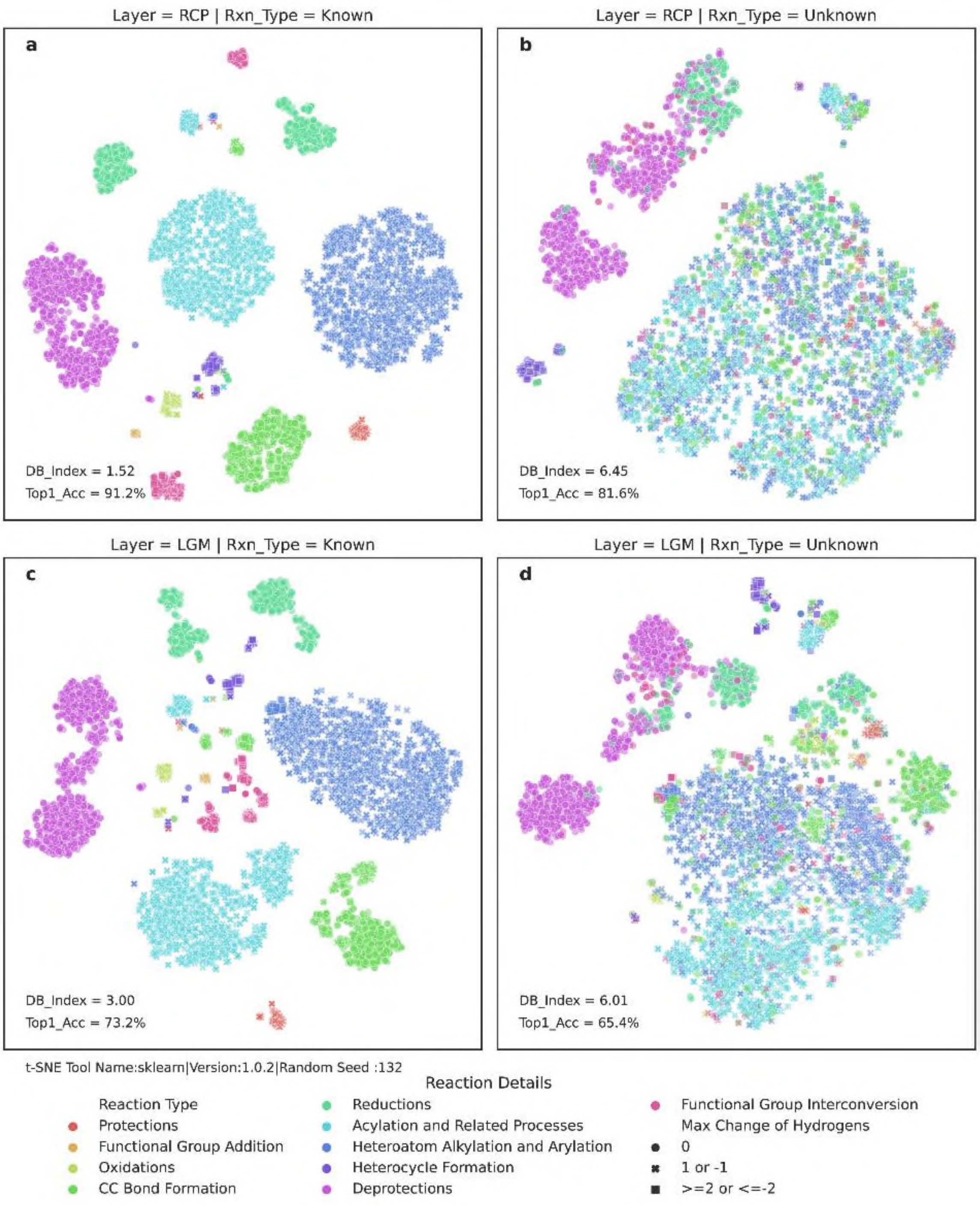

Fig. R4. Distributions of t-SNE from hidden layers of RetroExplainer with random seed of 132. **a-b.** Hidden features are extracted according to 1) whether the reaction type is known, 2) and where are the hidden features from (RCP layer or LGM layer), which are then compressed into two dimensions by t-SNE tools. Different colors denote different reaction types and different styles are determined by the maximum of hydrogen number change.

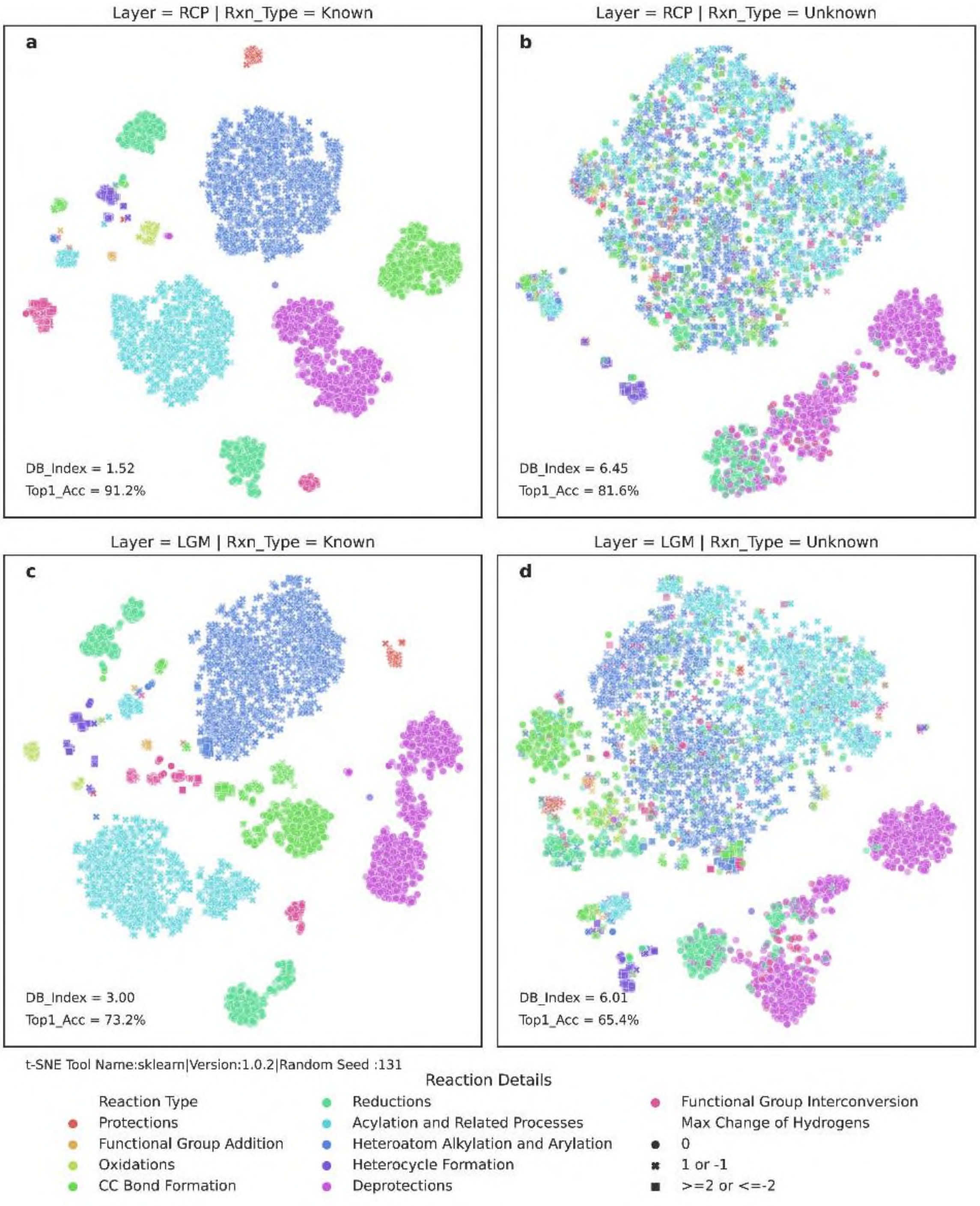

Fig. R5. Distributions of t-SNE from hidden layers of RetroExplainer with random seed of 131. **a-b.** Hidden features are extracted according to 1) whether the reaction type is known, 2) and where are the hidden features from (RCP layer or LGM layer), which are then compressed into two dimensions by t-SNE tools.

Different colors denote different reaction types and different styles are determined by the maximum of hydrogen number change.

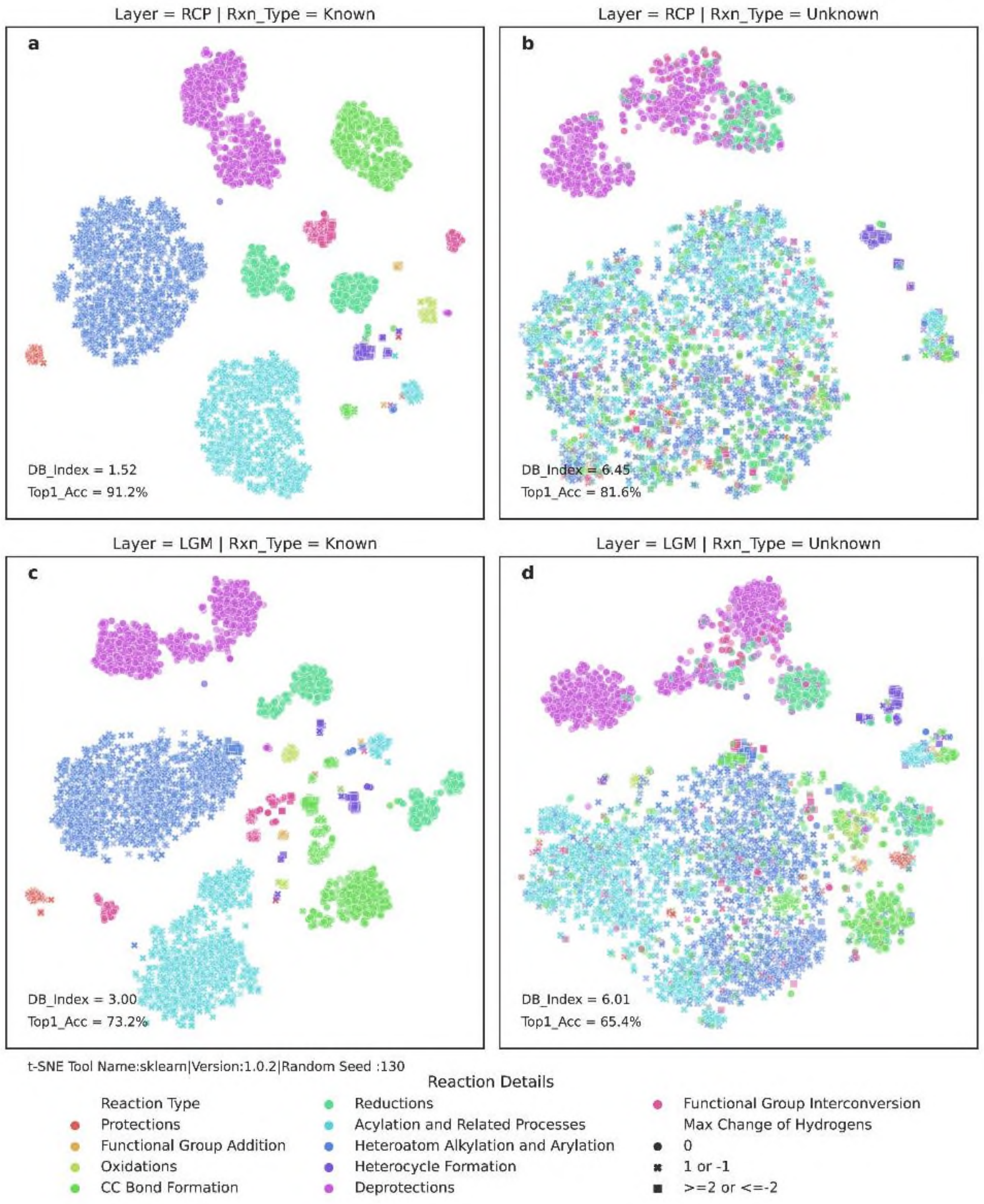

Fig. R6. Distributions of t-SNE from hidden layers of RetroExplainer with random seed of 130. **a-b.** Hidden features are extracted according to 1) whether the reaction type is known, 2) and where are the hidden features from (RCP layer or LGM layer), which are then compressed into two dimensions by t-SNE tools. Different colors denote different reaction types and different styles are determined by the maximum of hydrogen number change.

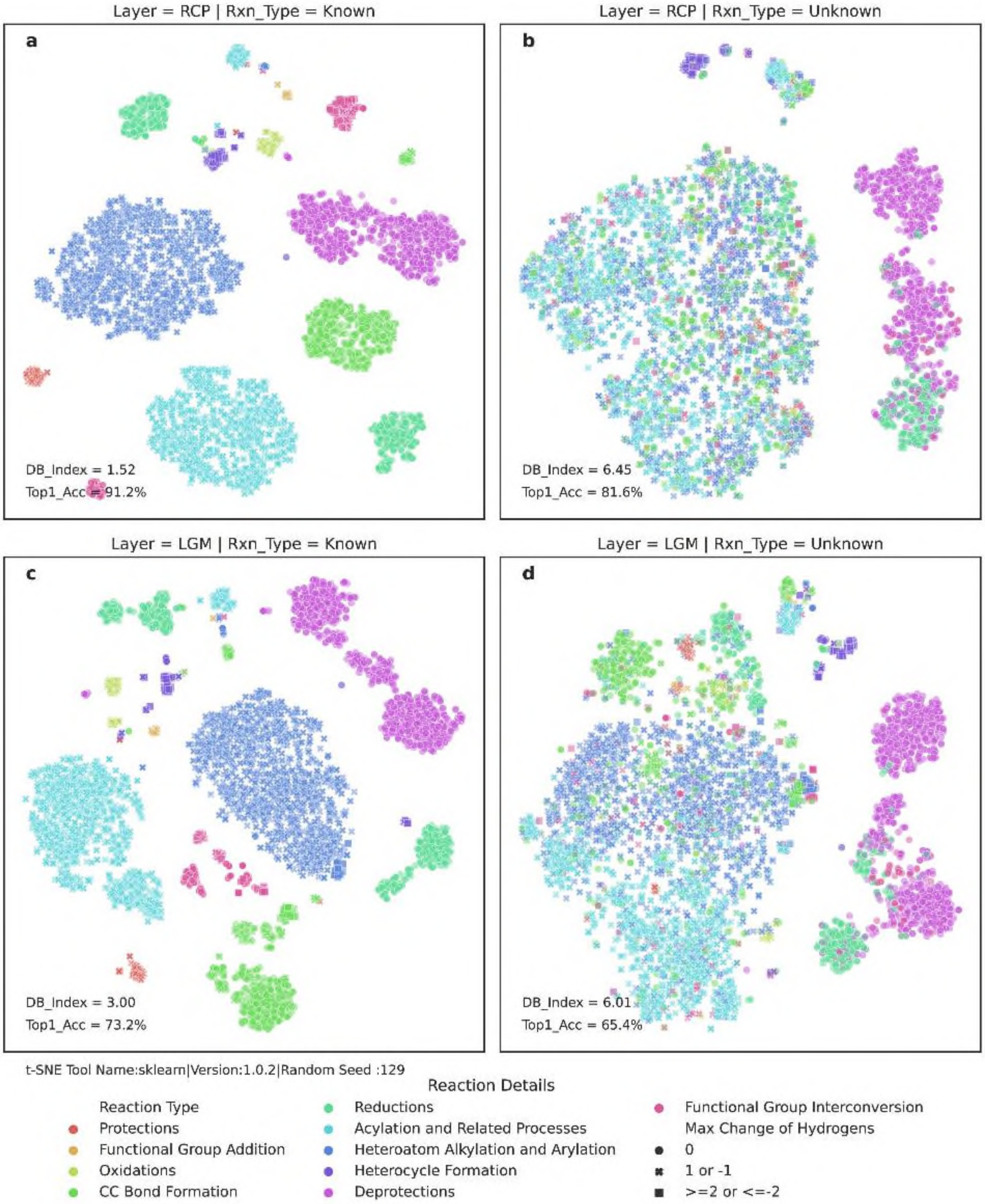

Fig. R7. Distributions of t-SNE from hidden layers of RetroExplainer with random seed of 129. **a-b.** Hidden features are extracted according to 1) whether the reaction type is known, 2) and where are the hidden features from (RCP layer or LGM layer), which are then compressed into two dimensions by t-SNE tools. Different colors denote different reaction types and different styles are determined by the maximum of hydrogen number change.

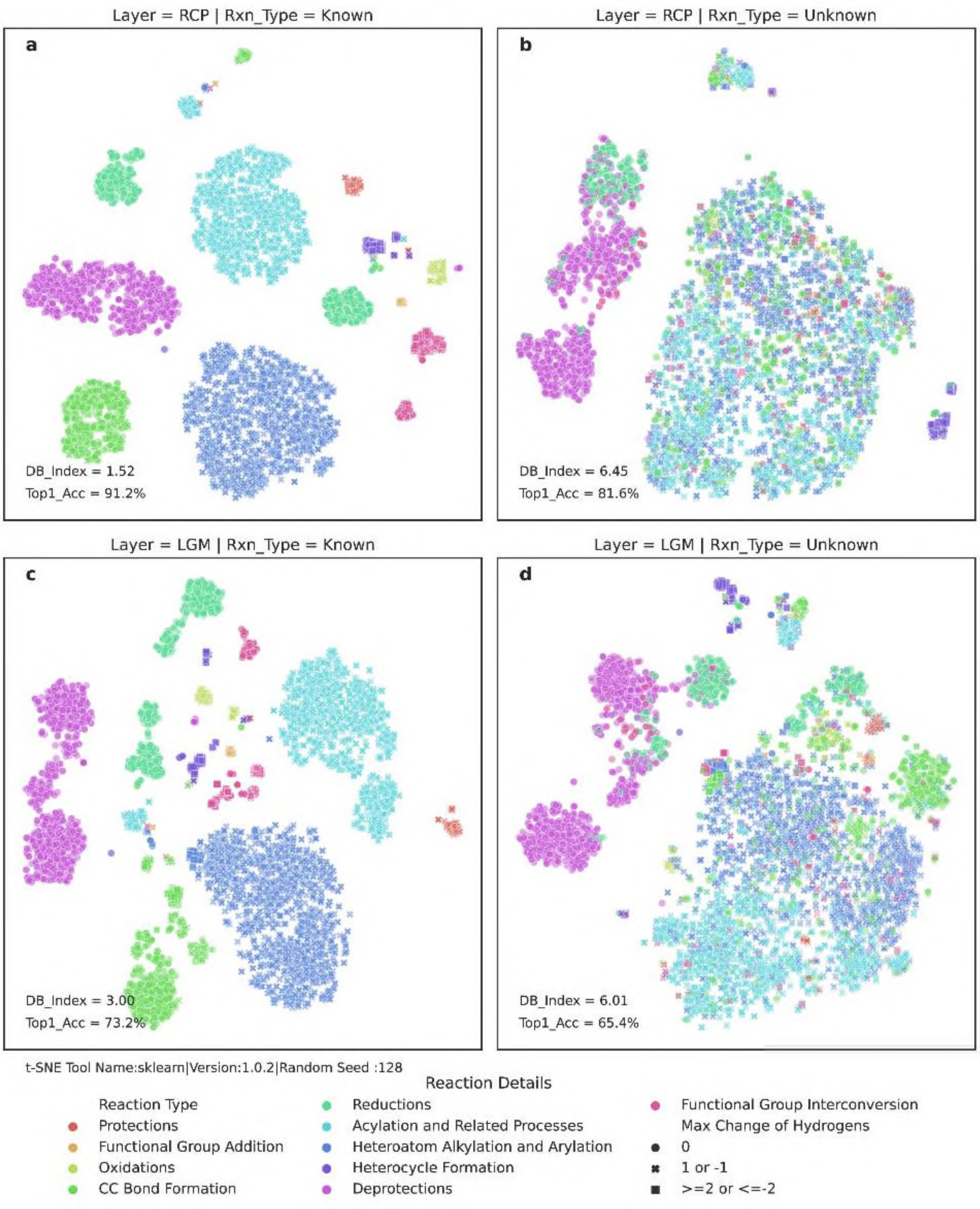

Fig. R8. Distributions of t-SNE from hidden layers of RetroExplainer with random seed of 128. **a-b.** Hidden features are extracted according to 1) whether the reaction type is known, 2) and where are the hidden features from (RCP layer or LGM layer), which are then compressed into two dimensions by t-SNE tools.

Different colors denote different reaction types and different styles are determined by the maximum of hydrogen number change.

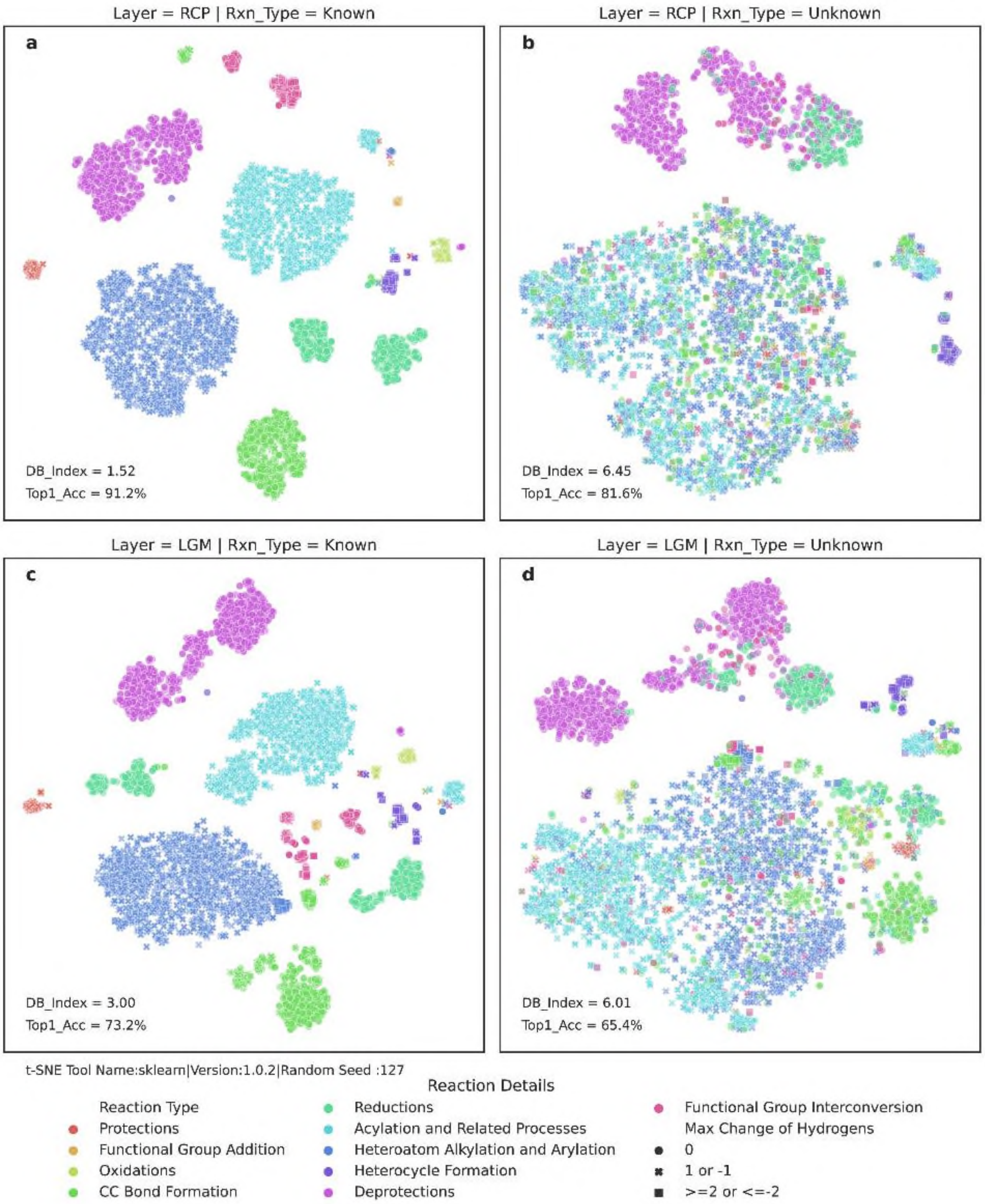

Fig. R9. Distributions of t-SNE from hidden layers of RetroExplainer with random seed of 127. **a-b.** Hidden features are extracted according to 1) whether the reaction type is known, 2) and where are the hidden features from (RCP layer or LGM layer), which are then compressed into two dimensions by t-SNE tools. Different colors denote different reaction types and different styles are determined by the maximum of hydrogen number change.

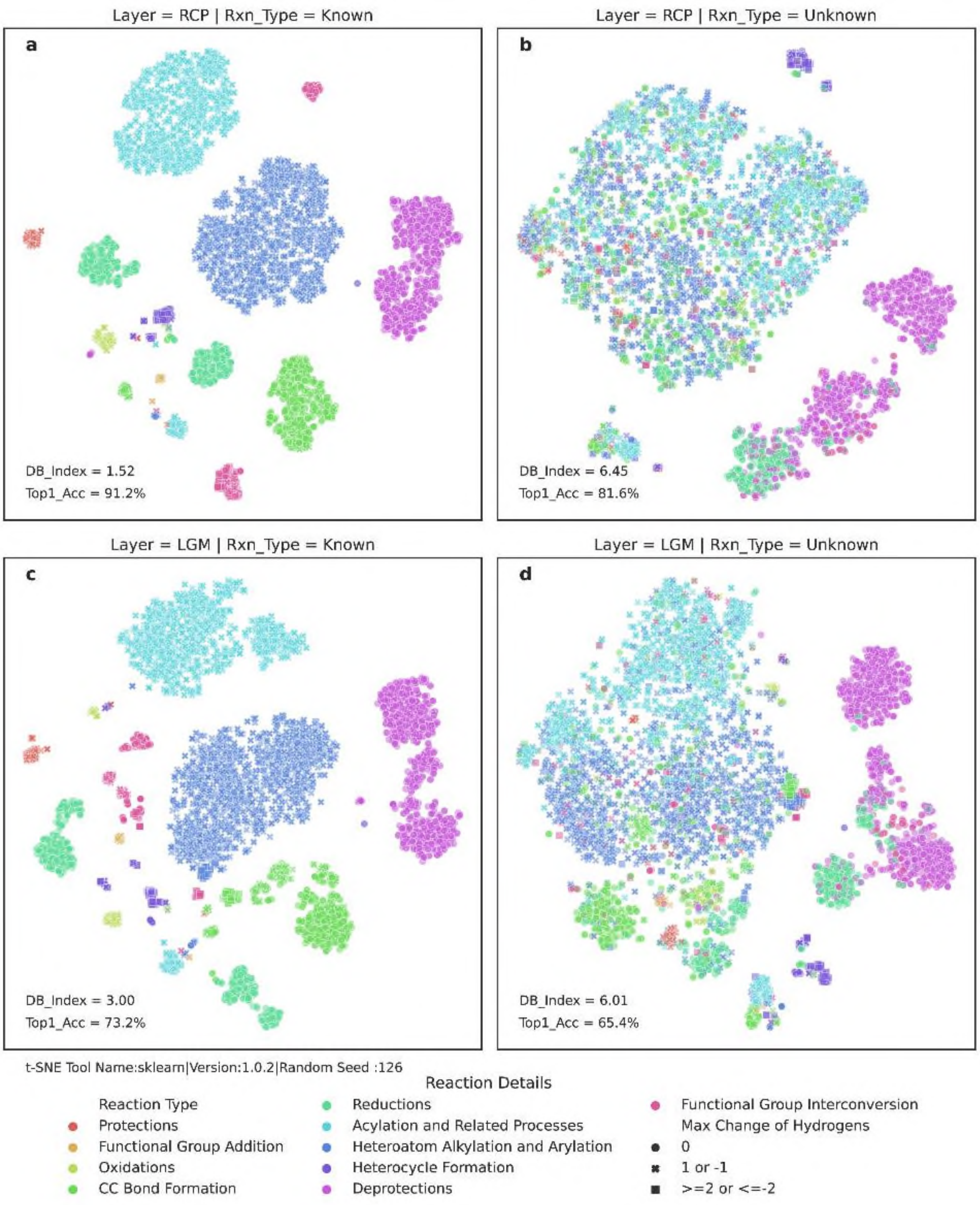

Fig. R10. Distributions of t-SNE from hidden layers of RetroExplainer with random seed of 126. **a-b.** Hidden features are extracted according to 1) whether the reaction type is known, 2) and where are the hidden features from (RCP layer or LGM layer), which are then compressed into two dimensions by t-SNE tools. Different colors denote different reaction types and different styles are determined by the maximum of hydrogen number change.

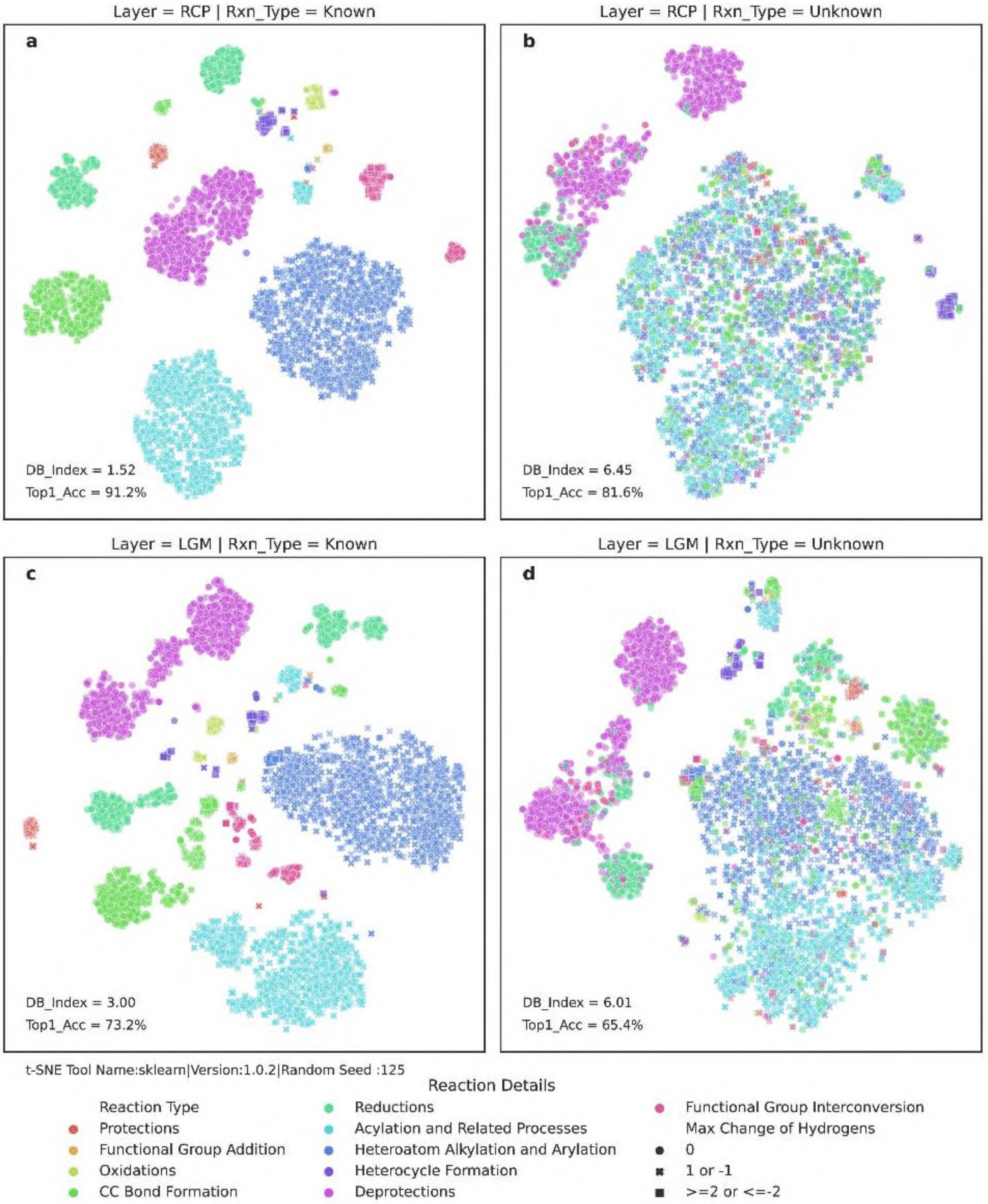

Fig. R11. Distributions of t-SNE from hidden layers of RetroExplainer with random seed of 125. **a-b.** Hidden features are extracted according to 1) whether the reaction type is known, 2) and where are the hidden features from (RCP layer or LGM layer), which are then compressed into two dimensions by t-SNE tools. Different colors denote different reaction types and different styles are determined by the maximum of hydrogen number change.

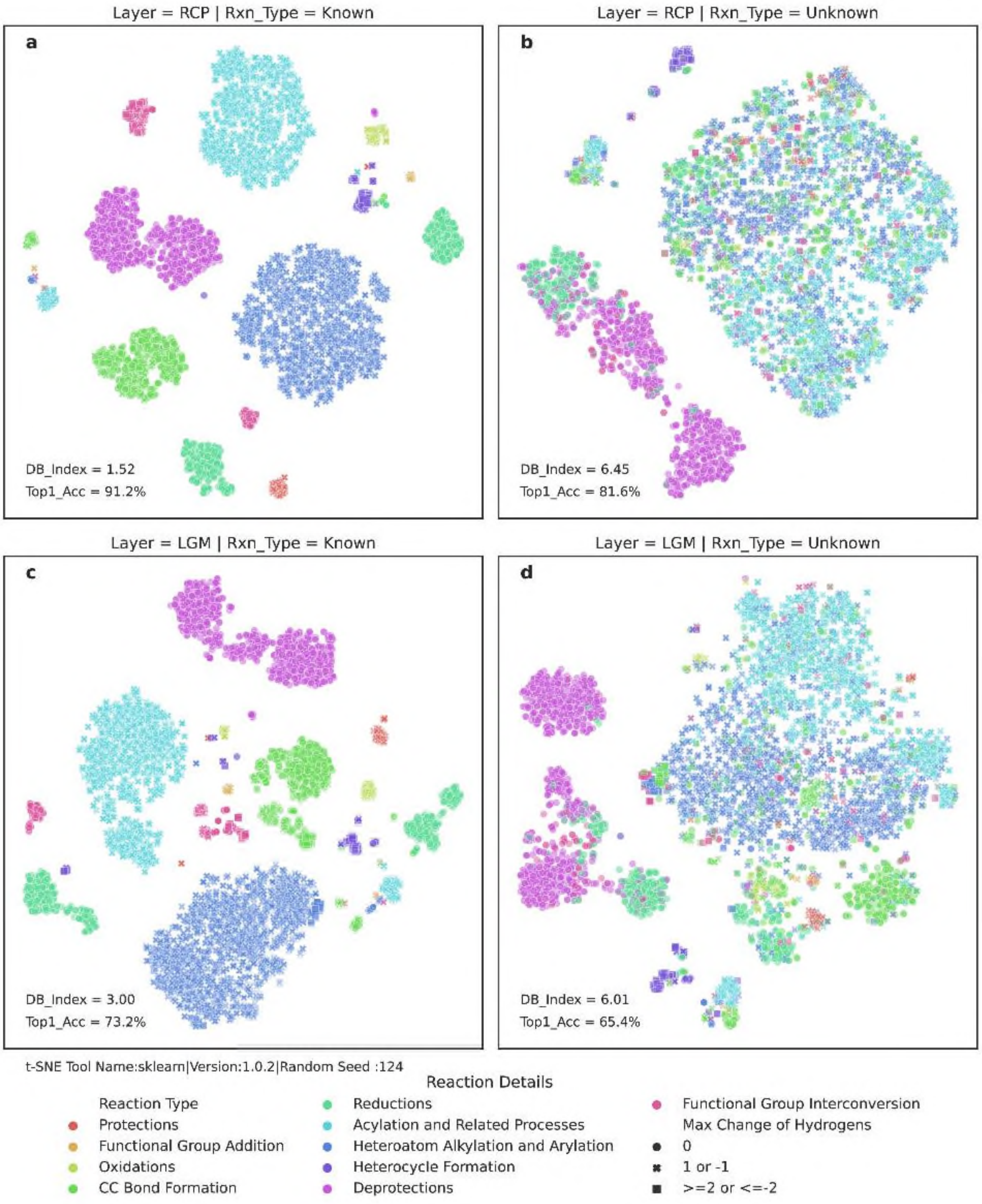

Fig. R12. Distributions of t-SNE from hidden layers of RetroExplainer with random seed of 124. **a-b.** Hidden features are extracted according to 1) whether the reaction type is known, 2) and where are the hidden features from (RCP layer or LGM layer), which are then compressed into two dimensions by t-SNE tools. Different colors denote different reaction types and different styles are determined by the maximum of hydrogen number change.

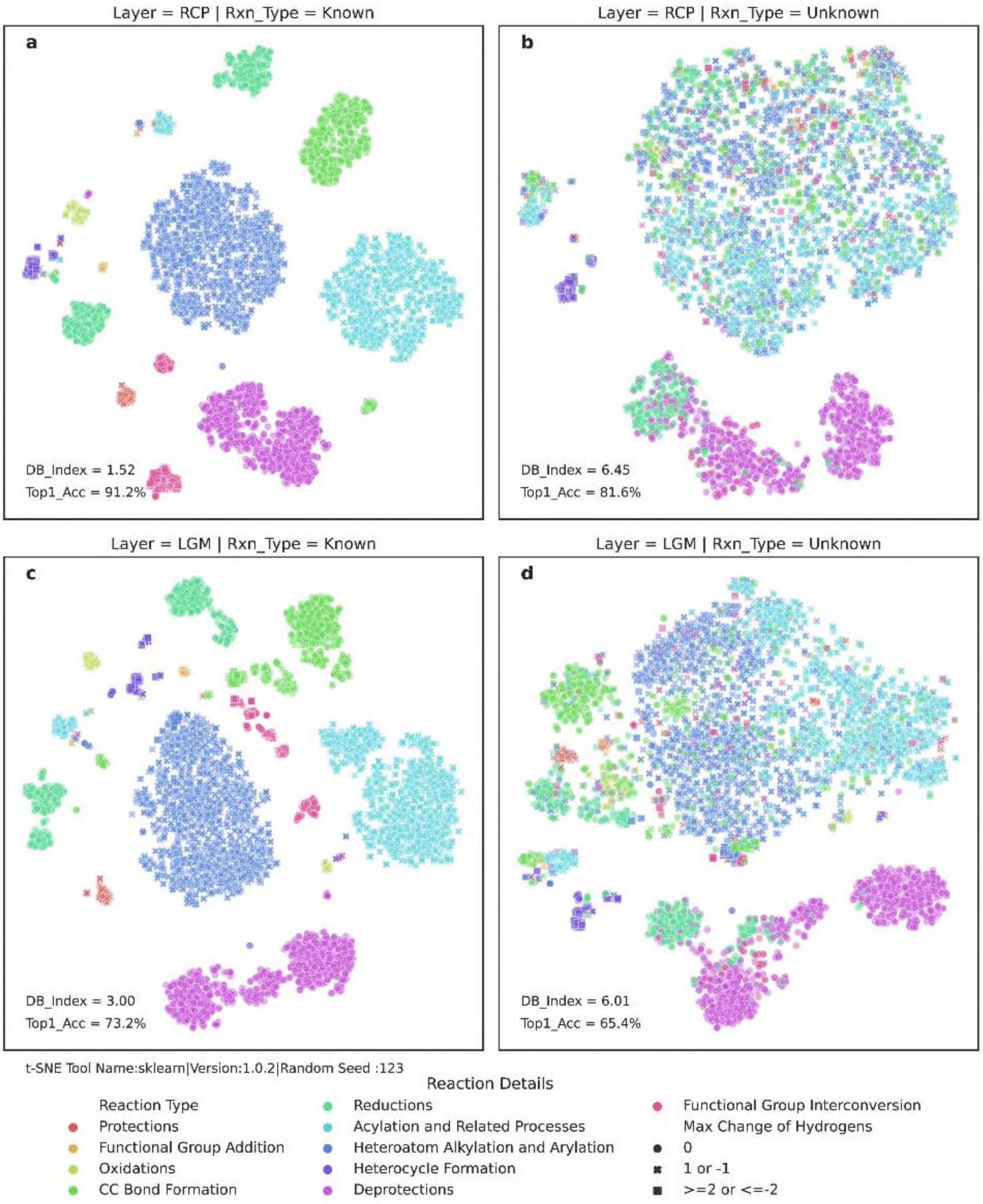

Fig. R13. Distributions of t-SNE from hidden layers of RetroExplainer with random seed of 123. **a-b.** Hidden features are extracted according to 1) whether the reaction type is known, 2) and where are the hidden features from (RCP layer or LGM layer), which are then compressed into two dimensions by t-SNE tools. Different colors denote different reaction types and different styles are determined by the maximum of hydrogen number change.

Comments:

15) Line 400: Reaction type mutation and APEx seem to be really model agnostic. It seems that the sections in lines 400 and 417 are safe to remove from the manuscript because they are worth a separate paper. The paper raises several research questions, which makes it too long in my opinion, and it would not hurt to remove the mentioned points and elaborate more on them in a separate paper, possibly coupling them with different single-step models and formulating the requirements for these models to be compatible with APEx.

Author's response:

Thank you for your valuable feedback regarding the sections on reaction type mutation and APEx in our manuscript. We appreciate your recognition of the importance of these topics.

Upon further consideration, we agree that these sections are somewhat tangential to the main focus of our paper and could be better presented in a separate publication. Therefore, we have moved the discussion of reaction type mutation to **SI. 15**, and the discussion of APEx to **SI. 17**.

We will take your suggestion to heart and plan to develop these sections in a separate, more comprehensive paper that explores the compatibility of different single-step models with APEx and formulates the requirements for these models.

Thank you again for your valuable feedback, which has helped us to improve the clarity and organization of our manuscript.

Comments:

16) Line 417: Where does the APEx algorithm come from? Did the authors invent it? If not, please give an appropriate reference.

Author's response:

Thank you for your question regarding the origin of the APEx algorithm in our manuscript. We would like to clarify that the APEx algorithm was developed independently by ourselves, and we have not found any previously reported algorithm that is identical to APEx.

However, we acknowledge that algorithms sharing a similar idea may have been proposed in another field before, and we apologize for any confusion caused by our use of the word "introduced." If the reviewer has identified any similar algorithms, we would greatly appreciate it if you could share the corresponding references with us so that we can properly acknowledge them in our manuscript.

Comments:

17) Line 485: Did you mean a probability distribution by fθ? If yes, please consider denoting it as pθ to make it clearer.

Author's response:

Yes, and we have modified Eq. (10) in the manuscript as follows:

$$p_\theta\left(\{\mathcal{G}_{r;i}\}_{i=1}^{N_r}\middle|\mathcal{G}_{p;m}\right) = \underbrace{p_{\theta_1}\left(\{\mathcal{G}_{r;j}\}_{j=1}^{N_r}\middle|\mathcal{G}_{p;m},\{\mathcal{G}_{l;j}\}_{j=1}^{K_l}\right)}_{LGC}\underbrace{p_{\theta_2}\left(\{\mathcal{G}_{c;i}\}_{i=1}^{K_c},\{\mathcal{G}_{l;i}\}_{i=1}^{K_l}\middle|\mathcal{G}_{p;m}\right)}_{RCP\&LGM}.$$

Comments:

18) Line 495: Also, synthons are a set of graphs modified by-products according to reaction centers. It seems that some words may be missing. It would be better to rephrase this line and formulate what a synthon is more clearly.

Author's response:

We apologize for the mistake in this sentence, which has been corrected as "Furthermore, synthons are a set of graphs modified by products according to RCs." We appreciate the reviewer's careful inspection.

Comments:

19) Line 534: Could you please provide some examples to support the claim "makes the model unable to distinguish some isomers"?

Author's response:

We have added an instance in **SI. 12** and in **Fig. R14**. **I**f considering the attached hydrogens, even for isomer pairs as simple as *n*-butane and isobutane, we still cannot distinguish them via a deep-learning model without suitable chemical bond encoders. Moreover, in practice, we additionally consider more atomic properties, such as the number of attached hydrogen atoms, hybrid state, and formal charge, to obtain sufficient prior information to avoid this problem. We thank the reviewer for mentioning this point.

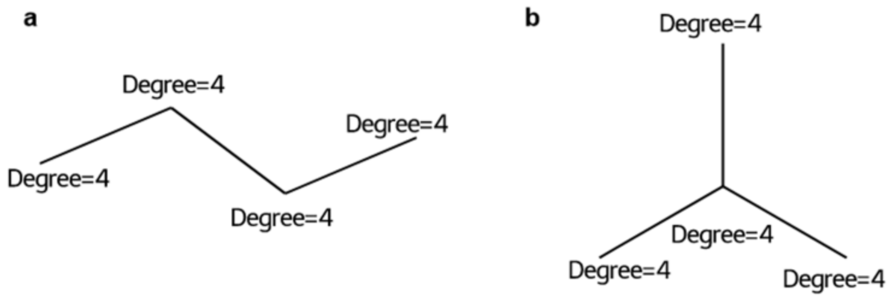

Fig. R14. a and b illustrate an isomer pair that cannot be distinguished by the models without a bond encoder.

Comments:

20) Line 539: Ai is said to belong to two different domains at the same time, which is impossible. Please fix the confusion in the notation.

Author's response:

We thank the reviewer for their careful review of our manuscript and for bringing to our attention the confusion in the notation in line 539. We apologize for any confusion caused by this error.

We have carefully reviewed the relevant section and made the necessary modifications to clarify the notation. Specifically, we have modified the original notation as "$\{A_i \in \{0,1\}^{N\times N}\}_{i=1}^{K_b}$".

Comments:

21) Eq 2: There is no clarification in the text what dk is. While it is obvious for those who are familiar with the Transformer architecture, a broader audience might need clarification.

Author's response:

We have added a descriptive sentence for d_k as "d_k is the hidden dimension of W_K." We thank the reviewer for questioning this point.

Comments:

Typos

1) Fig 1-c: "Dynamatic Adaptive Multi-Task Learning". Did you mean "Dynamic"?

2) Fig 1-d: "Transation state". Did you mean "Transition state"?

3) Fig 1-d: "Macth". Did you mean "Match"?

4) Fig 2-c: "Energy decresed in RCP". Did you mean "decreased"?

5) Line 343: "hot maps". Did you mean "heat maps"?

6) Line 481: A dot before a comma.

7) Line 497: "by attaching leaving group". Did you mean "by attaching a leaving group"?

8) Line 577: "The expressive of RetroExplainer". Did you mean "The expressive power of RetroExplainer"?

9) Line 673: "Decent rate". Did you mean "Descent rate"?

Author's response:

We appreciate the reviewer's careful inspection. All the typographical errors except for 7) have been corrected according to the reviewer's suggestion. Regarding typographical error 7), the original statement is modified as "by attaching leaving groups" because multiple leaving groups exist for many complex reactions.

Comments:

Figures

1) Fig 1-d: What is the difference between E4 and Es? They look identical in the figure.

Author's response:

The difference between E_4 and E_5 is determined by whether the number of attached hydrogen atoms is changed for each atom after the hydrogen changing (HC) stage. In the case of **Fig. 1**, the energy E_4 is equal to E_5 as there is no change during the HC stage. However, in many other reactions, like esterification, the two energies differ.

Comments:

2) Fig 2-c: It would be beneficial for the readability to increase the contrast in the colors of decision curves, especially those that are close to each other.

Author's response:

Thank you for your helpful suggestion regarding the readability of the original **Fig. 2c** (now **Fig. 2b** or **Fig. R15**) in our manuscript. We have carefully reviewed the figure and made the necessary modifications to improve the discriminability of the decision curves.

In addition to adjusting the contrast of the curves, we have also reassigned the thickness value to each prediction based on its final energy score. Thicker curves now represent lower energy scores, which we believe will help to further differentiate the curves and improve their readability.

Thank you again for your valuable feedback, which has helped us to improve the presentation of our results.

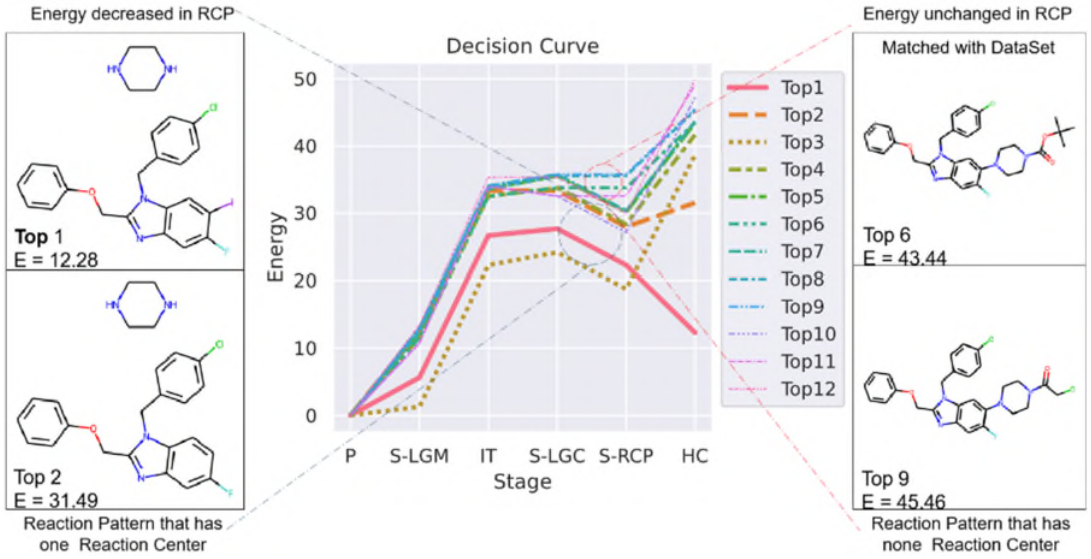

Fig. R15. The decision curve of top-12 predictions by RetroExplainer. The same reaction patterns have the same energy change.

Comments:

3) Fig 3: Features the name "MechRetro" which is never mentioned in the manuscript.

Author's response:

We thank the reviewer for their careful review of our manuscript and for bringing to our attention the inconsistency in the naming of the original **Fig. 3**. We apologize for any confusion caused by this error.

We have reviewed the relevant section and have made the necessary modifications to ensure consistency in the naming of the method. Specifically, we have updated the name "MechRetro" in **Fig. 2** (or **Fig. R16**) to "RetroExplainer," which is the correct name for the method used in our study.

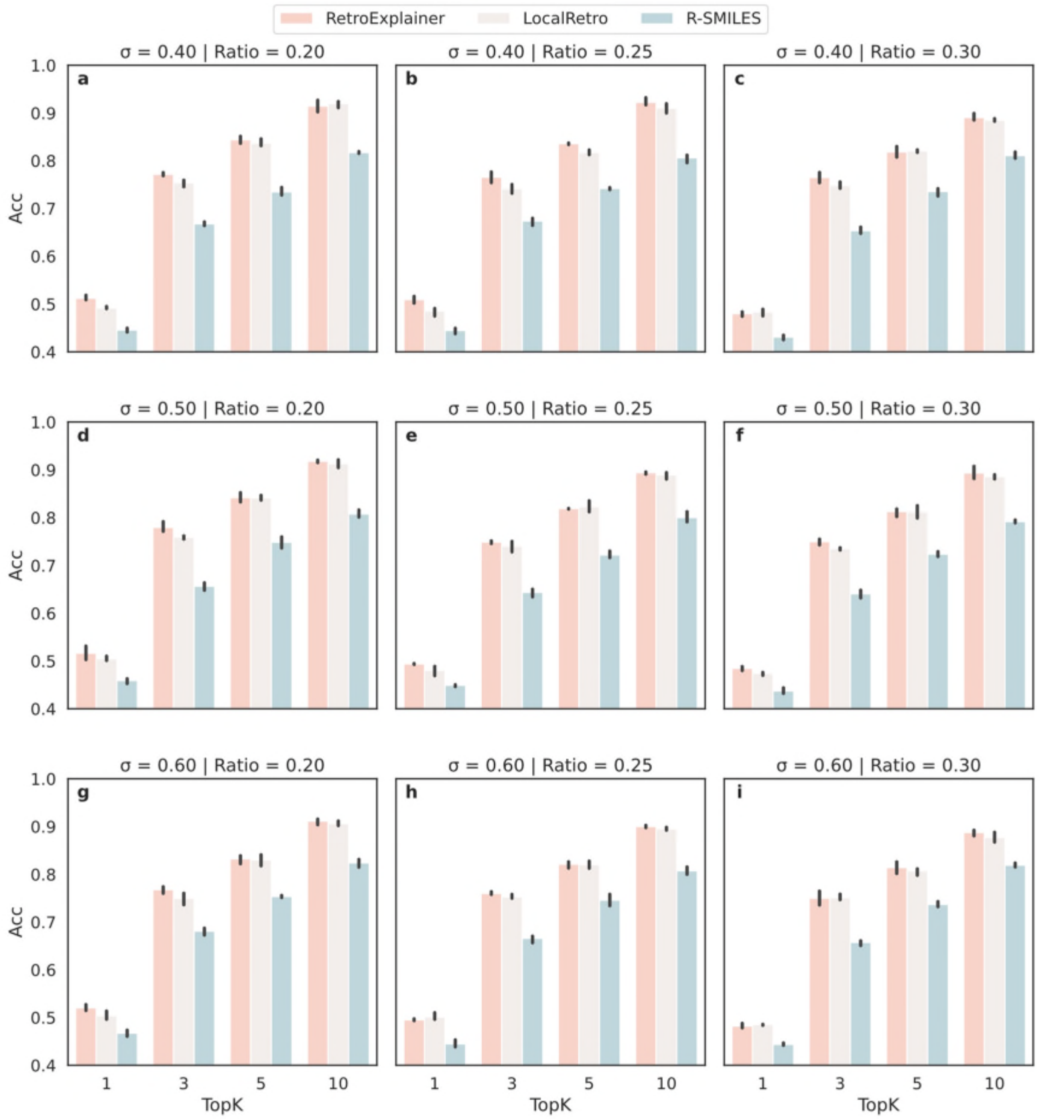

Fig. R16. Performance comparison on the USPTO-50K dataset with Tanimoto similarity splitting. **a–i** represent the top-*k* accuracies of our RetroExplainer and the existing methods on the USPTO-50K dataset under different similarity thresholds (i.e., 0.2, 0.25, and 0.3) and different degrees of splitting ratio (i.e., 0.2, 0.25, and 0.3), respectively.

Comments:

4) Fig 3: Hard to read, seems too cluttered. Please consider splitting it into several separate figures. For example, a-i, j-k, l could be three separate figures. The latter might be better in the supplement. This will also solve the problem with the caption, which otherwise seems haphazard.

Author's response:

We thank the reviewer for their helpful suggestion regarding the presentation of the original **Fig. 3** in the manuscript. We agree that the figure could be difficult to read and understand in its current form, and we appreciate your suggestion to split it into separate figures.

Guided by your recommendation, we have moved subplots **j** and **k** from the original figure to **Fig. S1** in the Supplementary Information (SI). This change has allowed us to better organize the information presented in the figure and has improved its overall readability.

Once again, we appreciate the reviewer's insightful feedback, which has helped us to improve the presentation of our results.

Comments:

5) Fig 4 is very hard to read, and its necessity is questionable. Please consider moving it to the supplement or removing it entirely.

Author's response:

We thank the reviewer for their feedback regarding **Fig. 4** in our manuscript. We appreciate your concern regarding the readability and necessity of this figure.

Guided by your comment, we moved the original **Fig. 4** with its corresponding section to **SI. 2** in the Supplementary Information (SI). This change has allowed us to streamline the main text and present the most important results in a more concise and focused manner.

We appreciate the reviewer's helpful suggestion, which has helped us to improve the presentation of our results.

Comments:

6) Fig 5: The legend is unclear. It is not obvious what classes are represented by numerical labels and why labels 2, 5 and 8 are missing.

Author's response:

Thank you for your feedback regarding the legend in **Fig. 5** of our manuscript. We apologize for any confusion that may have arisen from the unclear labeling of the numerical classes.

Guided by your comment, we have decided to replace the previously submitted t-SNE plot, which provides a clearer and more detailed representation of the data. This change has allowed us to revise the legend to make it more informative and address the issue of missing labels. The modified figure also can be found in **Fig. R17**.

We appreciate the reviewer's insightful feedback, which has helped us to improve the clarity and accuracy of our results.

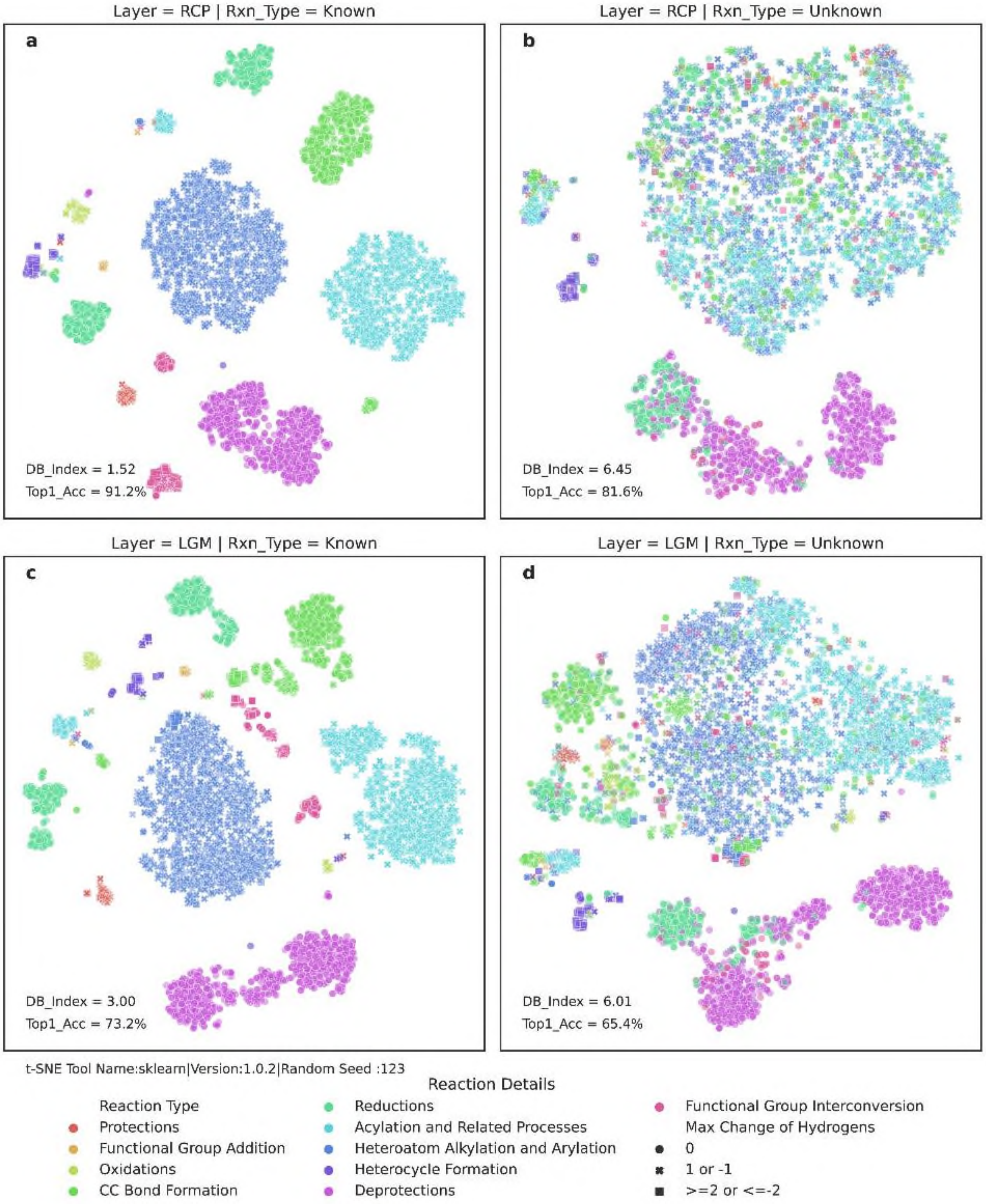

Fig. R17. Distributions of t-SNE from hidden layers of RetroExplainer. **a** and **b**. Hidden features are extracted according to (1) whether the reaction type is known and (2) where the hidden features are from (RCP layer or LGM layer), which are then compressed into two dimensions by t-SNE tools. Different colors denote different reaction types, and different styles are determined by the maximum number of hydrogen changes.

Comments:

7) Fig 8: Please clarify in the caption where the "evidence" (in blue) comes from.

Author's response:

We appreciate the reviewer's valuable comment. We have modified the corresponding sentences as "Although many of the proposed reactions could not be found, we were able to find similar reactions with high yields that matched the proposed reactions. These reactions were found in articles by Ley et al.[33], Nair et al.[34], Roberto et al.[35], and Neudörffer et al.[36], respectively."

Comments:

Supplementary information

1) Features the name "MechRetro", which is never mentioned in the main text.

2) The quality of the figures is too low, which hurts readability.

Author's response:

Thank you for your valuable feedback regarding our Supplementary Information (SI). We appreciate your comments regarding the naming inconsistency and the quality of the figures.

After reviewing your comments, we have corrected all instances of the incorrect name "MechRetro" to the correct name "RetroExplainer" throughout the SI. We apologize for any confusion this may have caused.

Additionally, we have made efforts to improve the resolution and quality of all the figures in the SI to enhance readability and clarity for the readers.

We appreciate the reviewer's helpful feedback.

Responses to Reviewer #3

Comments:

Overall, this is an excellent paper with noteworthy results. The authors present their RetroExplainer model, which provides transparent and interpretable retrosynthesis predictions. They claim it can offer post-hoc explanations and re-rank predictions made by other models, regardless of the model used. Furthermore, they introduce three new highly effective deep learning modules: MSMS-GT for molecular representation learning, DAMT for balanced multi-task learning, and SACL for fast graph-level contrastive learning. The effectiveness of their approach is demonstrated through experiments on several benchmark datasets.

The paper also features a thorough experimental analysis. The work is significant, as it proposes a novel, comprehensive, and effective technical framework that leverages chemical knowledge in model design. The paper presents many innovative designs.

Regarding the primary purpose of the review:

- The noteworthy results include the RetroExplainer model and the introduction of three new deep learning modules (MSMS-GT, DAMT, and SACL).

- The work will be of significance to the field and related fields, as it presents a novel framework and innovative designs that effectively utilize chemical knowledge.

- The work supports the conclusions and claims, as demonstrated by the experiments conducted on benchmark datasets.

- There do not appear to be any flaws in the data analysis, interpretation, or conclusions.

- The methodology is sound, and the work meets the expected standards in the field.

- The methods section provides sufficient detail for the work to be reproduced.

- The authors have conducted sufficient ablation experiments to demonstrate the effectiveness of the individual modules.

In conclusion, I recommend the publication of this paper in Nature Communications due to its significant contributions to the field, novel framework, and innovative designs.

Minor issues:

In addition to my previous comments, I have a few suggestions for improving the presentation of the paper:

1. Please optimize the overall structure of the paper by placing the most important results and methods in the main body, and moving the less critical information to the supplementary materials. This will help emphasize the key contributions of your work.

Author's response:

We appreciate the reviewer's insightful feedback on our manuscript. We agree that the number of research questions raised in the manuscript may have contributed to its inflated volume and suboptimal organization. To address this, we have carefully reviewed the research questions and reduced their number to focus on the most critical and relevant aspects. We have also moved four relatively unrelated sections to the Supplementary Information (SI), including:

1. ***Ablation studies on scale information and augmentation strategy of MSMS-GT*** in **SI. 1**.
2. ***Illustration for multi-scale expressivity on edge representation*** in **SI. 2**.
3. ***Atom-perturbation-based explainability (APEx) and reaction type tracing analysis*** in **SI. 15**.
4. ***Reaction type mutation experiment and directed retrosynthesis prediction*** in **SI. 17**.

These changes will help decrease the overall volume of the manuscript while maintaining its core value.

Furthermore, we have restructured the subsections of the manuscript to ensure a more logical and coherent flow, as suggested by the reviewer. Specifically, we have reorganized the sections in the following order:

1. The section "***Performance comparison on USPTO benchmark datasets***" now includes three subsections to evaluate the performance of RetroExplainer.

2. The section "***RetroExplainer provides interpretable insights***" now discusses three aspects of interpretability, including transparent decision-making processes, decision curves from a general view to discover counterfactual predictions, and a newly added section for quantitative attribution in a substructure level, which provides more granular insights.

3. We have added a new section titled "***Energy-based process enables effective re-ranking of existing approaches for improved predictions***" to verify the ranking ability of our model, as suggested by the reviewer.

4. "***Extending RetroExplainer to retrosynthesis pathway planning***," corresponding to the multi-step retrosynthesis.

5. "***Influence of reaction types***," discussing the effect of reaction type.

6. A newly added section titled "***Limitations***" where we discuss the shortcomings of our model and potential ideas to improve our work for further research in the future.

We believe these changes will make it easier for readers to follow the narrative and understand the research findings more effectively. We thank the reviewer for their helpful feedback and constructive criticism, which has greatly improved the quality and clarity of our manuscript.

Comments:

Minor issues:

2. In Figure 3, your work is referred to as "MechRetro." Please clarify if this is an alternate name for your model, or if it is a labeling error that should be corrected.

Author's response:

We have modified all instances of the error as "RetroExplainer." We thank the reviewer for pointing out this issue. The modified figures are renamed **Fig. R18**:

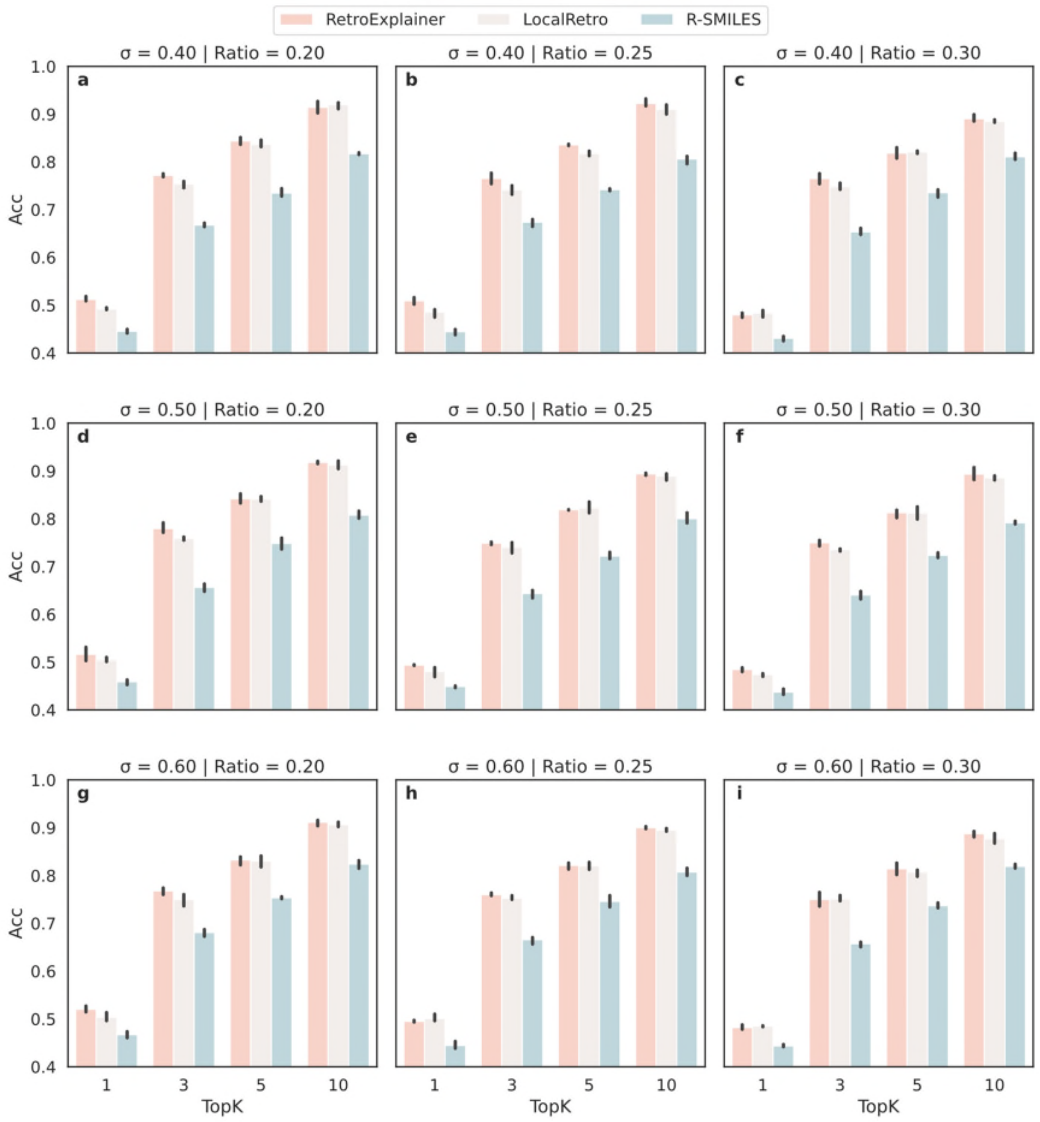

Fig. R18. Performance comparison on the USPTO-50K dataset with Tanimoto similarity splitting. **a–i** Represent the top-*k* accuracies of our RetroExplainer and the existing methods on the USPTO-50K dataset under different similarity thresholds (i.e., 0.2, 0.25, and 0.3) and different degrees of splitting ratio (i.e., 0.2, 0.25, and 0.3), respectively.

Comments:

3. I recommend improving the clarity of the images in the paper, as it will enhance the reader's understanding of the presented concepts.

Author’s response:

We appreciate the reviewer's feedback on the clarity of the images in our manuscript. In response, we have improved the resolutions of all the figures to enhance the reader's understanding of the presented concepts. We are grateful for the reviewer's efforts to improve the quality of our work.

Comments:

4. There are typographical errors in the paper that should be addressed:

- In Figure B, please correct the typo.

- In Figure D, please correct the typo.

Author’s response:

We appreciate the reviewer's careful inspection of our manuscript and the identification of the typographical errors in **Fig. 1b** and **1d**. We have corrected the misspelling of the words "molecule," "last," and "transition" in both figures. Thank you for bringing these errors to our attention. The modified figure is as follows:

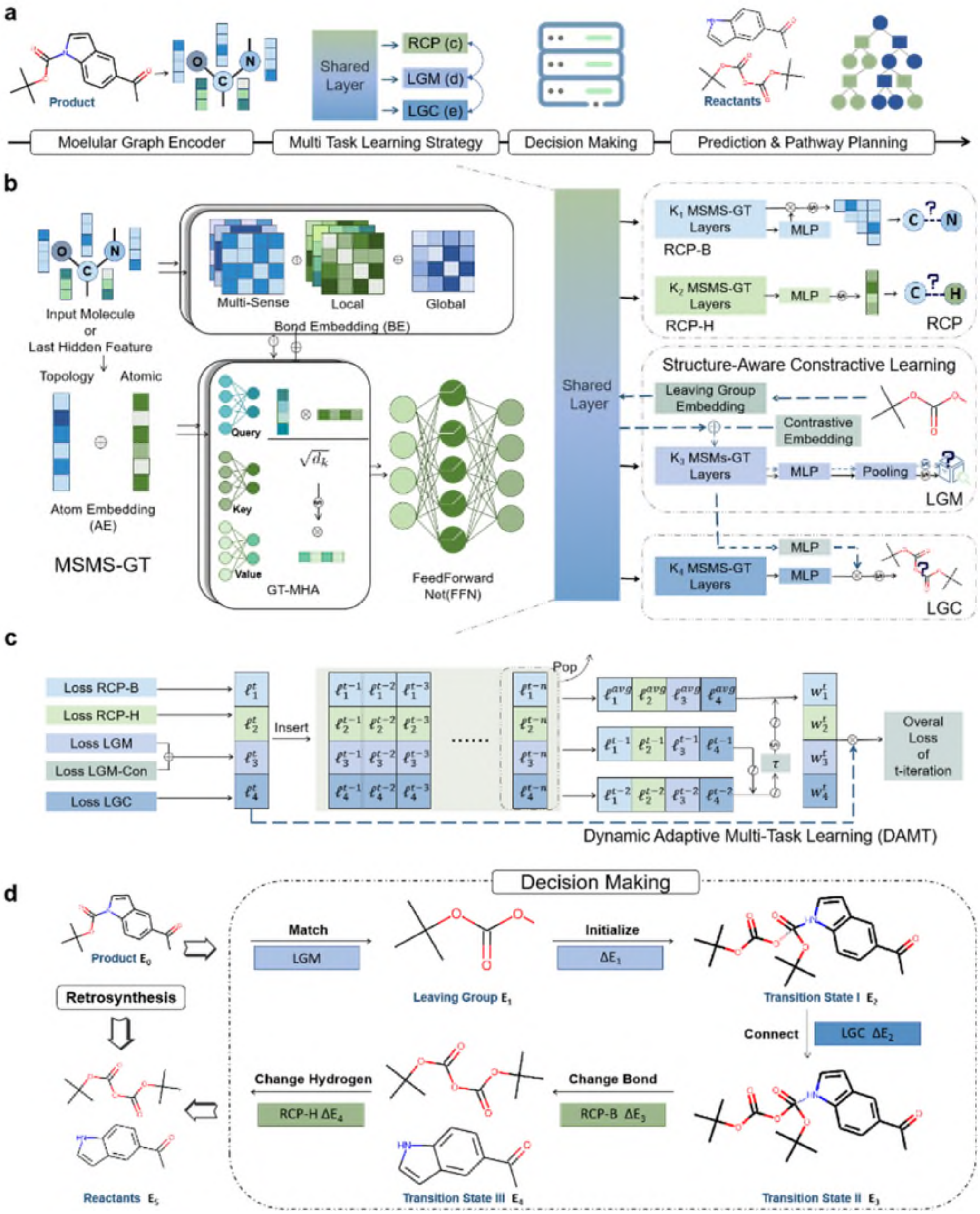

Fig. R17. Overview of RetroExplainer. **a**. The pipeline of RetroExplainer. We formulated the whole process as four distinct phases: (1) molecular graph encoding, (2) multi-task training with contrastive strategy, (3) interpretable decision-making, and (4) multi-step planning. **b**. The architecture of the multi-sense and multi-scale Graph Transformer (MSMS-GT) encoder. We considered multi-sense bond embeddings along with local and global receptive fields and blended them as attention biases during the self-attention execution phase. After obtaining shared features, we factorized the retrosynthesis into five scoring functions. **c**. The dynamic adaptive multi-task learning (DAMT) algorithm. We proposed DAMT to acquire a group of weights according to the descent rates of losses and their value ranges to optimize the five subtasks equally.

d. The chemical-mechanism-like decision process. We designed a transparent decision process which can be attributed to five stages.

Comments:

Incorporating these changes will further enhance the quality of the paper. I maintain my recommendation for publication in Nature Communications, given the significance of the contributions and the novel framework and designs presented in the paper.

Author's response:

We are grateful for the reviewer's positive feedback and recommendation for publication in *Nature Communications*. We have carefully incorporated all the suggested changes to enhance the quality of the paper and are pleased that the significance of our contributions and novel framework and designs have been recognized. Thank you for your time and valuable input throughout the review process.

End of Response

Reference

1 Durant, J. L., Leland, B. A., Henry, D. R. & Nourse, J. G. Reoptimization of MDL Keys for Use in Drug Discovery. *Journal of Chemical Information and Computer Sciences* **42**, 1273-1280, doi:10.1021/ci010132r (2002).

2 Yosef Taitz, D. W., John J Delany. *Daylight chemical information systems: Daylight*, <https://www.daylight.com/> (

3 Morgan, H. L. The Generation of a Unique Machine Description for Chemical Structures-A Technique Developed at Chemical Abstracts Service. *Journal of Chemical Documentation* **5**, 107-113, doi:10.1021/c160017a018 (1965).

4 Segler, M. H. S., Preuss, M. & Waller, M. P. Planning chemical syntheses with deep neural networks and symbolic AI. *Nature* **555**, 604-610, doi:10.1038/nature25978 (2018).

5 Coley, C. W., Rogers, L., Green, W. H. & Jensen, K. F. Computer-Assisted Retrosynthesis Based on Molecular Similarity. *ACS Central Science* **3**, 1237-1245, doi:10.1021/acscentsci.7b00355 (2017).

6 Chen, B., Li, C., Dai, H. & Song, L. in *International Conference on Machine Learning (ICML).* (2020).

7 Tetko, I. V., Karpov, P., Van Deursen, R. & Godin, G. State-of-the-art augmented NLP transformer models for direct and single-step retrosynthesis. *Nature Communications* **11**, 5575, doi:10.1038/s41467-020-19266-y (2020).

8 Sutskever, I., Vinyals, O. & Le, Q. V. Sequence to Sequence Learning with Neural Networks. arXiv:1409.3215 (2014). <https://ui.adsabs.harvard.edu/abs/2014arXiv1409.3215S>.

9 Cadeddu, A., Wylie, E. K., Jurczak, J., Wampler-Doty, M. & Grzybowski, B. A. Organic Chemistry as a Language and the Implications of Chemical Linguistics for Structural and Retrosynthetic Analyses. *Angewandte Chemie International Edition* **53**, 8108-8112, doi:10.1002/anie.201403708 (2014).

10 Seo, S. *et al.* in *AAAI.*

11 Shi, C., Xu, M., Guo, H., Zhang, M. & Tang, J. in *Proceedings of the 37th International Conference on Machine Learning* Article 818 (JMLR.org, 2020).

12 Lin, Z., Yin, S., Shi, L., Zhou, W. & Zhang, Y. G2GT: Retrosynthesis Prediction with Graph to Graph Attention Neural Network and Self-Training. arXiv:2204.08608 (2022). <https://ui.adsabs.harvard.edu/abs/2022arXiv220408608L>.

13 Weininger, D. SMILES, a chemical language and information system. 1. Introduction to methodology and encoding rules. *Journal of Chemical Information and Computer Sciences* **28**, 31-36, doi:10.1021/ci00057a005 (1988).

14 Mao, K. *et al.* Molecular graph enhanced transformer for retrosynthesis prediction. *Neurocomputing* **457**, 193-202, doi:https://doi.org/10.1016/j.neucom.2021.06.037 (2021).

15 Tu, Z. & Coley, C. W. Permutation invariant graph-to-sequence model for template-free retrosynthesis and reaction prediction. *Journal of chemical information and modeling* (2022).

16 Jin, W., Coley, C. W., Barzilay, R. & Jaakkola, T. in *Proceedings of the 31st International Conference on Neural Information Processing Systems* 2604–2613 (Curran Associates Inc., Long Beach, California, USA, 2017).

17 Weisfeiler, B. & Leman, A. A reduction of a Graph to a Canonical Form and an Algebra Arising during this Reduction (in Russian). *Nauchno-Technicheskaya Informatsia* **9** (1968).

18 <https://downloads.emolecules.com/free/2023-04-01/> (

19 Sun, R., Dai, H., Li, L., Kearnes, S. M. & Dai, B. in *NeurIPS.*

20 Segler, M. H. S. & Waller, M. P. Neural-Symbolic Machine Learning for Retrosynthesis and Reaction Prediction. *Chemistry – A European Journal* **23**, 5966-5971, doi:https://doi.org/10.1002/chem.201605499 (2017).

21 Zheng, S., Rao, J., Zhang, Z., Xu, J. & Yang, Y. Predicting Retrosynthetic Reactions Using Self-Corrected Transformer Neural Networks. *Journal of Chemical Information and Modeling* **60**, 47-55, doi:10.1021/acs.jcim.9b00949 (2020).
22 Karpov, P., Godin, G. & Tetko, I. V. in *Artificial Neural Networks and Machine Learning – ICANN 2019: Workshop and Special Sessions.* (eds Igor V. Tetko, Věra Kůrková, Pavel Karpov, & Fabian Theis) 817-830 (Springer International Publishing).
23 Lin, K., Xu, Y., Pei, J. & Lai, L. Automatic retrosynthetic route planning using template-free models. *Chemical Science* **11**, 3355-3364, doi:10.1039/C9SC03666K (2020).
24 Kim, E., Lee, D., Kwon, Y., Park, M. S. & Choi, Y.-S. Valid, Plausible, and Diverse Retrosynthesis Using Tied Two-Way Transformers with Latent Variables. *Journal of Chemical Information and Modeling* **61**, 123-133, doi:10.1021/acs.jcim.0c01074 (2021).
25 Chilingaryan, G. *et al.* BARTSmiles: Generative Masked Language Models for Molecular Representations. *ArXiv* **abs/2211.16349** (2022).
26 Wan, Y., Liao, B., Hsieh, K. & Zhang, S. *Retroformer: Pushing the Limits of Interpretable End-to-end Retrosynthesis Transformer*. (2022).
27 Wang, X. *et al.* RetroPrime: A Diverse, Plausible and Transformer-based Method for Single-Step Retrosynthesis Predictions. *Chemical Engineering Journal* **420**, 129845, doi:10.1016/j.cej.2021.129845 (2021).
28 Zhong, Z. *et al.* Root-aligned SMILES: a tight representation for chemical reaction prediction. *Chemical Science* **13**, 9023-9034, doi:10.1039/D2SC02763A (2022).
29 Chen, S. & Jung, Y. Deep Retrosynthetic Reaction Prediction using Local Reactivity and Global Attention. *JACS Au* **1**, 1612-1620, doi:10.1021/jacsau.1c00246 (2021).
30 Dai, H., Li, C., Coley, C. W., Dai, B. & Song, L. in *Proceedings of the 33rd International Conference on Neural Information Processing Systems* Article 796 (Curran Associates Inc., 2019).
31 Somnath, V. R., Bunne, C., Coley, C., Krause, A. & Barzilay, R. in *Advances in Neural Information Processing Systems.* (eds M. Ranzato *et al.*) 9405-9415 (Curran Associates, Inc.).
32 Yan, C. *et al.* in *Advances in Neural Information Processing Systems.* (eds H. Larochelle *et al.*) 11248-11258 (Curran Associates, Inc.).
33 Ley, J., Krammer, G., Kindel, G. & Bertram, H.-J. 68-74 (2005).
34 Nair, J. B., Hakes, L., Yazar-Klosinski, B. & Paisner, K. Fully Validated, Multi-Kilogram cGMP Synthesis of MDMA. *ACS Omega* **7**, 900-907, doi:10.1021/acsomega.1c05520 (2022).
35 Roberto Bortolaso (Vicenza), M. S. V. Process for preparing [R-(R*,R*)]-5-(3-chlorophenyl)-3-[2-(3,4-dimethoxylphenyl)-1-methyl-ethy l]-oxazolidin-2-one 5663360 (1996).
36 Neudörffer, A. *et al.* Synthesis and Neurotoxicity Profile of 2,4,5-Trihydroxymethamphetamine and Its 6-(N-Acetylcystein-S-yl) Conjugate. *Chemical Research in Toxicology* **24**, 968-978, doi:10.1021/tx2001459 (2011).

REVIEWERS' COMMENTS

Reviewer #1 (Remarks to the Author):

Thanks for the authors for the revision and the responses, in which they address all my concerns. In particular, the results have been updated with canonicalized SMILES with atom mapping removed, which would have got rid of any possible information leak issue. The discussion with multi-step planning has also been made clearer to make the overall story consistent. The introduction section has been significantly expanded and the contextualization is now much better. The main result table has been reorganized/expanded and is now much easier to follow. There are a few minor issues/inconsistencies remaining (introduced by the added texts) but should only require some quick fixes. I would like to recommend acceptance of the revised manuscript and hope the authors will fix these small things in the final version.

Minor issues:

- Line 75: “Coley et al. used the fingerprinting …”. Note that this work is technically template-based too, as it still looks up the template associated with similar products to be applied onto the given target, rather than “search for appropriate reactants” directly. You might want to consider some minor reorganization for it to not fall under template-free/semi-template.

- Line 165: “To ensure the purchasability of the product”. I think the authors mean “synthesizability” of the product, or "purchasability" of the building blocks. If the product is purchasable then there’s no need for performing retrosynthesis on it.

- Table 1: LocalRetro is not sequence-based, and DualTF is sequence-based.

- Line 200: “our model achieved the highest accuracy, with a difference of 1% and 0.1% compared to the runner-up models”. It is not quite clear what the 1% and 0.1% correspond to, although the advantage of RetroExplainer over the other methods is clear from Table 1.

- Please double check the stage counting as in my original comment. There are still inconsistencies here, e.g., “five stages” in Line 181, “six-stage” in Line 264 which are followed by 5 bullets in Lines 267-280.

Additional comments:

- The discussion on novelty (e.g., improvement over existing methods) in the response is quite informative. In particular, the criticism on GraphRetro and its inability to handle multi-leaving group situation is fair.

- In the response, the author thinks that their model is permutation invariant and free from data leak. You need to be very careful when making this claim as such information leak can be very subtle and hard to detect. In particular, in your featurization (data_utils.get_atoms_info()), you include the chiral tags (atom.GetChiralTag()) which are not permutation invariant.

```
>>> Chem.MolToSmiles(Chem.MolFromSmiles("Br[C@H](Cl)C"))
'C[C@@H](Cl)Br'
```

See how the chiral tag for the central carbon can change after canonicalization. Experiments with properly canonicalized SMILES (which the author has done) are the easiest way, if not the only way to ensure no information leak, to the best of my knowledge.

Reviewer #2 (Remarks to the Author):

The authors significantly improved the manuscript after a round of revisions. The reported method is an interesting advance in the field of machine-learning-aided synthesis planning. The story became more cohesive, and the pictures became more insightful. The methodology is sound and the workflow is reproducible. I would recommend to accept the manuscript to Nature Communications.

Reviewer #3 (Remarks to the Author):

The authors have made a considerable effort in revising their manuscript, addressing the previous concerns raised in the review process. They have done an excellent job in refining the structure of the paper, improving the clarity of the figures, and correcting the typographical errors. They have also demonstrated a clear understanding of the feedback and have thoughtfully responded to each point, which is reflected in the overall improved quality and clarity of the manuscript.

The authors have improved the manuscript by moving less critical sections to the Supplementary Information, which emphasizes the RetroExplainer model and new deep learning modules. The updated model name in Figure 3 and the corrections in Figures 1b and 1d add consistency and rectify previous errors. The clarity of the figures has been enhanced, reflecting the authors' commitment to clear, effective communication. Lastly, the addition of a "Limitations" section provides a balanced perspective and hints at future research avenues.

Overall, the authors have significantly improved the manuscript, addressing all the concerns raised in the initial review. I agree with the previous reviewer's recommendation and also recommend the publication of this paper in Nature Communications. This work presents novel and significant contributions to the field, and the revisions have enhanced the clarity and quality of the presentation. The authors have demonstrated substantial efforts in responding to the feedback, and the results of their work will indeed be of great interest to the readers of Nature Communications.

Responses to Reviewers

Responses to Reviewer #1

Comments:

Thanks for the authors for the revision and the responses, in which they address all my concerns. In particular, the results have been updated with canonicalized SMILES with atom mapping removed, which would have got rid of any possible information leak issue. The discussion with multi-step planning has also been made clearer to make the overall story consistent. The introduction section has been significantly expanded and the contextualization is now much better. The main result table has been reorganized/expanded and is now much easier to follow. There are a few minor issues/inconsistencies remaining (introduced by the added texts) but should only require some quick fixes. I would like to recommend acceptance of the revised manuscript and hope the authors will fix these small things in the final version.

Minor issues:

- Line 75: "Coley et al. used the fingerprinting …". Note that this work is technically template-based too, as it still looks up the template associated with similar products to be applied onto the given target, rather than "search for appropriate reactants" directly. You might want to consider some minor reorganization for it to not fall under template-free/semi-template.

Author's response:

Thank you for your valuable feedback. We agree with your comment regarding the categorization of the work by Coley et al. as template-based rather than template-free/semi-template. Therefore, we have replaced the originally cited Coley's work as FeedForward EBM (FF-EBM) proposed by Chen et al.[1]. FF-EBM presents molecules using fingerprint and is independent from complete templates when using GLN[2] or RetroXpert[3] to provide the candidate precursors. Therefore we revised the related sentences as follows:

"Chen et al.[1] introduced the FeedForward EBM (FF-EBM) method, complemented by template-free models. FF-EBM leverages the fingerprinting technique to prioritize potential precursors."

Comments:

- Line 165: "To ensure the purchasability of the product". I think the authors mean "synthesizability" of the product, or "purchasability" of the building blocks. If the product is purchasable then there's no need for performing retrosynthesis on it.

Author's response:

We appreciate the reviewer's insightful comment. Indeed, you are correct in pointing out the potential confusion in our use of the term "purchasability." We intended to refer to the "synthesizability" of the product or the feasibility of procuring the necessary building blocks. Therefore, we have revised the manuscript to accurately reflect these concepts and ensure clear communication of our intended meaning. Thank you for your valuable input.

Comments:

- Table 1: LocalRetro is not sequence-based, and DualTF is sequence-based.

Author's response:

Thank you for bringing attention to the incorrect categorizations. We have taken your feedback into account and have reclassified LocalRetro as a graph-based model due to its utilization of graph representations. Similarly, we have categorized DualTF as a sequence-based model. Your input has helped us improve the accuracy of our classifications.

Comments:

- Line 200: "our model achieved the highest accuracy, with a difference of 1% and 0.1% compared to the runner-up models". It is not quite clear what the 1% and 0.1% correspond to, although the advantage of RetroExplainer over the other methods is clear from Table 1.

Author's response:

We apologize for any confusion caused by our previous statement. To clarify, the comparison mentioned is grounded in accuracy metrics averaged across top-1, top-3, top-5, and top-10 predictions. Specifically, the competing models—LocalRetro for familiar reaction types and R-SMILES for unfamiliar reaction types—achieved accuracies of 84.8% and 78.2% respectively. Meanwhile, our model's performances stand at 85.8% and 78.3% for the respective categories. Correspondingly, we have revised the corresponding sentences as:

"Moreover, considering the accuracies within the two scenarios outlined above (contingent on the provision of the reaction class), averaged across top-1, top-3, top-5, and top-10 predictions, our model achieved the highest accuracy, with a difference of 1% and 0.1% compared to the runner-up models, namely LocalRetro for known reaction types and R-SMILES for unknown reaction types, respectively."

We appreciate your valuable feedback.

Comments:

Please double check the stage counting as in my original comment. There are still inconsistencies here, e.g., "five stages" in Line 181, "six-stage" in Line 264 which are followed by 5 bullets in Lines 267-280.

Author's response:

We wish to clarify that our decision process entails six stages, and we have duly amended the corresponding statement from "five stages" to "six stages." The reason for presenting only five bullet points is due to the consolidation of the details of S-LGM and S-RCP under one bullet (the third bullet point), with the intention of eliminating redundancy. However, we acknowledge that

this consolidation may have caused some ambiguity. Consequently, we have included a reminder of this aspect within the third bullet point:

“Afterward, we calculated the energy of the IT stage by adding the energy of the selected LG at the S-LGM stage and the probabilities of the corresponding event predicted by the RCP-H, RCP-B, and LGM modules, respectively. It's important to note that this description encompasses two distinct stages: the S-LGM stage and the S-RCP stage.”

Comments:

- The discussion on novelty (e.g., improvement over existing methods) in the response is quite informative. In particular, the criticism on GraphRetro and its inability to handle multi-leaving group situation is fair.

- In the response, the author thinks that their model is permutation invariant and free from data leak. You need to be very careful when making this claim as such information leak can be very subtle and hard to detect. In particular, in your featurization (data_utils.get_atoms_info()), you include the chiral tags (atom.GetChiralTag()) which are not permutation invariant.

```
>>> Chem.MolToSmiles(Chem.MolFromSmiles("Br[C@H](Cl)C"))
'C[C@@H](Cl)Br'
```

See how the chiral tag for the central carbon can change after canonicalization. Experiments with properly canonicalized SMILES (which the author has done) are the easiest way, if not the only way to ensure no information leak, to the best of my knowledge.

Author’s response:

Thank you for your valuable feedback. We appreciate your acknowledgment of our discussion on novelty and the recognition of our critique concerning GraphRetro's limitations in handling multi-leaving group situations.

Regarding your cautionary note, we fully understand the fact about the inclusion of chiral tags (atom.GetChiralTag()) in our featurization process can lead to the violation such a permutation invariant. Given this point, we waive the assertion related to the permutation invariant properties. We thank your precious comment again.

Overall, we would like to express our gratitude for your recommendation for acceptance. Your review of our revisions and responses is greatly appreciated. It's heartening to know that our efforts have successfully addressed your concerns. Your guidance and feedback have been invaluable throughout this process, and we're truly thankful for your input.

Responses to Reviewer #2

Comments:

The authors significantly improved the manuscript after a round of revisions. The reported method is an interesting advance in the field of machine-learning-aided synthesis planning. The story became more cohesive, and the pictures became more insightful. The methodology is sound and the workflow is reproducible. I would recommend to accept the manuscript to Nature Communications.

Author's response:

We deeply appreciate your positive feedback on our revised manuscript. It's gratifying to know that our efforts have led to significant improvements in the clarity and cohesiveness of the narrative, as well as the quality of the visual aids. Thank you for your recommendation to have our manuscript published in Nature Communications. We value your thoughtful evaluation and your recognition of our work's contributions. Your feedback serves as motivation to continually refine our research and presentation.

Responses to Reviewer #3

Comments:

The authors have made a considerable effort in revising their manuscript, addressing the previous concerns raised in the review process. They have done an excellent job in refining the structure of the paper, improving the clarity of the figures, and correcting the typographical errors. They have also demonstrated a clear understanding of the feedback and have thoughtfully responded to each point, which is reflected in the overall improved quality and clarity of the manuscript.

The authors have improved the manuscript by moving less critical sections to the Supplementary Information, which emphasizes the RetroExplainer model and new deep learning modules. The updated model name in Figure 3 and the corrections in Figures 1b and 1d add consistency and rectify previous errors. The clarity of the figures has been enhanced, reflecting the authors' commitment to clear, effective communication. Lastly, the addition of a "Limitations" section provides a balanced perspective and hints at future research avenues.

Overall, the authors have significantly improved the manuscript, addressing all the concerns raised in the initial review. I agree with the previous reviewer's recommendation and also recommend the publication of this paper in Nature Communications. This work presents novel and significant contributions to the field, and the revisions have enhanced the clarity and quality of the presentation. The authors have demonstrated substantial efforts in responding to the feedback, and the results of their work will indeed be of great interest to the readers of Nature Communications.

Author's response:

We would like to express our sincere gratitude for your thorough review and thoughtful evaluation of our revised manuscript. Your feedback is immensely valuable to us, and we are pleased to see that our efforts in addressing the concerns raised during the review process have been recognized. Your recommendation to publish our manuscript in Nature Communications is a tremendous honour and serves as a motivating validation of our work. We are grateful for your kind words regarding the novelty and significance of our contributions to the field.

Reference

1 Lin, M. H., Tu, Z. & Coley, C. W. Improving the performance of models for one-step retrosynthesis through re-ranking. *Journal of Cheminformatics* **14**, 15, doi:10.1186/s13321-022-00594-8 (2022).

2 Dai, H., Li, C., Coley, C. W., Dai, B. & Song, L. in *NeurIPS.*

3 Yan, C. *et al.* in *Advances in Neural Information Processing Systems.* (eds H. Larochelle *et al.*) 11248-11258 (Curran Associates, Inc.).
